# Design and synthesis of benzodiazepines as brain penetrating PARP-1 inhibitors

**DOI:** 10.1080/14756366.2022.2053524

**Published:** 2022-03-22

**Authors:** Jiang Yu, Wenfeng Gou, Haihua Shang, Yating Cui, Xiao Sun, Lingling Luo, Wenbin Hou, Tiemin Sun, Yiliang Li

**Affiliations:** a Tianjin Key Laboratory of Radiation Medicine and Molecular Nuclear Medicine, Institute of Radiation Medicine, Peking Union Medical College, Chinese Academy of Medical Sciences, Tianjin, China; b Key Laboratory of Structure-Based Drug Design and Discovery, Shenyang Pharmaceutical University, Ministry of Education, Shenyang, China

**Keywords:** PARP-1 inhibitor, cancer, Rucaparib, benzodiazepine, CNS

## Abstract

The poly (ADP-ribose) polymerase (PARP) inhibitors play a crucial role in cancer therapy. However, most approved PARP inhibitors cannot cross the blood-brain barrier, thus limiting their application in the central nervous system. Here, 55 benzodiazepines were designed and synthesised to screen brain penetrating PARP-1 inhibitors. All target compounds were evaluated for their PARP-1 inhibition activity, and compounds with better activity were selected for further assays *in vitro*. Among them, compounds **H34**, **H42**, **H48**, and **H52** displayed acceptable inhibition effects on breast cancer cells. Also, computational prediction together with the permeability assays *in vitro* and *in vivo* proved that the benzodiazepine PARP-1 inhibitors we synthesised were brain permeable. Compound **H52** exhibited a B/P ratio of 40 times higher than that of Rucaparib and would be selected to develop its potential use in neurodegenerative diseases. Our study provided potential lead compounds and design strategies for the development of brain penetrating PARP-1 inhibitors.

**HIGHLIGHTS**
- Structural fusion was used to screen brain penetrating PARP-1 inhibitors.
- 55 benzodiazepines were evaluated for their PARP-1 inhibition activity.
- Four compounds displayed acceptable inhibition effects on breast cancer cells.
- The benzodiazepine PARP-1 inhibitors were proved to be brain permeable.

## Graphical Abstract

**Figure:**
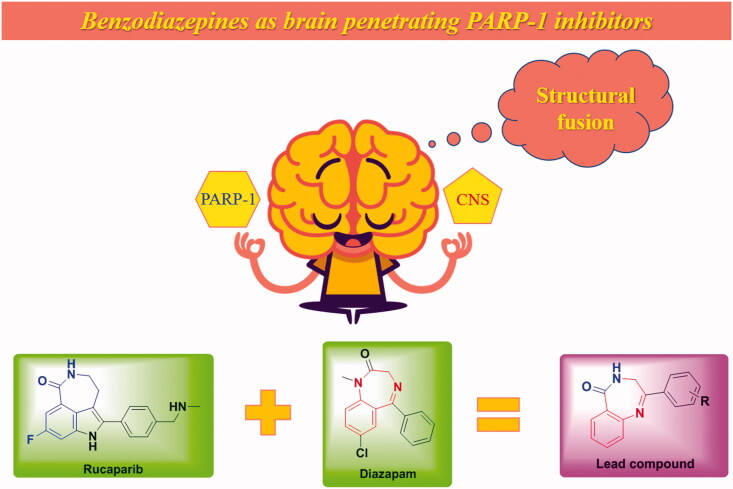

## 1. Introduction

**Figure 1.**
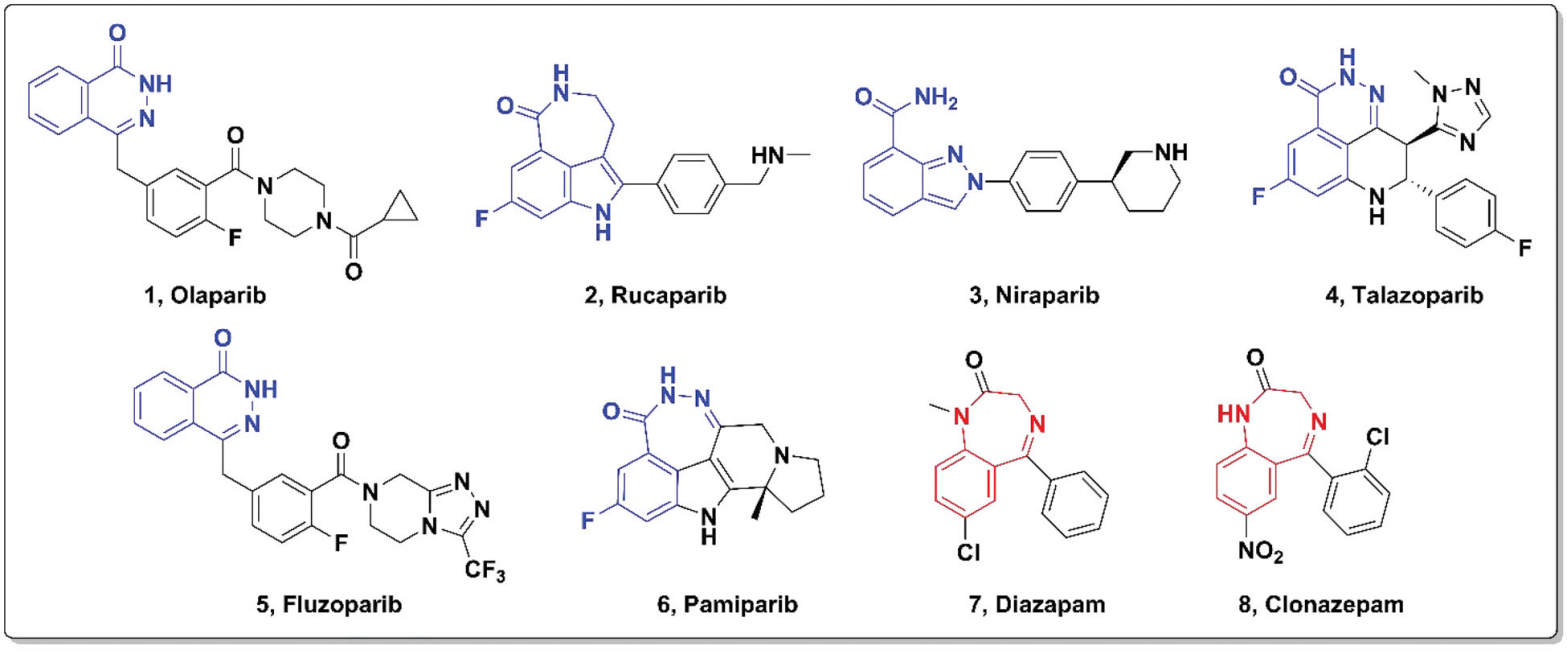
Approved PARP-1/2 inhibitors and representative benzodiazepine drugs.

Poly(ADP-ribose) polymerase-1 (PARP-1) is a nuclear protein that creates poly(ADP-ribose) (PAR) covalently attached to target proteins to mediate genomic stability, DNA damage repair, and apoptosis^1^. And PARP-1 is responsible for repairing DNA single-strand breaks (SSBs)^2^, whereas breast cancer susceptibility gene 1/2 (BRCA1/2) are key components involved in the repair of DNA double-strand breaks(DSBs) by homologous recombination (HR)^3^. Cancer cells with BRCA1/2 mutations treated with PARP inhibitors lead to cell cycle arrest and apoptosis through synthetic lethality^4^^,^^5^, thus the PARP-1 protein has become an attractive target for the treatment of cancers in recent years^6^. Since Olaparib^7^ was approved as the first PARP-1 inhibitor to treat advanced ovarian cancer associated with defective BRCA mutations in 2014, several PARP inhibitors were approved subsequently, including Rucaparib^8^, Niraparib^9^, Talazoparib^10^, Fluzoparib^11^^,^^12^, and Pamiparib^13^^,^^14^ (Figure 1).

Although PARP inhibitors play a crucial role in cancer therapy, effective therapy against the primary malignancy facilitates a more resilient systemic disease that leads to central nervous system (CNS) metastatic infiltration^15^. Breast cancer is the second-leading cause of metastatic disease in the CNS^16^. Patients with metastatic HER2^+^ primary cancer have a risk of 35–50% to develop metastatic brain disease^17^ and patients with adjuvant trastuzumab treatment have a risk of 2.5% to relapse in CNS firstly^18^. In addition, the risk of CNS involvement in metastatic triple-negative breast cancer (TNBC) is as high as 46%^19^. Similarly, primary CNS tumours, such as neurofibromatosis^20^, diffuse gliomas, medulloblastomas, and glioblastoma^21^, are also difficult to overcome because of the existence of the blood-brain barrier. However, several well-known PARP-1 inhibitors have been reported to be not CNS penetrant, including Olaparib^22^^,^^23^, Rucaparib^24^^,^^25^, and Talazoparib^26^. Pamiparib is a potent and selective PARP inhibitor with unique potential for the treatment of brain tumour, which is recently approved in China^13^^,^^14^. Nevertheless, more brain penetrating PARP-1 inhibitors are needed to meet the huge market.

PARP-1 is also known to participate in a range of important cellular processes including regulation of apoptosis, cell division and differentiation, transcriptional regulation, chromosome stability, and metabolic regulation^27^^,^^28^, and it has become an attractive therapeutic target for other diseases, such as neuroinflammation^29^, neurodegeneration^30^, cardiovascular disease^31^, and drug addiction^32^^,^^33^. To deal with these diseases, the factor of the blood–brain barrier (BBB) should also be considered^34^. In addition, the synergism between PARP-1 and various DNA damage response (DDR) targets is also being studied to trigger synthetic lethality artificially^35^. Lallo et al.^36^ combined Olaparib and the WEE1 inhibitor AZD1775 as a new therapeutic option for small cell lung cancer. Mak et al.^37^ reported that synergism between ataxia-telangiectasia mutated (ATM) and PARP-1 inhibition involves DNA damage and abrogating the G2 DNA damage checkpoint. Li et al.^38^ proposed that PARP inhibitors could enhance the priming and tumour-killing activities of T cells, boost the whole cancer-immunity cycle, and thereby improve the response to immune checkpoint blockade. The above-combined strategies show that PARP-1 inhibitors have a strong potential for the treatment of tumours, and the development of a new generation of PARP-1 inhibitors with high selectivity or brain penetration is helpful to make better use of its therapeutic potential. Based on the above facts, this paper is devoted to the study of CNS penetrant PARP-1 inhibitors, which are expected to meet the diverse needs of clinical treatment in the future.

## 2. Materials and methods

### 2.1. Compound design

**Figure 2.**
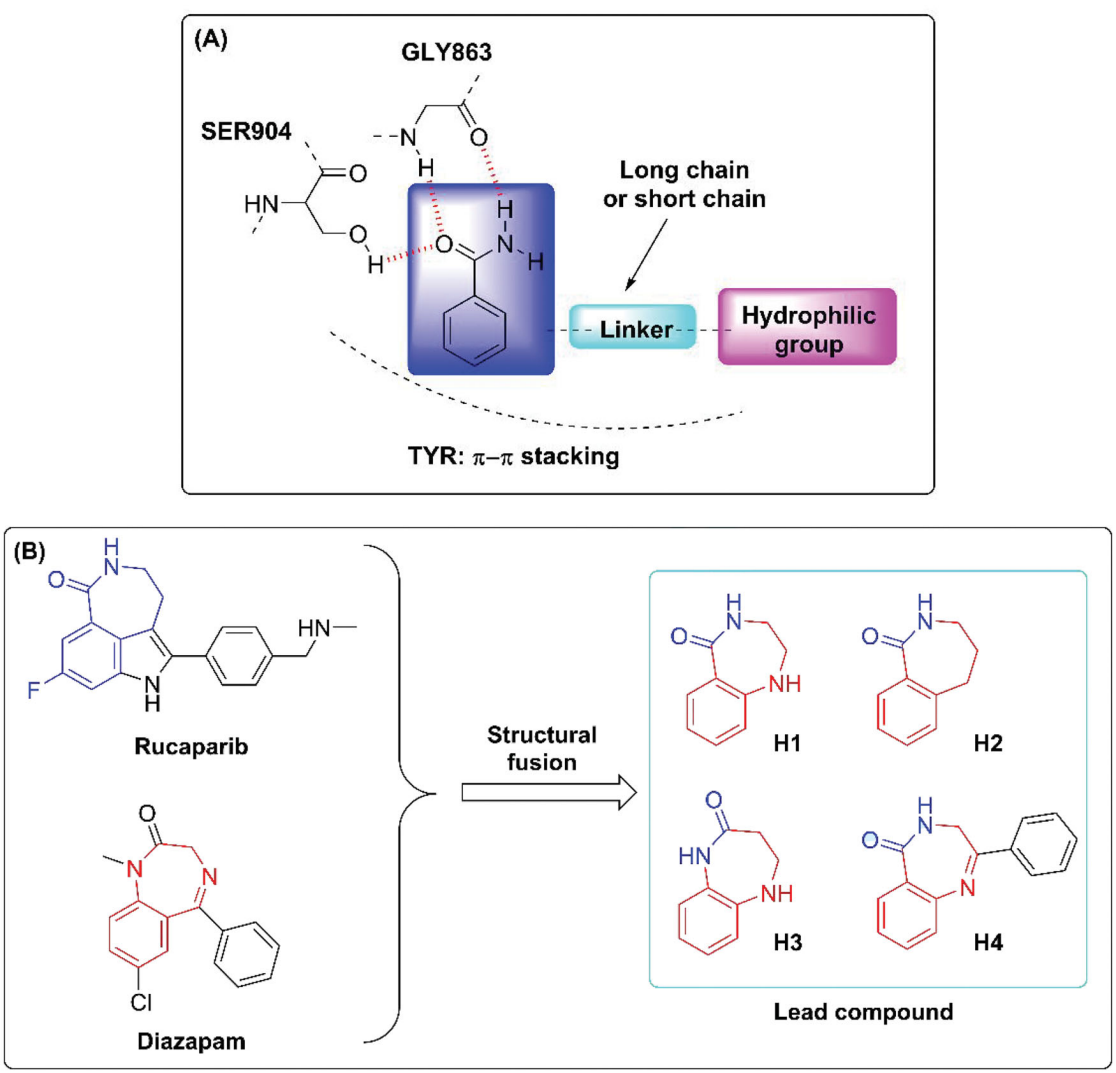
(A) Binding mode of PARP-1 inhibitors. (B) Drug design strategy.

**Figure 3.**
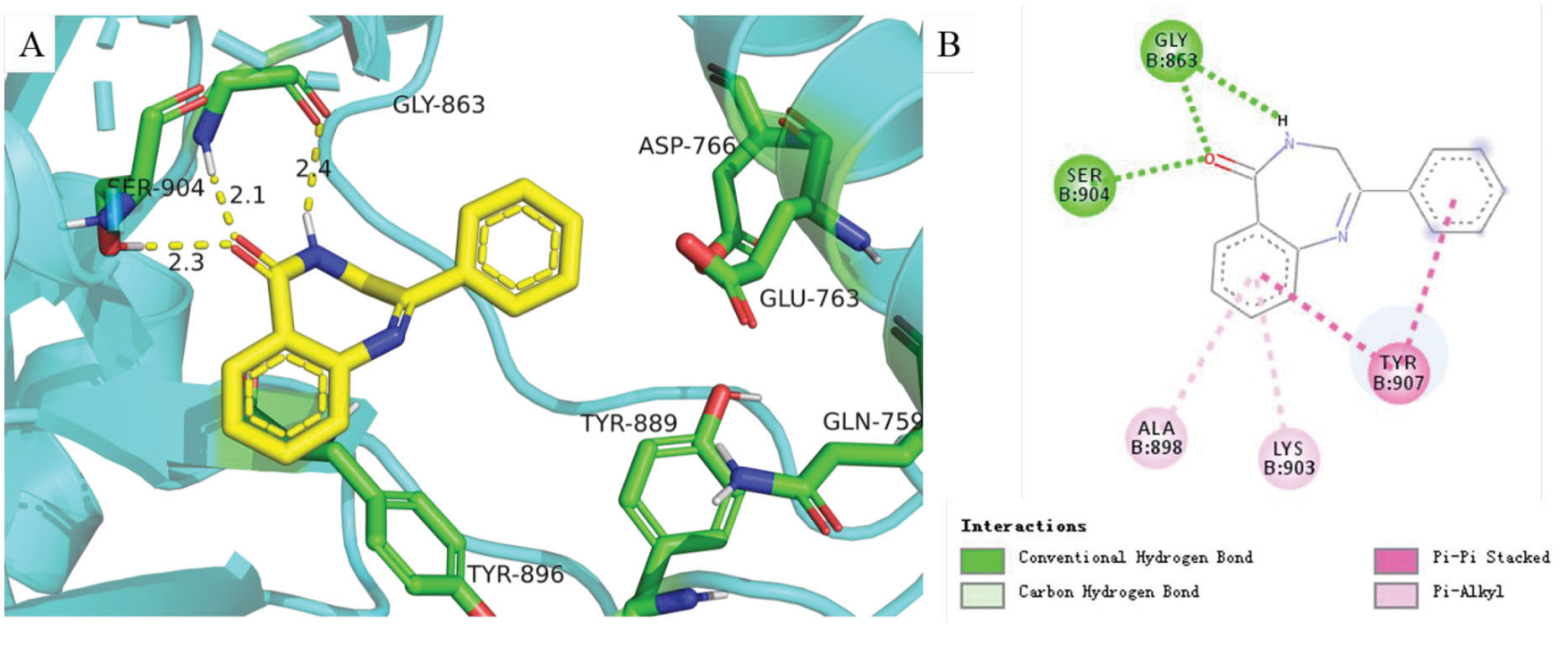
(A) Predicted binding mode of **H4** with PARP-1. (B) 2D diagram of **H4** interacting with PARP-1. The catalytic domain structure of PARP-1 is coloured in cyan (crystal structure: PARP-1 PDB code 4zzz). Compounds and the key residues within the binding pocket are shown as sticks and coloured in yellow and green, respectively. Hydrogen bonds are shown as yellow and the distances (Å) of H-bonds are also labelled. The molecular docking was accomplished using Glide flexible docking. The 3D images were prepared with PyMOL, and the 2D picture was drawn in Discovery Studio.

PARP inhibitors have been developed for several years and the efforts of predecessors have led to a good understanding of key pharmacophores for the binding of small molecules to PARP-1. Most PARP inhibitors are designed to mimic the nicotinamide moiety of natural substrate NAD^+^ and bind competitively with NAD^+^ at the NI site of the PARP-1 catalytic domain^6^. Key hydrogen bonding interactions between the inhibitors and residues SER904 and GLY863 of PARP-1 are crucial for activity retention^39^ (Figure 2). The π–π stacking formed between the ligand and the nearby Tyr residue is also essential. The strategies of restraint in amide rotation are designed either by constraining the amide into a cyclic amide, such as Rucaparib and Olaparib, or by forming a pseudo cyclic amide via an intramolecular H-bond as in Niraparib^40^. To our delight, benzodiazepines have been widely concerned by pharmaceutical chemists because of their effective penetration of the blood-brain barrier and significant inhibition of the central nervous system^41^, such as Diazepam and Clonazepam, which are all classical benzodiazepines (Figure 1). Accordingly, the pharmacophore of Rucaparib and benzodiazepine structure were fused in this paper to find brain penetrant PARP inhibitors (Figure 2), and preliminary compounds **H1**, **H2**, **H3**, and **H4** were obtained. As shown in Figure 3, compound **H4** exhibited similar interactions with known PARP-1 inhibitors and was used for subsequent structural optimisation.

### 2.2. Chemistry

**Scheme 1.**
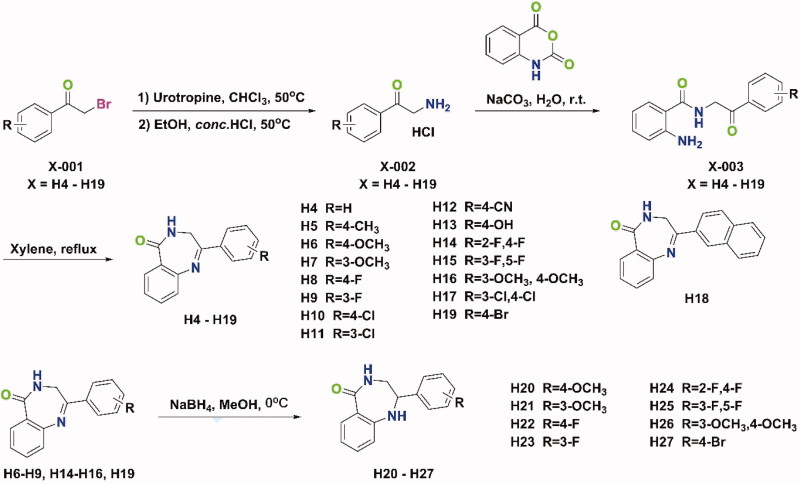
Synthetic route of compounds **H4–H27**.

**Scheme 2.**
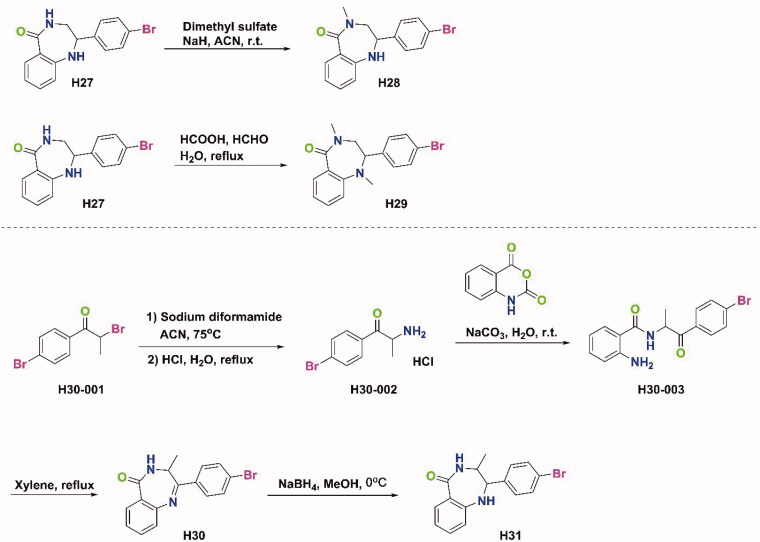
Synthetic route of compounds **H28–H31**.

**Scheme 3.**
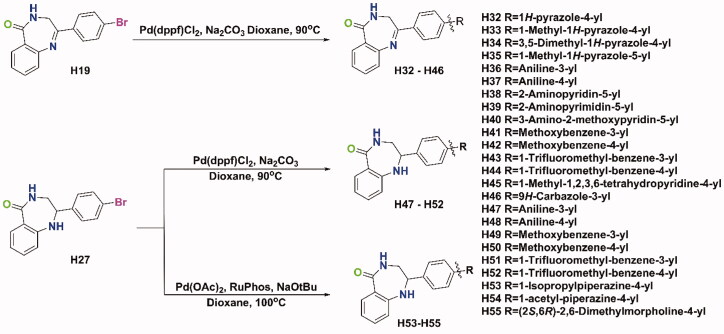
Synthetic route of compounds **H32–H55**.

The syntheses of all target compounds were accomplished according to the synthetic routes outlined in Schemes 1–3. The first series of 2-phenyl-3,4-dihydro-5*H*-benzo[e][1,4]diazepin-5-one derivatives were synthesised in three steps as shown in Scheme 1. Firstly, a variety of 2-bromo-1-phenylethanone derivatives (**X-001**) were used as starting materials to react with urotropine, and then the quaternary ammonium salt was hydrolysed in acid to synthesise primary amine hydrochloride (**X-002**). Then, the key intermediates (**X-003**) were synthesised in good yields by the reaction of these primary amine hydrochlorides with isatoic anhydride at room temperature. Finally, **H4–H19** were obtained by refluxing the above intermediates in xylene. After reduction with NaBH_4_ in methanol at 0 °C, compounds **H20–H27** were prepared. As shown in Scheme 2, compounds **H28** and **H29** were prepared by different methylation conditions. Sodium diformylamide was used to get **H30-002**, then compound **H30** and **H31** were also obtained by the above similar reactions. With **H19** and **H27** in hand, the target compound **H32–H55** were synthesised through Suzuki Coupling or Buchwald-Hartwig Coupling as shown in Scheme 3.

## 3. Results and discussion

### 3.1. Enzyme inhibition activity against PARP-1 and structure-activity relationship

All target compounds were evaluated for PARP-1 enzyme inhibition activity at the concentration of 50 nM determined by previous pre-experiments.

#### 3.1.1. Inhibitory activities of compounds H1–H19 against PARP-1

As shown in Table 1, the results of compound **H1–H4** show that the phenyl substituent improves the activity, which may form π–π stacking similar to that of Rucaparib. Because of its preliminary activity, **H4** was selected for structural optimisation. Then, **H5–H11** were obtained by introducing methyl, methoxy, fluorine, and chlorine into the benzene ring of **H4**, and they all showed slightly increased PARP-1 inhibitory activity except **H11**. For compounds **H6–H9**, the activities of meta-substitution were higher than para-substitution, while chlorine substituted **H10–H11** showed the opposite, indicating that para-substitution was more beneficial to the activity when the volume of substituents is larger. **H12** and **H13** were obtained by introducing polar cyano and hydroxyl groups into the para-position. **H12** dramatically decreased enzyme inhibition potency, while **H13** maintained the enzyme activity. Considering that fluorine, methoxy, and chlorine substitutions help to improve the activity and lipophilicity of the compounds, the corresponding disubstituted compounds **H14–H17** were synthesised. However, compounds **H14**, **H15**, and **H17** maintained parallel activity, while **H16** exhibited decreased potency. The benzene ring was replaced by naphthalene ring to obtain **H18**, which showed similar activity, indicating that the introduction of large substituents would not cause the decrease of activity. To introduce diverse substituents through the classical coupling reactions, compound **H19** containing bromine substitution was synthesised with an inhibition rate of 38.4%.

**Table 1.** The chemical structures and inhibitory activities of compounds **H1–H19**.

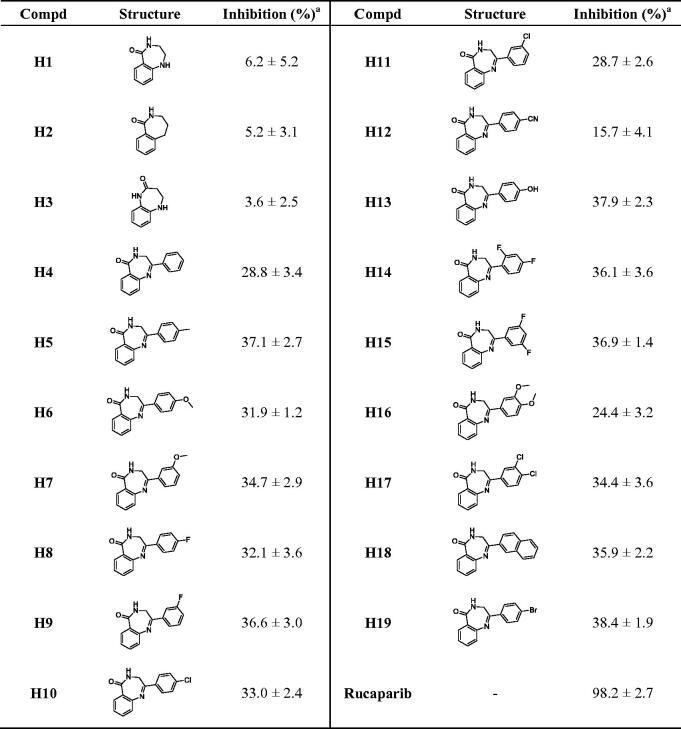	

^a^The inhibition (%) was evaluated at the compound concentration of 50 nM and values were calculated from the results of three independent tests.

#### 3.1.2. Inhibitory activities of compounds H20–H31 against PARP-1

To obtain better lead compounds, we investigated the structure-activity relationship of different variants of benzodiazepine structure. The inhibition rates are shown in Table 2. Compounds **H20**–**H27** were obtained by the reduction of the imines in compounds **H6**–**H9**, **H14**–**H16**, and **H19**. Compared with the corresponding unreduced compounds, the activities of **H20**–**H24** decreased slightly, while the activities of compounds **H25** and **H27** increased. Compound **H26** regained acceptable activity, which may be due to the increase of molecular flexibility after reduction. Compounds **H28** and **H29** were synthesised from compound **H27** by nitrogen methylation reaction, and the decreased inhibition indicated that the hydrogen of amide was necessary for PARP-1 inhibitory activity, which was consistent with the previously reported structure-activity relationship. When methyl was introduced into the ortho position of amide nitrogen in compound **H19**, the obtained compound **H30** showed no obvious change in activity. However, the activity of the reduction product **H31** decreased obviously, which may be due to the excessive flexibility of the seven-membered ring. For this series of compounds, the conclusions are as follows: (i) the reduction of imine structure has little effect on the activity and increases the flexibility of the compound; (ii) hydrogen on amide nitrogen is necessary for activity, and the introduction of substituents leads to the decrease of activity; (iii) the introduction of methyl into the ortho position of amide nitrogen has no effect on the activity on the rigid ring but has a greater effect on the flexible ring. Based on the above results, compounds **H19** and **H27** display the highest activity, and their bromine substitution is conducive to the introduction of various substituents through coupling reactions, which can be used as new lead compounds for follow-up optimisation.

**Table 2.** The chemical structures and inhibitory activities of compounds **H20–H31**.

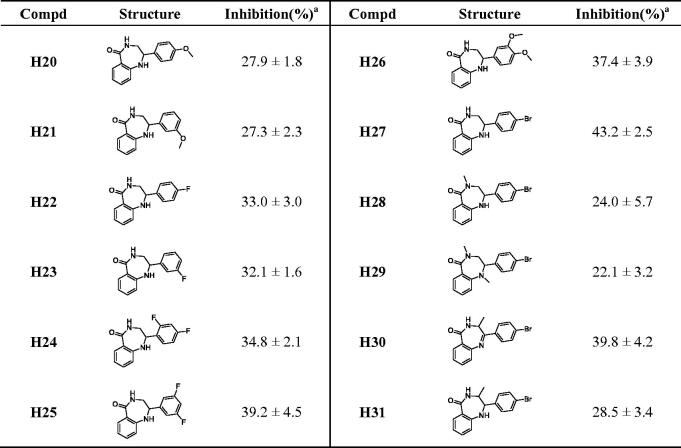	

^a^The inhibition (%) was evaluated at the compound concentration of 50 nM and values were calculated from the results of three independent tests.

#### 3.1.3. Inhibitory activities of compounds H32–H46 against PARP-1

According to our previous study^42^, three key amino acids in PARP-1 (GLN759, GLU763, ASP766) were found, which formed an acidic cavity and specifically contained the hydrophilic group. Therefore, compounds containing exposed amino groups were synthesised to match the acidic domain of the PARP-1 active pocket (Table 3). With **H19** in hand, compounds **H32–H35** were obtained by coupling with pyrazole and its derivatives, and their inhibition rates were significantly higher than that of **H19** at the same concentration, among which compound **H34** reached 56.7%. The activities of **H36** and **H37** synthesised by replacing pyrazole with aniline were only slightly improved. Increasing the number of heteroatoms on the aniline to get **H38–H40**, which exhibited decreased enzyme inhibition potency. It is considered that the increased number of heteroatoms will increase the polar surface area of the compound, which is not conducive to crossing the blood-brain barrier. Thus, compounds **H41–H44** were obtained by replacing the amino group on the benzene ring with methoxy and trifluoromethyl to improve the lipophilicity of the compound. The results show that the position of the methoxy group has a great influence on the activity, and the inhibition rate of para-substituted compound **H42** was 51.2%. On the contrary, trifluoromethyl substituted compounds showed similar activity. The results of **H36–H37** and **H41–H44** show that the activity of para-substitution is generally better than that of meta-substitution with the increase of length. Compound **H45** was obtained by replacing benzene with 1-methyl-1,2,3,6-tetrahydropyridine, and the activity was not significantly improved. **H46** was obtained by the introduction of carbazole, and the loss of activity indicated that the size of substituents needs to be controlled to avoid the decrease of activity caused by steric hindrance.

**Table 3.** The chemical structures and inhibitory activities of compounds **H32–H46**.

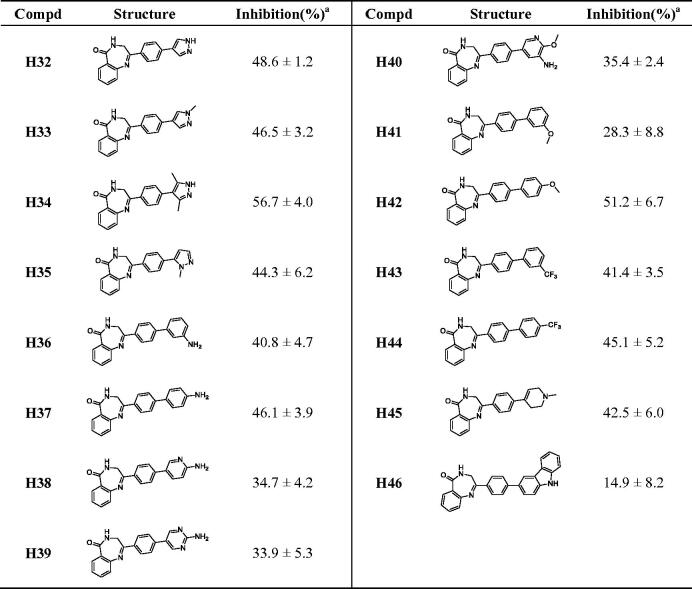	

^a^The inhibition (%) was evaluated at the compound concentration of 50 nM and values were calculated from the results of three independent tests.

#### 3.1.4. Inhibitory activities of compounds H47–H55 against PARP-1

The activity of **H27** is higher than that of **H19**, and its imine structure is more stable after reduction. Therefore, a series of compounds were synthesised based on **H27**, the results were shown in Table 4. The compounds **H47–H52** were obtained by coupling amino, methoxy and trifluoromethyl substituted benzene with **H27**, and their inhibition rates were higher than that of unreduced **H36–H37** and **H41–H44**. In addition, it was also concluded that para-substitution was better than meta-substitution, and the inhibition rates of **H48**, **H50**, and **H52** were all more than 50%. Unexpectedly, the activities of compounds **H53–H55** substituted by 1-isopropylpiperazine, 1-acetylpiperazine, and (2S, 6R)-2,6-dimethylmorpholine were lower than that of their lead compound **H27**, indicating that saturated heterocycles were not conducive to maintaining activity.

**Table 4.** The chemical structures and inhibitory activities of compounds **H47–H55**.

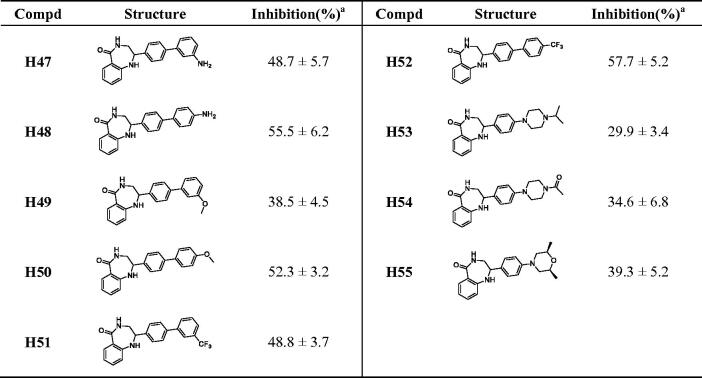	

^a^The inhibition (%) was evaluated at the compound concentration of 50 nM and values were calculated from the results of three independent tests.

To sum up, through the study of structure–activity relationship, five compounds (**H34** 56.7%, **H42** 51.2%, **H48** 55.5%, **H50** 52.3%, **H52** 57.7%) were obtained with an inhibition rate of more than 50%. Then, the IC_50_ values of compounds **H34**, **H42**, **H48**, **H50**, and **H52** against PARP-1 were determined, as shown in Table 5. Although their enzyme activity is weaker than that of the control drug Rucaparib, we think it is necessary to make a further evaluation, especially brain permeability, which is the focus of this article. And lower activity may also be allowed when such inhibitors are used as a combination strategy to treat tumours or alleviate neurological diseases. PARP-2 is the closest homolog of PARP-1, which also resides in the nucleus and functions similarly to PARP-1. The IC_50_ values of the compounds against PARP-2 were also determined (Table 5). In addition, compounds **H19** and **H27** have preliminary PARP-1 inhibitory activity and active reaction groups, and further structural optimisation of the compounds or analogues as lead compounds to find more active PARP-1 inhibitors will also be carried out.

**Table 5.** IC_50_ values of compounds **H34**, **H42**, **H48**, **H50**, and **H52** against PARP-1

Compound	IC_50_ (nM)		Compound	IC_50_ (nM)	
	PARP-1	PARP-2		PARP-1	PARP-2
Rucaparib	0.97	1.82	**H48**	37.8	40.7
**H34**	41.4	51.2	**H50**	46.3	45.1
**H42**	40.3	47.1	**H52**	30.2	39.6

### 3.2. Molecular docking

**Figure 4.**
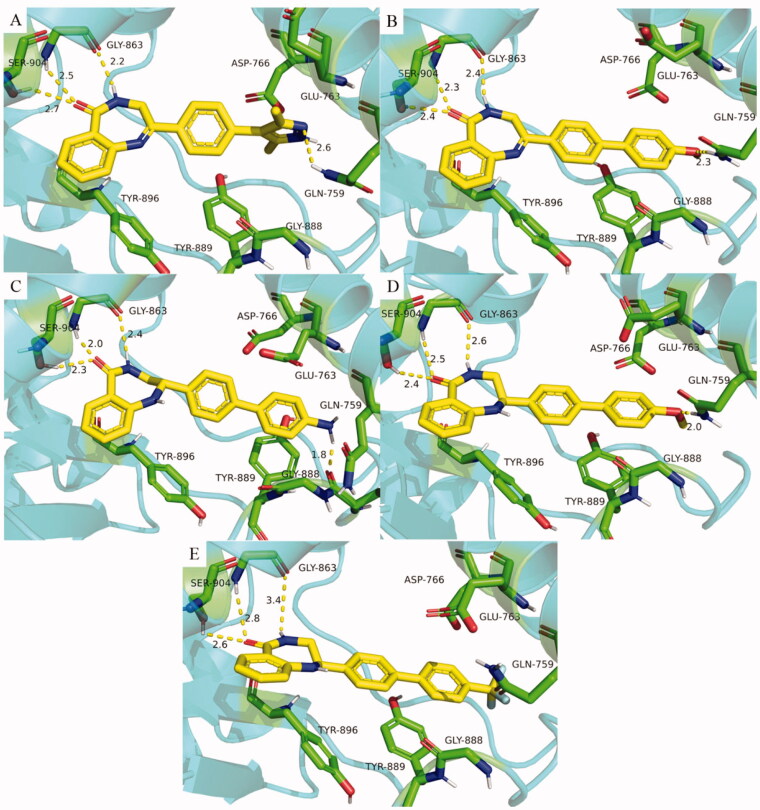
Predicted binding poses of compounds with PARP-1 (A: **H34**, B: **H42**, C: **H48**, D: **H50**, E: **H52**). The catalytic domain structure of PARP-1 is coloured in cyan (crystal structure: PARP-1 PDB code 4zzz). Compounds and the key residues within the binding pocket are shown as sticks and coloured in yellow and green, respectively. Hydrogen bonds are shown as yellow and the distances (Å) of H-bonds are also labelled. The molecular docking was accomplished using Glide flexible docking. The 3D images were prepared with PyMOL.

To rationalise the observed moderate activity of compounds **H34**, **H42**, **H48**, **H50**, and **H52**, we next obtained the possible binding patterns from molecular docking results. As shown in Figure 4, the three key hydrogen bonding interactions were formed between the five compounds and SER904 and GLY863 of PARP-1, which were necessary for activity and consistent with the binding patterns of Rucaparib. In addition, the pyrazole group of **H34** formed an additional hydrogen bond with GLN759, the phenolic group of **H42** and **H50** also form additional hydrogen bonds with GLN759. However, the aniline of **H48** formed an additional hydrogen bond with GLY888, while the trifluoromethyl of **H52** stayed in the cavity formed by ASP766, GLU763, and GLN759. These interactions mentioned above theoretically explained the moderate PARP-1 inhibitory activity of the benzodiazepines synthesised in this paper.

### 3.3. Inhibition of cellular proliferation

Due to their acceptable inhibition rates (more than 50%) at the concentration of 50 nM, compounds **H34**, **H42**, **H48**, **H50**, and **H52** were selected to evaluate the proliferation inhibition activity on several cancer cells, including BRCA1 mutant tumour cells (MX-1) and BRCA1 wild type tumour cells (MCF7 and A549). Rucaparib was used as the reference molecule and the results were outlined in Table 6. The results showed that all the five compounds showed a certain inhibitory effect on proliferation, and were weaker than those of Rucaparib, especially **H50**. In addition, the inhibitory effect of **H34**, **H42**, **H48**, and **H52** on breast cancer cells was stronger than that of lung cancer cells, and the compounds showed slightly stronger effects on MX-1 cells than MCF7 cells.

**Table 6.** Antiproliferation activity of compounds **H34**, **H42**, **H48**, **H50**, and **H52** in tumour cell lines.

Compound	IC_50_ (μM)		
	MX-1(BRCA1-)^a^	MCF7^b^	A549^c^
**H34**	40.3 ± 3.3	44.3 ± 5.2	120.7 ± 16.2
**H42**	31.4 ± 2.8	39.4 ± 4.2	162.4 ± 18.4
**H48**	30.7 ± 4.0	35.7 ± 2.9	176.4 ± 23.1
**H50**	130.2 ± 10.7	125.3 ± 13.2	>200
**H52**	24.6 ± 3.7	37.9 ± 3.5	157.1 ± 15.2
Rucaparib	5.5 ± 2.2	20.2 ± 3.1	34.9 ± 5.2

^a^Breast cancer cell line.

^b^Breast cancer cell line.

^c^Lung cancer cell line.

### 3.4. Prediction of ADME properties

The computational prediction of absorption, distribution, metabolism, and excretion (ADME) was used in evaluating the brain permeability of synthesised compounds. Several parameters of four compounds (**H34**, **H42**, **H48**, and **H52**) were displayed in Table 7, which were often used in the evaluation of blood-brain barrier and permeability. These include polar surface area (PSA), octanol/water partition coefficient (QPlogPo/w), central nervous system activity (CNS), brain/blood partition coefficient (QPlogBB), apparent MDCK cell permeability (QPPMDCK), apparent Caco-2 cell permeability (QPPCaco). Based on the above parameters, the prediction results show that compounds **H42** and **H52** have good brain permeability, while **H34**, **H48**, and Rucaparib are difficult to pass through the blood-brain barrier.

**Table 7.** Predicted ADME properties of compounds **H34**, **H42**, **H48**, and **H52**.

Compound	PSA	QPlogPo/w	CNS	QPlogBB	QPPMDCK	QPPCaco
**H34**	87.69	3.32	−1	−0.86	201	434
**H42**	65.94	4.37	0	−0.43	782	1528
**H48**	78.38	2.99	−2	−1.04	159	350
**H52**	52.08	4.96	0	−0.13	2992	1342
Rucaparib	68.26	2.80	0	−0.15	182.86	211.03
Standard range	7.0–200.0	−2.0–6.5	−2 to +2	−3.0–1.2	<25 poor, >500 great	<25 poor, >500 great

### 3.5. MDCK cell permeability

The MDCK cells monolayer model was also used to determine whether the four compounds were capable of crossing the blood-brain barrier^43^^,^^44^. The cell culture was carried out in accordance with routine operations or instructions. The MDCK monolayer model was established after 5 days of culture, and then the trans-epithelial electrical resistance (TEER) was measured using an EVOM epithelial volt-ohmmeter with STX2 electrodes to evaluate the integrity of the membrane. The tested compounds (10 µM) of 100 µL were added to the apical compartment of the transwell cell culture devices (Millipore), and then the devices were incubated at 37 °C for 3 h. The concentrations of tested compounds in an apical compartment and basolateral compartment were detected by HPLC. The apparent permeability in an apical compartment to the basolateral compartment (*P*_app_ (A→B)) was then calculated^45^, and the results are listed in Table 8. Rucaparib was not detected in the basolateral compartment, which was consistent with the literature that it could not pass through the blood-brain barrier^24^^,^^25^. The four compounds we synthesised all showed the permeability of the monolayer model, and the *P*_app_ (A→B) value of compound **H52** was the highest, reaching 19.7 cm/s × 10^−6^.

**Table 8.** MDCK cell permeability of compounds **H34**, **H42**, **H48**, and **H52**.

Compound	*C*_apical_ (μg/mL)^a^	*C*_basolateral_ (μg/mL)^b^	*P*_app_ (A→B) (cm/s × 10^−6^)^c^
**H34**	1.65 ± 0.32	0.31 ± 0.08	15.7 ± 1.32
**H42**	1.62 ± 0.37	0.33 ± 0.05	16.1 ± 1.08
**H48**	2.43 ± 0.42	0.23 ± 0.06	11.7 ± 0.51
**H52**	1.65 ± 0.05	0.45 ± 0.07	19.7 ± 0.92
Rucaparib	3.39 ± 0.82	ND	–

ND: not detected.

^a^The concentration of compounds in the apical compartment.

^b^The concentration of compounds in the basolateral compartment.

^c^*P*_app_ (A→B) = (Δ*Q*/Δ*t*)/(*A*·*C*_0_), the detailed information is displayed in the experiment section.

### 3.6. Brain/plasma pharmacokinetic studies in vivo

Further, the brain permeability of representative compounds was evaluated in ICR mice. After tail vein injection of a 1.5 mg/kg dose, the concentrations of the compounds in the brain and plasma were measured after 5 min and 60 min of administration, and the brain/plasma (B/P) ratio was calculated. As shown in Table 9, compounds **H34** and **H48** exhibited low B/P ratios in 5 min and 60 min, which were similar to that of Rucaparib. On the contrary, compounds **H42** and **H52** performed high B/P ratios, which were consistent with our initial expectations. Among them, the B/P ratio of **H42** reached balance rapidly *in vivo* and maintained above 3, while the proportion of **H52** in the brain increased gradually and reached 4.41 at 60 min. In addition, **H42** showed a low concentration in plasma and brain after administration of 60 min, while the concentration of **H52** was still high, indicating that **H52** was more likely to accumulate *in vivo*.

**Table 9.** Brain penetration study for compounds **H34**, **H42**, **H48**, and **H52**^a^.

Compound	Time (min)	Plasma (ng/mL)	Brain (ng/g)	B/P ratio (fold)
**H34**	5	3124.95 ± 220.45	300.68 ± 52.51	0.10 ± 0.01
	60	1054.90 ± 70.31	9.67 ± 8.75	0.01 ± 0.01
**H42**	5	554.97 ± 104.39	1816.17 ± 323.95	3.28 ± 0.28
	60	15.07 ± 5.44	51.83 ± 13.62	3.58 ± 0.53
**H48**	5	2645.17 ± 264.07	1051.48 ± 153.23	0.40 ± 0.05
	60	157.38 ± 32.30	10.05 ± 0.87	0.07 ± 0.02
**H52**	5	718.73 ± 39.93	1571.63 ± 264.39	2.18 ± 0.27
	60	597.12 ± 24.96	2633.52 ± 118.5	4.41 ± 0.07
Rucaparib	5	1175.43 ± 121.86	169.73 ± 48.50	0.14 ± 0.04
	60	160.75 ± 60.49	13.93 ± 3.78	0.10 ± 0.06

^a^The samples were collected at 5 and 60 min after tail vein injection of 1.5 mg/kg compounds in ICR mice. The results are depicted as the mean ± *SD* of six independent experiments.

**Figure 5.**
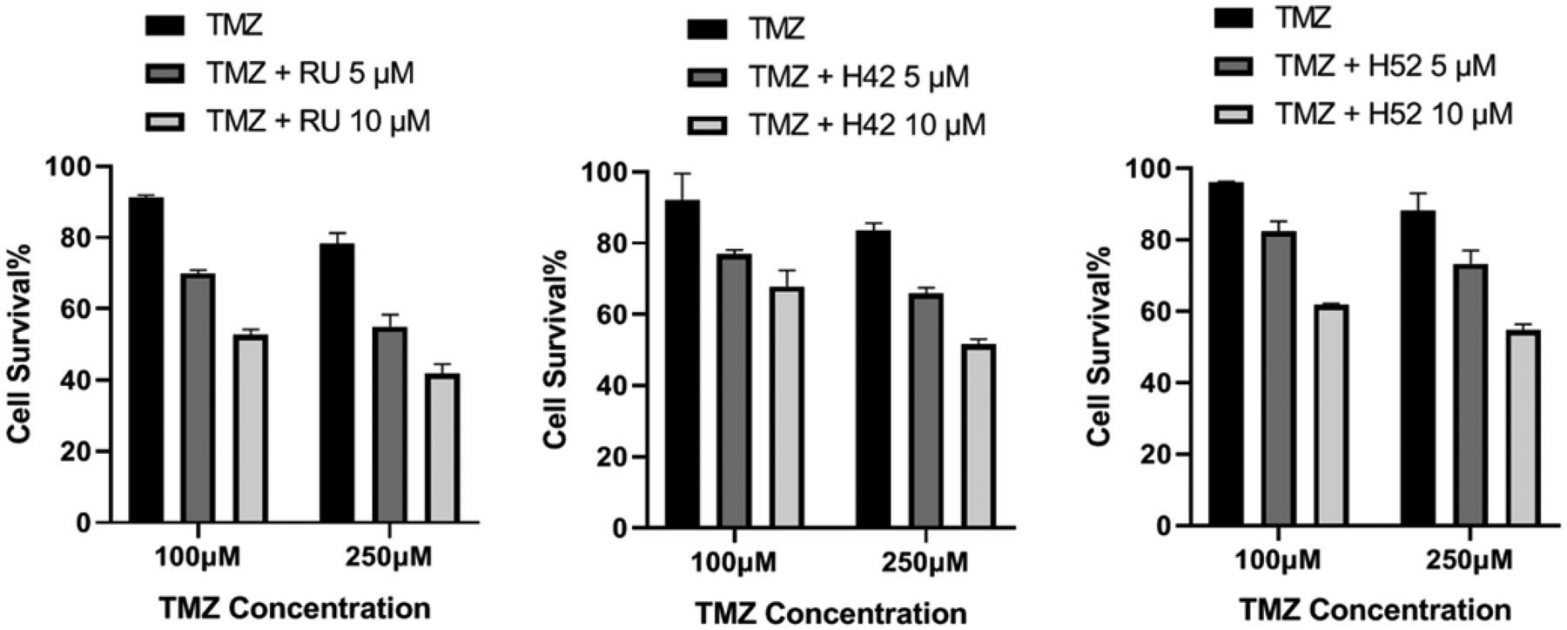
The cytotoxicity of **H42** and **H52** in combination with TMZ in glioblastoma (U87) cells. TMZ: Temozolomide; RU: Rucaparib.

Based on the above results, compounds **H42**, **H52**, and Rucaparib were then selected to evaluate their antiproliferation activity in combination with TMZ on glioblastoma (U87) cells. As shown in Figure 5, both **H42** and **H52** showed significant sensitisation effects in U87 cells.

## 4. Conclusions

Blood–brain barrier protects the central nervous system from the interruption and damage of xenobiotics. However, it prevents potential drugs aimed at the central nervous system, thus becomes an obstruction for the development of central nervous system drugs. The PARP inhibitors have played a crucial role in cancer therapy. However, most approved PARP inhibitors cannot cross the blood-brain barrier, thus limiting their application in the central nervous system. In this study, 55 benzodiazepines were designed and synthesised to screen brain penetrating PARP-1 inhibitors. All target compounds were evaluated for their PARP-1 inhibition activity, and compounds with high inhibition rates at 50 nM were selected to assess for cellular assays *in vitro*. Among them, compounds **H34**, **H42**, **H48**, and **H52** displayed acceptable inhibition effects on breast cancer cells. Also, computational prediction and the permeability assays *in vitro* and *in vivo* proved that the benzodiazepine PARP-1 inhibitors we synthesised are brain permeable. Compound **H52** exhibited acceptable PARP-1 inhibition and good brain penetration, and its B/P ratio was more than 40 times higher than that of Rucaparib. It has been reported in *Science* that strategies aimed at inhibiting PARP-1 activation could hold promise as a disease-modifying therapy to prevent the loss of dopamine neurons in Parkinson’s disease and related a-synucleinopathies^46^. Therefore, in addition to improving the anti-tumour activity of these benzodiazepine PARP-1 inhibitors, compound **H52** would be selected to develop its potential use in neurodegenerative diseases. Overall, our study provided potential lead compounds and design strategies for the development of brain penetrating PARP-1 inhibitors.

## 5. Experimental section

### 5.1. Chemistry

#### 5.1.1. General methods

All reagents and solvents were obtained from commercial suppliers and used without further purification. All reactions were performed in glassware containing a Teflon coated stir bar. The reactions were monitored by thin-layer chromatography (TLC) using silica gel GF254 and spots were visualised by UV light at 254 or 365 nm. Flash column chromatography on silica gel (200–300 mesh) was used for the routine purification of reaction products. Since the reactions were carried out at the millimole level, all yields have not been accurately calculated. The purity of the target compounds determined by high-performance liquid chromatography was all higher than 95%. The intermediates and the products were fully characterised by spectroscopic data. ^1^H and ^13^C NMR spectra were recorded with Bruker BioSpin GmbH at 400 and 100 MHz, respectively. DMSO-*d6* and CDCl_3_ were selected as deuterated solvents. Chemical shifts were expressed in δ (ppm) and coupling constants were given in Hz. Low-resolution mass spectra were obtained from Waters 3100.

#### 5.1.2. Synthesis procedure for X-002

##### 5.1.2.1. 2-Amino-1-phenylethanone hydrochloride (H4-002)

2-Bromo-1-phenylethanone (**H4-001**) (8 mmol, 1 equiv.) and urotropine (8 mmol, 1 equiv.) were dissolved in 40 ml of dry chloroform. The reaction mixture was stirred at 50 °C for 2 h. The precipitate was isolated by filtration and thoroughly washed with chloroform and ethanol. Then the urotropine salt was dissolved in 40 ml of absolute ethanol and 4 ml of concentrated hydrochloric acid. The mixture was heated for another 2 h. After the completion of reaction monitored by TLC, the solid was removed by filtration and washed with ethanol. The filtrate was evaporated under reduced pressure to yield the title compound (**H4-002**) as a white solid. ^1^H NMR (400 MHz, DMSO) δ 8.57 (s, 3H), 8.01 (d, *J* = 7.3 Hz, 2H), 7.72 (t, *J* = 7.4 Hz, 1H), 7.57 (t, *J* = 7.7 Hz, 2H), 4.60 (s, 2H); ^13 ^C NMR (101 MHz, DMSO) δ 193.29, 134.94, 134.13, 129.46, 128.67, 45.23; ESI-MS *m/z* = 136.03 [M + H]^+^.

##### 5.1.2.2. 2-Amino-1-(p-tolyl)ethanone hydrochloride (H5-002)

The title compound (**H5-002**) was synthesised from **H5-001** using the same method as the preparation of compound **H4-002**; ^1^H NMR (400 MHz, DMSO) δ 8.53 (s, 3H), 7.92 (d, *J* = 8.2 Hz, 2H), 7.39 (d, *J* = 8.1 Hz, 2H), 4.54 (s, 2H), 2.39 (s, 3H); ^13 ^C NMR (101 MHz, DMSO) δ 192.78, 145.58, 131.72, 129.99, 128.76, 45.05, 21.77. ESI-MS *m/z* = 150.11 [M + H]^+^.

##### 5.1.2.3. 2-Amino-1-(4-methoxyphenyl)ethanone hydrochloride (H6-002)

The title compound (**H6-002**) was synthesised from **H6-001** using the same method as the preparation of compound **H4-002**; ^1^H NMR (400 MHz, DMSO) δ 8.41 (s, 3H), 8.00 (d, *J* = 8.9 Hz, 2H), 7.10 (d, *J* = 8.9 Hz, 2H), 4.53 (d, *J* = 5.3 Hz, 2H), 3.86 (s, 3H); ^13 ^C NMR (101 MHz, DMSO) δ 191.56, 164.54, 131.12, 127.06, 114.71, 56.24, 44.83. ESI-MS *m/z* = 166.13 [M + H]^+^.

##### 5.1.2.4. 2-Amino-1-(3-methoxyphenyl)ethanone hydrochloride (H7-002)

The title compound (**H7-002**) was synthesised from **H7-001** using the same method as the preparation of compound **H4-002**; ^1^H NMR (400 MHz, DMSO) δ 8.51 (s, 3H), 7.64–7.59 (m, 1H), 7.53–7.47 (m, 3H), 4.59 (d, *J* = 5.2 Hz, 2H), 3.84 (s, 3H); ^13 ^C NMR (101 MHz, DMSO) δ 193.21, 159.95, 135.49, 130.70, 121.10, 120.99, 113.10, 56.01, 45.35. ESI-MS *m/z* = 166.13 [M + H]^+^.

##### 5.1.2.5. 2-Amino-1-(4-fluorophenyl)ethanone hydrochloride (H8-002)

The title compound (**H8-002**) was synthesised from **H8-001** using the same method as the preparation of compound **H4-002**; ^1^H NMR (400 MHz, DMSO) δ 8.48 (s, 3H), 8.19–8.07 (m, 2H), 7.44 (t, *J* = 8.8 Hz, 2H), 4.60 (s, 2H); ^13 ^C NMR (101 MHz, DMSO) δ 192.07, 166.15 (d, ^1^*J*_C-F_ = 235.7 Hz), 131.87 (d, ^3^*J*_C-F_ = 9.79 Hz, 2 C), 130.97 (d, ^4^*J*_C-F_ = 2.63 Hz), 116.61 (d, ^2^*J*_C-F_ = 22.11 Hz, 2 C), 45.22. ESI-MS *m/z* = 154.18 [M + H]^+^.

##### 5.1.2.6. 2-Amino-1-(3-fluorophenyl)ethanone hydrochloride (H9-002)

The title compound (**H9-002**) was synthesised from **H9-001** using the same method as the preparation of compound **H4-002**; ^1^H NMR (400 MHz, DMSO) δ 8.54 (s, 3H), 7.93–7.80 (m, 2H), 7.71–7.56 (m, 2H), 4.62 (d, *J* = 4.4 Hz, 2H); ^13 ^C NMR (101 MHz, DMSO) δ 192.60 (d, ^4^*J*_C-F_ = 2.22 Hz), 162.63 (d, ^1^*J*_C-F_ = 246.74 Hz), 136.26 (d, ^3^*J*_C-F_ = 6.67 Hz), 131.78 (d, ^3^*J*_C-F_ = 7.88 Hz), 124.95 (d, ^4^*J*_C-F_ = 2.83 Hz), 121.87 (d, ^2^*J*_C-F_ = 21.41 Hz), 115.30 (d, ^2^*J*_C-F_ = 22.62 Hz), 45.47. ESI-MS *m/z* = 154.12 [M + H]^+^.

##### 5.1.2.7. 2-Amino-1-(4-chlorophenyl)ethanone hydrochloride (H10-002)

The title compound (**H10-002**) was synthesised from **H10-001** using the same method as the preparation of compound **H4-002**; ^1^H NMR (400 MHz, DMSO) δ 8.56 (s, 3H), 8.04 (d, *J* = 8.6 Hz, 2H), 7.67 (d, *J* = 8.6 Hz, 2H), 4.59 (d, *J* = 3.9 Hz, 2H); ^13 ^C NMR (101 MHz, DMSO) δ 192.50, 139.81, 132.87, 130.63, 129.60, 45.27. ESI-MS *m/z* = 170.13 [M + H]^+^.

##### 5.1.2.8. 2-Amino-1-(3-chlorophenyl)ethanone hydrochloride (H11-002)

The title compound (**H11-002**) was synthesised from **H11-001** using the same method as the preparation of compound **H4-002**; ^1^H NMR (400 MHz, DMSO) δ 8.55 (s, 3H), 8.08–8.03 (m, 1H), 7.99 (d, *J* = 7.8 Hz, 1H), 7.84–7.78 (m, 1H), 7.63 (t, *J* = 7.9 Hz, 1H), 4.63 (s, 2H); ^13 ^C NMR (101 MHz, DMSO) δ 192.63, 135.97, 134.60, 134.35, 131.49, 128.40, 127.32, 45.42. ESI-MS *m/z* = 170.09 [M + H]^+^.

##### 5.1.2.9. 4-(2-Aminoacetyl)benzonitrile hydrochloride (H12-002)

The title compound (**H12-002**) was synthesised from **H12-001** using the same method as the preparation of compound **H4-002**; ^1^H NMR (400 MHz, DMSO) δ 8.63 (s, 3H), 8.18 (d, *J* = 8.6 Hz, 2H), 8.08 (d, *J* = 8.5 Hz, 2H), 4.66 (d, *J* = 4.5 Hz, 2H); ^13 ^C NMR (101 MHz, DMSO) δ 193.02, 137.29, 133.45, 129.34, 118.47, 116.62, 45.61. ESI-MS *m/z* = 161.13 [M + H]^+^.

##### 5.1.2.10. 2-Amino-1-(4-hydroxyphenyl)ethanone hydrochloride (H13-002)

The title compound (**H13-002**) was synthesised from **H13-001** using the same method as the preparation of compound **H4-002**; ^1^H NMR (400 MHz, DMSO) δ 10.92 (s, 1H), 8.43 (s, 3H), 7.88 (d, *J* = 8.8 Hz, 2H), 6.96 (d, *J* = 8.8 Hz, 2H), 4.45 (d, *J* = 5.3 Hz, 2H); ^13 ^C NMR (101 MHz, DMSO) δ 191.12, 163.88, 131.26, 125.56, 116.07, 44.55. ESI-MS *m/z* = 152.09 [M + H]^+^.

##### 5.1.2.11. 2-Amino-1-(2,4-difluorophenyl)ethanone hydrochloride (H14-002)

The title compound (**H14-002**) was synthesised from **H14-001** using the same method as the preparation of compound **H4-002**; ^1^H NMR (400 MHz, DMSO) δ 8.58 (s, 3H), 8.13–8.01 (m, 1H), 7.56–7.50 (m, 1H), 7.38–7.33 (m, 1H), 4.40 (s, 2H); ^13 ^C NMR (101 MHz, DMSO) δ 189.63, 189.59, 167.72, 165.05, 164.47, 161.90, 133.08, 133.01, 119.72, 119.60, 113.47, 113.22, 106.23, 105.97, 105.70, 48.05, 47.94. ESI-MS *m/z* = 172.10 [M + H]^+^.

##### 5.1.2.12. 2-Amino-1-(3,5-difluorophenyl)ethanone hydrochloride (H15-002)

The title compound (**H15-002**) was synthesised from **H15-001** using the same method as the preparation of compound **H4-002**; ^1^H NMR (400 MHz, DMSO) δ 8.63 (s, 3H), 7.80–7.67 (m, 3H), 4.61 (s, 2H); ^13 ^C NMR (101 MHz, DMSO) δ 191.71, 164.25, 164.13, 161.78, 161.66, 137.16, 112.25, 112.17, 112.06, 111.98, 110.26, 110.00, 45.58. ESI-MS *m/z* = 172.11 [M + H]^+^.

##### 5.1.2.13. 2-Amino-1-(3,4-dimethoxyphenyl)ethanone hydrochloride (H16-002)

The title compound (**H16-002**) was synthesised from **H16-001** using the same method as the preparation of compound **H4-002**; ^1^H NMR (400 MHz, DMSO) δ 8.48 (s, 3H), 7.69 (dd, *J* = 8.5, 1.9 Hz, 1H), 7.48 (d, *J* = 1.9 Hz, 1H), 7.13 (d, *J* = 8.5 Hz, 1H), 4.54 (d, *J* = 4.9 Hz, 2H), 3.85 (d, *J* = 10.2 Hz, 6H); ^13 ^C NMR (101 MHz, DMSO) δ 191.62, 154.48, 149.17, 126.98, 123.64, 111.57, 110.64, 56.39, 56.14, 44.82. ESI-MS *m/z* = 196.16 [M + H]^+^.

##### 5.1.2.14. 2-Amino-1-(3,4-dichlorophenyl)ethanone hydrochloride (H17-002)

The title compound (**H17-002**) was synthesised from **H17-001** using the same method as the preparation of compound **H4-002**; ^1^H NMR (400 MHz, DMSO) δ 8.63 (s, 3H), 8.26 (d, *J* = 1.9 Hz, 1H), 7.98 (dd, *J* = 8.4, 2.0 Hz, 1H), 7.88 (d, *J* = 8.4 Hz, 1H), 4.62 (d, *J* = 3.8 Hz, 2H); ^13 ^C NMR (101 MHz, DMSO) δ 191.92, 137.68, 134.32, 132.51, 131.81, 130.71, 128.67, 45.40. ESI-MS *m/z* = 204.04 [M + H]^+^.

##### 5.1.2.15. 2-Amino-1-(naphthalen-2-yl)ethanone hydrochloride (H18-002)

The title compound (**H18-002**) was synthesised from **H18-001** using the same method as the preparation of compound **H4-002**; ^1^H NMR (400 MHz, DMSO) δ 8.81 (s, 1H), 8.56 (s, 3H), 8.17 (d, *J* = 8.0 Hz, 1H), 8.11–7.99 (m, 3H), 7.75– 7.62 (m, 2H), 4.73 (d, *J* = 5.1 Hz, 2H); ^13 ^C NMR (101 MHz, DMSO) δ 193.28, 135.99, 132.49, 131.44, 131.32, 130.21, 129.77, 129.12, 128.28, 127.75, 123.52, 45.29. ESI-MS *m/z* = 136.03 [M + H]^+^. ESI-MS *m/z* = 186.08 [M + H]^+^.

##### 5.1.2.16. 2-Amino-1-(4-bromophenyl)ethanone hydrochloride (H19-002)

The title compound (**H19-002**) was synthesised from **H19-001** using the same method as the preparation of compound **H4-002**; ^1^H NMR (400 MHz, DMSO) δ 8.65 (s, 3H), 7.95 (d, *J* = 8.4 Hz, 2H), 7.79 (d, *J* = 8.4 Hz, 2H), 4.58 (s, 2H); ^13 ^C NMR (101 MHz, DMSO) δ 192.73, 133.19, 132.53, 130.67, 129.11, 45.22. ESI-MS *m/z* = 213.90, 215.92 [M + H]^+^.

##### 5.1.2.17. 2-Amino-1-(4-bromophenyl)propan-1-one hydrochloride (H30-002)

Sodium diformylamide (9.6 mmol, 1.2 eq) was suspended in 30 ml acetonitrile. 2-bromo-1-(4-bromophenyl)propan-1-one (**H30-001**) (8 mmol, 1 equiv.) was added drop-wise and with stirring. The mixture was then stirred at 75 °C for 4 h. The mixture was hot filtered and the solid was washed twice with acetonitrile. The combined organics were evaporated and the residue was dissolved in 20 ml water. 2 ml 10 N hydrochloric acid was added and the mixture was stirred at reflux for 1 h. After completed, the solvent was removed under reduced pressure. The residue was washed twice with ethyl acetate and the solid was collected by filtration and dried to give **H30-002**; ^1^H NMR (400 MHz, DMSO) δ 8.66 (s, 3H), 8.01 (d, *J* = 8.5 Hz, 2H), 7.81 (d, *J* = 8.5 Hz, 2H), 5.18–5.04 (m, 1H), 1.42 (d, *J* = 7.1 Hz, 3H); ^13 ^C NMR (101 MHz, DMSO) δ 196.41, 132.73, 132.37, 131.24, 129.21, 51.28, 17.39. ESI-MS *m/z* = 227.92, 229.91 [M + H]^+^.

#### 5.1.3. Synthesis procedure for X-003

##### 5.1.3.1. 2-Amino-N-(2-oxo-2-phenylethyl)benzamide (H4-003)

To a solution of 2-amino-1-phenylethanone hydrochloride (**H4-002**) (6 mmol, 1 equiv.) in 20 ml water, sodium carbonate (3.72 mmol, 0.62 eq) were added. After being stirred at room temperature for 10 min, isatoic anhydride (6 mmol, 1 equiv.) was dissolved in 10 ml water and added dropwise to reaction mixture. The solution was stirred at room temperature for 3 h and then monitored by TLC. After completion was indicated, the resultant solution was extracted with ethyl acetate and then the organic layer dried over anhydrous Na_2_SO_4_. After filtration and concentration, the crude product was obtained and purified with column chromatography to provide **H4-003**; ^1^H NMR (400 MHz, DMSO) δ 8.60 (t, *J* = 5.4 Hz, 1H), 8.05 (d, *J* = 7.8 Hz, 2H), 7.69 (t, *J* = 7.4 Hz, 1H), 7.63–7.51 (m, 3H), 7.17 (t, *J* = 7.6 Hz, 1H), 6.72 (d, *J* = 8.2 Hz, 1H), 6.55 (t, *J* = 7.5 Hz, 1H), 6.45 (s, 2H), 4.72 (d, *J* = 5.6 Hz, 2H); ^13 ^C NMR (101 MHz, DMSO) δ 196.12, 169.59, 150.27, 135.61, 134.00, 132.41, 129.31, 128.64, 128.32, 116.90, 115.06, 114.43, 46.61. ESI-MS *m/z* = 255.24 [M + H]^+^, 277.70 [M + Na]^+^.

##### 5.1.3.2. 2-Amino-N-(2-oxo-2-(p-tolyl)ethyl)benzamide (H5-003)

Compound **H5-003** was prepared by following a similar procedure to that for **H4-003**; ^1^H NMR (400 MHz, DMSO) δ 8.57 (t, *J* = 5.5 Hz, 1H), 7.95 (d, *J* = 8.1 Hz, 2H), 7.60 (d, *J* = 7.1 Hz, 1H), 7.37 (d, *J* = 8.0 Hz, 2H), 7.22–7.12 (m, 1H), 6.72 (d, *J* = 8.1 Hz, 1H), 6.55 (t, *J* = 7.5 Hz, 1H), 6.45 (s, 2H), 4.69 (d, *J* = 5.6 Hz, 2H), 2.40 (s, 3H); ^13 ^C NMR (101 MHz, DMSO) δ 195.56, 169.56, 150.26, 144.40, 133.10, 132.39, 129.84, 128.63, 128.42, 116.90, 115.05, 114.47, 46.47, 21.69. ESI-MS *m/z* = 269.13 [M + H]^+^, 291.28 [M + Na]^+^.

##### 5.1.3.3. 2-Amino-N-(2-(4-methoxyphenyl)-2-oxoethyl)benzamide (H6-003)

Compound **H6-003** was prepared by following a similar procedure to that for **H4-003**; ^1^H NMR (400 MHz, DMSO) δ 8.54 (t, *J* = 5.6 Hz, 1H), 8.03 (d, *J* = 8.9 Hz, 2H), 7.59 (d, *J* = 7.1 Hz, 1H), 7.21–7.14 (m, 1H), 7.08 (d, *J* = 8.9 Hz, 2H), 6.72 (d, *J* = 8.2 Hz, 1H), 6.55 (t, *J* = 7.5 Hz, 1H), 6.45 (s, 2H), 4.67 (d, *J* = 5.6 Hz, 2H), 3.86 (s, 3H); ^13 ^C NMR (101 MHz, DMSO) δ 194.36, 169.55, 163.80, 150.22, 132.36, 130.64, 128.63, 128.48, 116.89, 115.06, 114.58, 114.49, 56.03, 46.21. ESI-MS *m/z* = 285.16 [M + H]^+^, 307.15 [M + Na]^+^.

##### 5.1.3.4. 2-Amino-N-(2-(3-methoxyphenyl)-2-oxoethyl)benzamide (H7-003)

Compound **H7-003** was prepared by following a similar procedure to that for **H4-003**; ^1^H NMR (400 MHz, DMSO) δ 8.61 (t, *J* = 5.5 Hz, 1H), 7.68–7,57 (m, 2H), 7.54–7.45 (m, 2H), 7.28–7.22 (m, 1H), 7.20–7.14 (m, 1H), 6.72 (d, *J* = 8.1 Hz, 1H), 6.55 (t, *J* = 7.3 Hz, 1H), 6.45 (s, 2H), 4.71 (d, *J* = 5.6 Hz, 2H), 3.84 (s, 3H); ^13 ^C NMR (101 MHz, DMSO) δ 195.94, 169.58, 159.91, 150.27, 136.97, 132.41, 130.49, 128.64, 120.76, 120.02, 116.90, 115.06, 114.42, 112.81, 55.84, 46.76. ESI-MS *m/z* = 285.30 [M + H]^+^, 307.55 [M + Na]^+^.

##### 5.1.3.5. 2-Amino-N-(2-(4-fluorophenyl)-2-oxoethyl)benzamide (H8-003)

Compound **H8-003** was prepared by following a similar procedure to that for **H4-003**; ^1^H NMR (400 MHz, DMSO) δ 8.61 (t, *J* = 5.4 Hz, 1H), 8.20–8.08 (m, 2H), 7.59 (d, *J* = 7.9 Hz, 1H), 7.40 (t, *J* = 8.8 Hz, 2H), 7.21–7.13 (m, 1H), 6.71 (d, *J* = 8.3 Hz, 1H), 6.55 (t, *J* = 7.5 Hz, 1H), 6.45 (s, 2H), 4.71 (d, *J* = 5.6 Hz, 2H); ^13 ^C NMR (101 MHz, DMSO) δ 194.80, 169.59, 168.43, 166.77, 150.27, 146.75, 133.24, 133.21, 132.42, 132.34, 132.10, 131.42, 131.33, 131.00, 130.91, 130.34, 128.63, 127.06, 126.97, 126.18, 116.90, 116.45, 116.41, 116.24, 116.20, 115.06, 114.37, 46.57. ESI-MS *m/z* = 273.38 [M + H]^+^, 295.13 [M + Na]^+^.

##### 5.1.3.6. 2-Amino-N-(2-(3-fluorophenyl)-2-oxoethyl)benzamide (H9-003)

Compound **H9-003** was prepared by following a similar procedure to that for **H4-003**; ^1^H NMR (400 MHz, DMSO) δ 8.64 (t, *J* = 5.4 Hz, 1H), 7.93–7.80 (m, 2H), 7.67–7.52 (m, 3H), 7.22–7.13 (m, 1H), 6.71 (d, *J* = 8.2 Hz, 1H), 6.58–6.52 (m, 1H), 6.45 (s, 2H), 4.71 (d, *J* = 5.6 Hz, 2H); ^13 ^C NMR (101 MHz, DMSO) δ 195.28, 169.61, 168.33, 166.85, 163.89, 161.44, 150.30, 146.49, 139.18, 137.79, 137.73, 132.45, 132.15, 131.58, 131.51, 131.42, 131.34, 130.37, 128.64, 127.14, 127.04, 126.48, 124.59, 124.57, 121.00, 120.79, 118.79, 118.58, 116.92, 115.05, 114.80, 114.28, 46.85. ESI-MS *m/z* = 273.11 [M + H]^+^, 295.15 [M + Na]^+^.

##### 5.1.3.7. 2-Amino-N-(2-(4-chlorophenyl)-2-oxoethyl)benzamide (H10-003)

Compound **H10-003** was prepared by following a similar procedure to that for **H4-003**; ^1^H NMR (400 MHz, DMSO) δ 8.62 (t, *J* = 5.4 Hz, 1H), 8.06 (d, *J* = 8.5 Hz, 2H), 7.61 (dd, *J* = 22.5, 7.8 Hz, 3H), 7.22–7.12 (m, 1H), 6.71 (d, *J* = 8.0 Hz, 1H), 6.55 (t, *J* = 7.4 Hz, 1H), 6.44 (s, 2H), 4.70 (d, *J* = 5.5 Hz, 2H); ^13 ^C NMR (101 MHz, DMSO) δ 195.32, 169.59, 150.28, 138.86, 134.29, 132.45, 130.29, 129.43, 128.63, 116.91, 115.05, 114.29, 46.66. ESI-MS *m/z* = 289.15 [M + H]^+^.

##### 5.1.3.8. 2-Amino-N-(2-(3-chlorophenyl)-2-oxoethyl)benzamide (H11-003)

Compound **H11-003** was prepared by following a similar procedure to that for **H4-003**; ^1^H NMR (400 MHz, DMSO) δ 8.65 (t, *J* = 5.4 Hz, 1H), 8.05 (s, 1H), 8.01 (d, *J* = 7.8 Hz, 1H), 7.79–7.71 (m, 1H), 7.66–7.54 (m, 2H), 7.23–7.11 (m, 1H), 6.71 (d, *J* = 8.0 Hz, 1H), 6.55 (t, *J* = 7.4 Hz, 1H), 6.45 (s, 2H), 4.71 (d, *J* = 5.5 Hz, 2H); ^13 ^C NMR (101 MHz, DMSO) δ 195.38, 169.60, 150.31, 137.44, 134.23, 133.68, 132.47, 131.31, 128.64, 128.03, 127.04, 116.92, 115.05, 114.23, 46.82. ESI-MS *m/z* = 289.04 [M + H]^+^, 311.29 [M + Na]^+^.

##### 5.1.3.9. 2-Amino-N-(2-(4-cyanophenyl)-2-oxoethyl)benzamide (H12-003)

Compound **H12-003** was prepared by following a similar procedure to that for **H4-003**. The crude product was used for the follow-up reaction directly without further purification. ESI-MS *m/z* = 280.26 [M + H]^+^, 302.11 [M + Na]^+^.

##### 5.1.3.10. 2-Amino-N-(2-(4-hydroxyphenyl)-2-oxoethyl)benzamide (H13-003)

Compound **H13-003** was prepared by following a similar procedure to that for **H4-003**. ^1^H NMR (400 MHz, DMSO) δ 10.45 (s, 1H), 8.49 (t, *J* = 5.6 Hz, 1H), 7.93 (d, *J* = 8.7 Hz, 2H), 7.59 (d, *J* = 7.9 Hz, 1H), 7.21–7.12 (m, 1H), 6.89 (d, *J* = 8.7 Hz, 2H), 6.71 (d, *J* = 8.2 Hz, 1H), 6.55 (t, *J* = 7.5 Hz, 1H), 6.43 (s, 2H), 4.63 (d, *J* = 5.6 Hz, 2H); ^13 ^C NMR (101 MHz, DMSO) δ 194.00, 169.53, 162.76, 150.19, 132.33, 130.85, 128.62, 127.14, 116.87, 115.82, 115.06, 114.66, 46.00. ESI-MS *m/z* = 271.11 [M + H]^+^, 293.58 [M + Na]^+^.

##### 5.1.3.11. 2-Amino-N-(2-(2,4-difluorophenyl)-2-oxoethyl)benzamide (H14-003)

Compound **H14-003** was prepared by following a similar procedure to that for **H4-003**. ^1^H NMR (400 MHz, DMSO) δ 8.66–8.57 (m, 1H), 7.98 (dd, *J* = 15.3, 8.5 Hz, 1H), 7.57 (d, *J* = 7.8 Hz, 1H), 7.48–7.36 (m, 1H), 7.24 (t, *J* = 8.4 Hz, 1H), 7.15 (t, *J* = 7.6 Hz, 1H), 6.71 (d, *J* = 8.2 Hz, 1H), 6.54 (t, *J* = 7.5 Hz, 1H), 6.42 (s, 2H), 4.54 (dd, *J* = 5.2, 2.8 Hz, 2H); ^13 ^C NMR (101 MHz, DMSO) δ 193.20, 193.15, 169.62, 168.35, 167.00, 166.88, 165.67, 164.75, 164.71, 164.47, 164.35, 163.87, 163.74, 163.04, 162.77, 162.64, 161.32, 161.19, 160.25, 160.12, 150.27, 146.01, 132.99, 132.95, 132.88, 132.84, 132.39, 132.11, 130.35, 128.62, 127.17, 127.06, 126.62, 121.16, 121.13, 121.02, 120.99, 116.90, 115.02, 114.22, 113.09, 113.06, 112.88, 112.85, 112.71, 112.67, 105.87, 105.60, 105.34, 105.30, 79.72, 79.39, 79.06, 49.74, 49.65. ESI-MS *m/z* = 291.20 [M + H]^+^, 313.16 [M + Na]^+^.

##### 5.1.3.12. 2-Amino-N-(2-(3,5-difluorophenyl)-2-oxoethyl)benzamide (H15-003)

Compound **H15-003** was prepared by following a similar procedure to that for **H4-003**. ^1^H NMR (400 MHz, DMSO) δ 8.67 (t, *J* = 5.4 Hz, 1H), 7.80–7.71 (m, 2H), 7.68–7.56 (m, 2H), 7.21–7.13 (m, 1H), 6.71 (d, *J* = 8.1 Hz, 1H), 6.55 (t, *J* = 7.5 Hz, 1H), 6.45 (s, 2H), 4.70 (d, *J* = 5.5 Hz, 2H); ^13 ^C NMR (101 MHz, DMSO) δ 194.41, 169.62, 168.21, 165.93, 164.37, 164.24, 161.91, 161.79, 150.32, 146.13, 140.31, 132.50, 132.20, 130.40, 128.63, 127.18, 127.08, 126.79, 116.92, 115.04, 114.11, 111.82, 111.67, 111.41, 107.41, 107.15, 106.89, 46.97. ESI-MS *m/z* = 291.35 [M + H]^+^, 313.16 [M + Na]^+^.

##### 5.1.3.13. 2-Amino-N-(2-(3,4-dimethoxyphenyl)-2-oxoethyl)benzamide (H16-003)

Compound **H16-003** was prepared by following a similar procedure to that for **H4-003**. The crude product was used for the follow-up reaction directly without further purification. ESI-MS *m/z* = 315.29 [M + H]^+^, 337.25 [M + Na]^+^.

##### 5.1.3.14. 2-Amino-N-(2-(3,4-dichlorophenyl)-2-oxoethyl)benzamide (H17-003)

Compound **H17-003** was prepared by following a similar procedure to that for **H4-003**. The crude product was used for the follow-up reaction directly without further purification. ESI-MS *m/z* = 323.06 [M + H]^+^.

##### 5.1.3.15. 2-Amino-N-(2-(naphthalen-2-yl)-2-oxoethyl)benzamide (H18-003)

Compound **H18-003** was prepared by following a similar procedure to that for **H4-003**. The crude product was used for the follow-up reaction directly without further purification.

##### 5.1.3.16. 2-Amino-N-(2-(4-bromophenyl)-2-oxoethyl)benzamide (H19-003)

Compound **H19-003** was prepared by following a similar procedure to that for **H4-003**. ^1^H NMR (400 MHz, DMSO) δ 8.68–8.56 (m, 1H), 8.01–7.95 (m, 2H), 7.84–7.75 (m, 2H), 7.62–7.55 (m, 1H), 7.25–7.11 (m, 1H), 6.77–6.67 (m, 1H), 6.61–6.51 (m, 1H), 6.44 (s, 2H), 4.68 (d, *J* = 5.6 Hz, 2H). ESI-MS *m/z* = 333.18, 335.11 [M + H]^+^, 354.99 [M + Na]^+^.

##### 5.1.3.17. 2-Amino-N-(1-(4-bromophenyl)-1-oxopropan-2-yl)benzamide (H30-003)

Compound **H30-003** was prepared by following a similar procedure to that for **H4-003**. ^1^H NMR (400 MHz, DMSO) δ 8.74–8.58 (m, 1H), 7.94 (d, *J* = 8.2 Hz, 2H), 7.75 (d, *J* = 8.2 Hz, 2H), 7.59–7.47 (m, 1H), 7.14 (t, *J* = 7.0 Hz, 1H), 6.72–6.62 (m, 1H), 6.51 (t, *J* = 7.0 Hz, 1H), 6.37 (s, 2H), 5.44–5.27 (m, 1H), 1.37 (d, *J* = 6.8 Hz, 3H); ^13 ^C NMR (101 MHz, DMSO) δ 199.26, 168.97, 150.25, 134.67, 132.46, 132.24, 130.67, 128.92, 127.63, 116.77, 114.92, 114.12, 50.52, 16.83. ESI-MS *m/z* = 347.08, 349.38 [M + H]^+^, 370.26 [M + Na]^+^.

#### 5.1.4. Synthesis procedure for compounds H4–H19

##### 5.1.4.1. 2-Phenyl-3,4-dihydro-5H-benzo[e][1,4]diazepin-5-one (H4)

2-Amino-*N*-(2-oxo-2-phenylethyl)benzamide (**H4-003**) (2 mmol) was dissolved in xylene (10 ml), and the reaction was heated and refluxed in nitrogen atmosphere for 3 h. The solvent xylene was evaporated to dryness, and the crude product was purified and crystallised from ethyl acetate to afford compound **H4**; ^1^H NMR (400 MHz, DMSO) δ 8.61 (t, *J* = 5.8 Hz, 1H), 8.09 (dd, *J* = 7.7, 1.6 Hz, 2H), 7.88 (d, *J* = 7.4 Hz, 1H), 7.65–7.52 (m, 4H), 7.42–7.31 (m, 2H), 3.96 (s, 2H); ^13 ^C NMR (101 MHz, DMSO) δ 168.46, 167.87, 146.86, 136.67, 132.09, 131.86, 130.34, 129.29, 128.37, 127.09, 126.99, 126.15, 38.99. ESI-MS *m/z* = 237.02 [M + H]^+^, 259.13 [M + Na]^+^.

##### 5.1.4.2. 2-(p-Tolyl)-3,4-dihydro-5H-benzo[e][1,4]diazepin-5-one (H5)

Starting from **H5-003** (2 mmol), and following the procedure similar to that of preparation of **H4** to give **H5**; ^1^H NMR (400 MHz, DMSO) δ 8.59 (t, *J* = 5.9 Hz, 1H), 7.99 (d, *J* = 8.2 Hz, 2H), 7.90–7.83 (m, 1H), 7.64–7.57 (m, 1H), 7.40–7.30 (m, 4H), 3.93 (s, 2H), 2.40 (s, 3H); ^13 ^C NMR (101 MHz, DMSO) δ 168.51, 167.60, 146.99, 141.95, 133.95, 132.05, 130.32, 129.88, 128.40, 127.09, 126.95, 125.95, 38.88, 21.51. ESI-MS *m/z* = 251.06 [M + H]^+^, 273.31 [M + Na]^+^.

##### 5.1.4.3. 2-(4-Methoxyphenyl)-3,4-dihydro-5H-benzo[e][1,4]diazepin-5-one (H6)

Starting from **H6-003** (2 mmol), and following the procedure similar to that of preparation of **H4** to give **H6**; ^1^H NMR (400 MHz, DMSO) δ 8.65–8.44 (m, 1H), 8.06 (d, J = 7.5 Hz, 2H), 7.86 (d, J = 7.0 Hz, 1H), 7.66–7.50 (m, 1H), 7.33 (d, J = 7.0 Hz, 2H), 7.10 (d, J = 7.5 Hz, 2H), 3.93 (s, 2H), 3.86 (s, 3H); 13 C NMR (101 MHz, DMSO) δ 168.60, 166.93, 162.39, 147.17, 132.01, 130.28, 129.02, 127.02, 126.94, 125.68, 114.66, 55.95, 38.73. ESI-MS *m/z* = 267.33 [M + H]^+^, 289.01 [M + Na]^+^.

##### 5.1.4.4. 2-(3-Methoxyphenyl)-3,4-dihydro-5H-benzo[e][1,4]diazepin-5-one (H7)

Starting from **H7-003** (2 mmol), and following the procedure similar to that of preparation of **H4** to give **H7**; ^1^H NMR (400 MHz, DMSO) δ 8.60 (t, *J* = 5.9 Hz, 1H), 7.87 (dd, *J* = 7.7, 1.0 Hz, 1H), 7.70–7.56 (m, 3H), 7.47 (t, *J* = 7.9 Hz, 1H), 7.40–7.33 (m, 2H), 7.16 (dd, *J* = 8.1, 2.0 Hz, 1H), 3.94 (s, 2H), 3.85 (s, 3H); ^13 ^C NMR (101 MHz, DMSO) δ 168.40, 167.71, 160.02, 146.77, 138.14, 132.07, 130.38, 130.32, 127.10, 126.99, 126.19, 120.83, 117.72, 113.29, 55.81, 39.17. ESI-MS *m/z* = 267.15 [M + H]^+^, 289.33 [M + Na]^+^.

##### 5.1.4.5. 2-(4-Fluorophenyl)-3,4-dihydro-5H-benzo[e][1,4]diazepin-5-one (H8)

Starting from **H8-003** (2 mmol), and following the procedure similar to that of preparation of **H4** to give **H8**; ^1^H NMR (400 MHz, DMSO) δ 8.58 (t, *J* = 5.9 Hz, 1H), 8.15 (dd, *J* = 8.8, 5.6 Hz, 2H), 7.87 (dd, *J* = 8.1, 1.4 Hz, 1H), 7.66–7.59 (m, 1H), 7.44–7.33 (m, 4H), 3.96 (d, *J* = 3.3 Hz, 2H); ^13 ^C NMR (101 MHz, DMSO) δ 168.43, 166.76, 165.79, 163.30, 146.75, 133.24, 133.21, 132.10, 131.00, 130.91, 130.34, 127.06, 126.98, 126.18, 116.41, 116.20, 38.95. ESI-MS *m/z* = 255.24 [M + H]^+^, 277.13 [M + Na]^+^.

##### 5.1.4.6. 2-(3-Fluorophenyl)-3,4-dihydro-5H-benzo[e][1,4]diazepin-5-one (H9)

Starting from **H9-003** (2 mmol), and following the procedure similar to that of preparation of **H4** to give **H9**; ^1^H NMR (400 MHz, DMSO) δ 8.63–8.49 (m, 1H), 7.97–7.82 (m, 3H), 7.69–7.55 (m, 2H), 7.50–7.32 (m, 3H), 3.96 (s, 2H); ^13 ^C NMR (101 MHz, DMSO) δ 168.33, 166.85, 166.82, 164.08, 161.66, 146.49, 139.18, 139.11, 132.14, 131.41, 131.33, 130.36, 127.14, 127.03, 126.47, 124.56, 124.53, 118.78, 118.57, 115.00, 114.78, 49.07, 39.02. ESI-MS *m/z* = 255.09 [M + H]^+^, 277.25 [M + Na]^+^.

##### 5.1.4.7. 2-(4-Chlorophenyl)-3,4-dihydro-5H-benzo[e][1,4]diazepin-5-one (H10)

Starting from **H10-003** (2 mmol), and following the procedure similar to that of preparation of **H4** to give **H10**; ^1^H NMR (400 MHz, DMSO) δ 8.59 (t, *J* = 5.7 Hz, 1H), 8.10 (d, *J* = 8.6 Hz, 2H), 7.87 (dd, *J* = 8.0, 1.1 Hz, 1H), 7.67–7.59 (m, 3H), 7.40–7.33 (m, 2H), 3.94 (s, 2H); ^13 ^C NMR (101 MHz, DMSO) δ 168.37, 166.88, 146.63, 136.73, 135.46, 132.13, 130.37, 130.18, 129.37, 127.11, 127.00, 126.35, 38.85. ESI-MS *m/z* = 271.26 [M + H]^+^, 293.54 [M + Na]^+^.

##### 5.1.4.8. 2-(3-Chlorophenyl)-3,4-dihydro-5H-benzo[e][1,4]diazepin-5-one (H11)

Starting from **H11-003** (2 mmol), and following the procedure similar to that of preparation of **H4** to give **H11**; ^1^H NMR (400 MHz, DMSO) δ 8.56 (t, *J* = 5.8 Hz, 1H), 8.13 (s, 1H), 8.04 (d, *J* = 7.7 Hz, 1H), 7.88 (dd, *J* = 7.7, 1.0 Hz, 1H), 7.70–7.55 (m, 3H), 7.44–7.34 (m, 2H), 3.95 (d, *J* = 4.1 Hz, 2H); ^13 ^C NMR (101 MHz, DMSO) δ 168.29, 166.76, 146.46, 138.77, 134.23, 132.15, 131.55, 131.21, 130.36, 128.00, 127.15, 127.02, 126.52, 38.98. ESI-MS *m/z* = 271.29 [M + H]^+^, 293.00 [M + Na]^+^.

##### 5.1.4.9. 4-(5-Oxo-4,5-dihydro-3H-benzo[e][1,4]diazepin-2-yl)benzonitrile (H12)

Starting from **H12-003** (2 mmol), and following the procedure similar to that of preparation of **H4** to give **H12**; ^1^H NMR (400 MHz, DMSO) δ 8.60 (t, *J* = 5.9 Hz, 1H), 8.24 (d, *J* = 8.5 Hz, 2H), 8.05 (d, *J* = 8.5 Hz, 2H), 7.89 (dd, *J* = 8.1, 1.4 Hz, 1H), 7.69–7.61 (m, 1H), 7.43–7.36 (m, 2H), 3.98 (d, *J* = 5.0 Hz, 2H); ^13 ^C NMR (101 MHz, DMSO) δ 168.25, 166.90, 146.32, 140.72, 133.24, 132.22, 130.43, 129.04, 127.14, 126.82, 118.94, 113.87, 38.91. ESI-MS *m/z* = 262.26 [M + H]^+^, 284.15 [M + Na]^+^.

##### 5.1.4.10. 2-(4-Hydroxyphenyl)-3,4-dihydro-5H-benzo[e][1,4]diazepin-5-one (H13)

Starting from **H13-003** (2 mmol), and following the procedure similar to that of preparation of **H4** to give **H13**; ^1^H NMR (400 MHz, DMSO) δ 10.21 (s, 1H), 8.53 (t, *J* = 5.9 Hz, 1H), 7.96 (d, *J* = 8.7 Hz, 2H), 7.84 (dd, *J* = 8.0, 1.4 Hz, 1H), 7.62–7.54 (m, 1H), 7.34–7.26 (m, 2H), 6.91 (d, *J* = 8.7 Hz, 2H), 3.90 (s, 2H); ^13 ^C NMR (101 MHz, DMSO) δ 170.80, 146.90, 143.78, 132.27, 131.56, 129.58, 128.77, 127.55, 127.27, 120.52, 119.19, 117.06, 45.97. ESI-MS *m/z* = 253.08 [M + H]^+^.

##### 5.1.4.11. 2-(2,4-Difluorophenyl)-3,4-dihydro-5H-benzo[e][1,4]diazepin-5-one (H14)

Starting from **H14-003** (2 mmol), and following the procedure similar to that of preparation of **H4** to give **H14**; ^1^H NMR (400 MHz, DMSO) δ 8.69 (t, *J* = 5.6 Hz, 1H), 8.00–7.85 (m, 2H), 7.68–7.60 (m, 1H), 7.53–7.44 (m, 1H), 7.43–7.35 (m, 2H), 7.31–7.24 (m, 1H), 3.85 (d, *J* = 5.4 Hz, 2H); ^13 ^C NMR (101 MHz, DMSO) δ 168.30, 165.53, 164.84, 163.15, 162.74, 162.61, 160.22, 160.09, 146.01, 133.04, 133.00, 132.94, 132.90, 132.21, 130.39, 127.20, 127.07, 126.72, 122.89, 122.77, 113.02, 112.80, 105.68, 105.42, 105.16, 41.66, 41.59. ESI-MS *m/z* = 273.13 [M + H]^+^, 295.06 [M + Na]^+^.

##### 5.1.4.12. 2-(3,5-Difluorophenyl)-3,4-dihydro-5H-benzo[e][1,4]diazepin-5-one (H15)

Starting from **H15-003** (2 mmol), and following the procedure similar to that of preparation of **H4** to give **H15**; ^1^H NMR (400 MHz, DMSO) δ 8.49 (t, *J* = 5.6 Hz, 1H), 7.91–7.84 (m, 1H), 7.83–7.73 (m, 2H), 7.68–7.59 (m, 1H), 7.56–7.47 (m, 1H), 7.43–7.34 (m, 2H), 3.95 (d, *J* = 5.1 Hz, 2H); ^13 ^C NMR (101 MHz, DMSO) δ 168.20, 165.94, 164.37, 164.24, 161.92, 161.79, 146.13, 140.31, 132.20, 130.40, 127.19, 127.08, 126.79, 111.68, 111.42, 107.42, 107.16, 38.96. ESI-MS *m/z* = 273.35 [M + H]^+^, 295.31 [M + Na]^+^.

##### 5.1.4.13. 2-(3,4-Dimethoxyphenyl)-3,4-dihydro-5H-benzo[e][1,4]diazepin-5-one (H16)

Starting from **H16-003** (2 mmol), and following the procedure similar to that of preparation of **H4** to give **H16**; ^1^H NMR (400 MHz, DMSO) δ 8.57 (t, *J* = 5.9 Hz, 1H), 7.85 (dd, *J* = 7.8, 1.2 Hz, 1H), 7.72–7.63 (m, 2H), 7.63–7.56 (m, 1H), 7.38–7.28 (m, 2H), 7.11 (d, *J* = 8.4 Hz, 1H), 3.94 (s, 2H), 3.86 (s, 6H); ^13 ^C NMR (101 MHz, DMSO) δ 168.59, 166.98, 152.32, 149.33, 147.15, 131.99, 130.29, 129.08, 127.02, 126.95, 125.68, 122.42, 111.60, 110.75, 56.16, 56.01, 38.73. ESI-MS *m/z* = 297.25 [M + H]^+^.

##### 5.1.4.14. 2-(3,4-Dichlorophenyl)-3,4-dihydro-5H-benzo[e][1,4]diazepin-5-one (H17)

Starting from **H17-003** (2 mmol), and following the procedure similar to that of preparation of **H4** to give **H17**; ^1^H NMR (400 MHz, DMSO) δ 8.53 (t, *J* = 5.9 Hz, 1H), 8.30 (d, *J* = 1.9 Hz, 1H), 8.05 (dd, *J* = 8.5, 2.0 Hz, 1H), 7.90–7.80 (m, 2H), 7.64 (td, *J* = 7.8, 1.4 Hz, 1H), 7.43–7.34 (m, 2H), 3.95 (d, *J* = 4.5 Hz, 2H); ^13 ^C NMR (101 MHz, DMSO) δ 168.25, 165.96, 146.31, 137.18, 134.53, 132.29, 132.18, 131.55, 130.39, 130.11, 128.39, 127.16, 127.05, 126.65, 38.81. ESI-MS *m/z* = 305.03 [M + H]^+^.

##### 5.1.4.15. 2-(Naphthalen-2-yl)-3,4-dihydro-5H-benzo[e][1,4]diazepin-5-one (H18)

Starting from **H18-003** (2 mmol), and following the procedure similar to that of preparation of **H4** to give **H18**; ^1^H NMR (400 MHz, DMSO) δ 8.71–8.62 (m, 2H), 8.27 (dd, *J* = 8.7, 1.5 Hz, 1H), 8.10–7.98 (m, 3H), 7.90 (dd, *J* = 7.8, 1.2 Hz, 1H), 7.69–7.59 (m, 3H), 7.45–7.34 (m, 2H), 4.12 (s, 2H); ^13 ^C NMR (101 MHz, DMSO) δ 168.53, 167.68, 146.97, 134.66, 133.91, 133.01, 132.12, 130.37, 129.43, 128.92, 128.44, 128.18, 127.41, 127.08, 126.20, 124.77, 38.84. ESI-MS *m/z* = 287.75 [M + H]^+^.

##### 5.1.4.16. 2-(4-Bromophenyl)-3,4-dihydro-5H-benzo[e][1,4]diazepin-5-one (H19)

Starting from **H19-003** (2 mmol), and following the procedure similar to that of preparation of **H4** to give **H19**; ^1^H NMR (400 MHz, DMSO) δ 8.56 (t, *J* = 5.9 Hz, 1H), 8.02 (d, *J* = 8.6 Hz, 2H), 7.87 (dd, *J* = 8.0, 1.3 Hz, 1H), 7.76 (d, *J* = 8.6 Hz, 2H), 7.65–7.58 (m, 1H), 7.40–7.32 (m, 2H), 3.93 (d, *J* = 5.2 Hz, 2H); ^13 ^C NMR (101 MHz, DMSO) δ 168.37, 167.02, 146.63, 135.83, 132.30, 132.13, 130.37, 130.35, 127.11, 126.99, 126.36, 125.72, 38.82. ESI-MS *m/z* = 315.12, 317.01 [M + H]^+^.

#### 5.1.5. Synthesis procedure for compound H20–H27

##### 5.1.5.1. 2-(4-Methoxyphenyl)-1,2,3,4-tetrahydro-5H-benzo[e][1,4]diazepin-5-one (H20)

The solution of 2-(4-methoxyphenyl)-3*H*-benzo[e][1,4]diazepin-5(4*H*)-one (**H6**) (1 mmol, 1eq) in methanol was placed in an ice bath, then the sodium borohydride (4 mmol, 4 equiv.) was added to the reaction bottle in batches maintaining the temperature below 10 °C. The reaction was monitored by TLC. After completed, the solvent methanol was removed under reduced pressure. The residue was extracted twice with ethyl acetate and water, the combined organic layer was washed with brine, dried over anhydrous Na_2_SO_4_. After filtration and concentration, the crude product was obtained and purified with column chromatography to afford the compound **H20**; ^1^H NMR (400 MHz, DMSO) δ 7.89 (t, *J* = 5.9 Hz, 1H), 7.62 (dd, *J* = 7.9, 1.4 Hz, 1H), 7.28–7.17 (m, 3H), 6.92 (d, *J* = 8.7 Hz, 2H), 6.86 (d, *J* = 8.0 Hz, 1H), 6.67 (t, *J* = 7.4 Hz, 1H), 6.48 (d, *J* = 3.4 Hz, 1H), 4.73–4.65 (m, 1H), 3.74 (s, 3H), 3.40–3.34 (m, 2H); ^13 ^C NMR (101 MHz, DMSO) δ 170.78, 158.90, 146.88, 135.70, 132.32, 132.24, 128.32, 119.36, 119.20, 116.99, 114.16, 62.25, 55.56, 46.22. ESI-MS *m/z* = 291.06 [M + Na]^+^.

##### 5.1.5.2. 2-(3-Methoxyphenyl)-1,2,3,4-tetrahydro-5H-benzo[e][1,4]diazepin-5-one (H21)

Starting from **H7** (1 mmol), and following the procedure similar to that of preparation of **H20** to give **H21**; ^1^H NMR (400 MHz, DMSO) δ 7.90 (t, *J* = 5.8 Hz, 1H), 7.61 (dd, *J* = 7.9, 1.4 Hz, 1H), 7.31–7.17 (m, 2H), 6.93–6.81 (m, 4H), 6.68 (t, *J* = 7.1 Hz, 1H), 6.54 (d, *J* = 3.7 Hz, 1H), 4.78–4.69 (m, 1H), 3.74 (s, 3H), 3.44–3.37 (m, 2H); ^13 ^C NMR (101 MHz, DMSO) δ 170.76, 159.74, 146.84, 145.40, 132.36, 132.25, 129.84, 119.48, 119.44, 119.17, 117.10, 113.07, 112.75, 62.63, 55.44, 45.90. ESI-MS *m/z* = 269.19 [M + H]^+^, 291.05 [M + Na]^+^.

##### 5.1.5.3. 2-(4-Fluorophenyl)-1,2,3,4-tetrahydro-5H-benzo[e][1,4]diazepin-5-one (H22)

Starting from **H8** (1 mmol), and following the procedure similar to that of preparation of **H20** to give **H22**;^1^H NMR (400 MHz, DMSO) δ 7.89 (t, *J* = 5.8 Hz, 1H), 7.62 (dd, *J* = 7.9, 1.2 Hz, 1H), 7.40–7.31 (m, 2H), 7.26–7.15 (m, 3H), 6.86 (d, *J* = 8.1 Hz, 1H), 6.69 (t, *J* = 7.4 Hz, 1H), 6.57 (d, *J* = 4.1 Hz, 1H), 4.82–4.75 (m, 1H), 3.46–3.32 (m, 2H); ^13 ^C NMR (101 MHz, DMSO) δ 170.80, 163.02, 160.61, 146.76, 139.98, 139.95, 132.39, 132.25, 129.23, 129.15, 119.52, 119.17, 117.20, 115.53, 115.32, 61.94, 45.79. ESI-MS *m/z* = 257.12 [M + H]^+^, 279.08 [M + Na]^+^.

##### 5.1.5.4. 2-(3-Fluorophenyl)-1,2,3,4-tetrahydro-5H-benzo[e][1,4]diazepin-5-one (H23)

Starting from **H9** (1 mmol), and following the procedure similar to that of preparation of **H20** to give **H23**;^1^H NMR (400 MHz, DMSO) δ 7.90 (t, *J* = 5.9 Hz, 1H), 7.62 (dd, *J* = 7.9, 1.4 Hz, 1H), 7.45–7.36 (m, 1H), 7.28–7.21 (m, 1H), 7.20–7.06 (m, 3H), 6.87 (d, *J* = 8.0 Hz, 1H), 6.70 (t, *J* = 7.1 Hz, 1H), 6.63 (d, *J* = 4.5 Hz, 1H), 4.86–4.79 (m, 1H), 3.48–3.41 (m, 2H); ^13 ^C NMR (101 MHz, DMSO) δ 170.78, 163.89, 161.47, 146.95, 146.88, 146.68, 132.43, 132.26, 130.71, 130.63, 123.35, 123.33, 119.55, 119.16, 117.31, 114.32, 114.17, 114.11, 113.96, 62.01, 45.42. ESI-MS *m/z* = 257.18 [M + H]^+^, 279.14 [M + Na]^+^.

##### 5.1.5.5. 2-(2,4-Difluorophenyl)-1,2,3,4-tetrahydro-5H-benzo[e][1,4]diazepin-5-one (H24)

Starting from **H14** (1 mmol), and following the procedure similar to that of preparation of **H20** to give **H24**; ^1^H NMR (400 MHz, DMSO) δ 7.89 (t, *J* = 5.5 Hz, 1H), 7.61 (d, *J* = 7.8 Hz, 1H), 7.39–7.21 (m, 3H), 7.10 (t, *J* = 8.5 Hz, 1H), 6.85 (d, *J* = 8.2 Hz, 1H), 6.71 (t, *J* = 7.4 Hz, 1H), 6.57 (d, *J* = 4.6 Hz, 1H), 5.02–4.94 (m, 1H), 3.54–3.33 (m, 2H); ^13 ^C NMR (101 MHz, DMSO) δ 170.72, 163.25, 163.12, 160.81, 160.69, 160.57, 158.24, 158.12, 146.59, 132.47, 132.23, 131.08, 126.72, 126.62, 119.55, 119.20, 117.45, 111.81, 111.57, 104.45, 104.19, 103.94, 60.23, 43.85. ESI-MS *m/z* = 296.96 [M + H]^+^.

##### 5.1.5.6. 2-(3,5-Difluorophenyl)-1,2,3,4-tetrahydro-5H-benzo[e][1,4]diazepin-5-one (H25)

Starting from **H15** (1 mmol), and following the procedure similar to that of preparation of **H20** to give **H25**; ^1^H NMR (400 MHz, DMSO) δ 7.88 (t, *J* = 5.9 Hz, 1H), 7.60 (dd, *J* = 7.9, 1.2 Hz, 1H), 7.29–7.20 (m, 1H), 7.18–7.09 (m, 1H), 7.07–6.98 (m, 2H), 6.85 (d, *J* = 8.1 Hz, 1H), 6.71 (t, *J* = 7.4 Hz, 1H), 6.65 (d, *J* = 5.0 Hz, 1H), 4.89–4.77 (m, 1H), 3.51–3.40 (m, 2H); ^13 ^C NMR (101 MHz, DMSO) δ 170.72, 164.07, 163.94, 161.63, 161.50, 148.93, 148.85, 148.77, 146.44, 132.48, 132.23, 119.68, 119.14, 117.55, 110.55, 110.49, 110.30, 103.05, 102.79, 102.53, 61.69, 44.91. ESI-MS *m/z* = 297.11 [M + H]^+^.

##### 5.1.5.7. 2-(3,4-Dimethoxyphenyl)-1,2,3,4-tetrahydro-5H-benzo[e][1,4]diazepin-5-one (H26)

Starting from **H16** (1 mmol), and following the procedure similar to that of preparation of **H20** to give **H26**; ^1^H NMR (400 MHz, DMSO) δ 7.91 (t, *J* = 5.9 Hz, 1H), 7.61 (dd, *J* = 7.9, 1.4 Hz, 1H), 7.25–7.18 (m, 1H), 6.95–6.90 (m, 2H), 6.88 (d, *J* = 8.0 Hz, 1H), 6.83 (dd, *J* = 8.3, 1.5 Hz, 1H), 6.67 (t, *J* = 7.2 Hz, 1H), 6.45 (d, *J* = 3.4 Hz, 1H), 4.71–4.64 (m, 1H), 3.73 (d, *J* = 2.7 Hz, 6H), 3.42 − 3.35 (m, 2H); ^13 ^C NMR (101 MHz, DMSO) δ 170.77, 149.07, 148.43, 146.85, 136.12, 132.30, 132.20, 119.55, 119.21, 117.06, 112.10, 111.01, 62.57, 56.04, 55.88, 46.21. ESI-MS *m/z* = 321.16 [M + H]^+^.

##### 5.1.5.8. 2-(4-Bromophenyl)-1,2,3,4-tetrahydro-5H-benzo[e][1,4]diazepin-5-one (H27)

Starting from **H19** (1 mmol), and following the procedure similar to that of preparation of **H20** to give **H27**; ^1^H NMR (400 MHz, DMSO) δ 7.86–7.74 (m, 1H), 7.61 (d, *J* = 7.8 Hz, 1H), 7.55 (d, *J* = 7.6 Hz, 2H), 7.27 (d, *J* = 7.7 Hz, 2H), 7.22 (t, *J* = 7.6 Hz, 1H), 6.85 (d, *J* = 8.2 Hz, 1H), 6.69 (t, *J* = 7.3 Hz, 1H), 6.55 (s, 1H), 4.83–4.71 (m, 1H), 3.48–3.33 (m, 2H); ^13 ^C NMR (101 MHz, DMSO) δ 170.75, 146.69, 143.26, 132.38, 132.27, 131.56, 129.58, 120.52, 119.51, 119.14, 117.25, 61.94, 45.46. ESI-MS *m/z* = 317.23, 319.05 [M + H]^+^, 341.02 [M + Na]^+^.

#### 5.1.6. Synthesis procedure for compounds H28–H31

##### 5.1.6.1. 2-(4-Bromophenyl)-4-methyl-1,2,3,4-tetrahydro-5H-benzo[e][1,4]diazepin-5-one (H28)

Compound **H27** (317 mg, 1 mmol) and 10 ml acetonitrile were added to 50 ml reaction flask to obtain uniform turbid solution. Then NaH (80 mg, 2 mmol) was added in batches in an ice bath and slowly warmed to room temperature. The acetonitrile solution of dimethyl sulphate (252 mg, 2 mmol) was then slowly dripped into the reaction bottle and reacted at room temperature for about 2 h. The progress of the reaction was monitored by TLC. After the reaction was completed, the inorganic salts were removed by filtration, and the residue was washed twice with ethyl acetate. The combined organic layers were concentrated and filtered to afford the desired compound. ^1^H NMR (400 MHz, DMSO) δ 7.60–7.52 (m, 3H), 7.33 (d, *J* = 8.4 Hz, 2H), 7.27–7.20 (m, 1H), 6.86 (d, *J* = 7.9 Hz, 1H), 6.74 (t, *J* = 7.4 Hz, 1H), 6.45 (d, *J* = 4.2 Hz, 1H), 4.95–4.89 (m, 1H), 3.75–3.56 (m, 2H), 2.63 (s, 3H); ^13 ^C NMR (101 MHz, DMSO) δ 169.29, 146.33, 142.84, 132.20, 131.98, 131.64, 129.42, 121.44, 120.68, 119.17, 117.91, 61.98, 53.57, 36.52. ESI-MS *m/z* = 331.23, 333.07 [M + H]^+^, 355.39 [M + Na]^+^.

##### 5.1.6.2. 2-(4-Bromophenyl)-1,4-dimethyl-1,2,3,4-tetrahydro-5H-benzo[e][1,4]diazepin-5-one (H29)

Formic acid (88%, 5 mmol) was added to the aqueous solution of **H27** (1 mmol) in an ice bath, and the mixture was slowly warmed until solution was obtained. Then 1.2 mmol CH2O (37%) was added at room temperature and the mixture was heated at 100 °C until the generation of CO2 ceased. The solution was cooled, acidified with concentrated HCl and evaporated. The residue was dissolved in H2O and alkalified with NaOH and extracted three times with ethyl acetate. The combined organic layers were washed with brine, dried over anhydrous Na_2_SO_4_. After filtration and concentration, the crude product was obtained and purified with column chromatography to afford the desired compound. ^1^H NMR (400 MHz, DMSO) δ 7.59–7.52 (m, 3H), 7.50–7.45 (m, 1H), 7.13 (d, *J* = 8.2 Hz, 2H), 7.05 (t, *J* = 7.4 Hz, 1H), 6.95 (d, *J* = 8.1 Hz, 1H), 5.03 (d, *J* = 9.8 Hz, 1H), 4.64–4.55 (m, 1H), 3.57–3.50 (m, 1H), 3.23 (s, 3H), 2.58 (s, 3H); ^13 ^C NMR (101 MHz, DMSO) δ 170.34, 146.53, 139.12, 132.34, 131.52, 130.25, 129.26, 128.49, 121.01, 120.89, 119.12, 76.65, 70.00, 50.94, 38.18. ESI-MS *m/z* = 345.08, 347.05 [M + H]^+^.

##### 5.1.6.3. 2-(4-Bromophenyl)-3-methyl-3,4-dihydro-5H-benzo[e][1,4]diazepin-5-one (H30)

Starting from **H30-003** (1 mmol), and following the procedure similar to that of preparation of **H4** to give **H30**; ^1^H NMR (400 MHz, DMSO) δ 8.70 (d, *J* = 7.3 Hz, 1H), 7.87 (d, *J* = 7.6 Hz, 3H), 7.75 (d, *J* = 8.5 Hz, 2H), 7.66–7.59 (m, 1H), 7.42–7.31 (m, 2H), 4.53 (s, 1H), 1.10 (d, *J* = 6.9 Hz, 3H); ^13 ^C NMR (101 MHz, DMSO) δ 169.76, 167.34, 145.93, 136.79, 132.46, 132.11, 130.66, 130.30, 127.06, 126.36, 125.06, 46.21, 15.91. ESI-MS *m/z* = 329.19, 331.77 [M + H]^+^, 351.02 [M + Na]^+^.

##### 5.1.6.4. 2-(4-Bromophenyl)-3-methyl-1,2,3,4-tetrahydro-5H-benzo[e][1,4]diazepin-5-one (H31)

Starting from **H30** (1 mmol), and following the procedure similar to that of preparation of **H20** to give **H31**; ^1^H NMR (400 MHz, DMSO) δ 7.64 (d, *J* = 5.5 Hz, 1H), 7.61–7.53 (m, 3H), 7.28–7.17 (m, 3H), 6.81 (d, *J* = 8.0 Hz, 1H), 6.71 (t, *J* = 7.4 Hz, 1H), 6.59 (d, *J* = 5.5 Hz, 1H), 4.58 (d, *J* = 3.8 Hz, 1H), 3.91–3.78 (m, 1H), 0.99 (d, *J* = 6.9 Hz, 3H); ^13 ^C NMR (101 MHz, DMSO) δ 169.70, 146.61, 141.33, 132.35, 131.84, 131.23, 130.97, 120.83, 120.37, 119.04, 117.56, 66.84, 49.08, 17.12. ESI-MS *m/z* = 331.23, 333.05 [M + H]^+^, 353.02, 355.00 [M + Na]^+^.

#### 5.1.7. Synthesis procedure for compounds H32–H55

##### 5.1.7.1. 2-(4-(1H-pyrazol-4-yl)phenyl)-3,4-dihydro-5H-benzo[e][1,4]diazepin-5-one (H32)

4-Pyrazole-pinacol borate (1.1 mmol, 1.1 eq), **H19** (1 mmol, 1 equiv.), Na_2_CO_3_ (2 mmol, 2 equiv.) and 10 ml dioxane were placed into a 50 ml three-necked bottle, the reaction system was evacuated and backfilled with argon three times. Pd(dppf)Cl_2_ (0.05 mmol, 0.05 eq) was added to the reaction bottle under argon flow. The resulting solution was heated to 90 °C and stirred at this temperature for 2 h. The reaction was monitored by TLC. The mixture was diluted with water and extracted with ethyl acetate. The combined organic layers were washed with brine, dried over anhydrous Na_2_SO_4_, and concentrated to give a crude product, which was purified by column chromatography to give compound **H32**; ^1^H NMR (400 MHz, DMSO) δ 13.11 (s, 1H), 8.60 (t, *J* = 5.9 Hz, 1H), 8.37 (s, 1H), 8.08 (d, *J* = 8.4 Hz, 3H), 7.87 (dd, *J* = 7.8, 1.2 Hz, 1H), 7.81 (d, *J* = 8.4 Hz, 2H), 7.66–7.58 (m, 1H), 7.40–7.31 (m, 2H), 3.97 (s, 2H). ^13 ^C NMR (101 MHz, DMSO) δ 168.54, 167.34, 147.06, 136.57, 133.82, 132.05, 130.33, 129.04, 128.63, 127.10, 127.01, 125.92, 125.62, 125.46, 120.85, 38.79. ESI-MS *m/z* = 303.13 [M + H]^+^.

##### 5.1.7.2. 2-(4-(1-Methyl-1H-pyrazol-4-yl)phenyl)-3,4-dihydro-5H-benzo[e][1,4]diazepin-5-one (H33)

This compound was synthesised by employing the procedure for **H32** using **H19** (1 mmol, 1 equiv.) and 1-methyl-4-pyrazole-pinacol borate (1.1 mmol, 1.1 eq) as the starting reagents; ^1^H NMR (400 MHz, DMSO) δ 8.59 (t, *J* = 6.0 Hz, 1H), 8.31 (s, 1H), 8.08 (d, *J* = 8.5 Hz, 2H), 8.02 (s, 1H), 7.87 (dd, *J* = 7.8, 1.4 Hz, 1H), 7.75 (d, *J* = 8.5 Hz, 2H), 7.66–7.58 (m, 1H), 7.40–7.30 (m, 2H), 3.96 (s, 2H), 3.89 (s, 3H). ^13 ^C NMR (101 MHz, DMSO) δ 168.54, 167.31, 147.05, 137.02, 136.21, 133.90, 132.05, 130.33, 129.09, 127.10, 127.00, 125.93, 125.43, 121.54, 38.78. ESI-MS *m/z* = 317.34 [M + H]^+^.

##### 5.1.7.3. 2-(4-(3,5-Dimethyl-1H-pyrazol-4-yl)phenyl)-3,4-dihydro-5H-benzo[e][1,4]diazepin-5-one (H34)

This compound was synthesised by employing the procedure for **H32** using **H19** (1 mmol, 1 equiv.) and 3,5-dimethyl-4-pyrazole-pinacol borate (1.1 mmol, 1.1 eq) as the starting reagents; ^1^H NMR (400 MHz, DMSO) δ 12.46 (s, 1H), 8.61 (t, *J* = 5.9 Hz, 1H), 8.12 (d, *J* = 8.3 Hz, 2H), 7.91–7.84 (m, 1H), 7.66–7.58 (m, 1H), 7.48 (d, *J* = 8.3 Hz, 2H), 7.41–7.31 (m, 2H), 3.98 (s, 2H), 2.26 (s, 6H). ^13 ^C NMR (101 MHz, DMSO) δ 168.52, 167.50, 147.05, 137.91, 133.77, 132.06, 130.34, 129.22, 128.60, 127.09, 127.01, 125.98, 116.63, 38.86, 26.81, 25.85. ESI-MS *m/z* = 331.10 [M + H]^+^.

##### 5.1.7.4. 2-(4-(1-Methyl-1H-pyrazol-5-yl)phenyl)-3,4-dihydro-5H-benzo[e][1,4]diazepin-5-one (H35)

This compound was synthesised by employing the procedure for **H32** using **H19** (1 mmol, 1 equiv.) and 1-methyl-5-pyrazole boric acid (1.1 mmol, 1.1 eq) as the starting reagents; ^1^H NMR (400 MHz, DMSO) δ 8.63 (t, *J* = 5.9 Hz, 1H), 8.19 (d, *J* = 8.3 Hz, 2H), 7.92–7.85 (m, 1H), 7.74 (d, *J* = 8.3 Hz, 2H), 7.64 (t, *J* = 7.6 Hz, 1H), 7.53 (d, *J* = 1.7 Hz, 1H), 7.43–7.34 (m, 2H), 6.55 (d, *J* = 1.7 Hz, 1H), 4.00 (s, 2H), 3.93 (s, 3H). ^13 ^C NMR (101 MHz, DMSO) δ 168.44, 167.33, 146.80, 142.33, 138.56, 136.26, 133.25, 132.13, 130.38, 129.12, 128.78, 127.12, 127.06, 126.29, 106.89, 38.93, 38.29. ESI-MS *m/z* = 317.11 [M + H]^+^.

##### 5.1.7.5. 2-(3'-Amino-[1,1'-biphenyl]-4-yl)-3,4-dihydro-5H-benzo[e][1,4]diazepin-5-one (H36)

This compound was synthesised by employing the procedure for **H32** using **H19** (1 mmol, 1 equiv.) and 3-aminophenylboronic acid pinacol ester (1.1 mmol, 1.1 eq) as the starting reagents; ^1^H NMR (400 MHz, DMSO) δ 8.62 (t, *J* = 5.9 Hz, 1H), 8.15 (d, *J* = 8.4 Hz, 2H), 7.89 (dd, *J* = 7.8, 1.2 Hz, 1H), 7.75 (d, *J* = 8.4 Hz, 2H), 7.67–7.58 (m, 1H), 7.43–7.32 (m, 2H), 7.15 (t, *J* = 7.8 Hz, 1H), 6.98–6.93 (m, 1H), 6.89 (d, *J* = 7.6 Hz, 1H), 6.63 (dd, *J* = 7.9, 1.3 Hz, 1H), 5.26 (s, 2H), 3.99 (s, 2H); ^13 ^C NMR (101 MHz, DMSO) δ 168.51, 167.45, 149.75, 146.98, 144.27, 140.20, 135.23, 132.09, 130.36, 130.05, 128.95, 127.17, 127.11, 127.03, 126.09, 114.92, 114.33, 112.53, 60.24, 38.89, 21.24, 14.56. ESI-MS *m/z* = 328.36 [M + H]^+^.

##### 5.1.7.6. 2-(4′-Amino-[1,1′-biphenyl]-4-yl)-3,4-dihydro-5H-benzo[e][1,4]diazepin-5-one (H37)

This compound was synthesised by employing the procedure for **H32** using **H19** (1 mmol, 1 equiv.) and 4-aminophenylboronic acid pinacol ester (1.1 mmol, 1.1 eq) as the starting reagents; ^1^H NMR (400 MHz, DMSO) δ 8.56 (t, *J* = 5.9 Hz, 1H), 8.08 (d, *J* = 8.4 Hz, 2H), 7.87 (dd, *J* = 7.8, 1.2 Hz, 1H), 7.73 (d, *J* = 8.5 Hz, 2H), 7.64–7.57 (m, 1H), 7.50 (d, *J* = 8.5 Hz, 2H), 7.40–7.29 (m, 2H), 6.68 (d, *J* = 8.5 Hz, 2H), 5.39 (s, 2H), 3.96 (d, *J* = 4.6 Hz, 2H); ^13 ^C NMR (101 MHz, DMSO) δ 168.56, 167.36, 149.72, 147.13, 143.91, 133.59, 132.03, 130.33, 128.98, 127.95, 127.10, 127.02, 126.31, 125.87, 125.68, 114.67, 38.81. ESI-MS *m/z* = 328.29 [M + H]^+^.

##### 5.1.7.7. 2-(4-(6-Aminopyridin-3-yl)phenyl)-3,4-dihydro-5H-benzo[e][1,4]diazepin-5-one (H38)

This compound was synthesised by employing the procedure for **H32** using **H19** (1 mmol, 1 equiv.) and 2-aminopyridine-5-borate pinacol ester (1.1 mmol, 1.1 eq) as the starting reagents; ^1^H NMR (400 MHz, DMSO) δ 8.71–8.52 (m, 1H), 8.41 (s, 1H), 8.11 (d, *J* = 6.9 Hz, 2H), 7.93–7.71 (m, 4H), 7.68–7.54 (m, 1H), 7.43–7.26 (m, 2H), 6.57 (d, *J* = 7.5 Hz, 1H), 6.26 (s, 2H), 3.97 (s, 2H). ^13 ^C NMR (101 MHz, DMSO) δ 168.53, 167.36, 160.18, 147.04, 146.74, 141.42, 135.84, 134.22, 132.07, 130.34, 129.08, 127.11, 127.03, 125.98, 125.71, 122.98, 108.50, 38.83. ESI-MS *m/z* = 329.01 [M + H]^+^.

##### 5.1.7.8. 2-(4-(2-Aminopyrimidin-5-yl)phenyl)-3,4-dihydro-5H-benzo[e][1,4]diazepin-5-one (H39)

This compound was synthesised by employing the procedure for **H32** using **H19** (1 mmol, 1 equiv.) and 2-aminopyrimidine-5-borate pinacol ester (1.1 mmol, 1.1 eq) as the starting reagents; ^1^H NMR (400 MHz, DMSO) δ 8.72 (s, 2H), 8.62 (t, *J* = 5.9 Hz, 1H), 8.14 (d, *J* = 8.4 Hz, 2H), 7.90–7.81 (m, 3H), 7.67–7.58 (m, 1H), 7.41–7.31 (m, 2H), 6.98 (s, 2H), 3.98 (s, 2H). ^13 ^C NMR (101 MHz, DMSO) δ 168.50, 167.34, 163.66, 156.70, 146.96, 138.60, 134.87, 132.09, 130.35, 129.13, 127.11, 127.04, 126.07, 125.64, 121.29, 38.86. ESI-MS *m/z* = 330.19 [M + H]^+^.

##### 5.1.7.9. 2-(4-(5-Amino-6-methoxypyridin-3-yl)phenyl)-3,4-dihydro-5H-benzo[e][1,4]diazepin-5-one (H40)

This compound was synthesised by employing the procedure for **H32** using **H19** (1 mmol, 1 equiv.) and 3-amino-2-methoxypyridine-5-borate pinacol ester (1.1 mmol, 1.1 eq) as the starting reagents; ^1^H NMR (400 MHz, DMSO) δ 8.62 (t, *J* = 5.9 Hz, 1H), 8.15 (d, *J* = 8.5 Hz, 2H), 7.88 (dd, *J* = 7.8, 1.3 Hz, 1H), 7.79 (d, *J* = 2.2 Hz, 1H), 7.75 (d, *J* = 8.5 Hz, 2H), 7.66–7.60 (m, 1H), 7.41–7.33 (m, 2H), 7.25 (d, *J* = 2.2 Hz, 1H), 5.17 (s, 2H), 3.98 (s, 2H), 3.92 (s, 3H). ^13 ^C NMR (101 MHz, DMSO) δ 168.50, 167.39, 152.51, 146.96, 141.38, 135.11, 133.12, 132.09, 131.06, 130.36, 129.38, 129.06, 127.11, 127.03, 126.81, 126.08, 117.15, 53.55, 38.88. ESI-MS *m/z* = 359.03 [M + H]^+^.

##### 5.1.7.10. 2-(3′-Methoxy-[1,1′-biphenyl]-4-yl)-3,4-dihydro-5H-benzo[e][1,4]diazepin-5-one (H41)

This compound was synthesised by employing the procedure for **H32** using **H19** (1 mmol, 1 equiv.) and 3-methoxyphenylboronic acid (1.1 mmol, 1.1 eq) as the starting reagents; ^1^H NMR (400 MHz, DMSO) δ 8.62–8.54 (m, 1H), 8.16 (d, *J* = 7.9 Hz, 2H), 7.92–7.82 (m, 3H), 7.62 (t, *J* = 7.5 Hz, 1H), 7.46–7.26 (m, 5H), 7.00 (d, *J* = 8.0 Hz, 1H), 3.99 (d, *J* = 4.1 Hz, 2H), 3.85 (s, 3H); ^13 ^C NMR (101 MHz, DMSO) δ 168.49, 167.42, 160.33, 146.94, 143.16, 141.02, 135.72, 132.07, 130.61, 130.36, 128.98, 127.56, 127.14, 127.05, 126.13, 119.66, 114.21, 112.86, 55.69, 38.95. ESI-MS *m/z* = 343.18 [M + H]^+^.

##### 5.1.7.11. 2-(4′-Methoxy-[1,1′-biphenyl]-4-yl)-3,4-dihydro-5H-benzo[e][1,4]diazepin-5-one (H42)

This compound was synthesised by employing the procedure for **H32** using **H19** (1 mmol, 1 equiv.) and 4-methoxyphenylboronic acid (1.1 mmol, 1.1 eq) as the starting reagents; ^1^H NMR (400 MHz, DMSO) δ 8.58 (t, *J* = 5.9 Hz, 1H), 8.14 (d, *J* = 8.3 Hz, 2H), 7.88 (d, *J* = 7.6 Hz, 1H), 7.81 (d, *J* = 8.3 Hz, 2H), 7.73 (d, *J* = 8.6 Hz, 2H), 7.65–7.59 (m, 1H), 7.40–7.32 (m, 2H), 7.06 (d, *J* = 8.7 Hz, 2H), 3.98 (d, *J* = 5.0 Hz, 2H), 3.81 (s, 3H); ^13 ^C NMR (101 MHz, DMSO) δ 168.51, 167.40, 159.99, 147.00, 142.95, 134.81, 132.07, 131.74, 130.36, 129.03, 128.50, 127.12, 127.04, 126.77, 126.04, 114.99, 55.72, 38.89. ESI-MS *m/z* = 343.14 [M + H]^+^.

##### 5.1.7.12. 2-(3′-(Trifluoromethyl)-[1,1′-biphenyl]-4-yl)-3,4-dihydro-5H-benzo[e][1,4]diazepin-5-one (H43)

This compound was synthesised by employing the procedure for **H32** using **H19** (1 mmol, 1 equiv.) and 3-(trifluoromethyl)phenylboronic acid (1.1 mmol, 1.1 eq) as the starting reagents; ^1^H NMR (400 MHz, DMSO) δ 8.60 (t, *J* = 5.9 Hz, 1H), 8.21 (d, *J* = 8.4 Hz, 2H), 8.13–8.07 (m, 2H), 7.97 (d, *J* = 8.4 Hz, 2H), 7.92–7.87 (m, 1H), 7.82–7.72 (m, 2H), 7.67–7.61 (m, 1H), 7.43–7.34 (m, 2H), 4.01 (d, *J* = 5.3 Hz, 2H); ^13 ^C NMR (101 MHz, DMSO) δ 168.45, 167.38, 146.86, 141.54, 140.60, 136.35, 132.09, 131.48, 130.66, 130.37, 129.13, 127.82, 127.14, 127.06, 126.23, 125.20, 123.77, 38.97. ESI-MS *m/z* = 381.11 [M + H]^+^.

##### 5.1.7.13. 2-(4′-(Trifluoromethyl)-[1,1′-biphenyl]-4-yl)-3,4-dihydro-5H-benzo[e][1,4]diazepin-5-one (H44)

This compound was synthesised by employing the procedure for **H32** using **H19** (1 mmol, 1 equiv.) and 4-(trifluoromethyl)phenylboronic acid (1.1 mmol, 1.1 eq) as the starting reagents; ^1^H NMR (400 MHz, DMSO) δ 8.59 (t, *J* = 5.9 Hz, 1H), 8.21 (d, *J* = 8.4 Hz, 2H), 8.00 (d, *J* = 8.2 Hz, 2H), 7.96–7.82 (m, 5H), 7.67–7.58 (m, 1H), 7.42–7.32 (m, 2H), 4.00 (d, *J* = 5.2 Hz, 2H); ^13 ^C NMR (101 MHz, DMSO) δ 168.45, 167.36, 146.84, 143.52, 141.57, 136.54, 132.09, 130.37, 129.14, 128.16, 127.88, 127.72, 127.58, 127.15, 127.06, 126.36, 126.32, 38.96. ESI-MS *m/z* = 381.30 [M + H]^+^.

##### 5.1.7.14. 2-(4-(1-Methyl-1,2,3,6-tetrahydropyridin-4-yl)phenyl)-3,4-dihydro-5H-benzo[e][1,4]diazepin-5-one (H45)

This compound was synthesised by employing the procedure for **H32** using **H19** (1 mmol, 1 equiv.) and 1-methyl-1, 2, 3, 6-tetrahydropyridine-4-borate pinacol ester (1.1 mmol, 1.1 eq) as the starting reagents; ^1^H NMR (400 MHz, DMSO) δ 8.63 (t, *J* = 5.6 Hz, 1H), 8.05 (d, *J* = 8.4 Hz, 2H), 7.90–7.83 (m, 1H), 7.65–7.56 (m, 3H), 7.39–7.30 (m, 2H), 6.36 (s, 1H), 3.97 (d, *J* = 22.2 Hz, 2H), 3.05 (d, *J* = 2.6 Hz, 2H), 2.61–2.55 (m, 2H), 2.54–2.48 (m, 2H), 2.28 (s, 3H). ^13 ^C NMR (101 MHz, DMSO) δ 168.49, 167.36, 146.97, 143.27, 134.98, 133.40, 132.06, 130.34, 128.54, 127.10, 127.01, 126.03, 125.23, 124.58, 55.13, 52.16, 45.87, 38.81, 27.68. ESI-MS *m/z* = 332.09 [M + H]^+^.

##### 5.1.7.15. 2-(4-(9H-carbazol-3-yl)phenyl)-3,4-dihydro-5H-benzo[e][1,4]diazepin-5-one (H46)

This compound was synthesised by employing the procedure for **H32** using **H19** (1 mmol, 1 equiv.) and 9*H*-carbazole-3-boronic acid pinacol ester (1.1 mmol, 1.1 eq) as the starting reagents; ^1^H NMR (400 MHz, DMSO) δ 11.44 (s, 1H), 8.66 (t, *J* = 6.0 Hz, 1H), 8.61 (d, *J* = 1.3 Hz, 1H), 8.27 (d, *J* = 7.7 Hz, 1H), 8.20 (d, *J* = 8.5 Hz, 2H), 7.99 (d, *J* = 8.5 Hz, 2H), 7.93–7.81 (m, 2H), 7.67–7.58 (m, 2H), 7.53 (d, *J* = 8.1 Hz, 1H), 7.47–7.33 (m, 3H), 7.21 (t, *J* = 7.4 Hz, 1H), 4.06–3.96 (m, 2H). ^13 ^C NMR (101 MHz, DMSO) δ 168.56, 167.47, 147.07, 144.51, 140.74, 140.21, 134.39, 132.09, 130.37, 130.05, 129.07, 127.17, 127.13, 127.06, 126.39, 126.01, 125.16, 123.66, 123.06, 121.05, 119.30, 119.12, 111.94, 111.65, 38.89. ESI-MS *m/z* = 402.46 [M + H]^+^.

##### 5.1.7.16. 2-(3′-Amino-[1,1′-biphenyl]-4-yl)-1,2,3,4-tetrahydro-5H-benzo[e][1,4]diazepin-5-one (H47)

This compound was synthesised by employing the procedure for **H32** using **H27** (1 mmol, 1 equiv.) and (3-aminophenyl)boronic acid (1.1 mmol, 1.1 eq) as the starting reagents; ^1^H NMR (400 MHz, DMSO) δ 7.92 (t, *J* = 5.9 Hz, 1H), 7.64 (dd, *J* = 7.9, 1.3 Hz, 1H), 7.54 (d, *J* = 8.2 Hz, 2H), 7.37 (d, *J* = 8.2 Hz, 2H), 7.27–7.19 (m, 1H), 7.09 (t, *J* = 7.8 Hz, 1H), 6.88 (d, *J* = 8.1 Hz, 1H), 6.83 (s, 1H), 6.77 (d, *J* = 7.7 Hz, 1H), 6.69 (t, *J* = 7.2 Hz, 1H), 6.60–6.52 (m, 2H), 5.15 (s, 2H), 4.83–4.76 (m, 1H), 3.50–3.39 (m, 2H); ^13 ^C NMR (101 MHz, DMSO) δ 170.83, 149.59, 146.89, 142.60, 141.16, 140.41, 132.38, 132.30, 129.88, 127.72, 126.83, 119.40, 119.20, 117.08, 114.75, 113.56, 112.48, 62.51, 45.91. ESI-MS *m/z* = 330.30 [M + H]^+^.

##### 5.1.7.17. 2-(4′-Amino-[1,1′-biphenyl]-4-yl)-1,2,3,4-tetrahydro-5H-benzo[e][1,4]diazepin-5-one (H48)

This compound was synthesised by employing the procedure for **H32** using **H27** (1 mmol, 1 equiv.) and 4-aminophenylboronic acid pinacol ester (1.1 mmol, 1.1 eq) as the starting reagents**;** ^1^H NMR (400 MHz, DMSO) δ 7.95–7.85(m, 1H), 7.63 (d, *J* = 7.8 Hz, 1H), 7.51 (d, *J* = 7.1 Hz, 2H), 7.40–7.26 (m, 4H), 7.22 (t, *J* = 7.5 Hz, 1H), 6.88 (d, *J* = 8.1 Hz, 1H), 6.73–6.59 (m, 3H), 6.53 (s, 1H), 5.21 (s, 2H), 4.81 − 4.69(m, 1H), 3.50–3.38 (m, 2H); ^13 ^C NMR (101 MHz, DMSO) δ 170.82, 148.81, 146.92, 141.03, 140.10, 132.36, 132.29, 127.67, 127.55, 125.72, 119.36, 119.20, 117.02, 114.70, 62.53, 46.03. ESI-MS *m/z* = 330.49 [M + H]^+^.

##### 5.1.7.18. 2-(3′-Methoxy-[1,1′-biphenyl]-4-yl)-1,2,3,4-tetrahydro-5H-benzo[e][1,4]diazepin-5-one (H49)

This compound was synthesised by employing the procedure for **H32** using **H27** (1 mmol, 1 equiv.) and 3-methoxyphenylboronic acid (1.1 mmol, 1.1 eq) as the starting reagents; ^1^H NMR (400 MHz, DMSO) δ 7.91 (t, *J* = 5.9 Hz, 1H), 7.70–7.61 (m, 3H), 7.43–7.34 (m, 3H), 7.28–7.15 (m, 3H), 6.96–6.86 (m, 2H), 6.69 (t, *J* = 7.4 Hz, 1H), 6.58 (d, *J* = 3.9 Hz, 1H), 4.86–4.77 (m, 1H), 3.82 (s, 3H), 3.53–3.39 (m, 2H); ^13 ^C NMR (101 MHz, DMSO) δ 170.82, 160.22, 146.89, 143.16, 141.95, 139.36, 132.40, 132.31, 130.48, 127.85, 127.17, 119.43, 119.39, 119.20, 117.11, 113.33, 112.63, 62.40, 55.57, 45.85. ESI-MS *m/z* = 345.35 [M + H]^+^.

##### 5.1.7.19. 2-(4′-Methoxy-[1,1′-biphenyl]-4-yl)-1,2,3,4-tetrahydro-5H-benzo[e][1,4]diazepin-5-one (H50)

This compound was synthesised by employing the procedure for **H32** using **H27** (1 mmol, 1 equiv.) and 4-methoxyphenylboronic acid (1.1 mmol, 1.1 eq) as the starting reagents; ^1^H NMR (400 MHz, DMSO) δ 7.90 (t, *J* = 5.8 Hz, 1H), 7.66–7.56 (m, 5H), 7.36 (d, *J* = 8.1 Hz, 2H), 7.26–7.18 (m, 1H), 7.02 (d, *J* = 8.7 Hz, 2H), 6.89 (d, *J* = 8.2 Hz, 1H), 6.69 (t, *J* = 7.4 Hz, 1H), 6.56 (d, *J* = 3.8 Hz, 1H), 4.84–4.75 (m, 1H), 3.79 (s, 3H), 3.48–3.40 (m, 2H); ^13 ^C NMR (101 MHz, DMSO) δ 170.83, 159.32, 146.90, 142.21, 139.15, 132.78, 132.38, 132.30, 128.12, 127.83, 126.54, 119.40, 119.20, 117.07, 114.85, 62.43, 55.63, 45.91. ESI-MS *m/z* = 345.35 [M + H]^+^.

##### 5.1.7.20. 2-(3′-(Trifluoromethyl)-[1,1′-biphenyl]-4-yl)-1,2,3,4-tetrahydro-5H-benzo[e][1,4]diazepin-5-one (H51)

This compound was synthesised by employing the procedure for **H32** using **H27** (1 mmol, 1 equiv.) and 3-(trifluoromethyl)phenylboronic acid (1.1 mmol, 1.1 eq) as the starting reagents; ^1^H NMR (400 MHz, DMSO) δ 8.02–7.93 (m, 2H), 7.90 (t, *J* = 5.4 Hz, 1H), 7.80–7.67 (m, 4H), 7.64 (d, *J* = 7.8 Hz, 1H), 7.45 (d, *J* = 7.9 Hz, 2H), 7.24 (t, *J* = 7.6 Hz, 1H), 6.89 (d, *J* = 8.2 Hz, 1H), 6.69 (t, *J* = 7.4 Hz, 1H), 6.64–6.56 (m, 1H), 4.90–4.79 (m, 1H), 3.53–3.41 (m, 2H); ^13 ^C NMR (101 MHz, DMSO) δ 170.82, 146.86, 143.96, 141.45, 137.78, 132.40, 132.31, 131.16, 130.57, 128.11, 127.36, 123.36, 119.47, 119.20, 117.16, 62.33, 45.74. ESI-MS *m/z* = 383.24 [M + H]^+^.

##### 5.1.7.21. 2-(4′-(Trifluoromethyl)-[1,1′-biphenyl]-4-yl)-1,2,3,4-tetrahydro-5H-benzo[e][1,4]diazepin-5-one (H52)

This compound was synthesised by employing the procedure for **H32** using **H27** (1 mmol, 1 equiv.) and 4-(trifluoromethyl)phenylboronic acid (1.1 mmol, 1.1 eq) as the starting reagents; ^1^H NMR (400 MHz, DMSO) δ 7.97–7.86 (m, 3H), 7.81 (d, *J* = 8.1 Hz, 2H), 7.74 (d, *J* = 7.8 Hz, 2H), 7.66 (d, *J* = 7.8 Hz, 1H), 7.46 (d, *J* = 7.9 Hz, 2H), 7.25 (t, *J* = 7.6 Hz, 1H), 6.91 (d, *J* = 8.2 Hz, 1H), 6.70 (t, *J* = 7.4 Hz, 1H), 6.62 (d, *J* = 3.7 Hz, 1H), 4.92–4.80 (m, 1H), 3.54–3.42 (m, 2H); ^13 ^C NMR (101 MHz, DMSO) δ 170.86, 146.86, 144.39, 144.19, 137.81, 132.41, 132.32, 128.38, 128.12, 127.80, 127.43, 126.28, 126.25, 126.21, 119.45, 119.20, 117.16, 62.34, 45.71. ESI-MS *m/z* = 383.20 [M + H]^+^.

##### 5.1.7.22. 2-(4-(4-Isopropylpiperazin-1-yl)phenyl)-1,2,3,4-tetrahydro-5H-benzo[e][1,4]diazepin-5-one (H53)

**H27** (1 mmol, 1 equiv.), NaO*t*Bu (2 mmol, 2 equiv.), Pd(OAc)_2_ (0.05 mmol, 0.05 eq) and RuPhos (0.1 mmol, 0.11 eq) were placed into a 10 ml Schlenk tube. The tube was evacuated and backfilled with argon three times, then 1-isopropylpiperazine (2 mmol, 2 equiv.) and dioxane (4 ml) were added via syringe under argon flow . The reaction mixture was heated at 100 °C for 24 h under vigorous stirring. The cooled solution was diluted with ethyl acetate, washed with brine. The organic phase was dried over anhydrous Na_2_SO_4_, concentrated *in vacuo*, and purified by column chromatography to afford the corresponding compound 7 m; ^1^H NMR (400 MHz, DMSO) δ 7.90 (t, *J* = 5.4 Hz, 1H), 7.60 (d, *J* = 7.6 Hz, 1H), 7.24–7.08 (m, 3H), 6.97–6.79 (m, 3H), 6.65 (t, *J* = 7.3 Hz, 1H), 6.46–6.35 (m, 1H), 4.70–4.55 (m, 1H), 3.48–3.32 (m, 2H), 3.18–2.99 (m, 4H), 2.75–2.63 (m, 1H), 2.62–2.51 (m, 4H), 1.00 (d, *J* = 6.4 Hz, 6H); ^13 ^C NMR (101 MHz, DMSO) δ 170.76, 150.86, 146.93, 133.89, 132.28, 132.24, 127.79, 119.26, 119.20, 116.87, 115.71, 62.43, 54.21, 49.19, 48.54, 46.35, 18.68. ESI-MS *m/z* = 365.24 [M + H]^+^.

##### 5.1.7.23. 2-(4-(4-Acetylpiperazin-1-yl)phenyl)-1,2,3,4-tetrahydro-5H-benzo[e][1,4]diazepin-5-one (H54)

This compound was synthesised by employing the procedure for **H53** using **H27** (1 mmol, 1 equiv.) and 1-(piperazin-1-yl)ethanone (2 mmol, 2 equiv.) as the starting reagents; ^1^H NMR (400 MHz, DMSO) δ 7.90 (t, *J* = 5.6 Hz, 1H), 7.61 (d, *J* = 7.8 Hz, 1H), 7.25–7.12 (m, 3H), 6.96 (d, *J* = 8.5 Hz, 2H), 6.85 (d, *J* = 8.1 Hz, 1H), 6.66 (t, *J* = 7.4 Hz, 1H), 6.44 (d, *J* = 2.8 Hz, 1H), 4.72–4.58 (m, 1H), 3.64–3.50 (m, 4H), 3.35–3.27 (m, 2H), 3.16–3.01 (m, 4H), 2.04 (s, 3H); ^13 ^C NMR (101 MHz, DMSO) δ 170.77, 168.70, 150.57, 146.91, 134.57, 132.30, 132.25, 127.89, 119.27, 119.19, 116.91, 116.33, 62.35, 49.39, 49.03, 46.27, 45.94, 41.12, 21.68. ESI-MS *m/z* = 387.20 [M + H]^+^.

##### 5.1.7.24. 2-(4-((2S,6R)-2,6-dimethylmorpholino)phenyl)-1,2,3,4-tetrahydro-5H-benzo[e][1,4]diazepin-5-one (H55)

This compound was synthesised by employing the procedure for **H53** using **H27** (1 mmol, 1 equiv.) and (2S,6R)-2,6-dimethylmorpholine (2 mmol, 2 equiv.) as the starting reagents; ^1^H NMR (400 MHz, CDCl_3_) δ 7.90–7.82 (m, 1H), 7.33–7.20 (m, 4H), 6.94–6.84 (m, 3H), 6.66 (d, *J* = 8.0 Hz, 1H), 6.60 (t, *J* = 5.8 Hz, 1H), 4.74 (t, *J* = 4.7 Hz, 1H), 4.16 (s, 1H), 3.86–3.74 (m, 2H), 3.52 (t, *J* = 5.6 Hz, 2H), 3.48–3.40 (m, 1H), 2.41 (t, *J* = 11.1 Hz, 2H), 1.27 (d, *J* = 6.3 Hz, 6H); ^13 ^C NMR (101 MHz, CDCl_3_) δ 172.04, 150.84, 145.29, 133.16, 132.91, 132.23, 127.55, 120.27, 119.27, 115.80, 71.60, 64.22, 54.61, 54.55, 47.04, 19.09. ESI-MS *m/z* = 352.23 [M + H]^+^, 374.34 [M + Na]^+^.

### 5.2. Virtual computational studies

#### 5.2.1. Molecular docking

The X-ray cocrystal structure of PARP-1 (PDB code: 4zzz) was obtained from the RCSB Protein Data Bank. The protein was prepared by deleting waters and adding hydrogen atoms using BIOVIA Discovery Studio 2017 R2 (DS 2017). The preparation of ligands was conducted by ChemDraw 19.0, DS 2017 and LigPrep (Schrödinger @2018). After the preliminary protein preparation and receptor grid generation were completed, the docking calculations were completed through Glide flexible docking. The other parameters were set to the default values. There were 20 docking conformations per docking run, and the results were ranked based on the value of their docking scores. Finally, the graphical display was processed by PyMOL.

#### 5.2.2. Prediction of ADME parameters

The preparation of ligands and the prediction of ADME parameters of compounds were carried out with LigPrep and QikProp, respectively (Schrodinger Suite 2018). The LigPrep process consists of a series of steps that perform conversions, apply corrections to the structures, generate variations on the structures, eliminate unwanted structures, and optimise the structures. Many of the steps are optional and are controlled by selecting options in the LigPrep panel or by specifying command-line options. QikProp can efficiently evaluate a widely applicable set of physically significant descriptors and pharmaceutically relevant properties, making it an indispensable tool for applying ADME principles in lead discovery and optimisation. We output all the parameters according to the user's manual and the parameters related to BBB were displayed.

### 5.3. Biological evaluation

#### 5.3.1. PARP-1 enzyme inhibition assay

The ability of the compounds to inhibit PARP-1 enzyme activity was assessed using BPS Bioscience's PARP Colorimetric Assay Kit (BPS Bioscience, Catalog # 80580). PARP-1 is known to catalyse the NAD-dependent addition of poly(ADP-ribose) to histones. The PARP-1 assay kit comes in a convenient 96-well format, with purified PARP-1 enzyme, histone mixture, activated DNA, and PARP-1 assay buffer. The key to the PARP-1 Colorimetric Activity Assay is the biotinylated substrate. With this kit, only three simple steps are required for PARP-1 reactions. First, histone proteins are coated on a 96-well plate. Next, the PARP-1 biotinylated substrate is incubated with an assay buffer that contains the PARP-1 enzyme. Finally, the plate is treated with streptavidin-HRP followed by the addition of the colorimetric HRP substrate to produce colour that can then be measured using a UV/Vis spectrophotometer microplate reader. IC_50_ values were calculated using GraphPad Prism5 software.

#### 5.3.2. Cellular inhibition assay

The MTT assay was performed to detect the sensitivity of cells to anticancer drugs *in vitro*. The human A549, MCF7, and U87 tumour cells, as well as human BRCA mutant tumour cells (MX-1), were tested in this study. The cell culture was carried out in accordance with routine operations or instructions. Briefly, 5,000 cells were seeded in each well of a 96-well plate. After cell attachment to the bottom of the well, different concentrations of tested compounds were added into designated wells. After 48 h of incubation, 20 µL of MTT solution (5 mg/mL) were added to each well for cell staining. After 4 h, the media were removed and 150 µL of DMSO were added to each well. Finally, the absorbance was determined at 570 nm using a multifunctional microplate reader (TECAN).

#### 5.3.3. MDCK cell permeability assay

The MDCK cells were cultured in accordance with the instructions, and the integrity of the membrane was evaluated by measuring the trans-epithelial electrical resistance (TEER) using an EVOM epithelial volt-ohmmeter with STX2 electrodes. The tested compounds (10 µM) of 100 µL were added to the apical compartment of the transwell cell culture devices (Millipore), and then the devices were incubated at 37 °C for 3 h. The concentrations of tested compounds in an apical compartment and basolateral compartment were then detected by HPLC, and the apparent permeability was then calculated. The calculation was *P*_app_ (A→B) = (Δ*Q*/Δ*t*)/(*A*·*C_0_*) where Δ*Q* is the amount of compound in the basolateral compartment (µg), Δ*t* is incubation time (s), *A* is the surface area of transwell in cm^2^ (0.33 cm^2^), and *C*_0_ is starting concentration (10 µM).

#### 5.3.4. Brain/plasma pharmacokinetic studies in vivo

All animal experiments were complied with the ARRIVE guidelines and were carried out in accordance with the U.K. Animals Act, 1986 and associated guidelines. The ICR mice were used in plasma–brain distribution assay of compounds. After tail vein injection of a 1.5 mg/kg dose, the concentrations of the compounds in the brain and plasma were measured after 5 min and 60 min of administration, and the brain/plasma (B/P) ratio was calculated. The samples of plasma and brain tissue were prepared as previously reported^47^. An established LC-MS/MS method was used for determining the concentration of representative compounds in plasma and brain tissue.

## Supplementary Material

Supplemental Material
