# Supplementary material for: Design and synthesis of benzodiazepines as brain penetrating PARP-1 inhibitors
Source: J Enzyme Inhib Med Chem. 2022 Mar 22;37(1):952–72. doi: 10.1080/14756366.2022.2053524 (PMC8942544; doi:10.1080/14756366.2022.2053524)
Supplement: Supplemental Material [file IENZ_A_2053524_SM1442.pdf]

Supplementary data

Design and synthesis of benzodiazepines as brain penetrating PARP-1 inhibitors

^{1}H-NMR, ^{13}C-NMR and Mass Spectra of Target Compounds

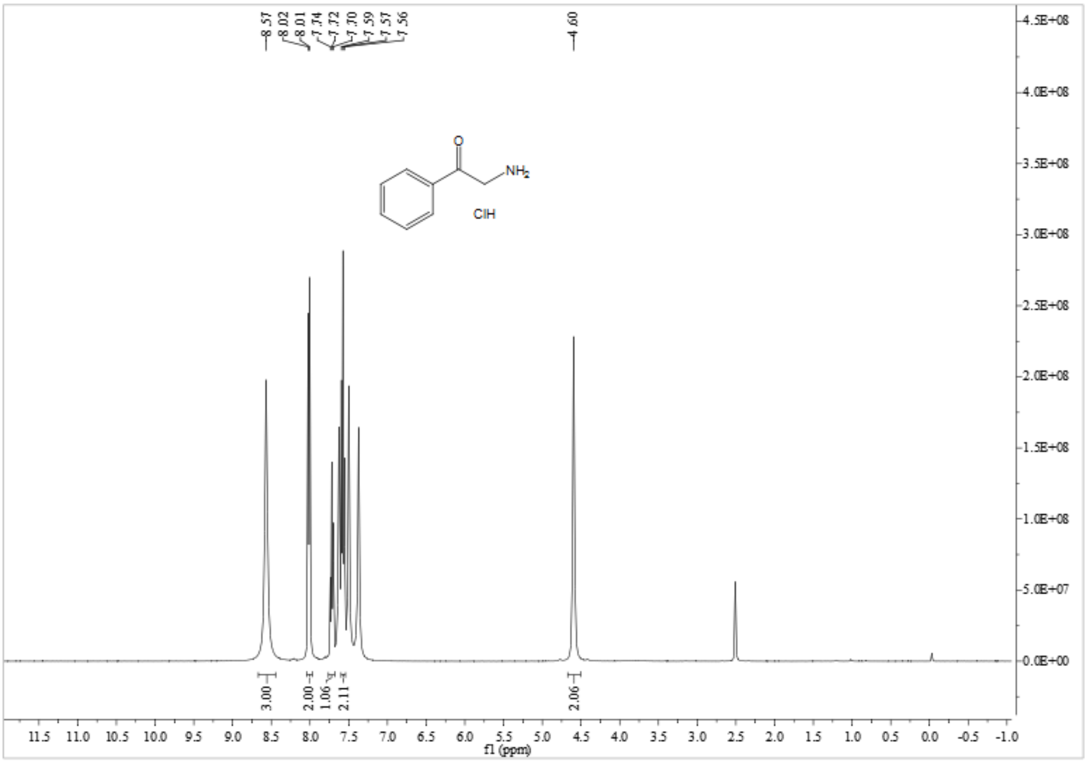

^{1}H-NMR spectrum of compound H4-002

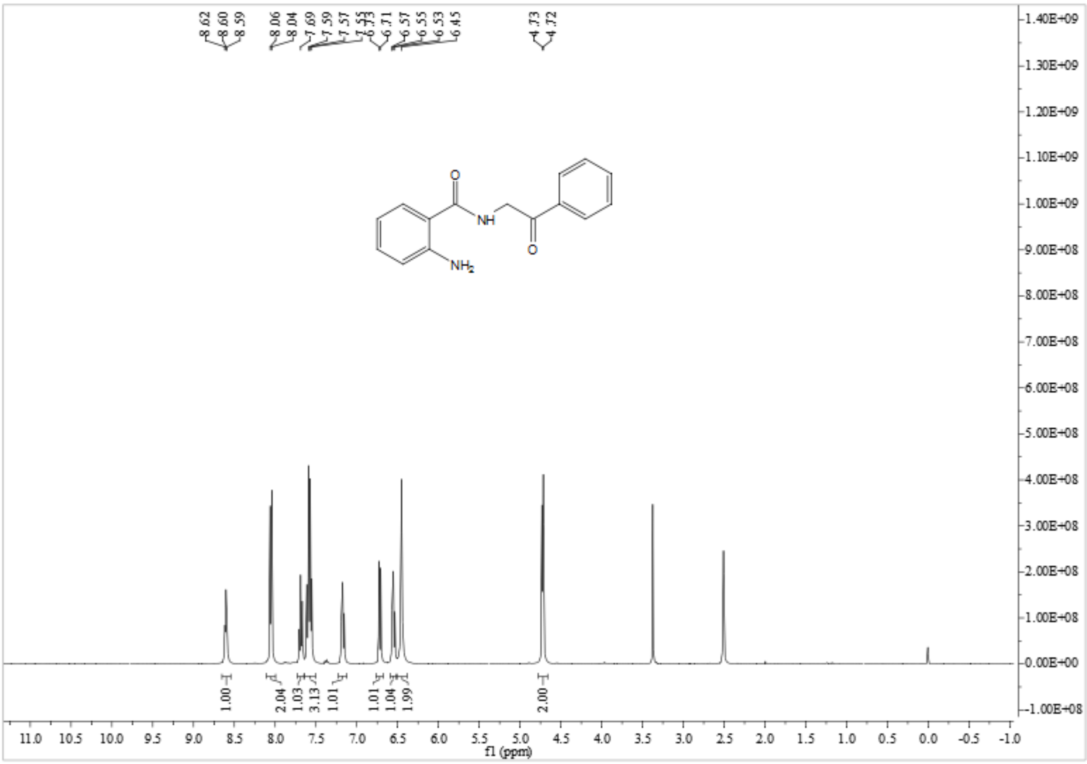

^{1}H-NMR spectrum of compound H4-003

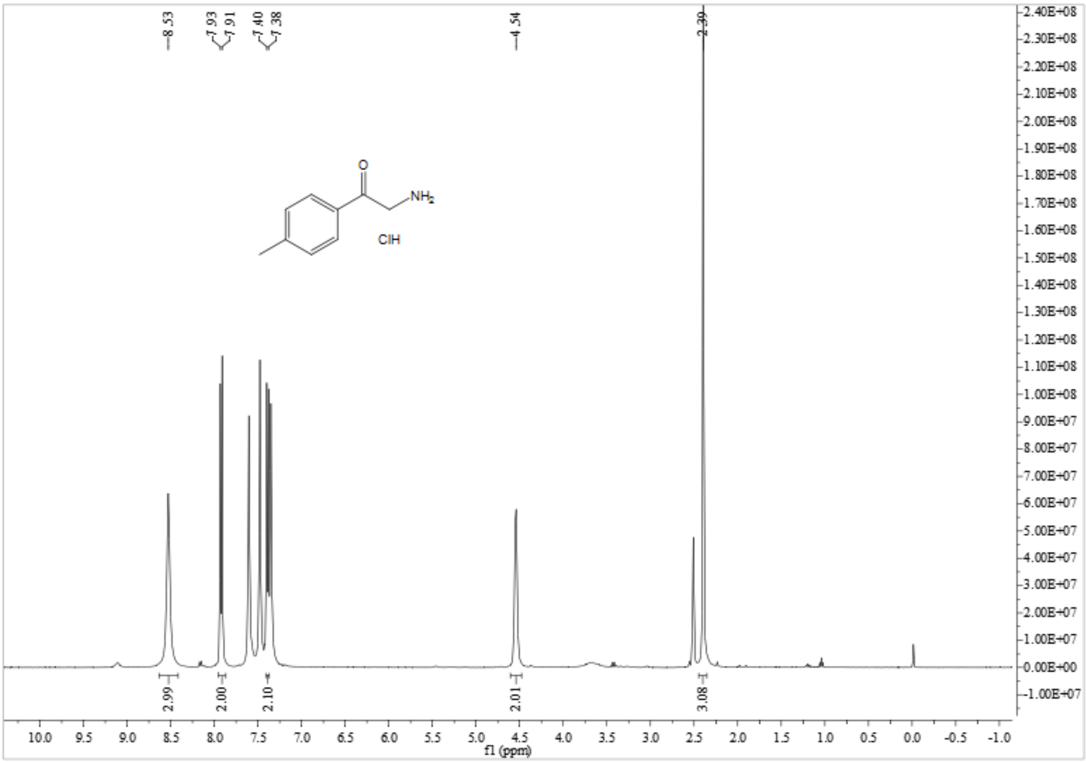

^{1}H-NMR spectrum of compound H5-002

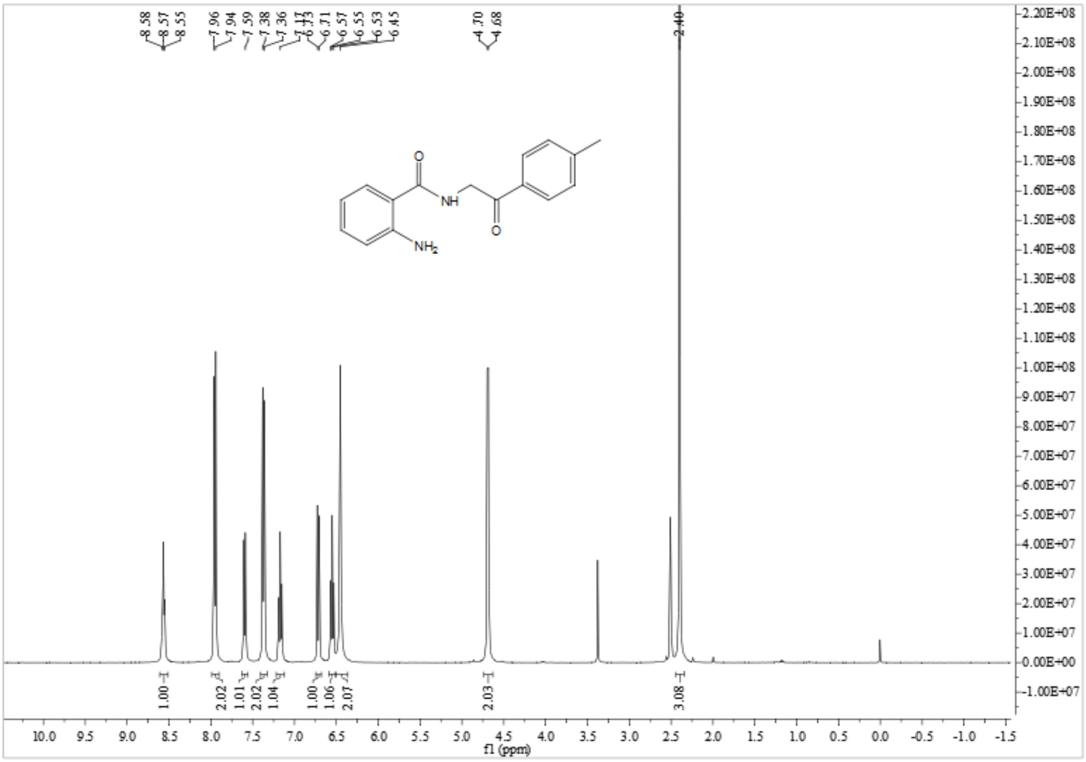

^{1}H-NMR spectrum of compound H5-003

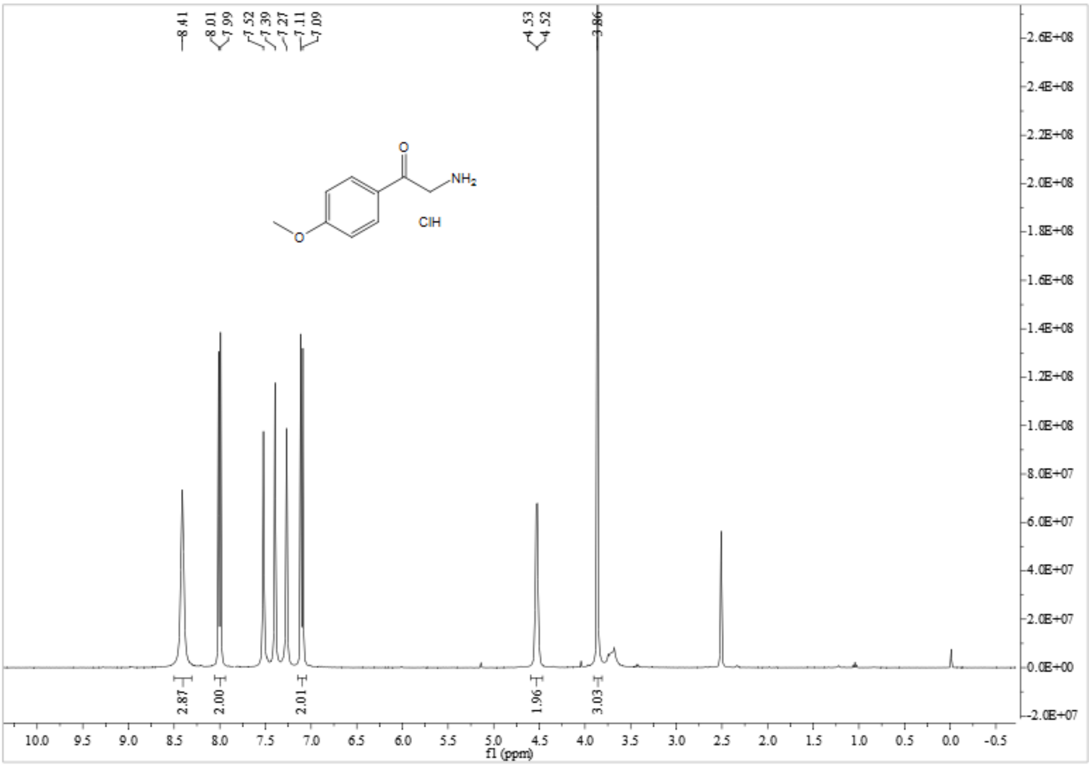

^{1}H-NMR spectrum of compound H6-002

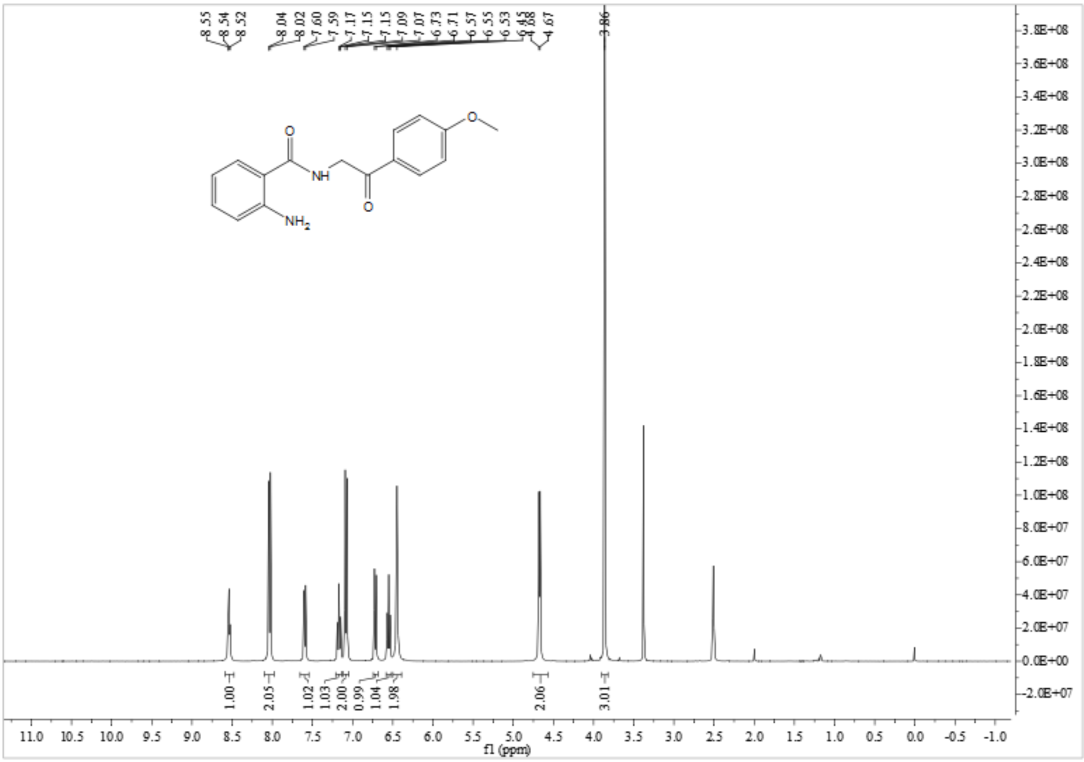

^{1}H-NMR spectrum of compound H6-003

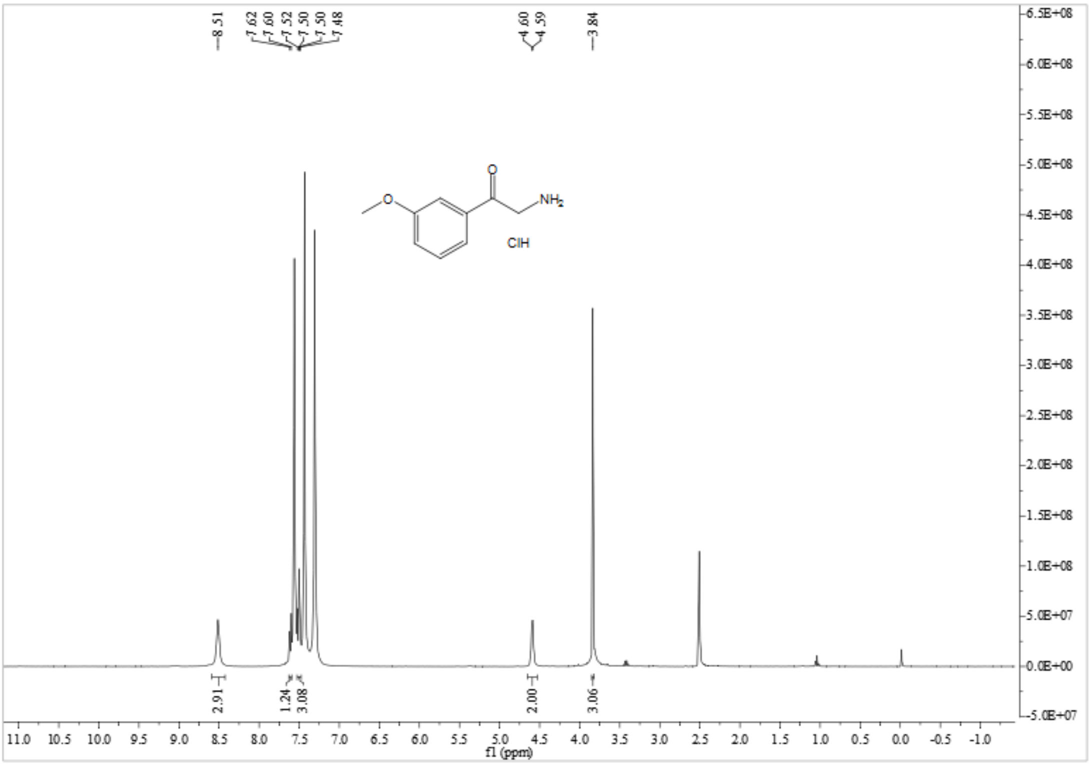

^{1}H-NMR spectrum of compound H7-002

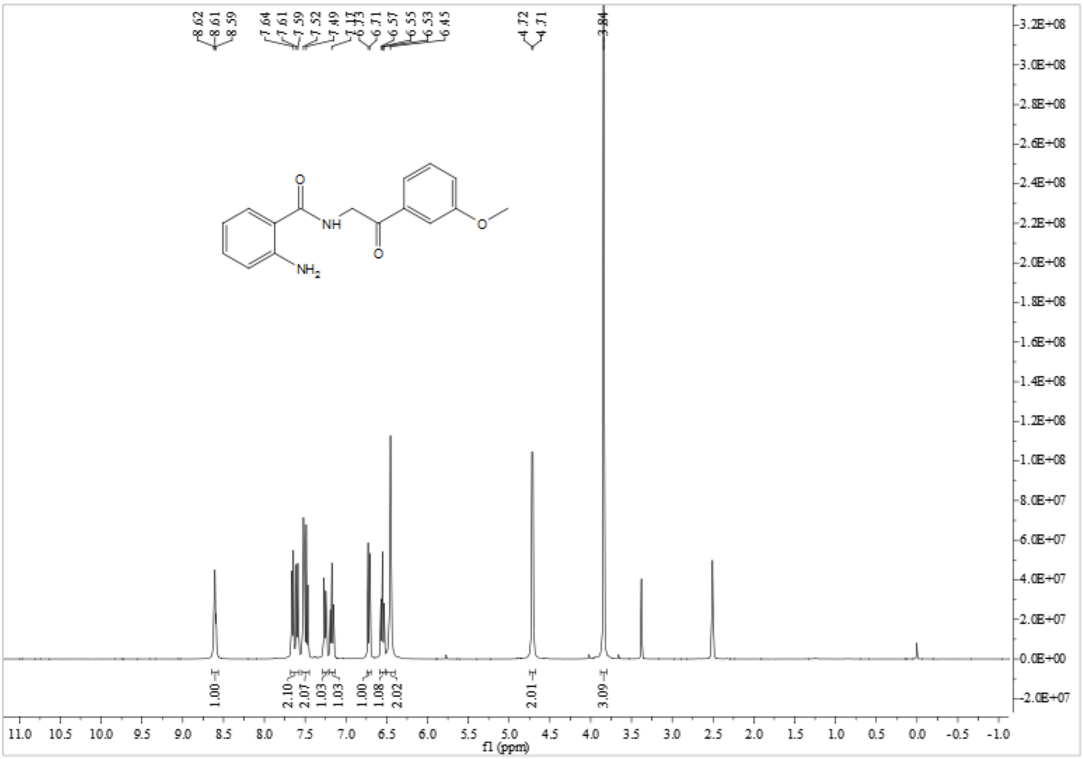

^{1}H-NMR spectrum of compound H7-003

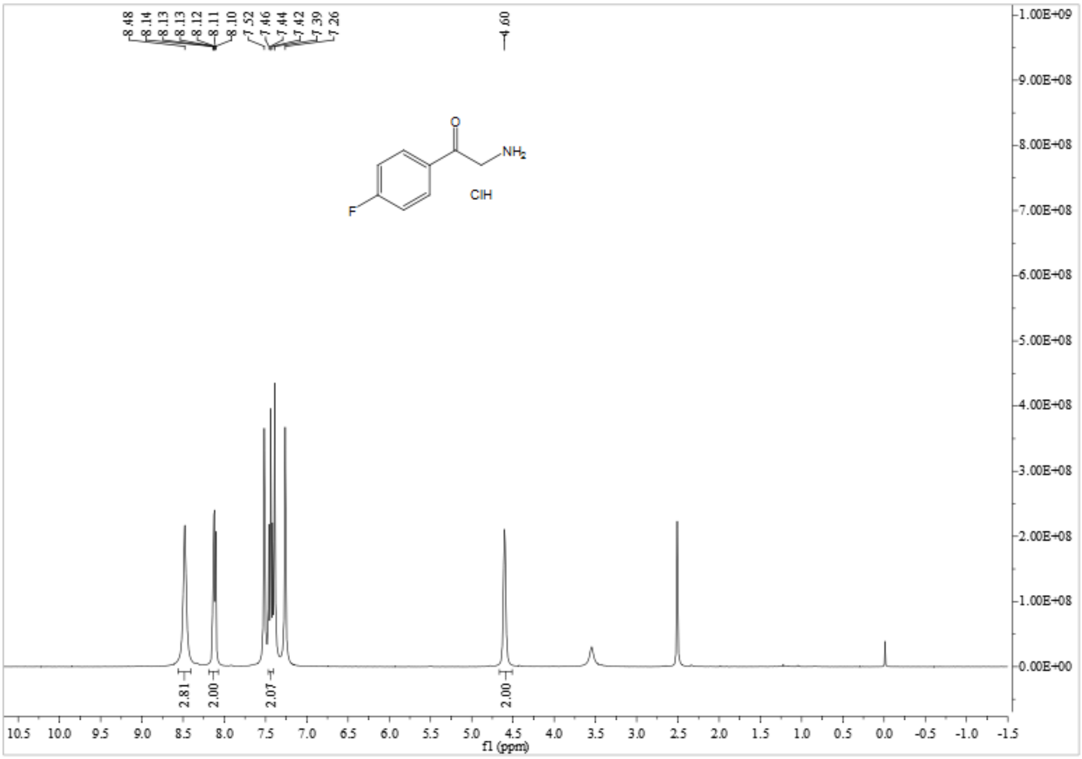

^{1}H-NMR spectrum of compound H8-002

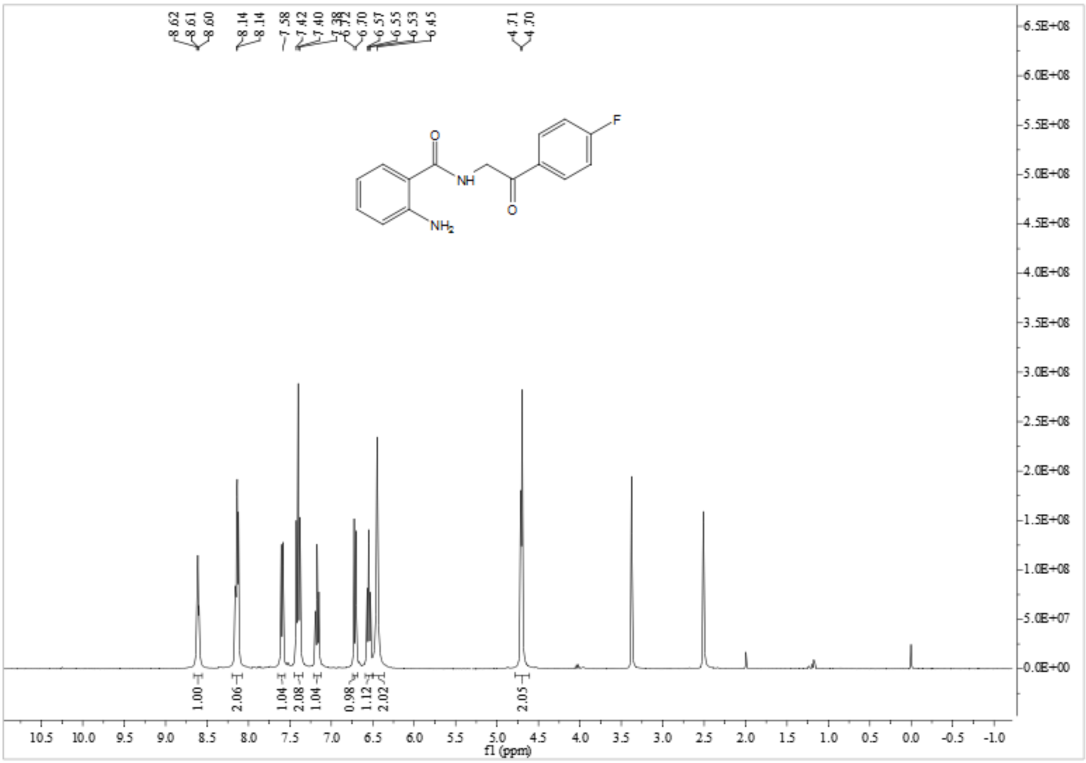

^{1}H-NMR spectrum of compound H8-003

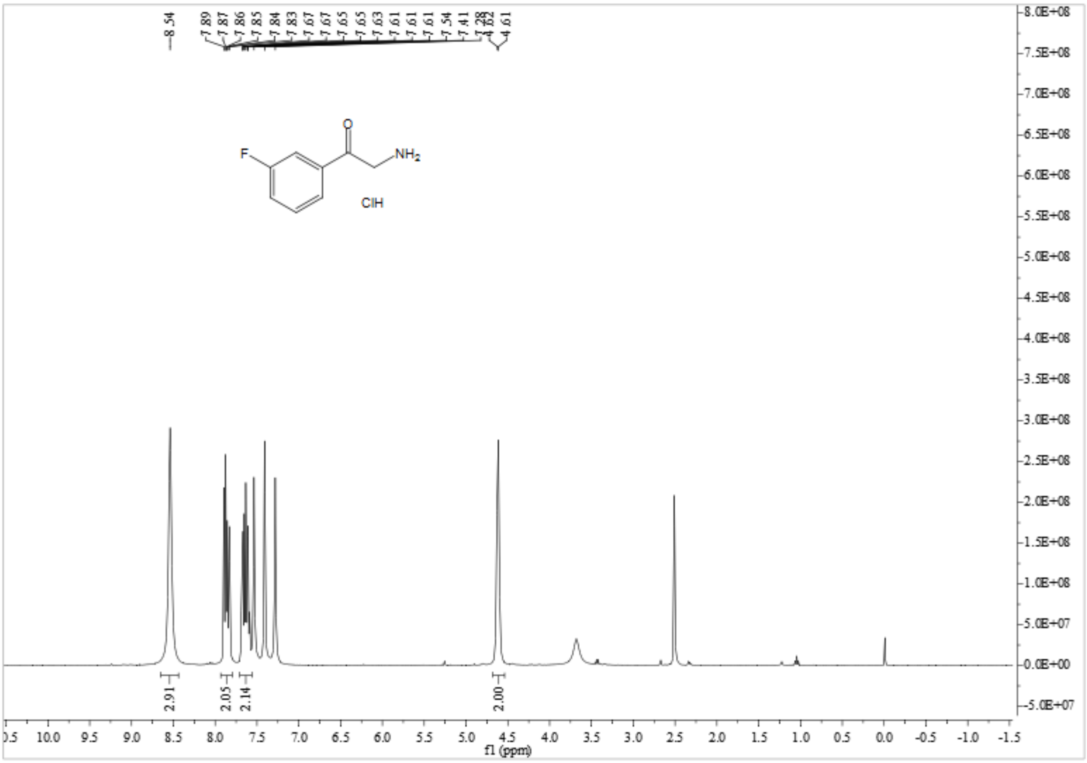

^{1}H-NMR spectrum of compound H9-002

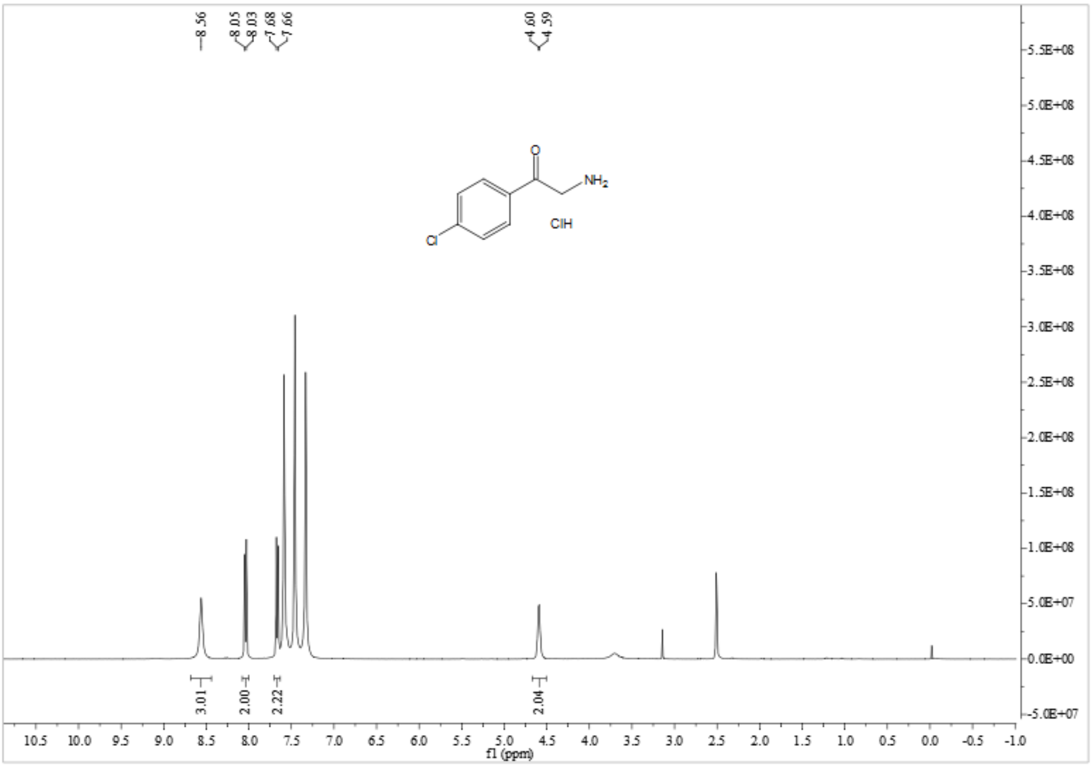

^{1}H-NMR spectrum of compound H10-002

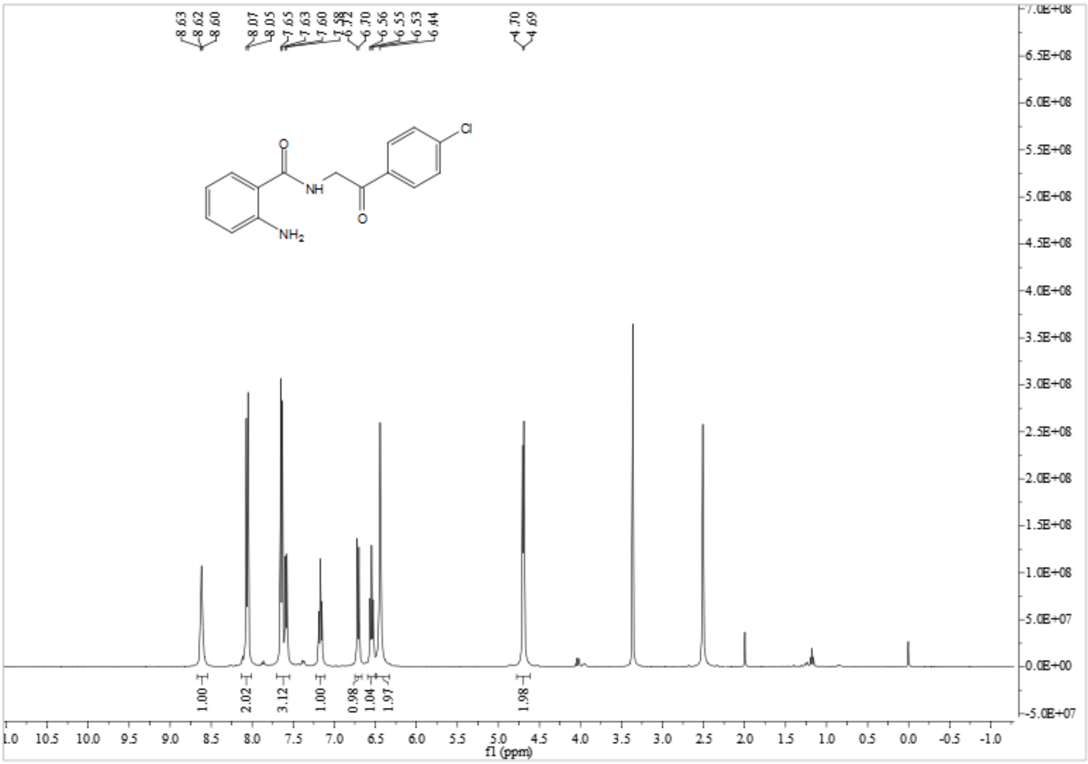

^{1}H-NMR spectrum of compound H10-003

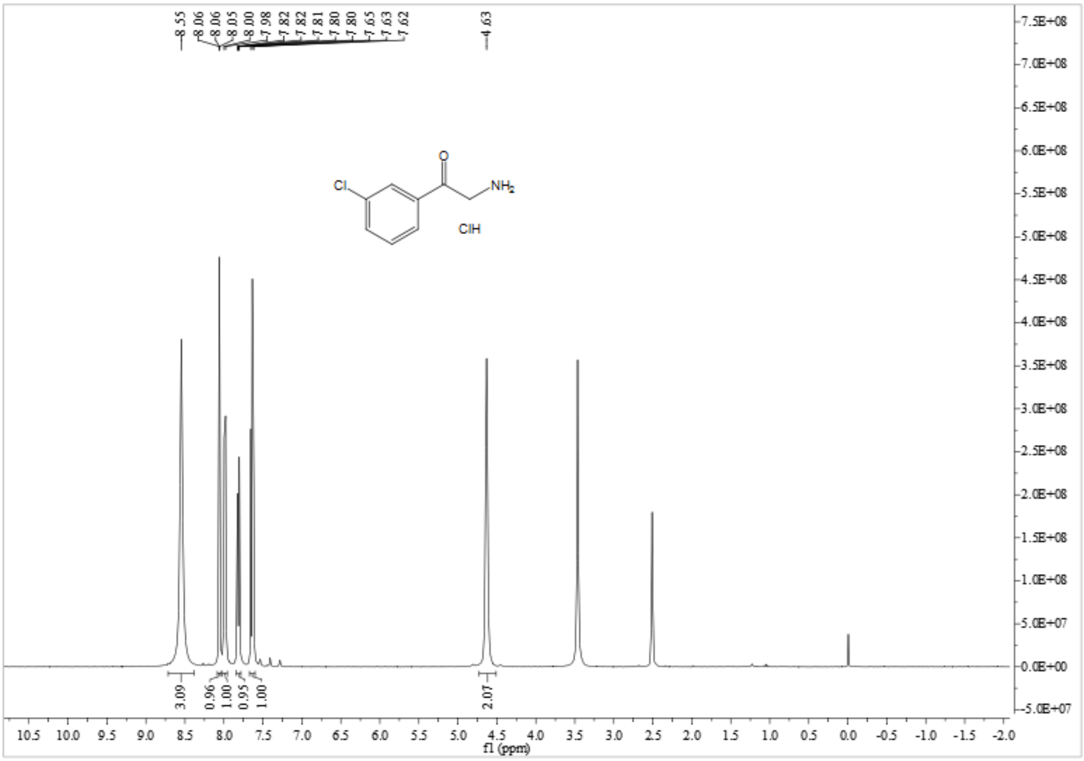

^{1}H-NMR spectrum of compound H11-002

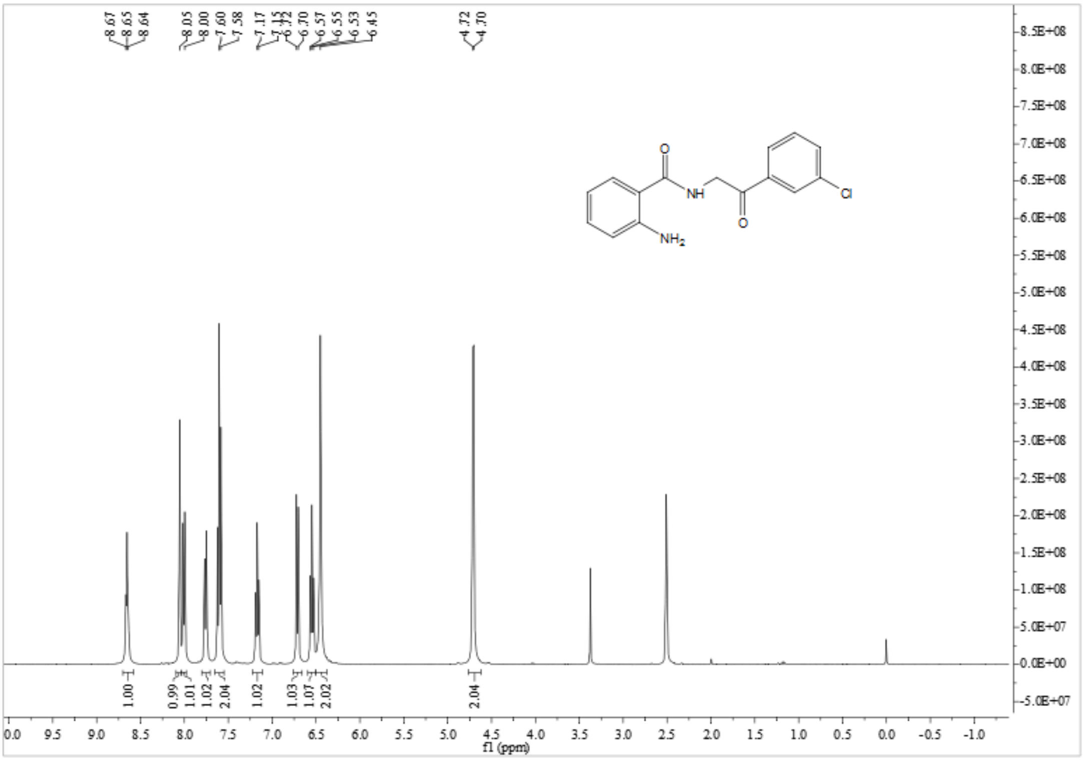

^{1}H-NMR spectrum of compound H11-003

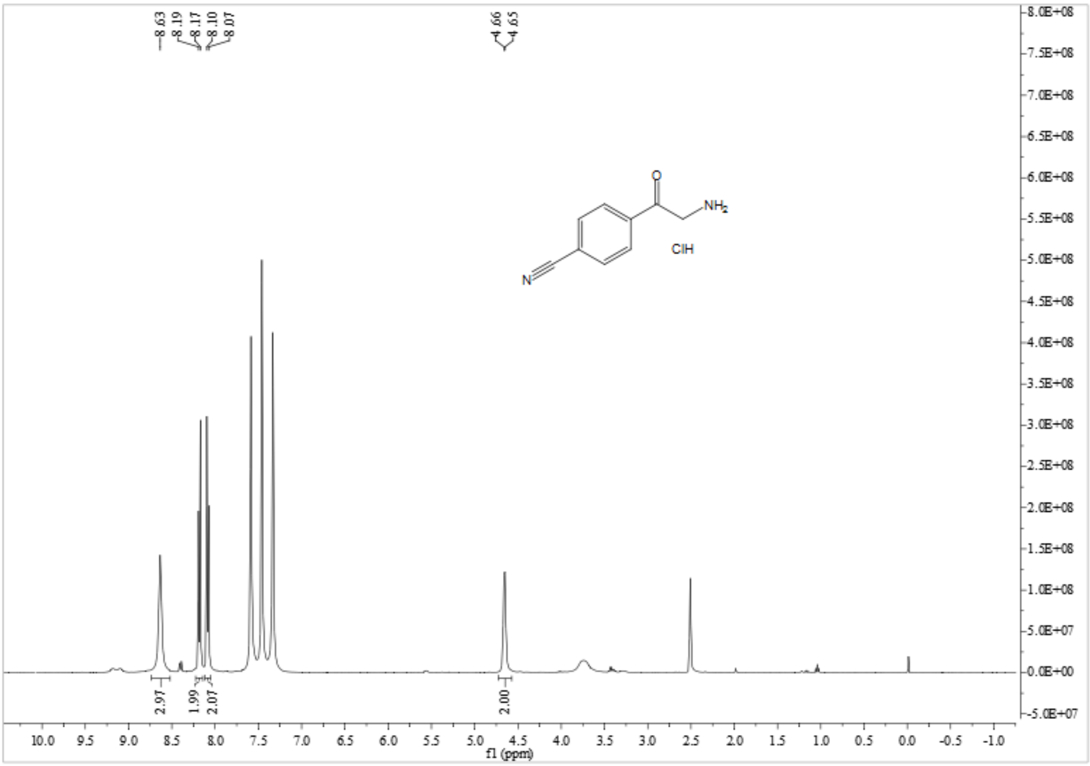

^{1}H-NMR spectrum of compound H12-002

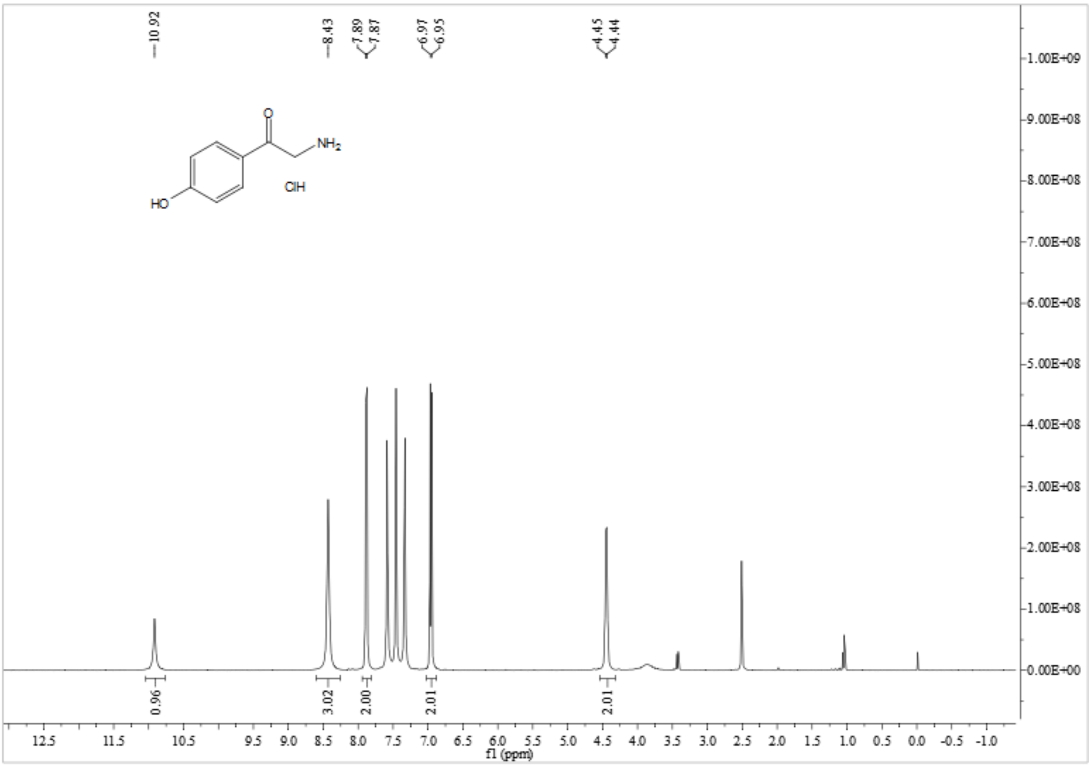

^{1}H-NMR spectrum of compound H13-002

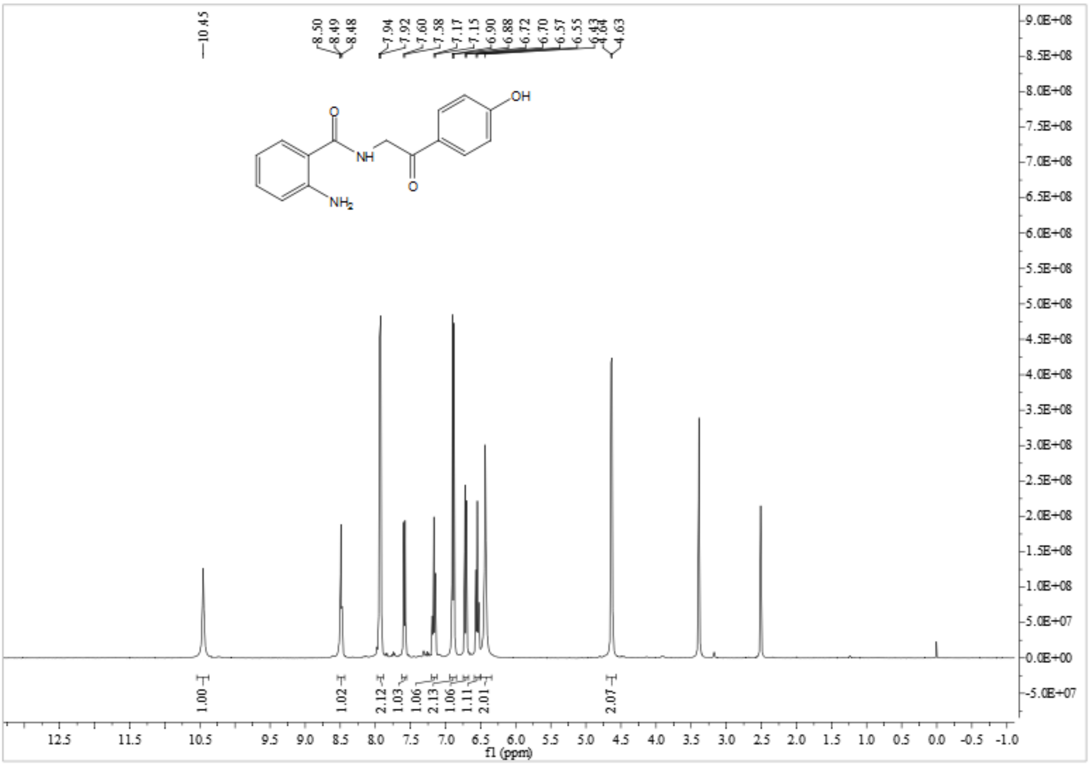

^{1}H-NMR spectrum of compound H13-003

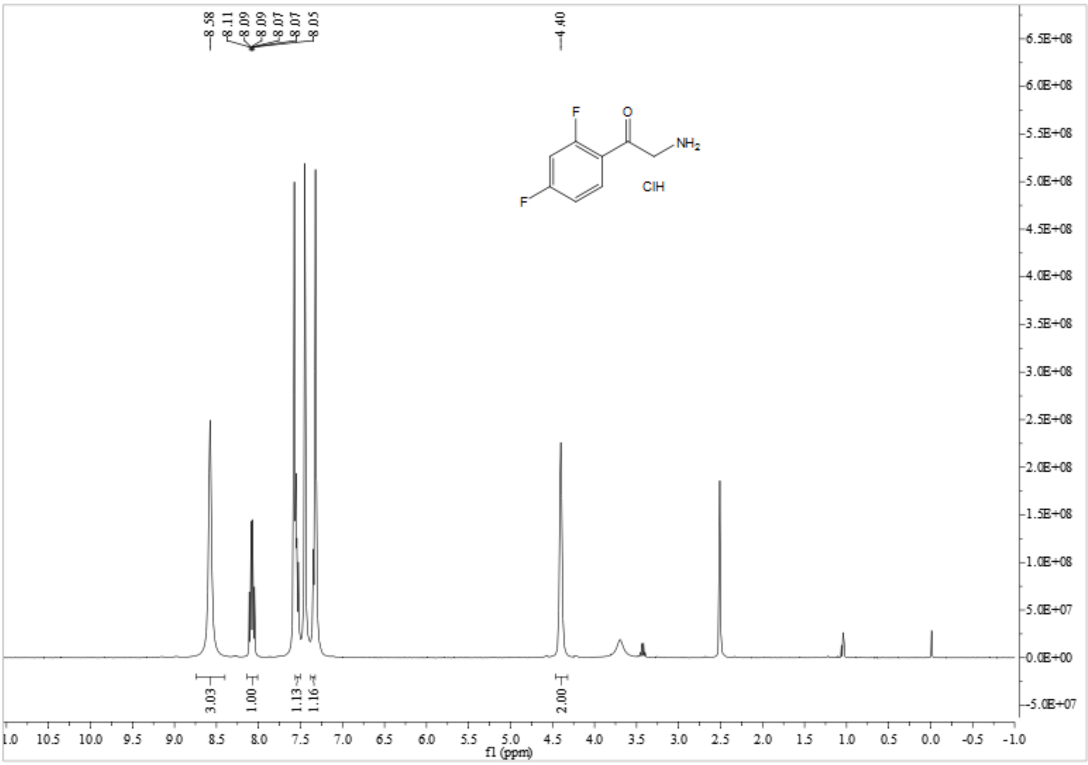

^{1}H-NMR spectrum of compound H14-002

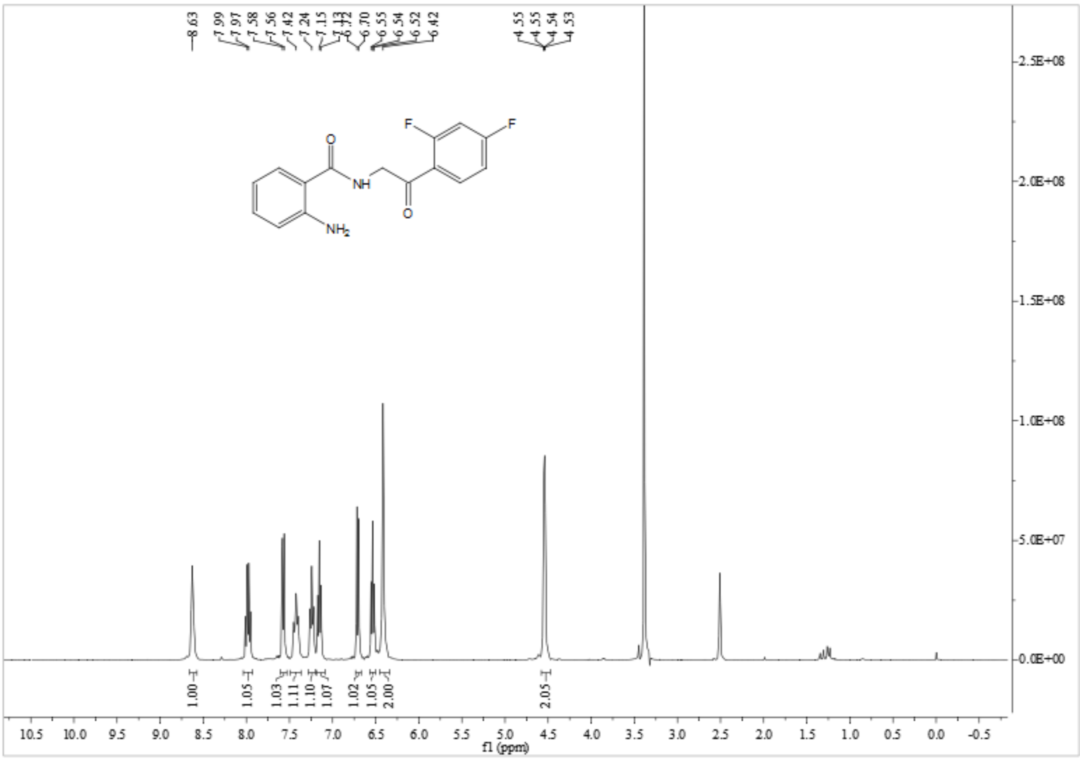

^{1}H-NMR spectrum of compound H14-003

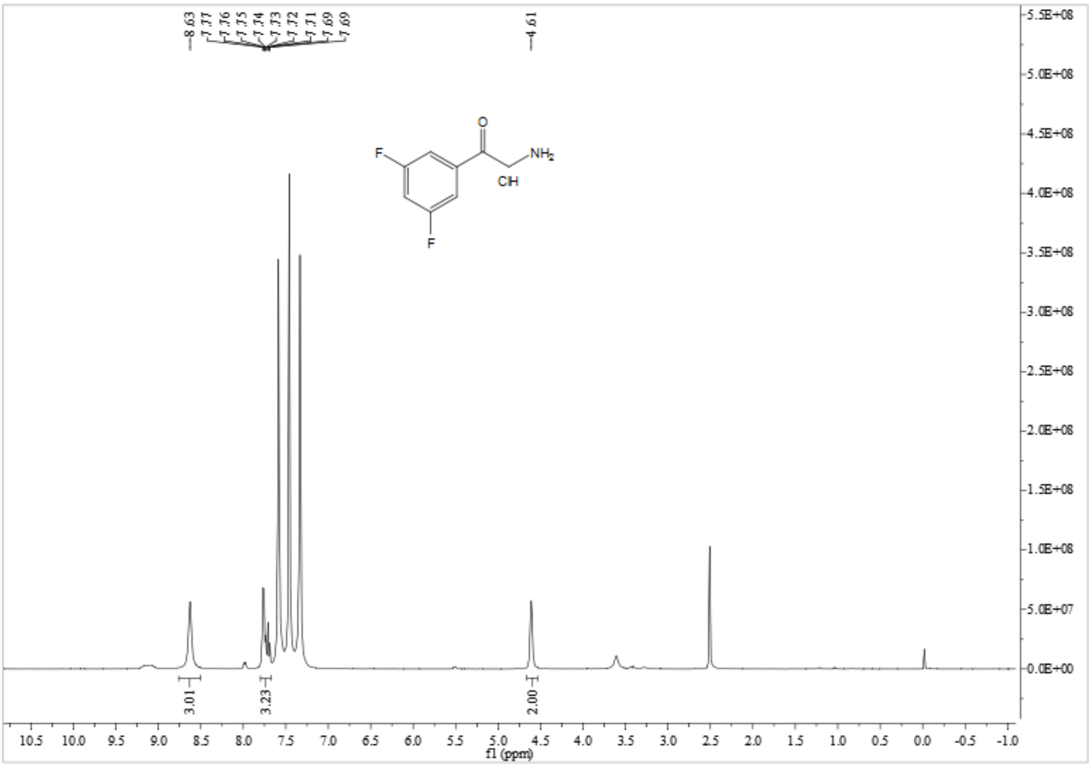

^{1}H-NMR spectrum of compound H15-002

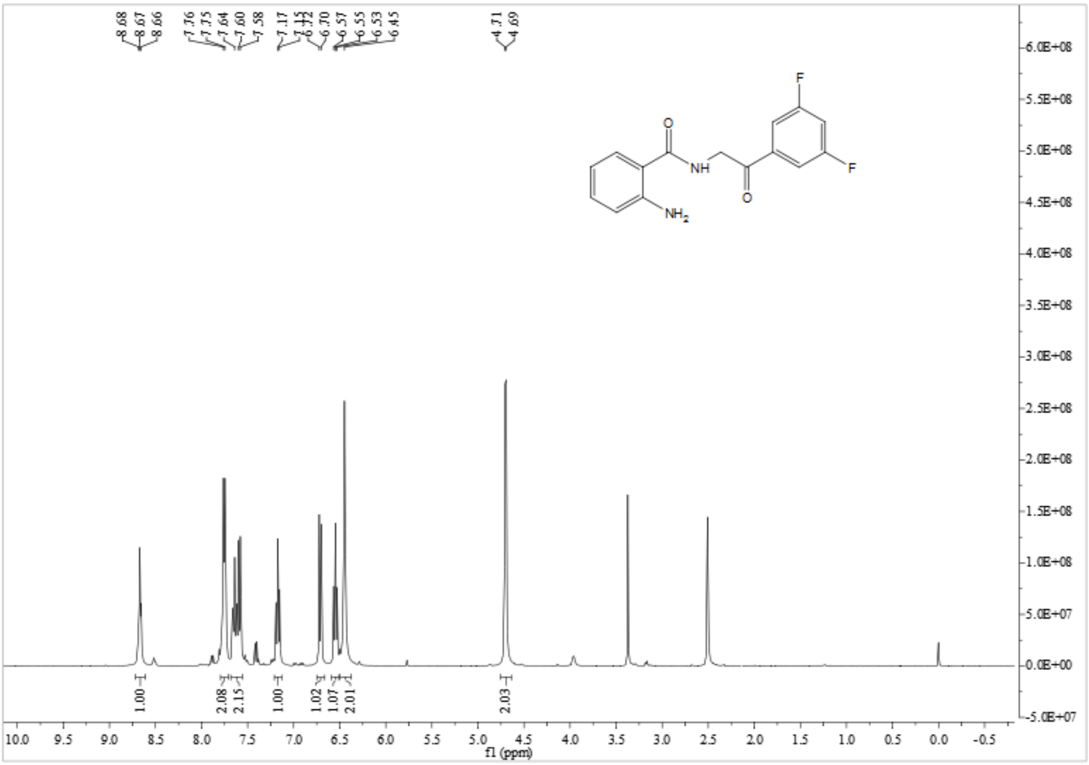

^{1}H-NMR spectrum of compound H15-003

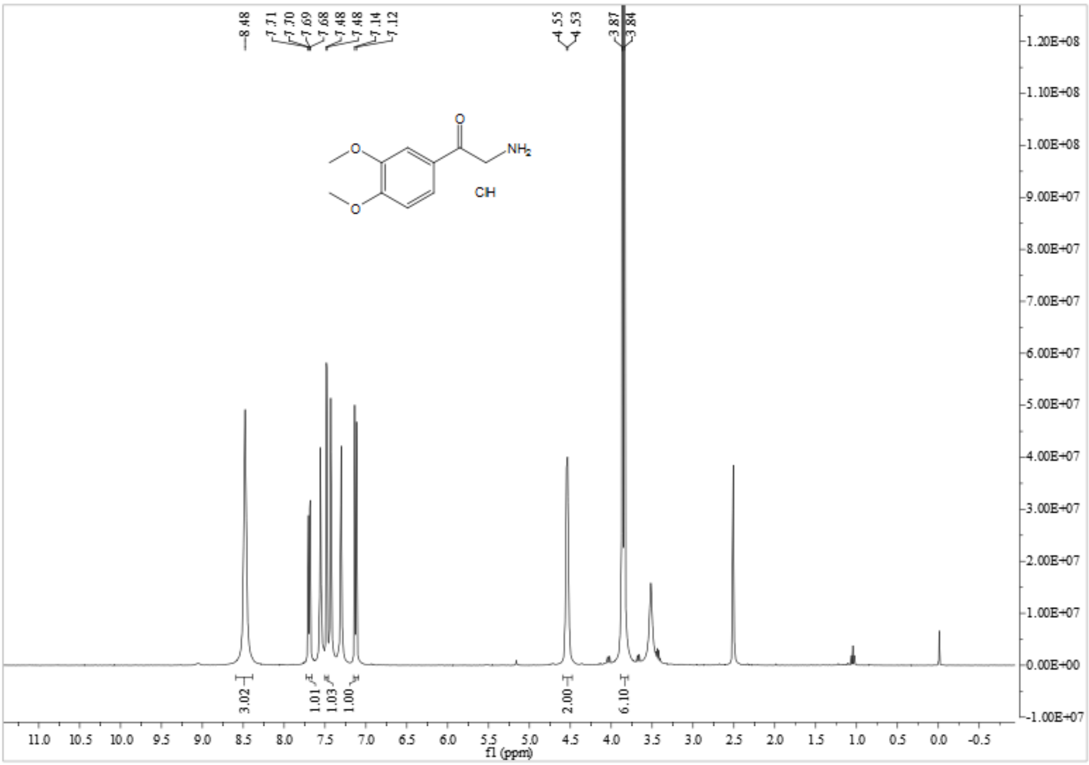

^{1}H-NMR spectrum of compound H16-002

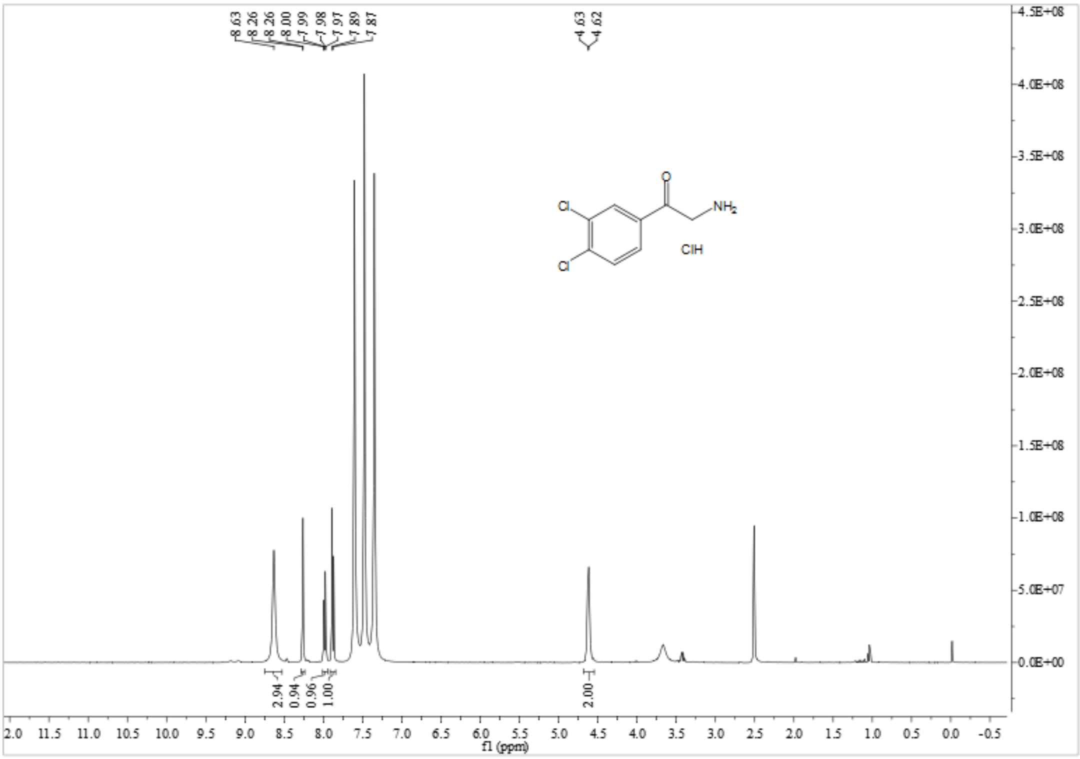

^{1}H-NMR spectrum of compound H17-002

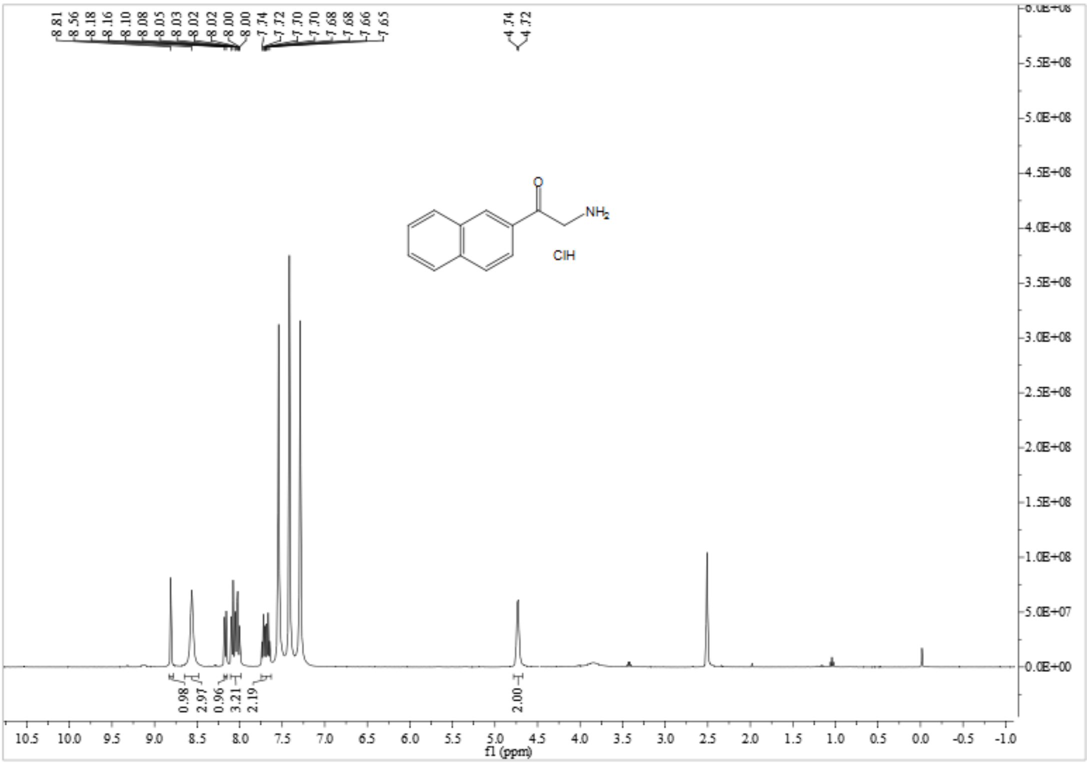

^{1}H-NMR spectrum of compound H18-002

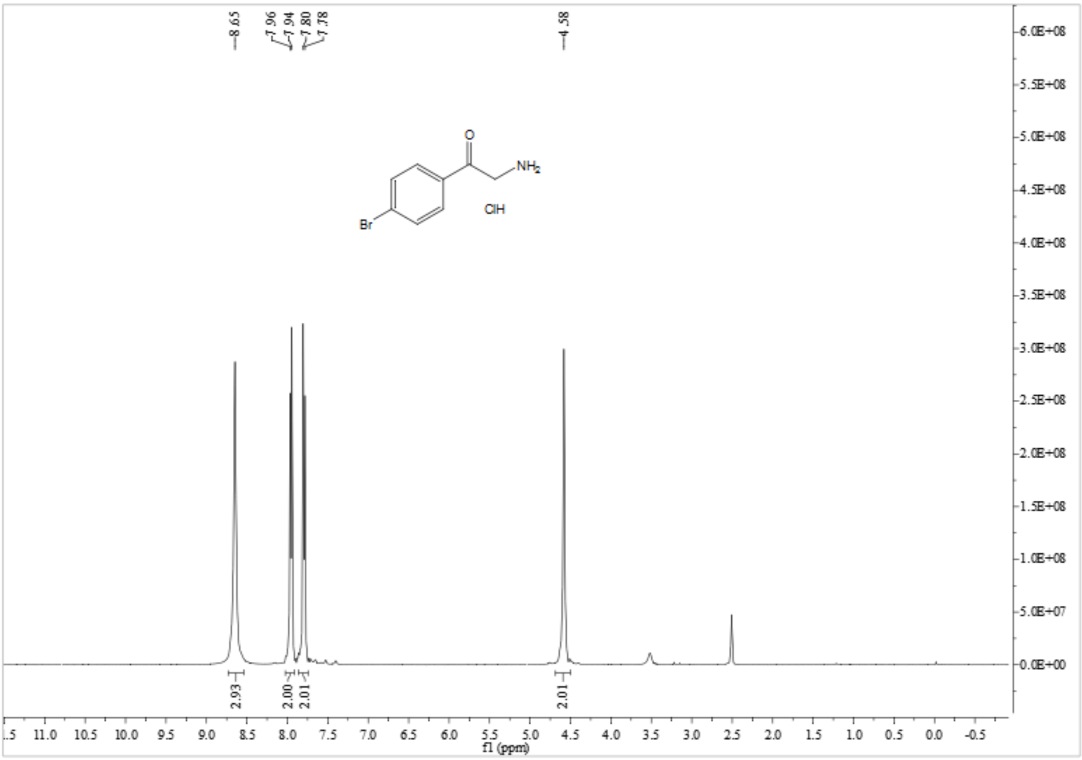

^{1}H-NMR spectrum of compound H19-002

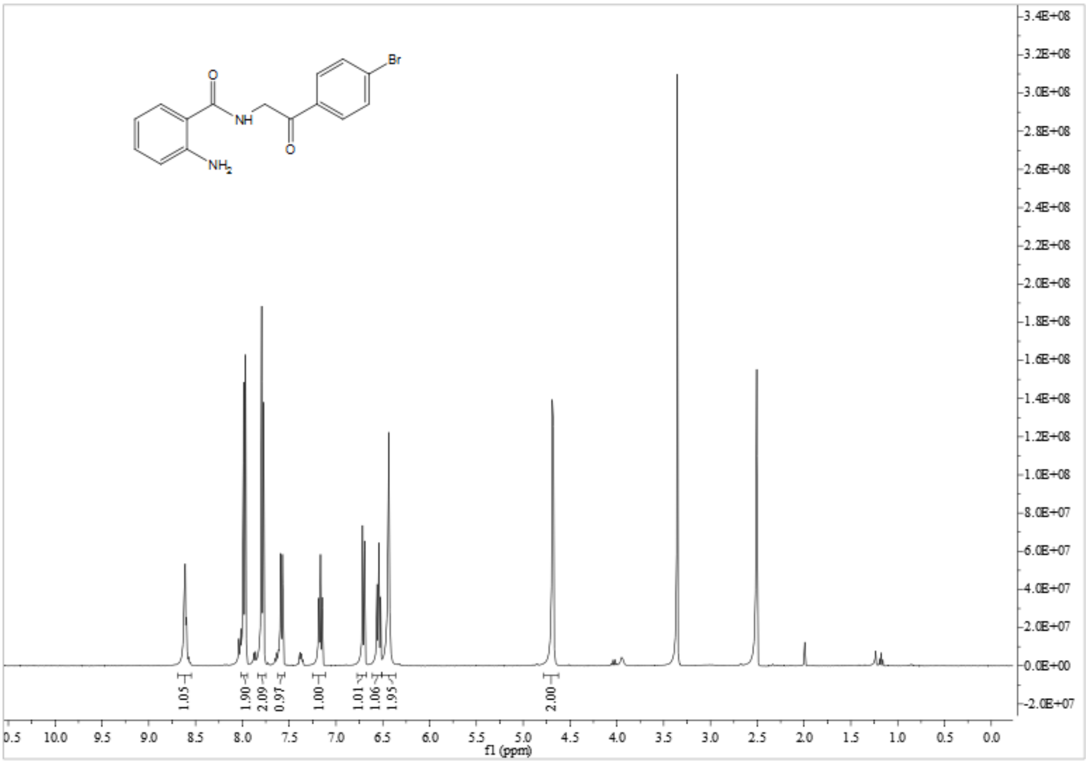

^{1}H-NMR spectrum of compound H19-003

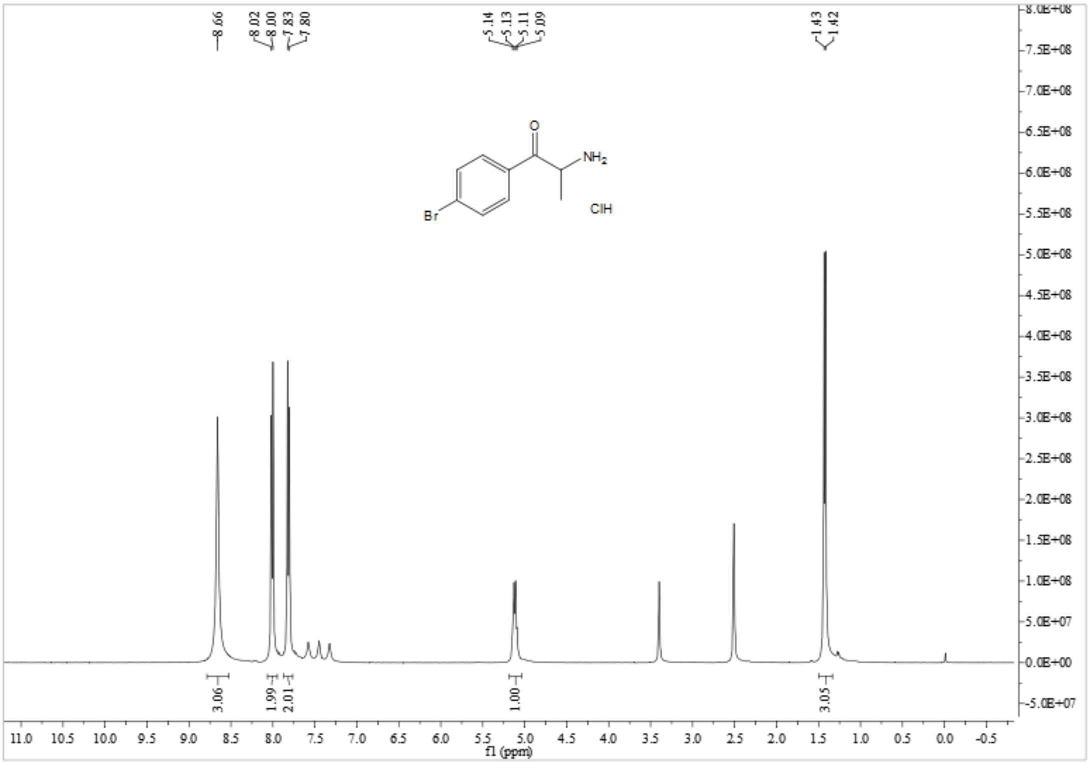

^{1}H-NMR spectrum of compound H30-002

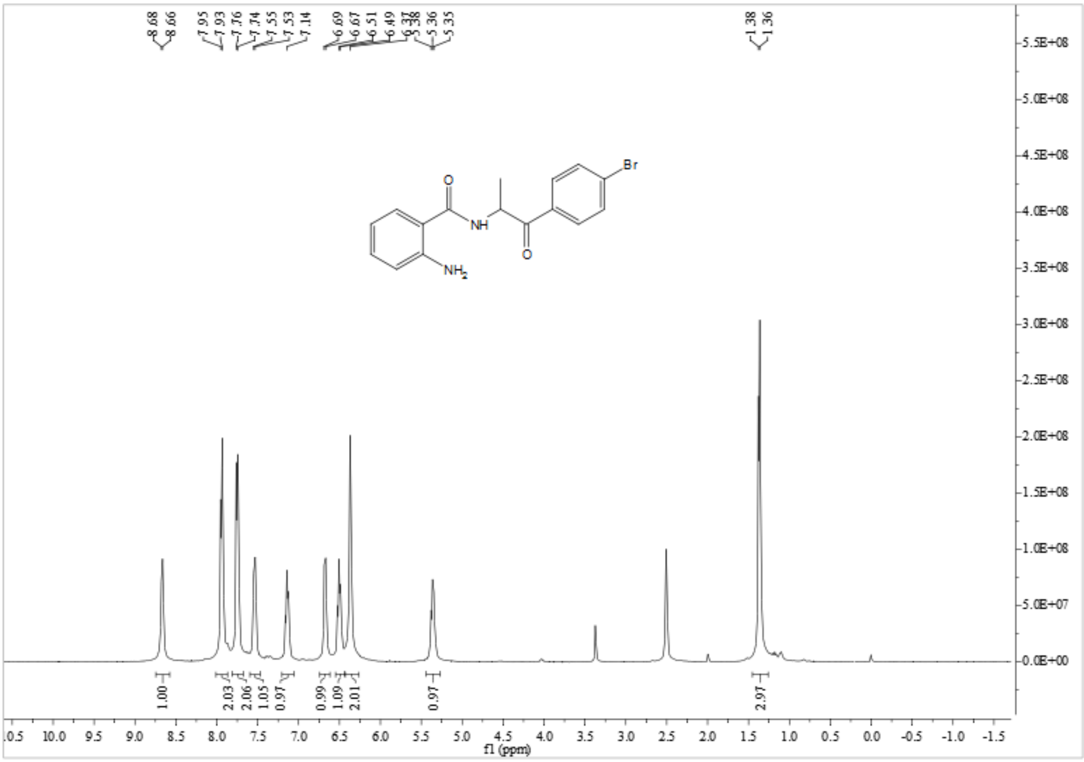

^{1}H-NMR spectrum of compound H30-003

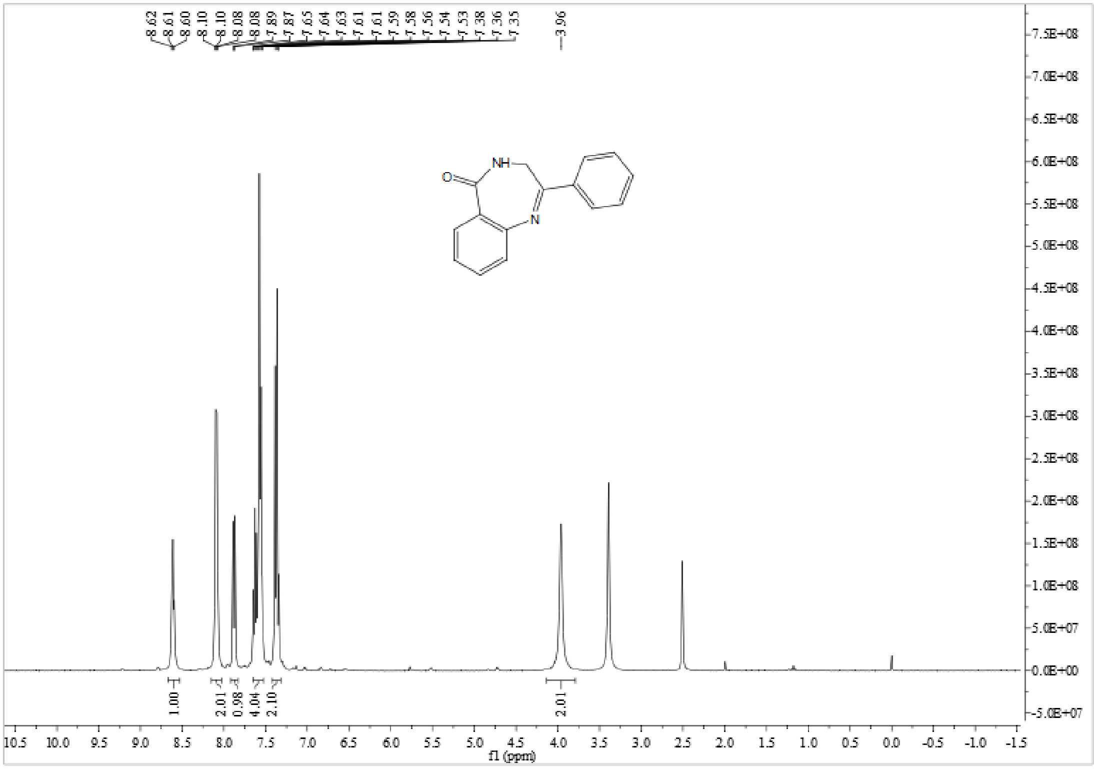

^{1}H-NMR spectrum of compound H4

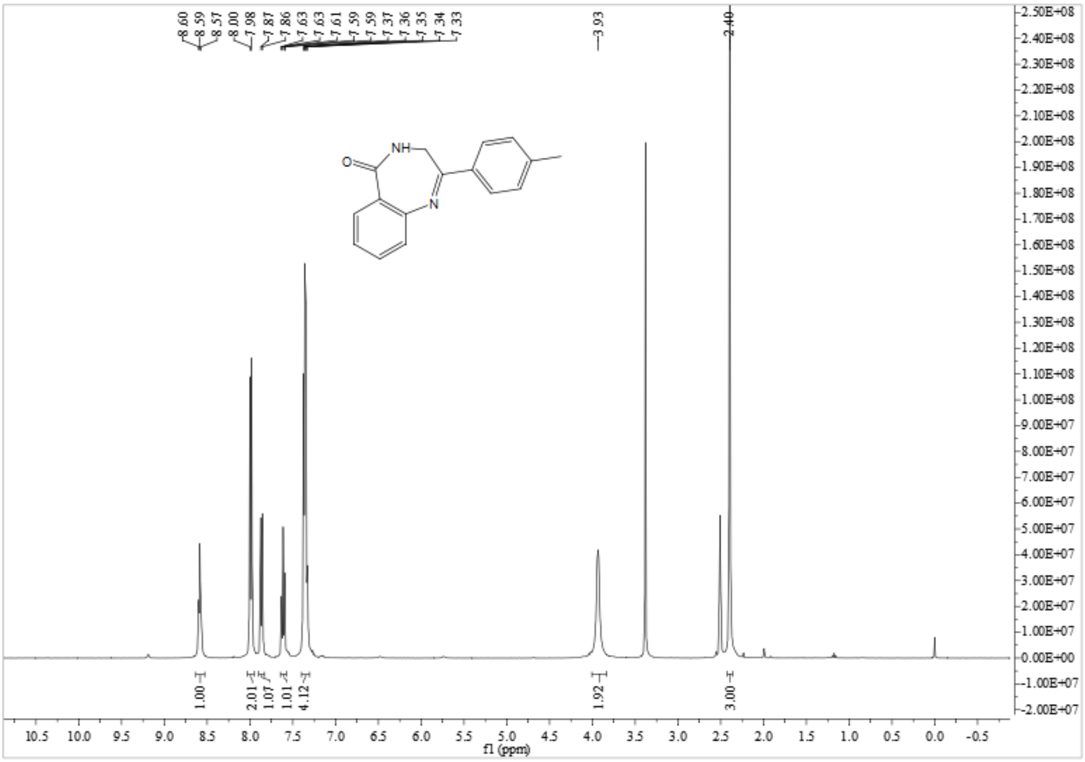

^{1}H-NMR spectrum of compound H5

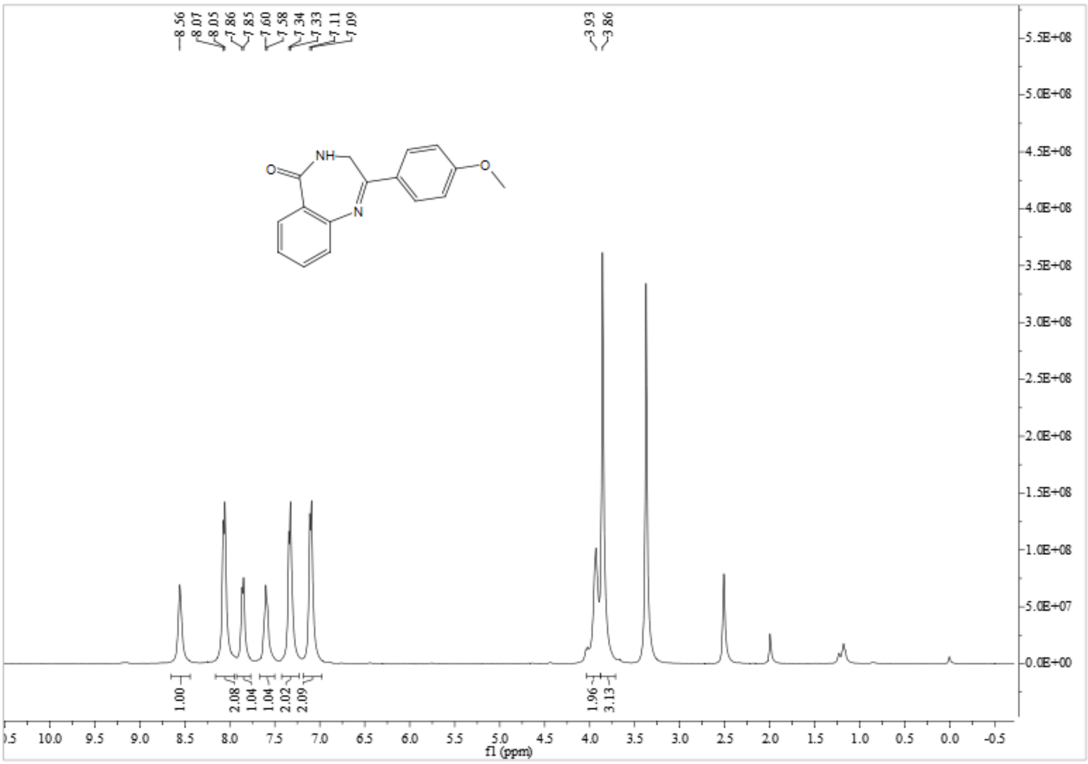

^{1}H-NMR spectrum of compound H6

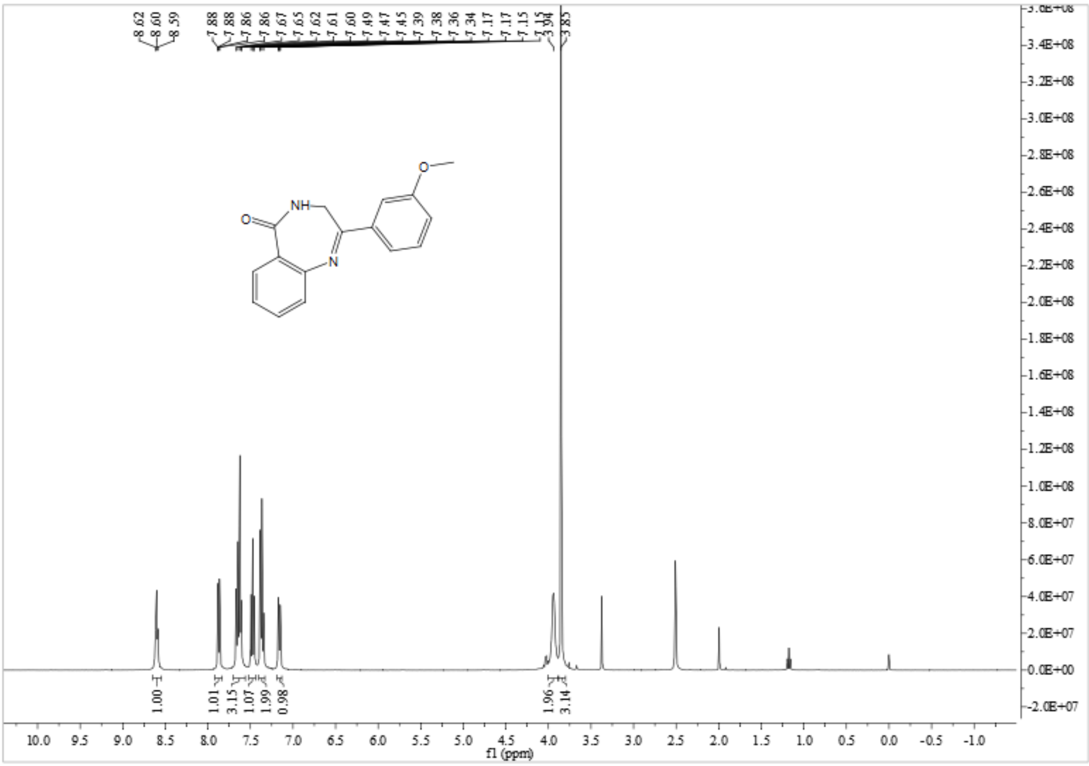

^{1}H-NMR spectrum of compound H7

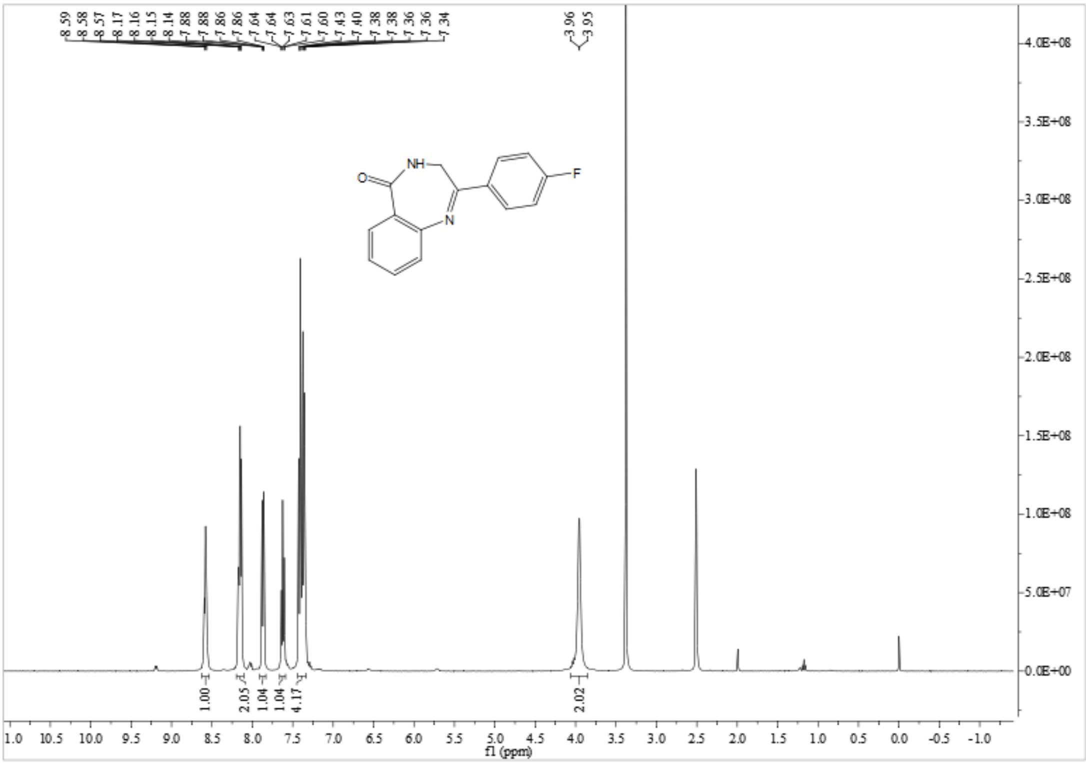

^{1}H-NMR spectrum of compound H8

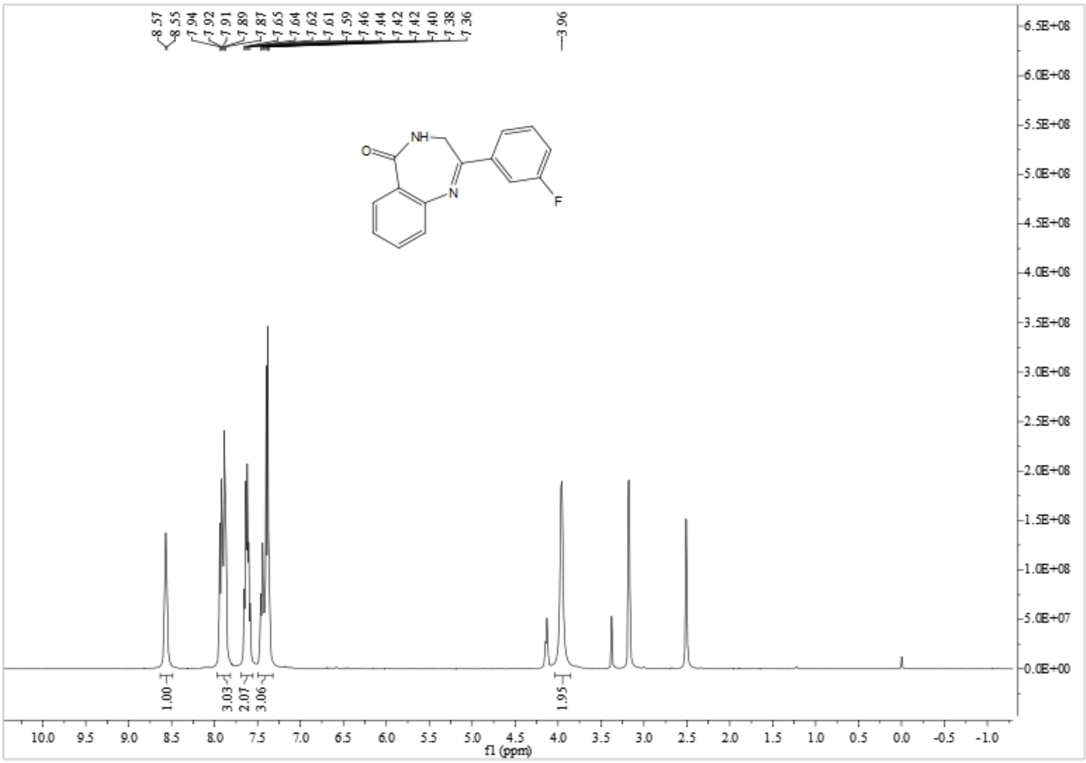

^{1}H-NMR spectrum of compound H9

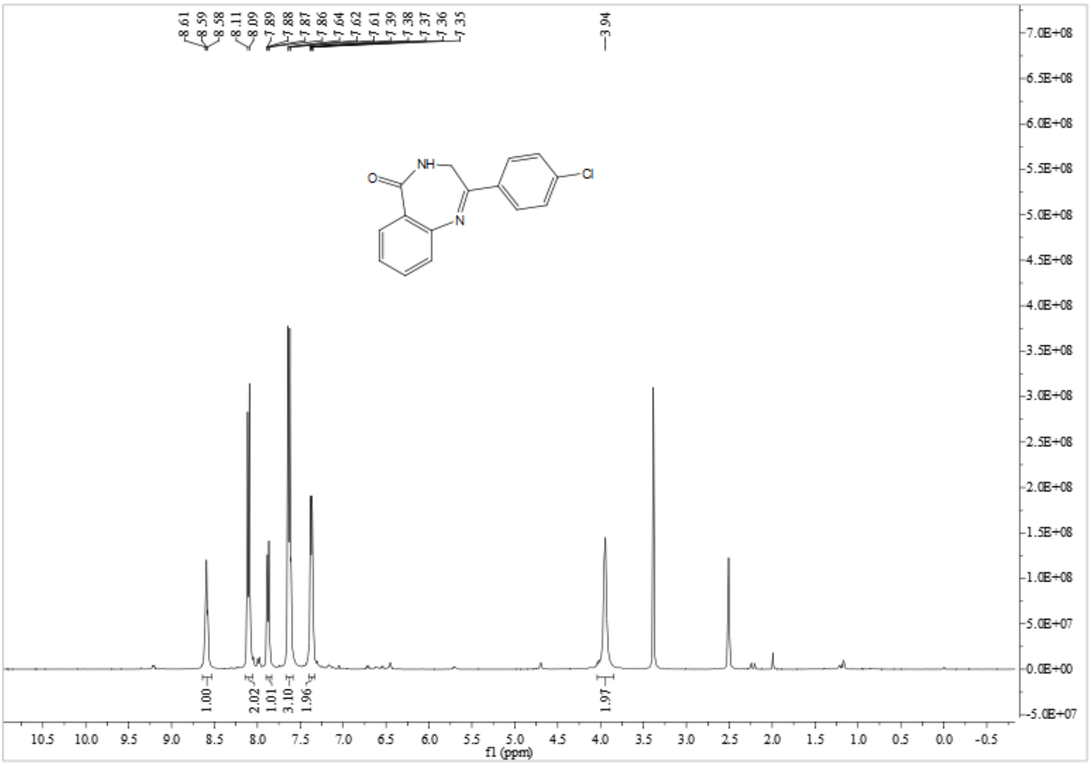

^{1}H-NMR spectrum of compound H10

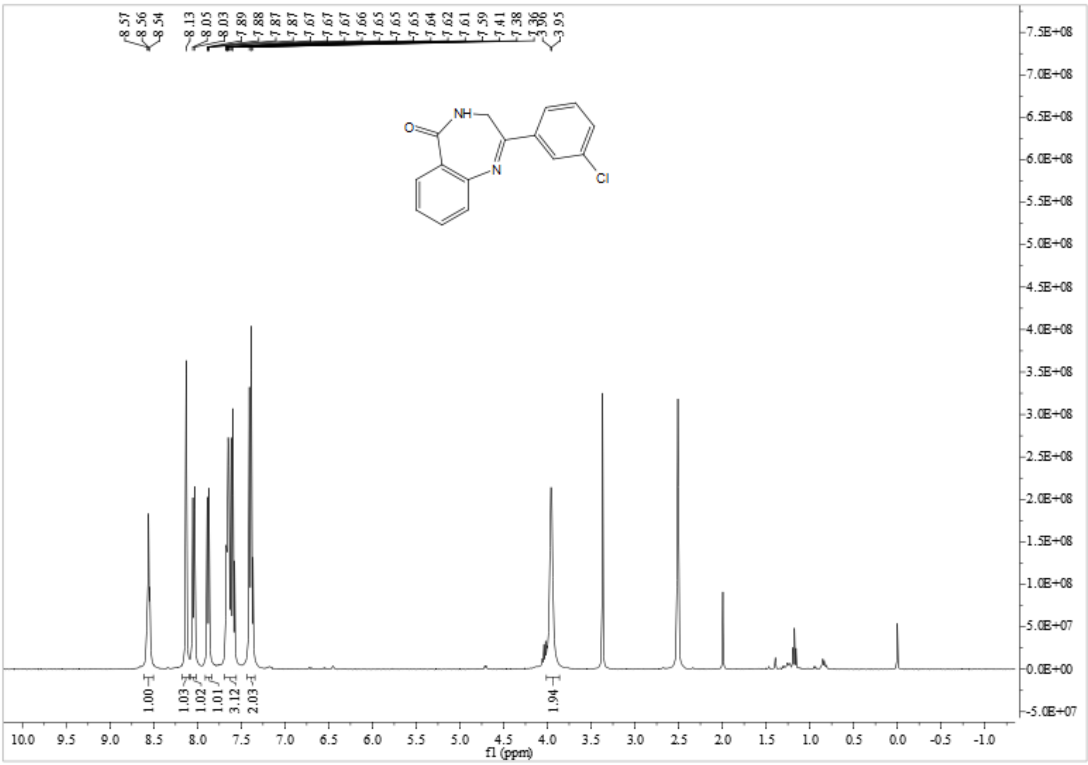

^{1}H-NMR spectrum of compound H11

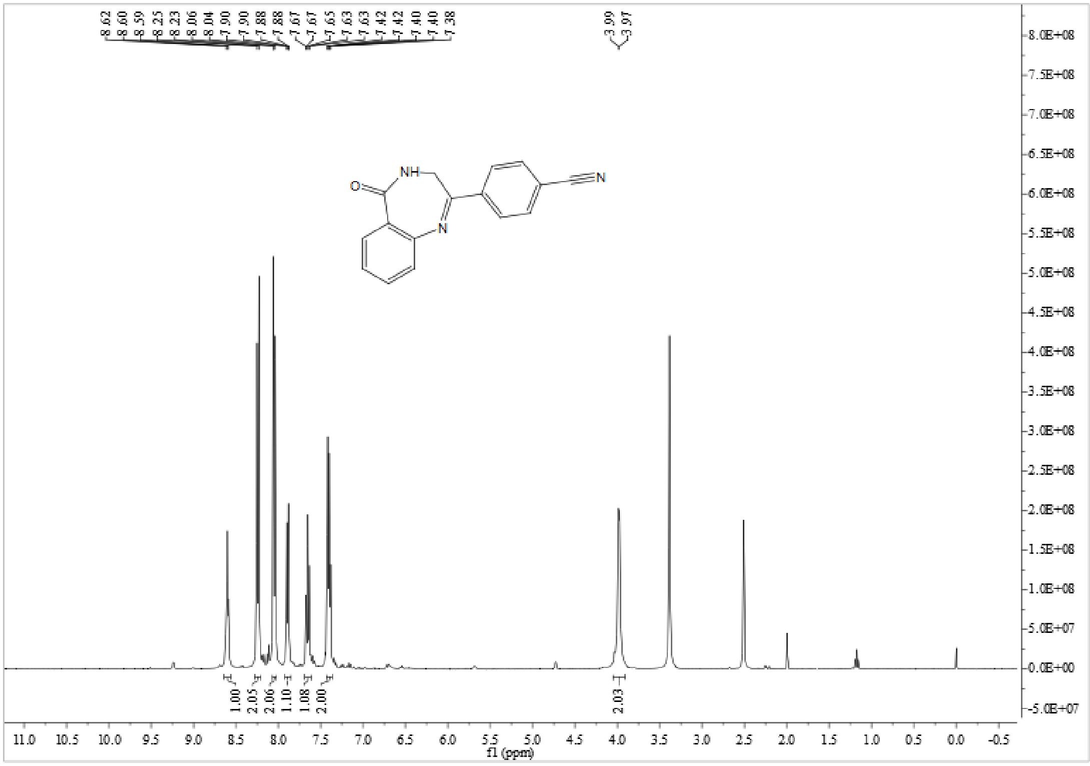

^{1}H-NMR spectrum of compound H12

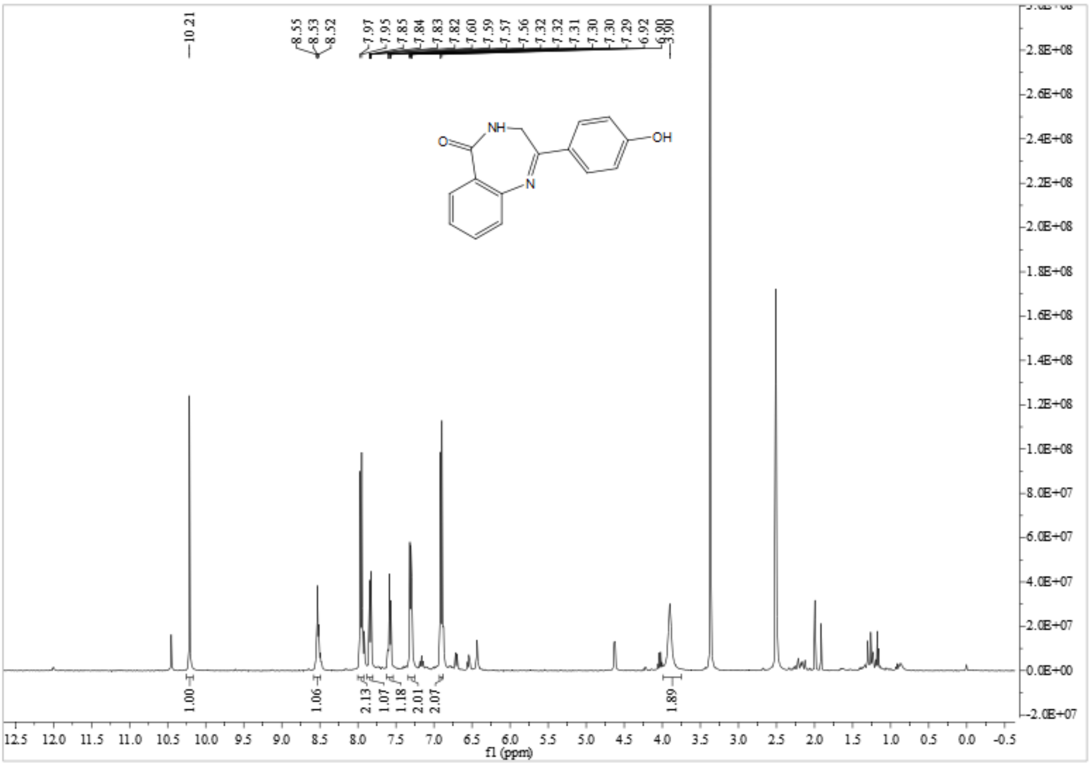

^{1}H-NMR spectrum of compound H13

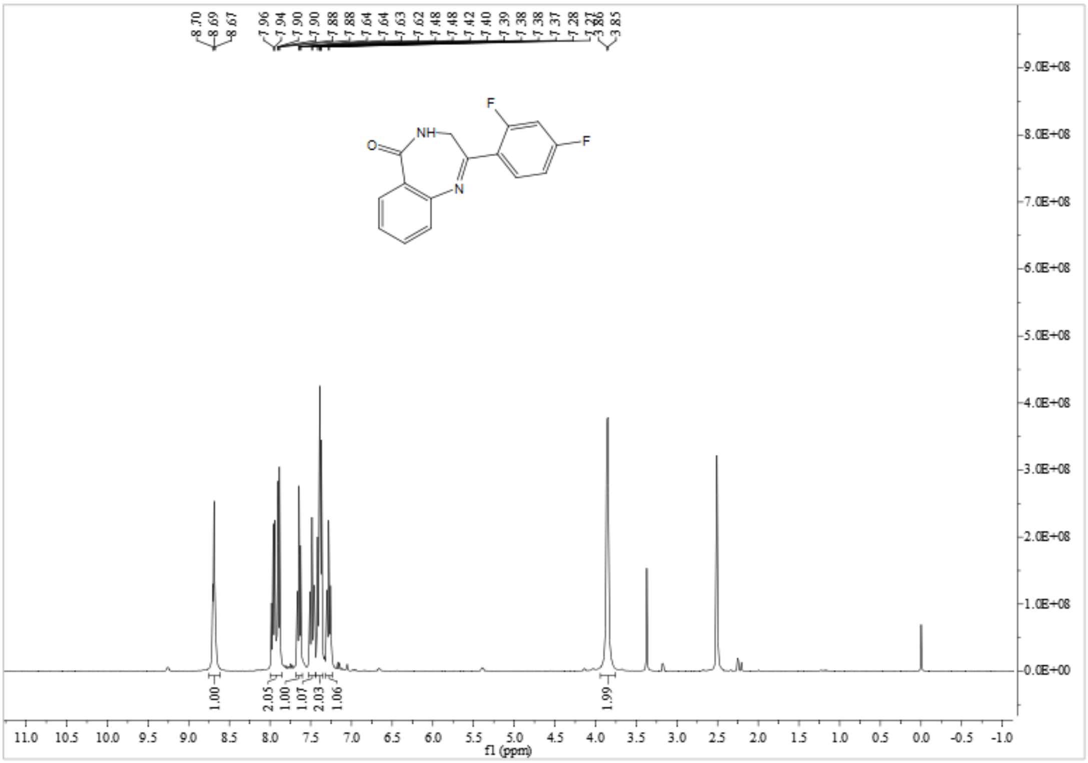

^{1}H-NMR spectrum of compound H14

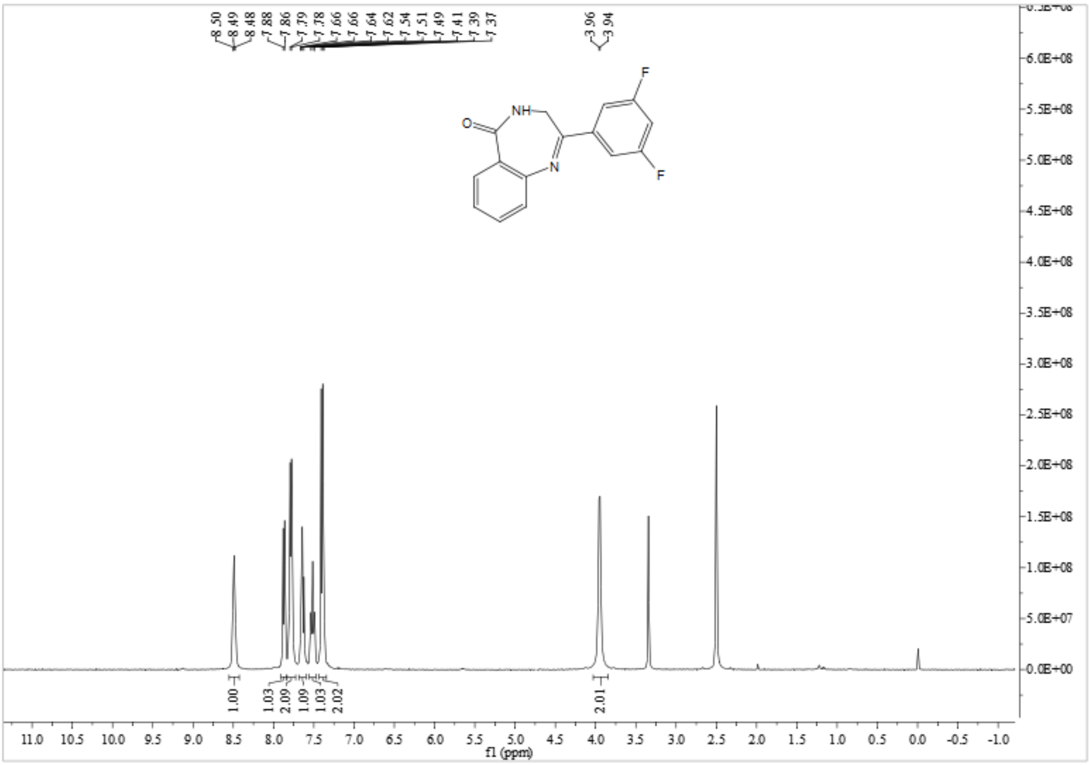

^{1}H-NMR spectrum of compound H15

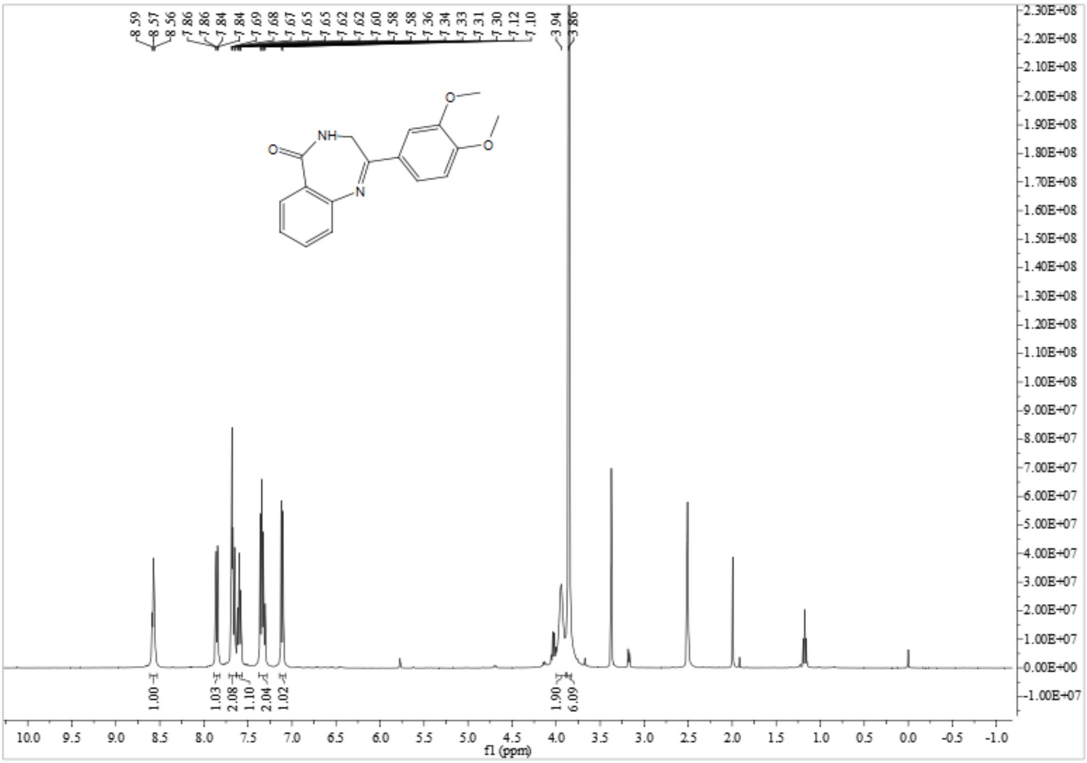

^{1}H-NMR spectrum of compound H16

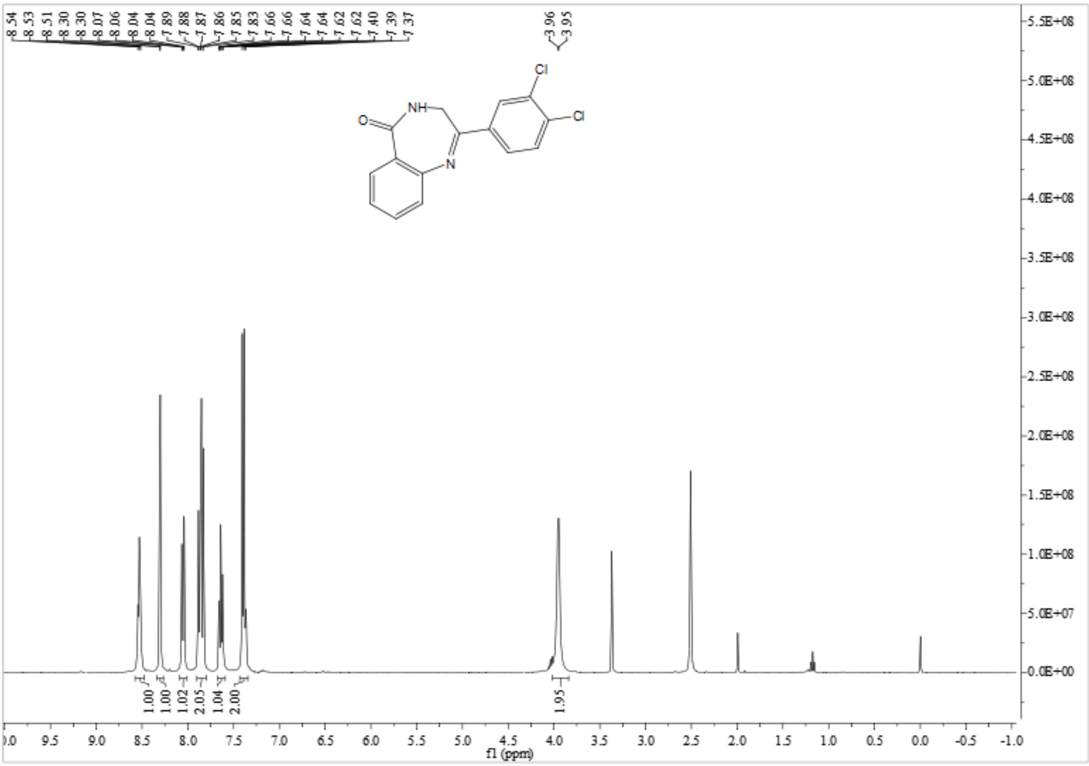

^{1}H-NMR spectrum of compound H17

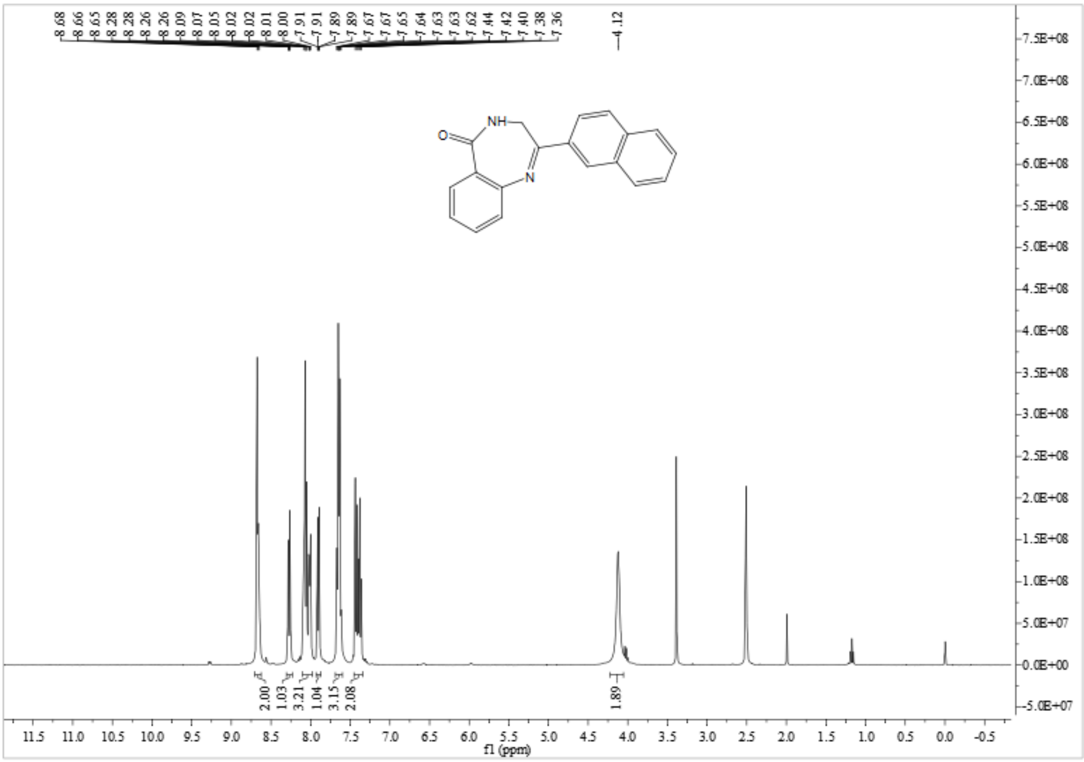

^{1}H-NMR spectrum of compound H18

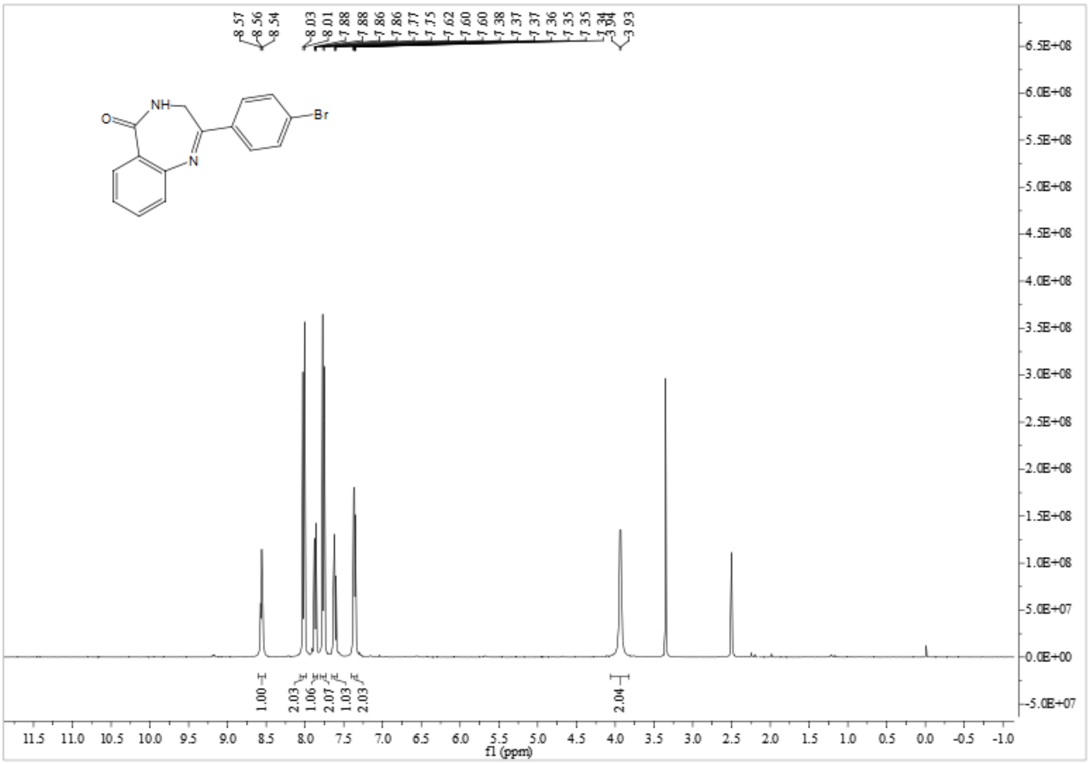

^{1}H-NMR spectrum of compound H19

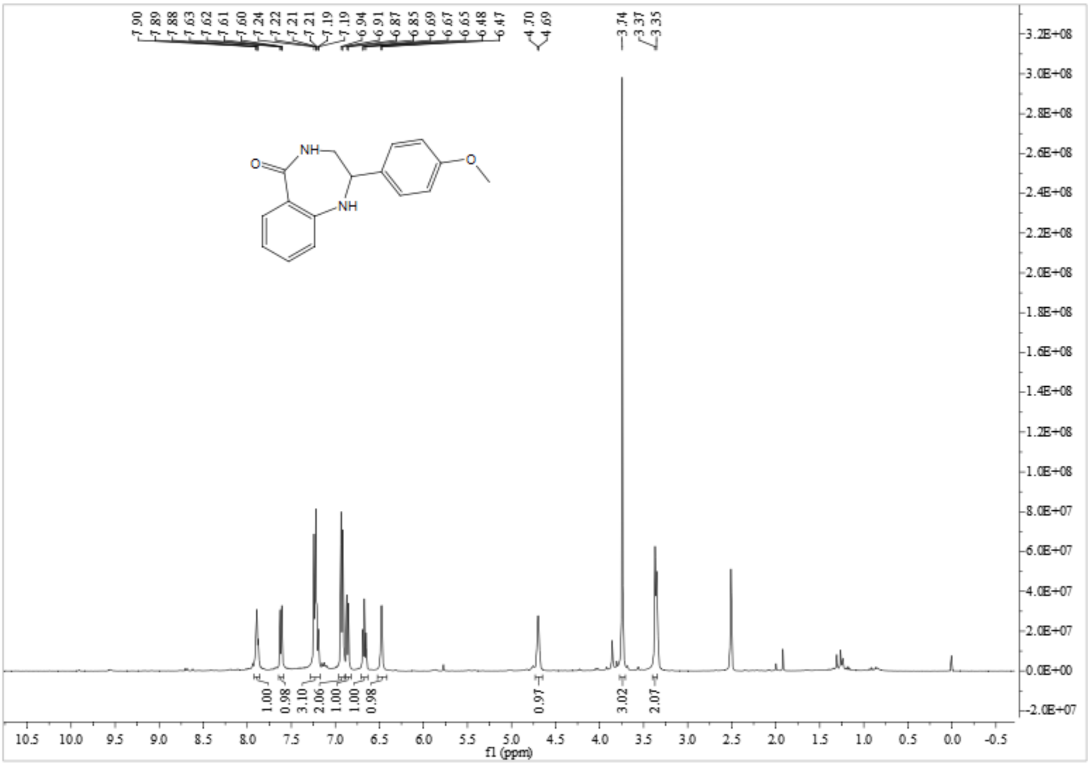

^{1}H-NMR spectrum of compound H20

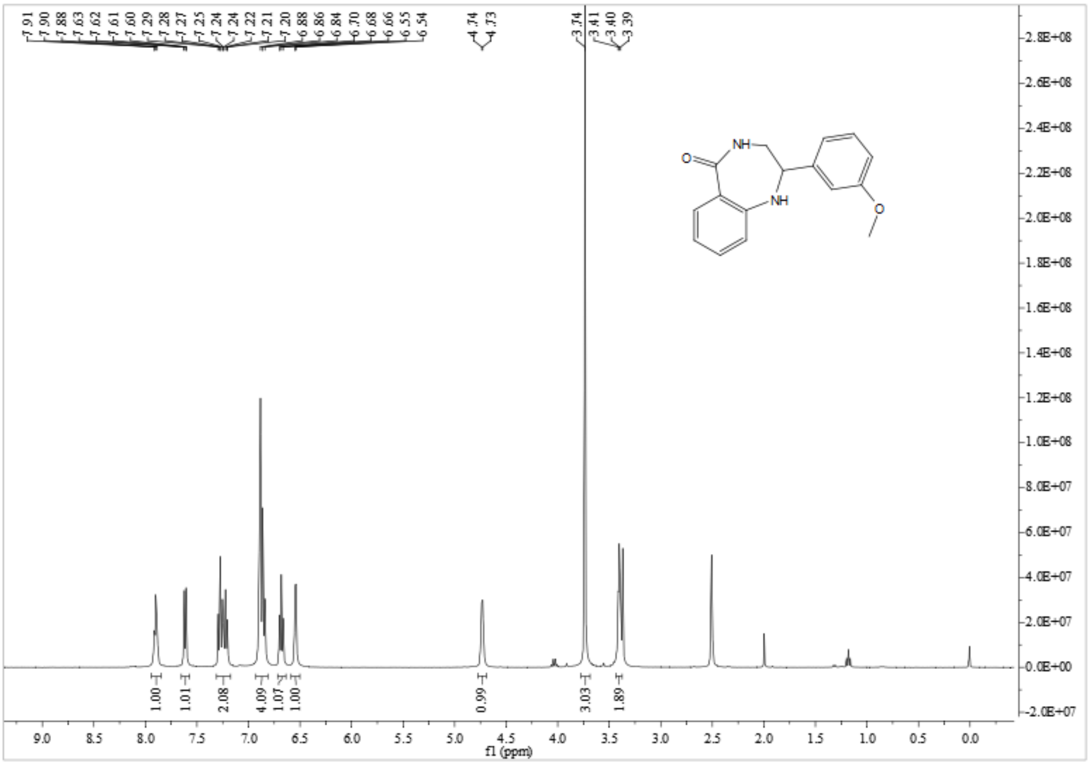

^{1}H-NMR spectrum of compound H21

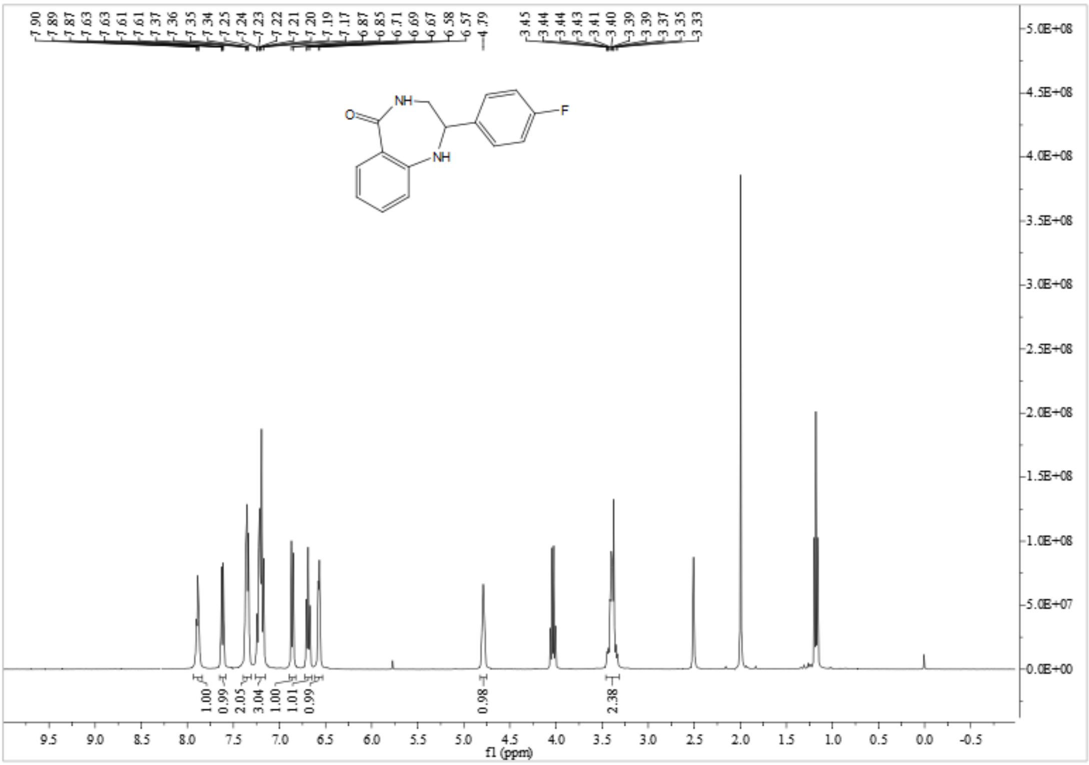

^{1}H-NMR spectrum of compound H22

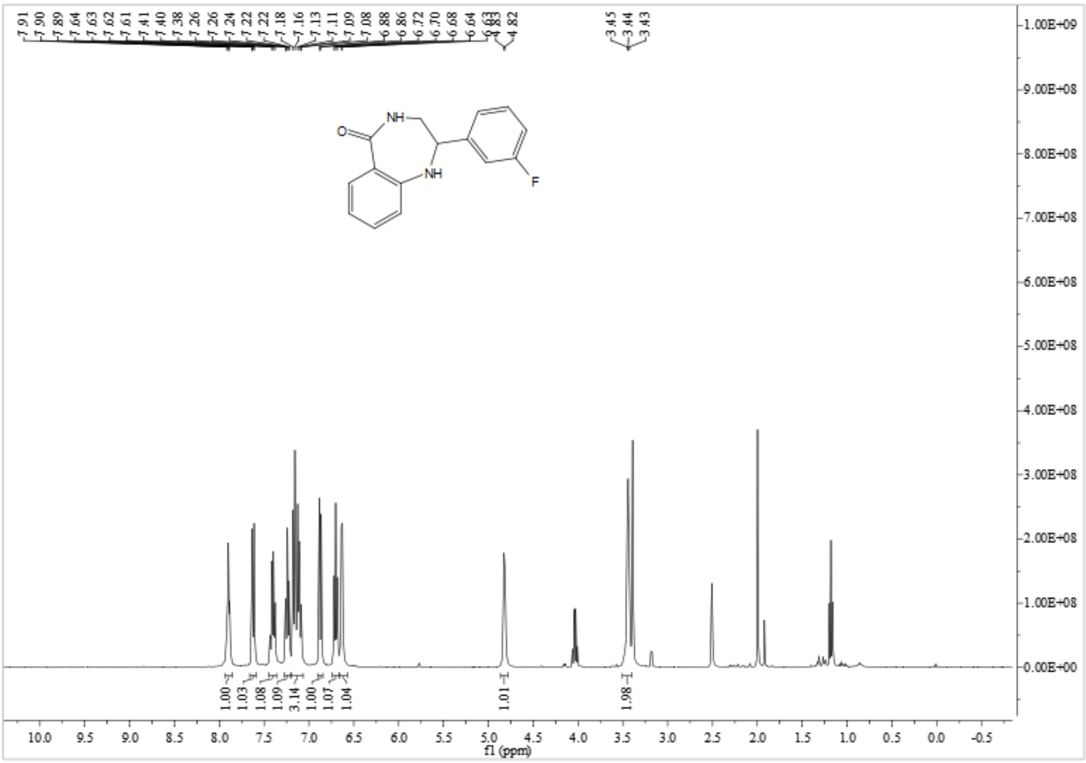

^{1}H-NMR spectrum of compound H23

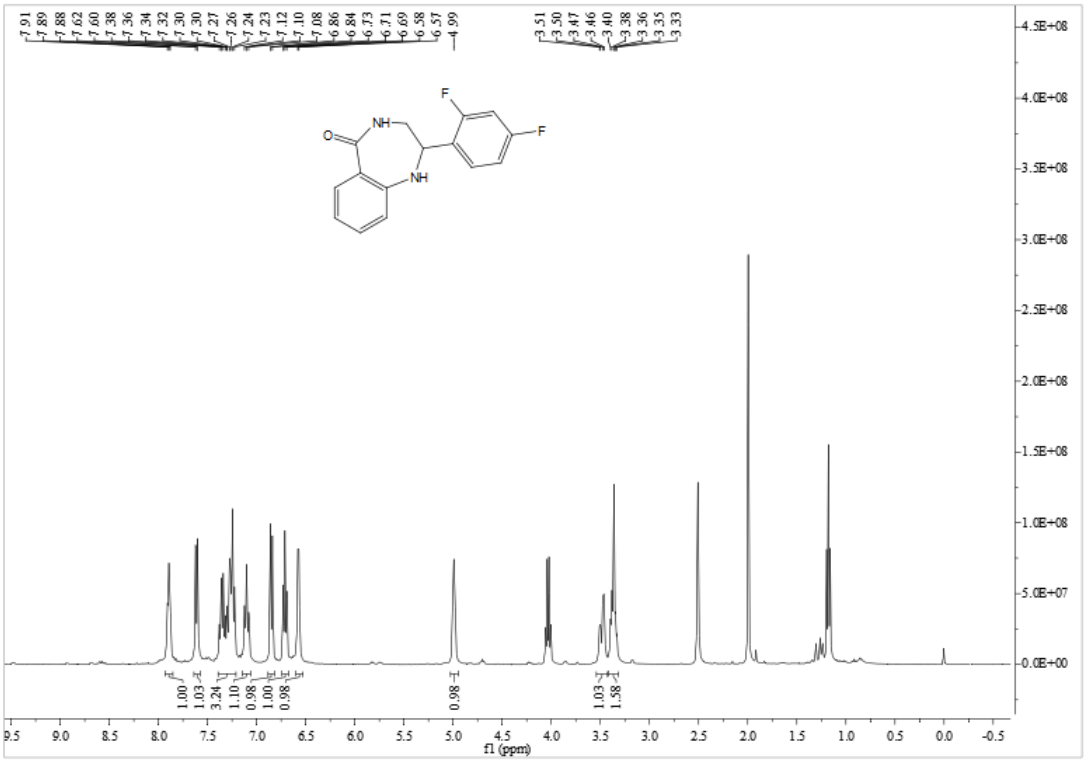

^{1}H-NMR spectrum of compound H24

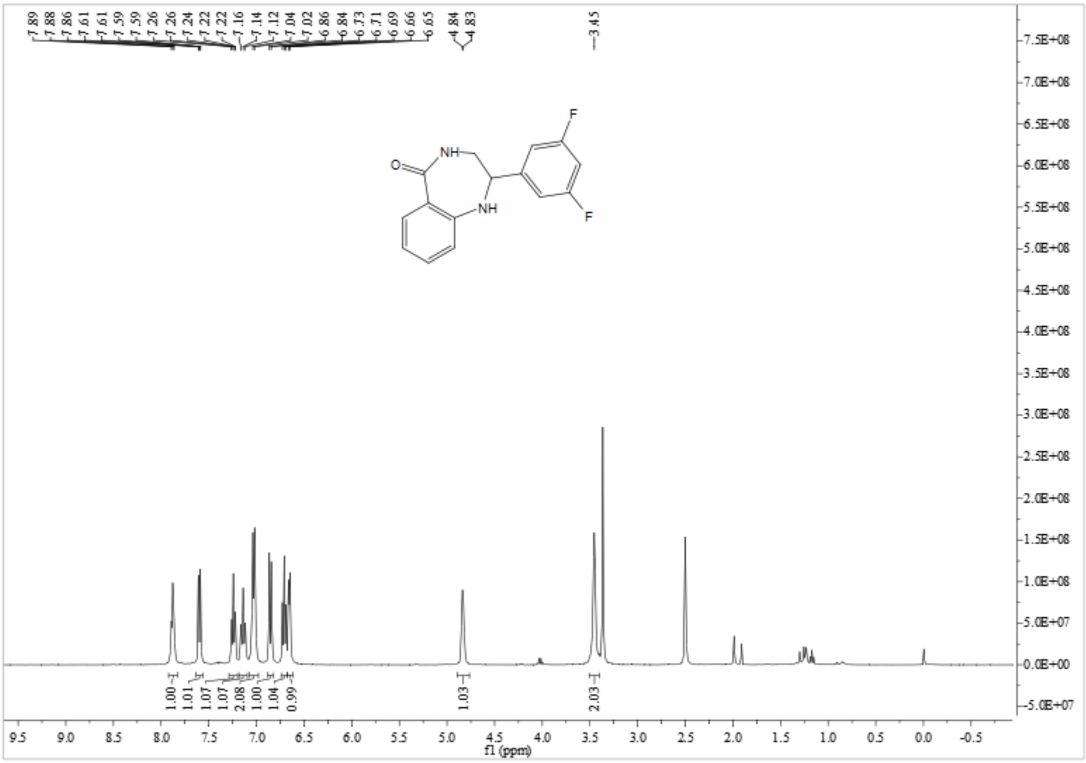

^{1}H-NMR spectrum of compound H25

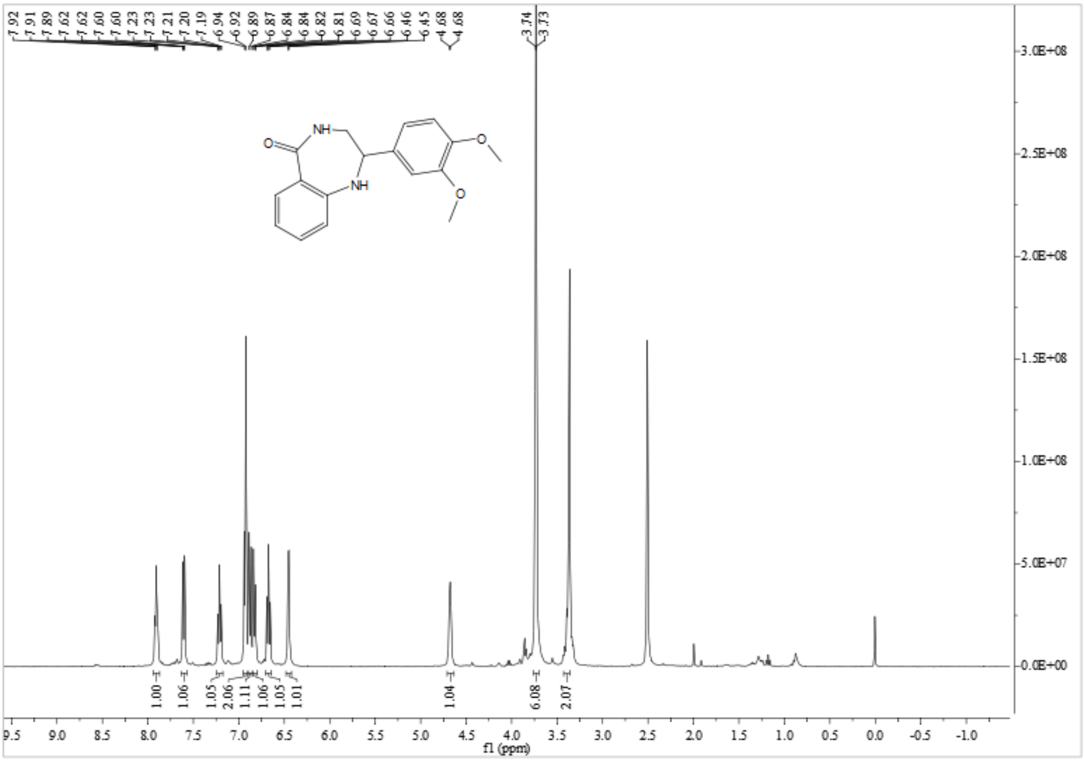

^{1}H-NMR spectrum of compound H26

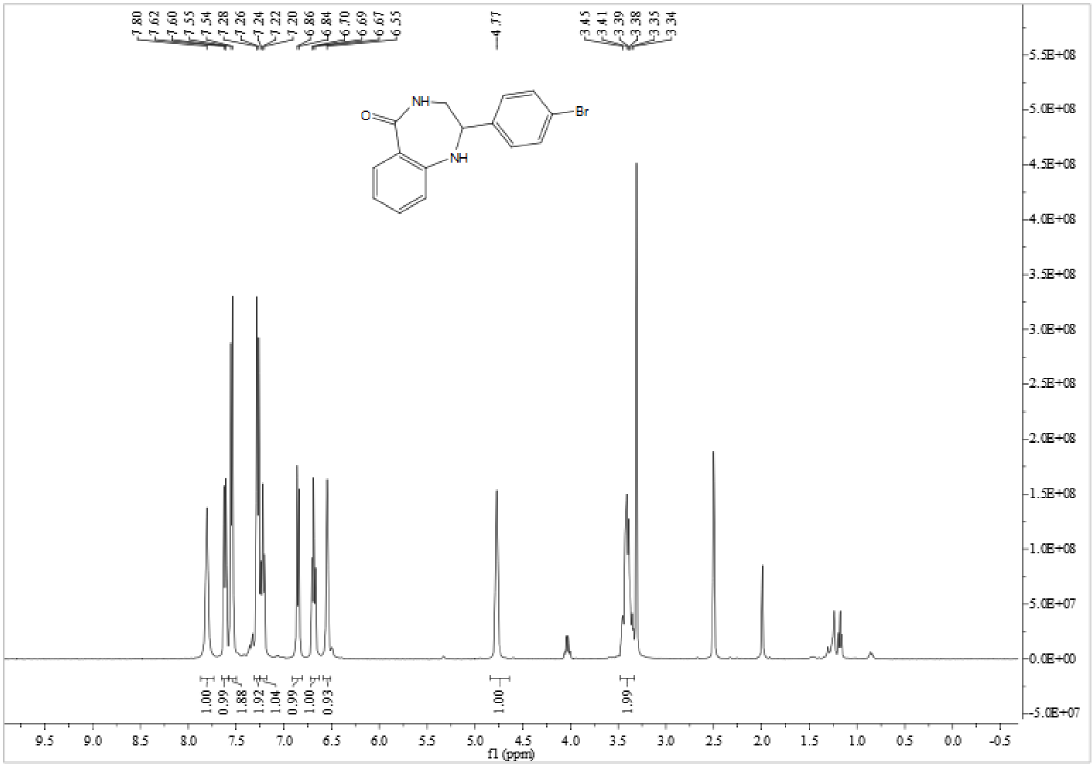

^{1}H-NMR spectrum of compound H27

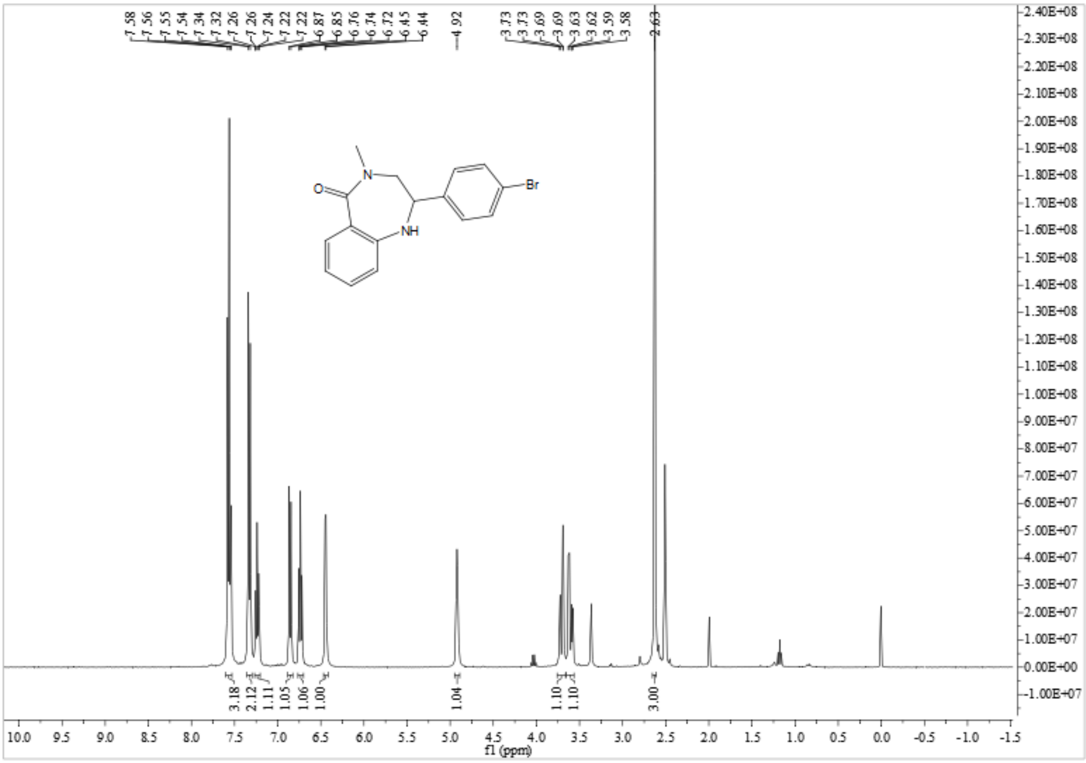

^{1}H-NMR spectrum of compound H28

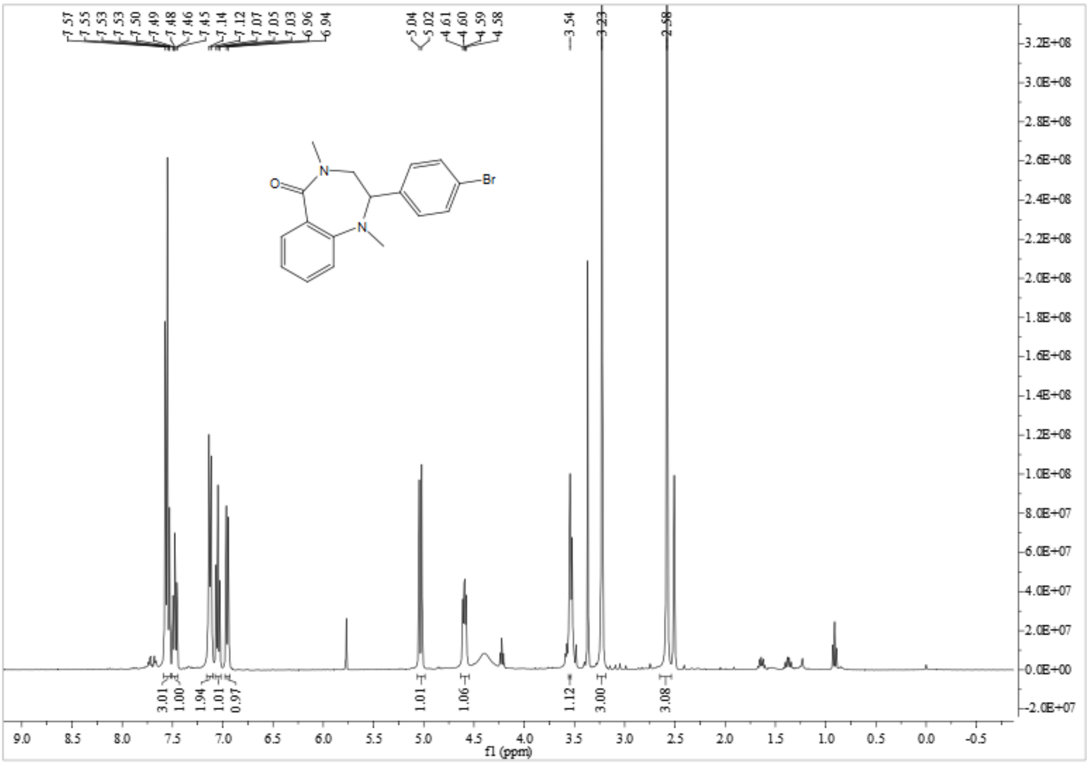

^{1}H-NMR spectrum of compound H29

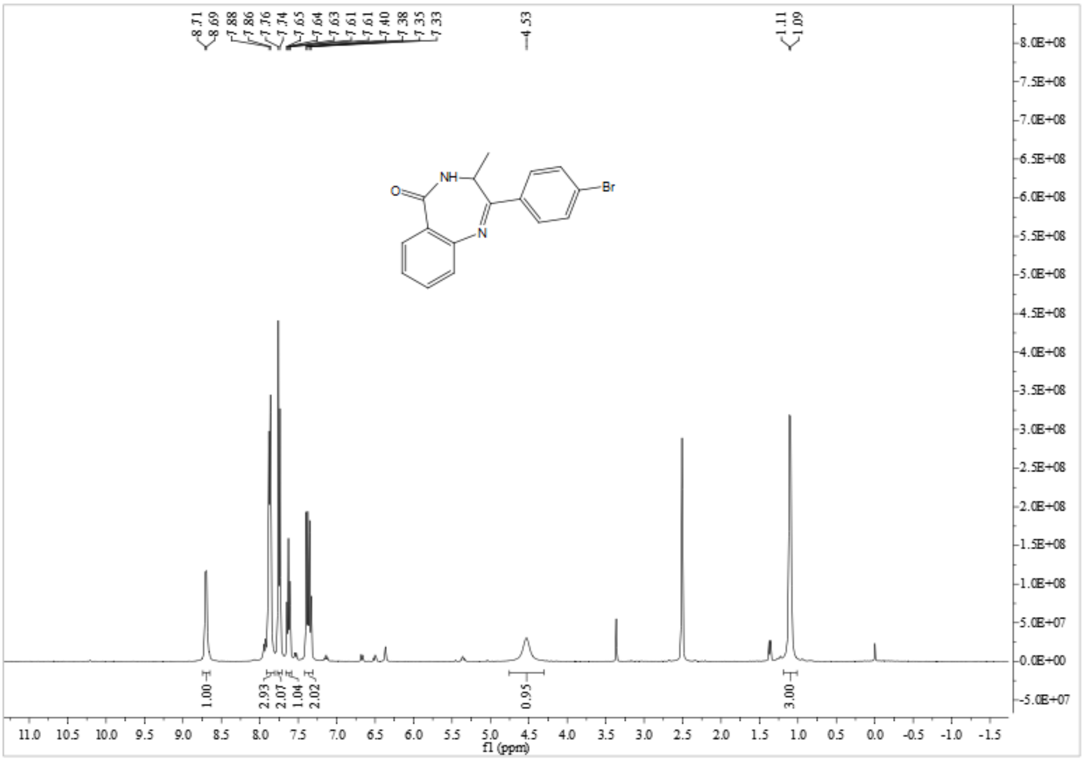

^{1}H-NMR spectrum of compound H30

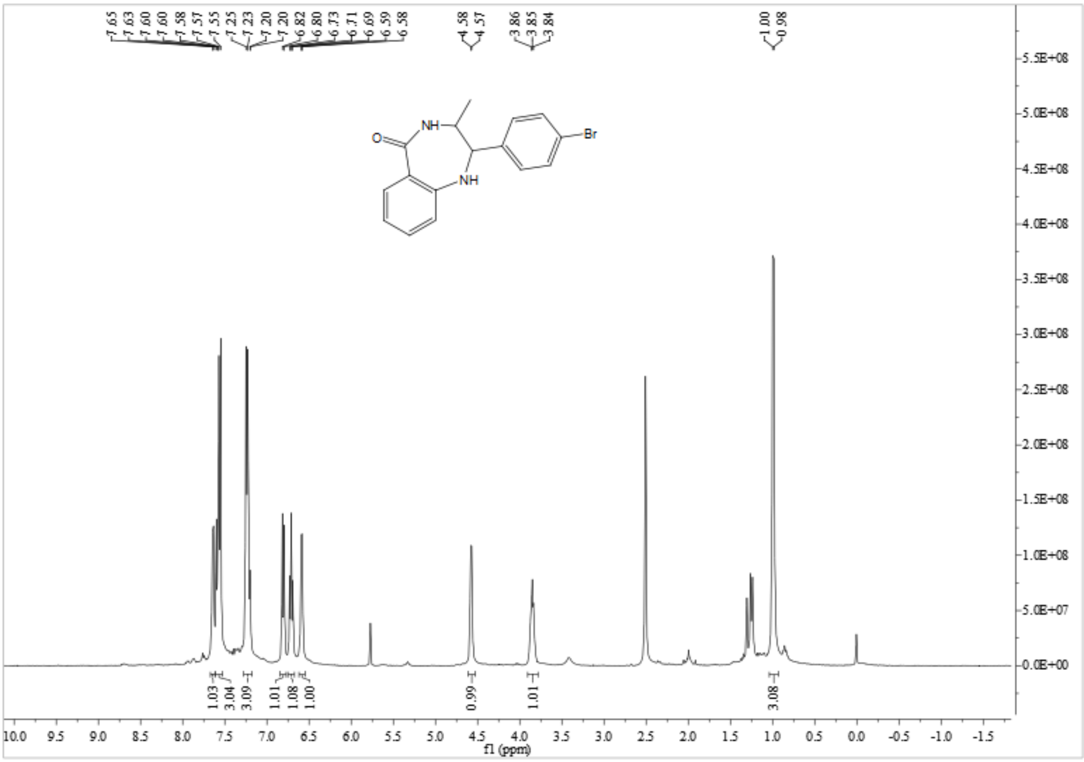

^{1}H-NMR spectrum of compound H31

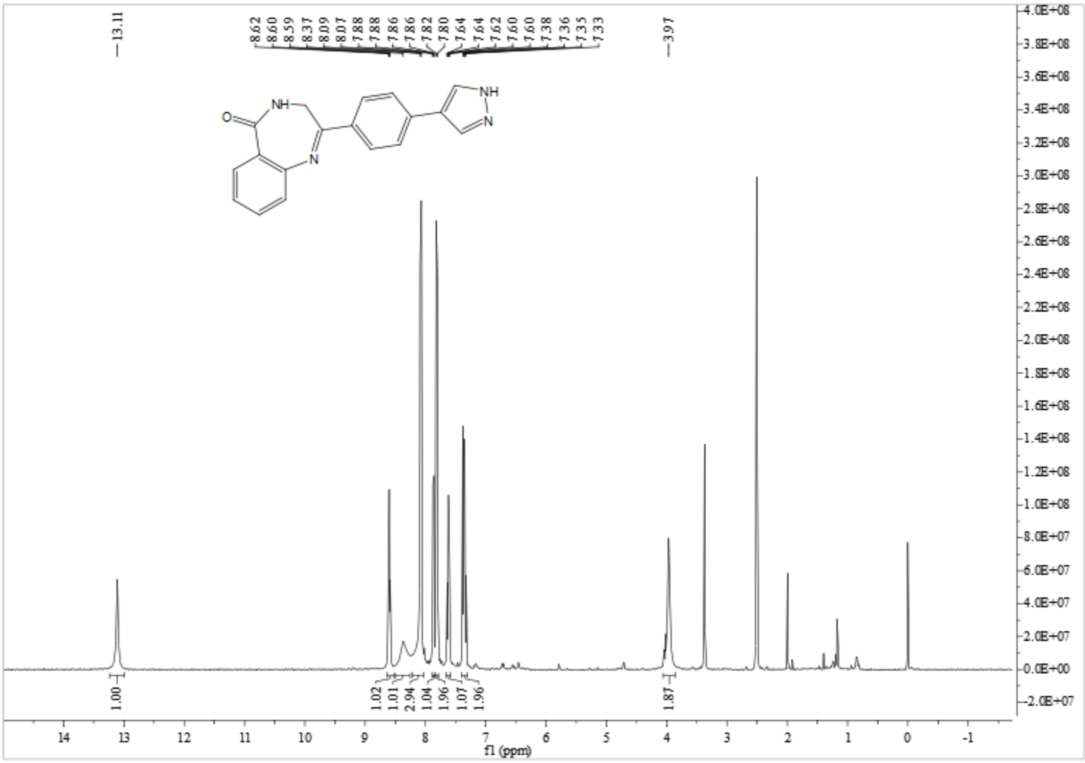

^{1}H-NMR spectrum of compound H32

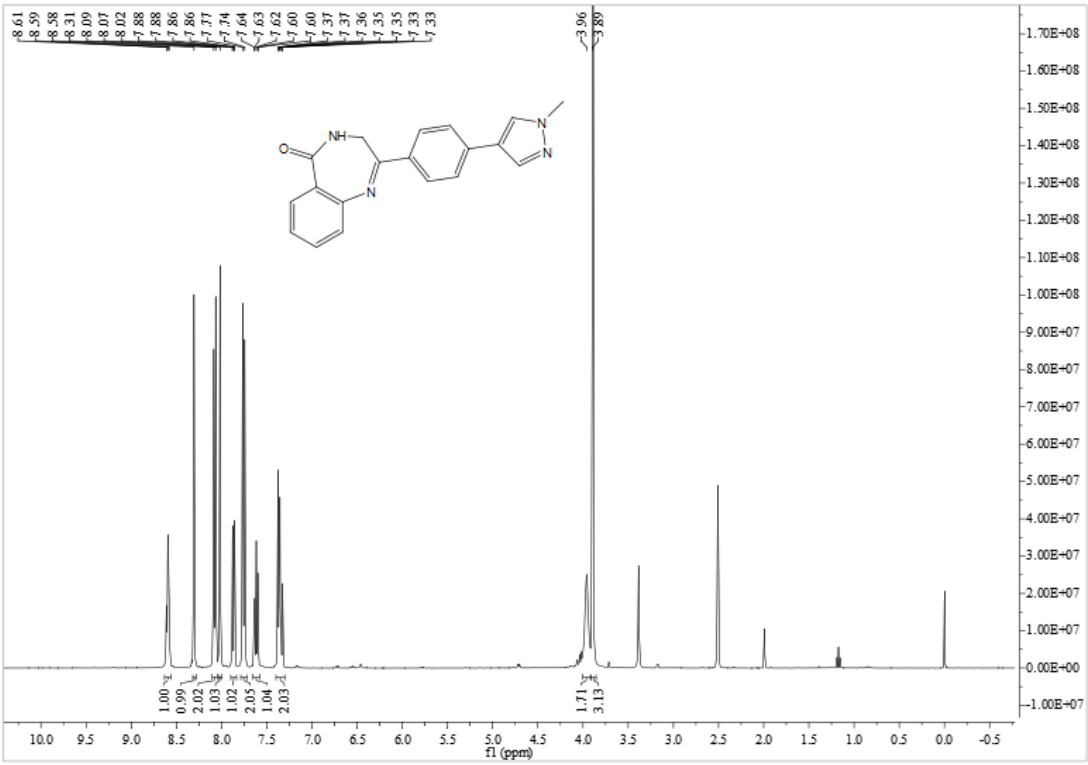

^{1}H-NMR spectrum of compound H33

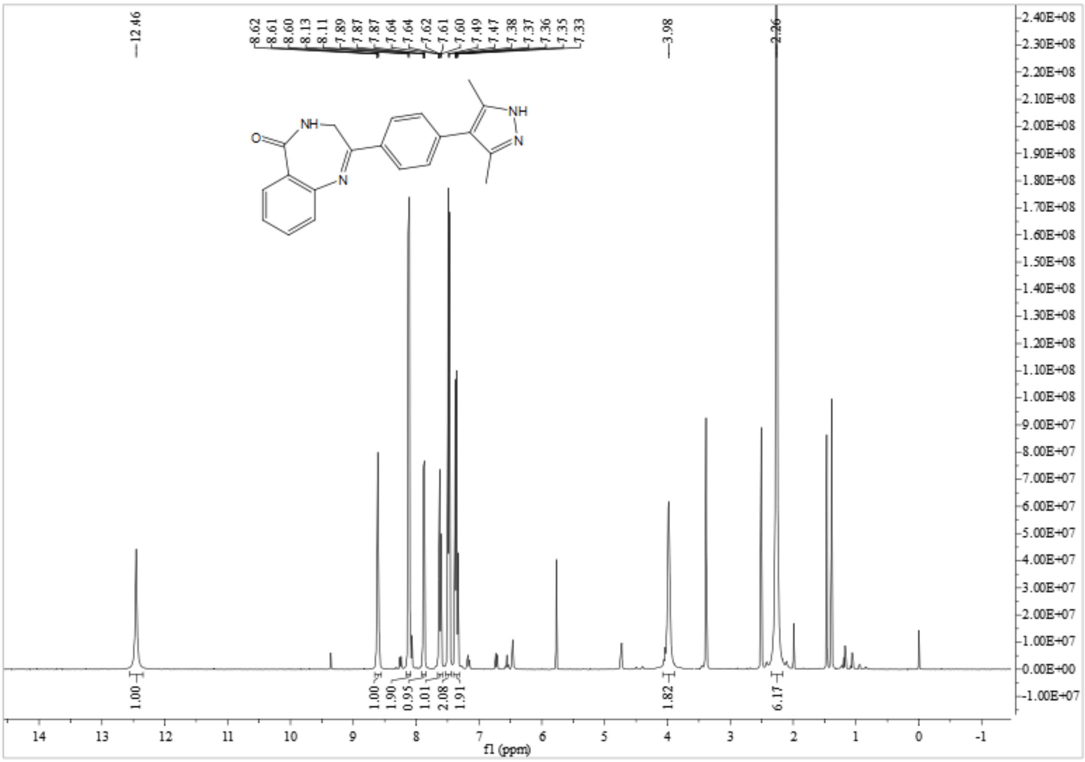

^{1}H-NMR spectrum of compound H34

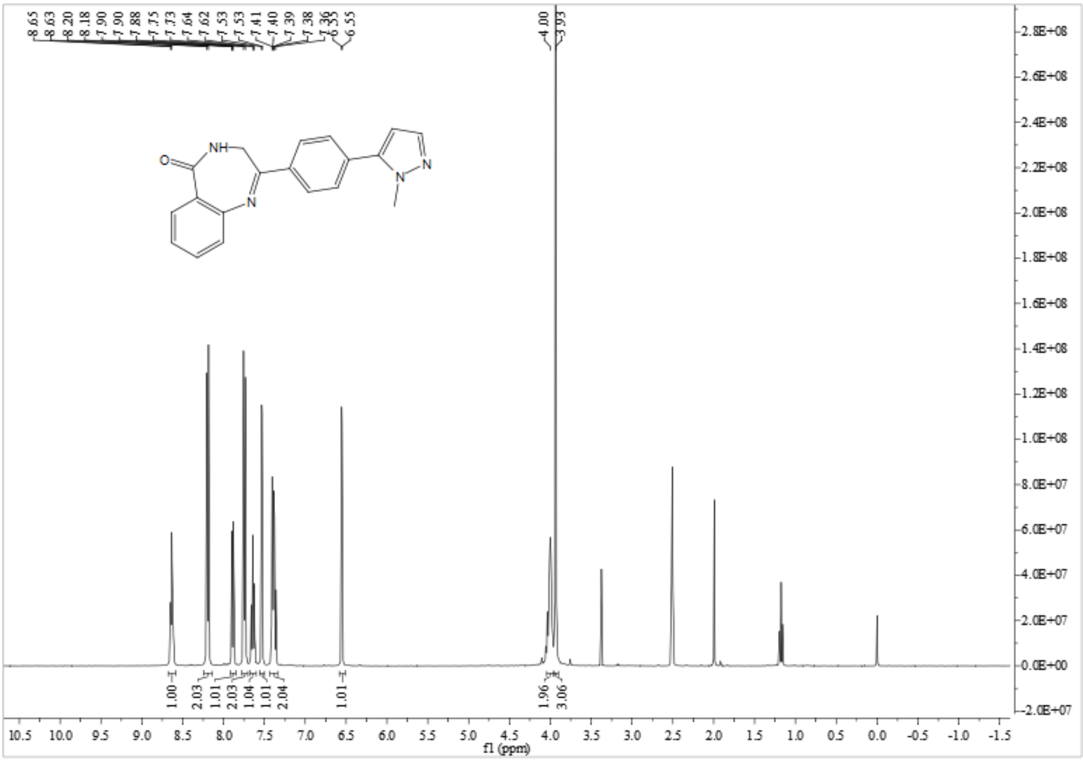

^{1}H-NMR spectrum of compound H35

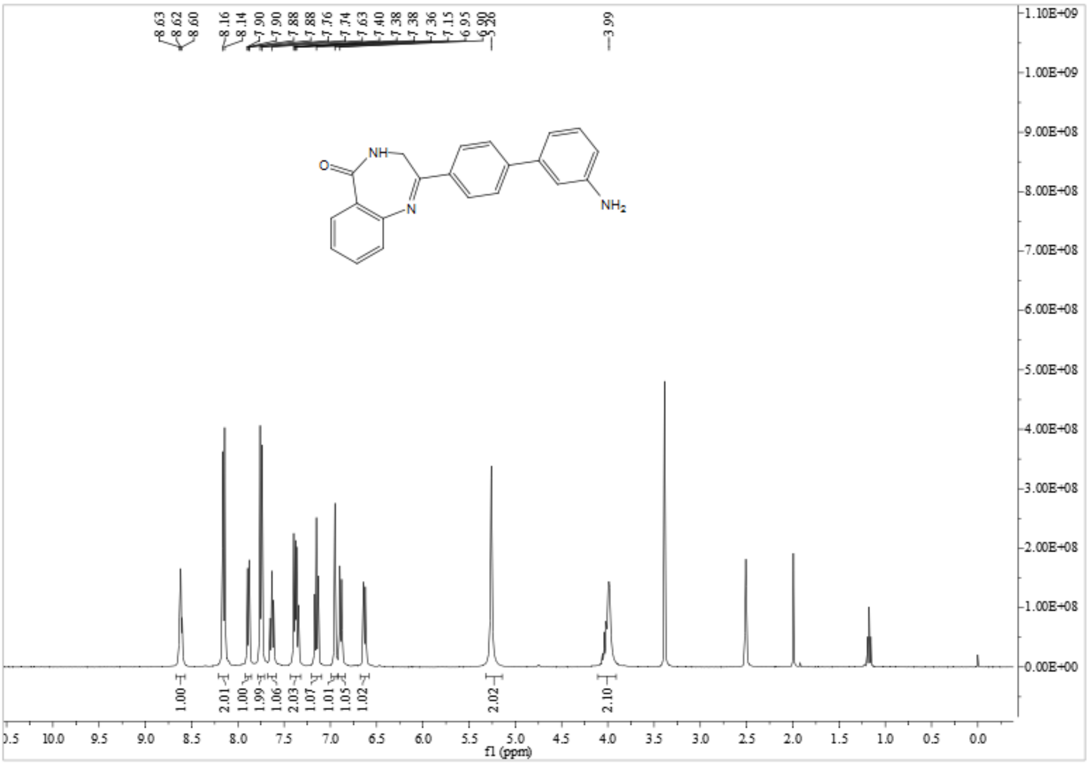

^{1}H-NMR spectrum of compound H36

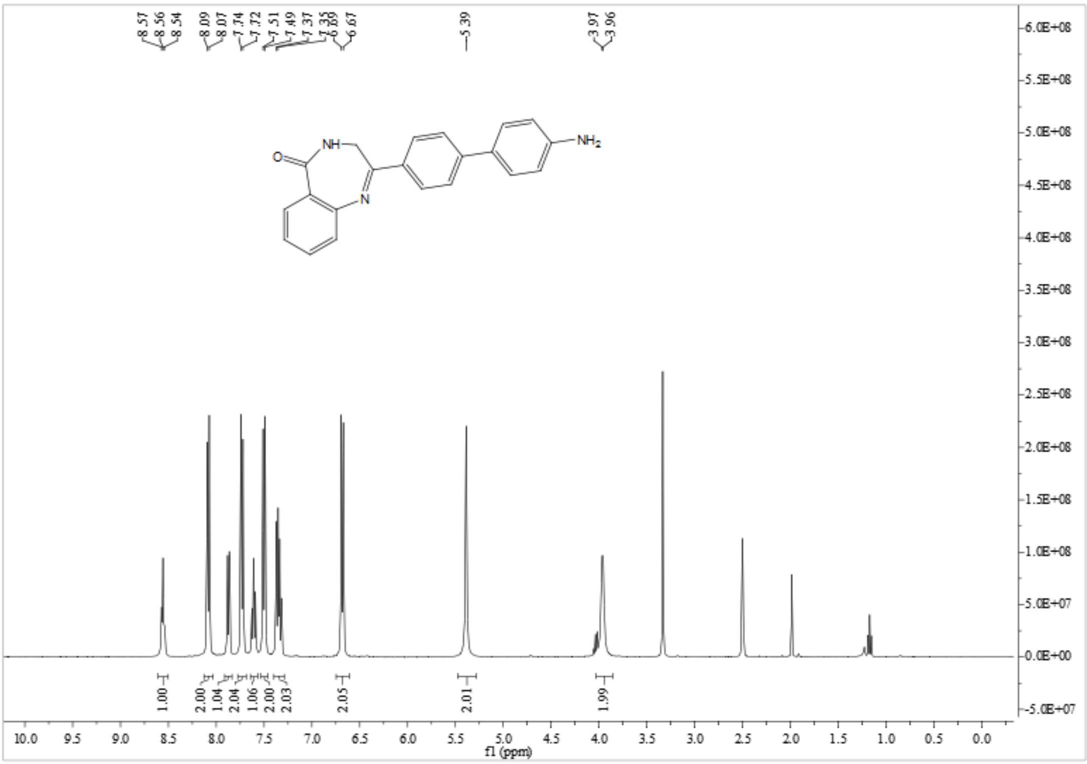

^{1}H-NMR spectrum of compound H37

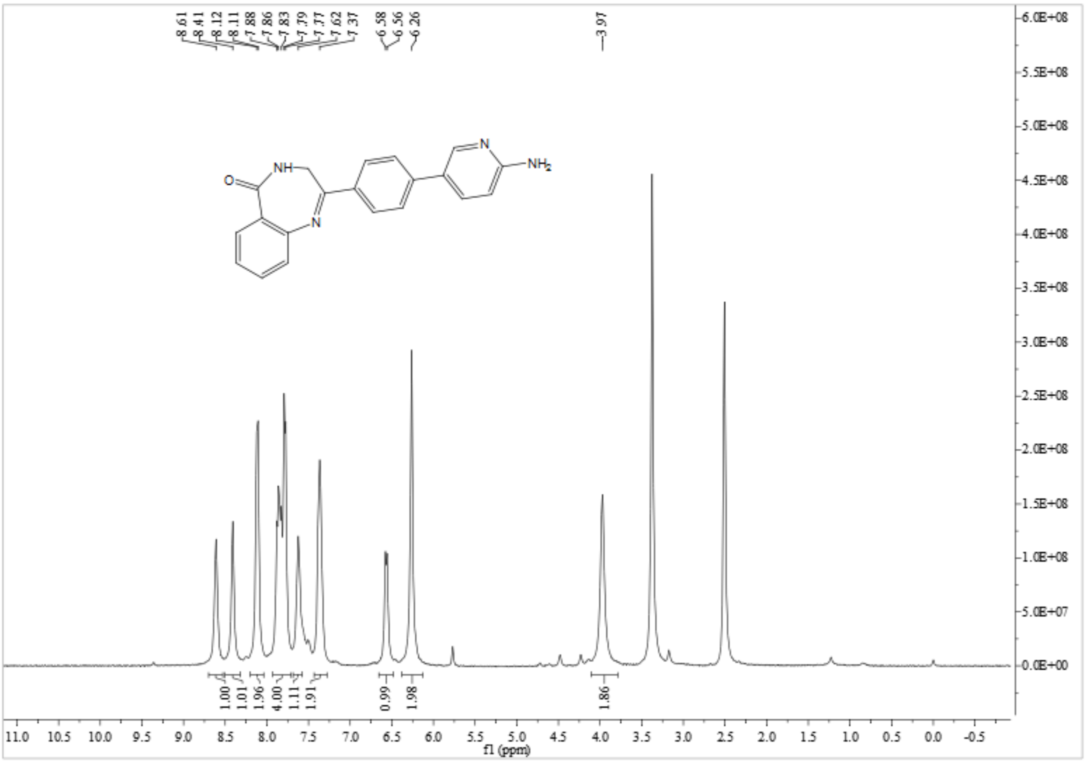

^{1}H-NMR spectrum of compound H38

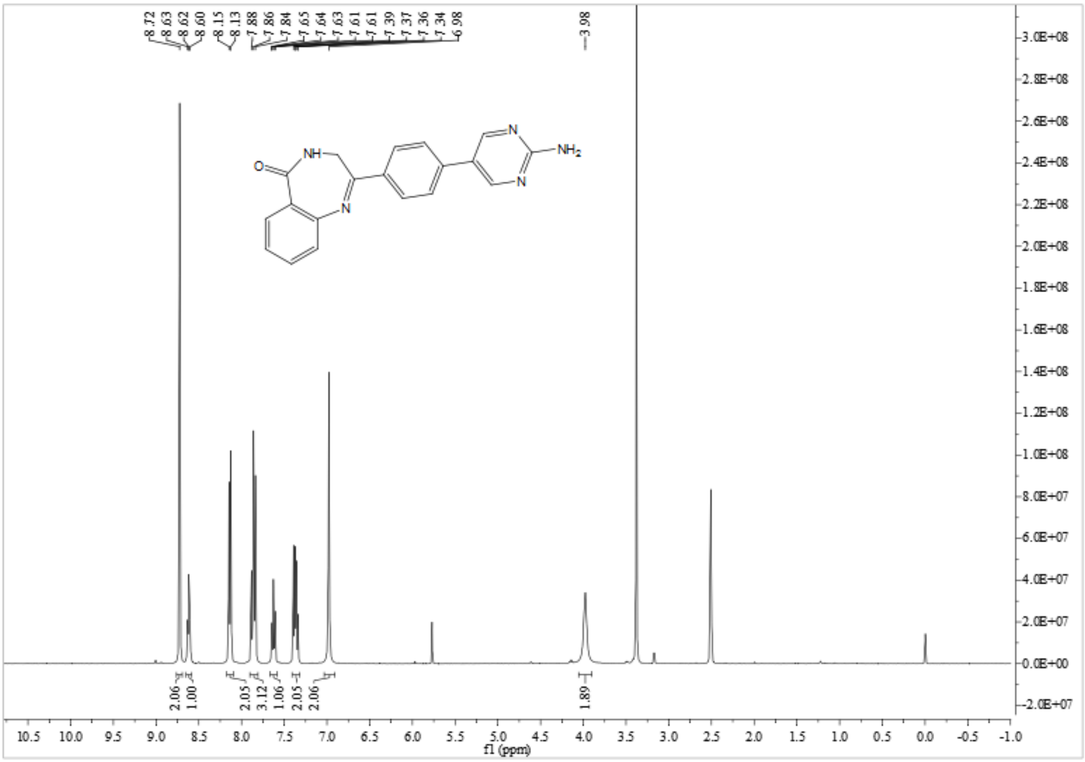

^{1}H-NMR spectrum of compound H39

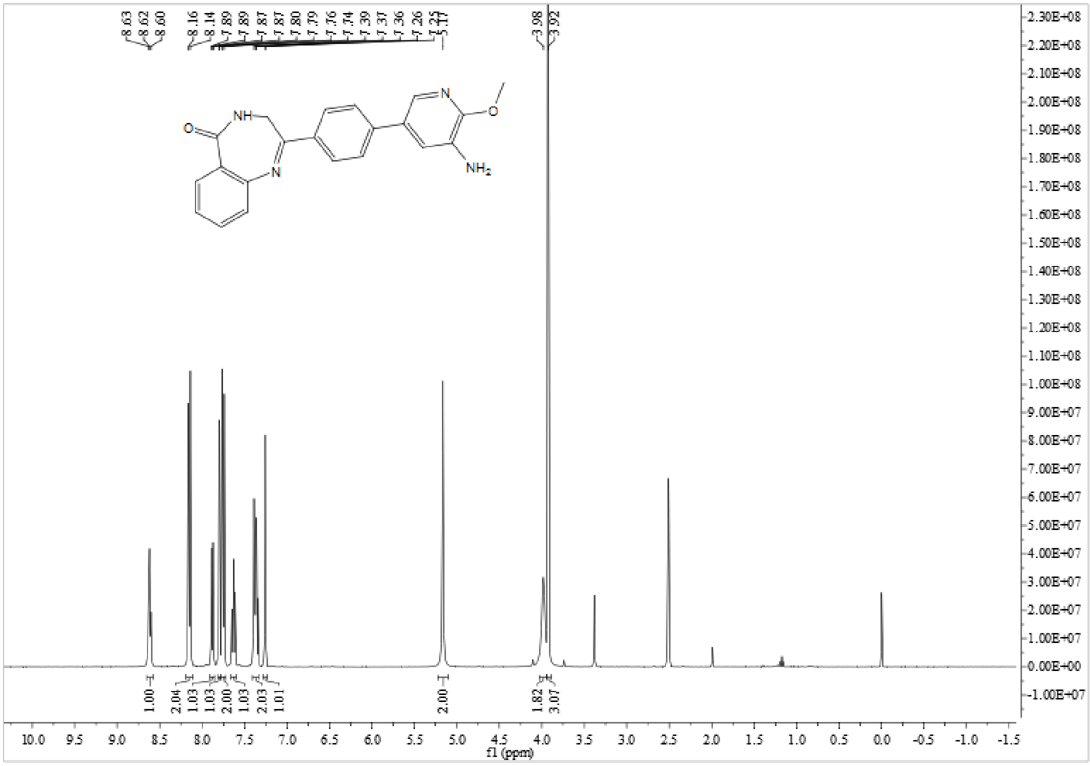

^{1}H-NMR spectrum of compound H40

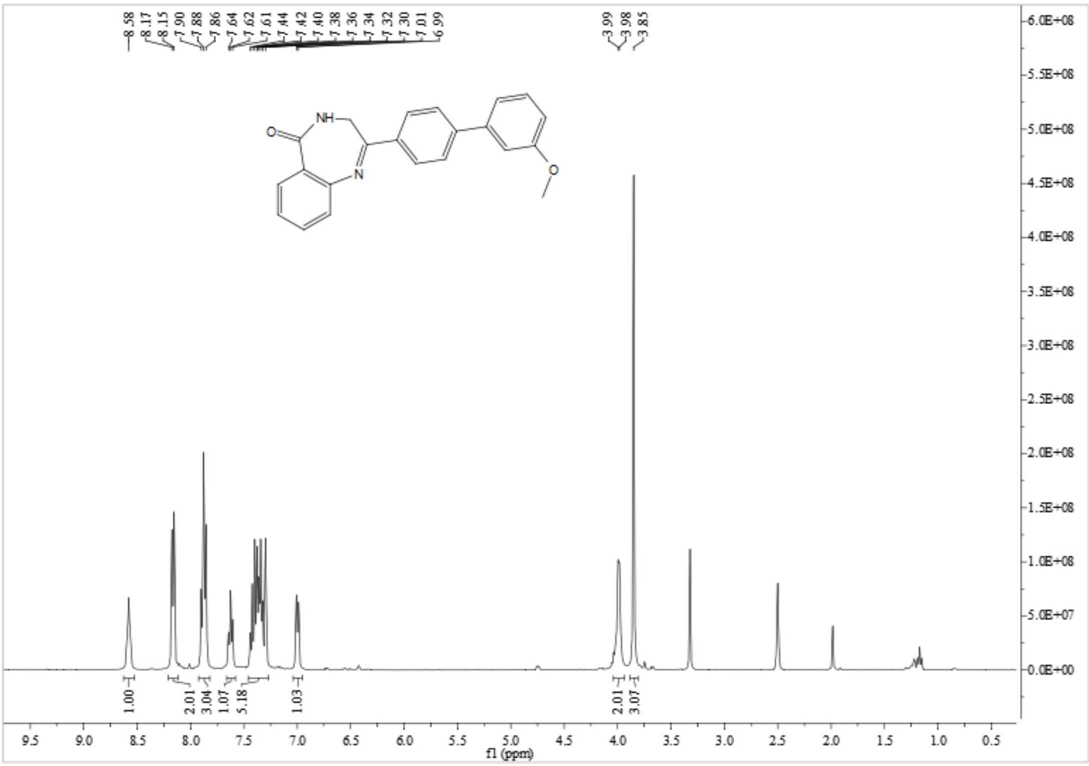

^{1}H-NMR spectrum of compound H41

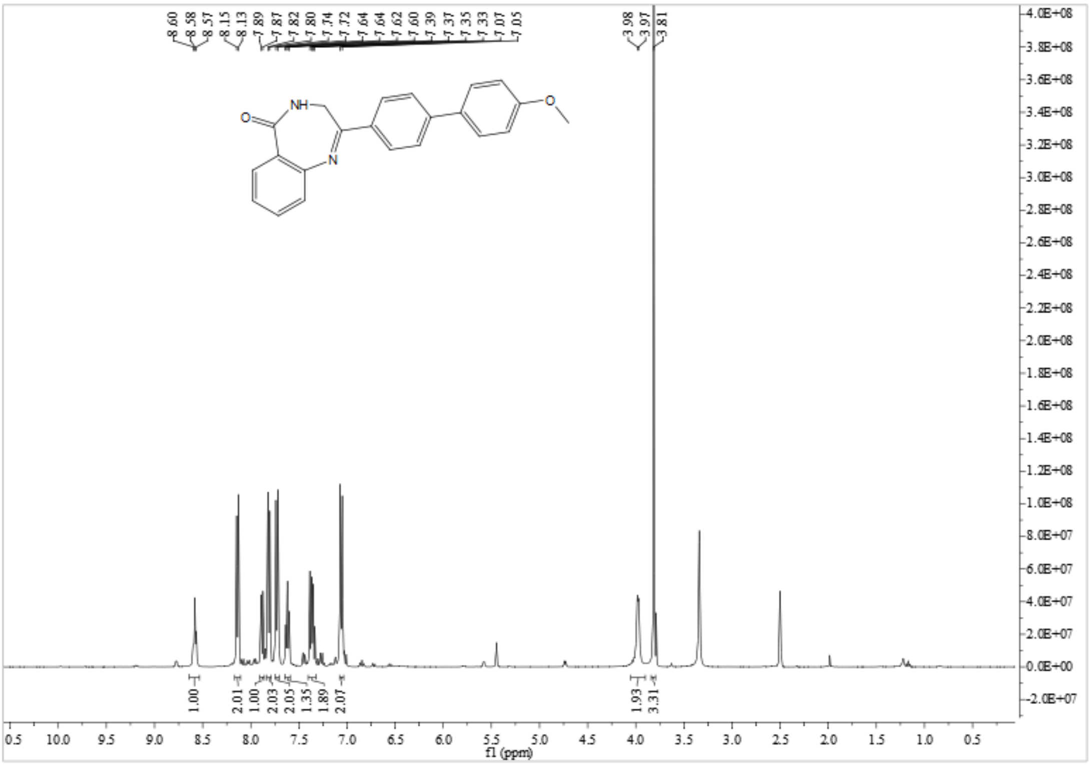

^{1}H-NMR spectrum of compound H42

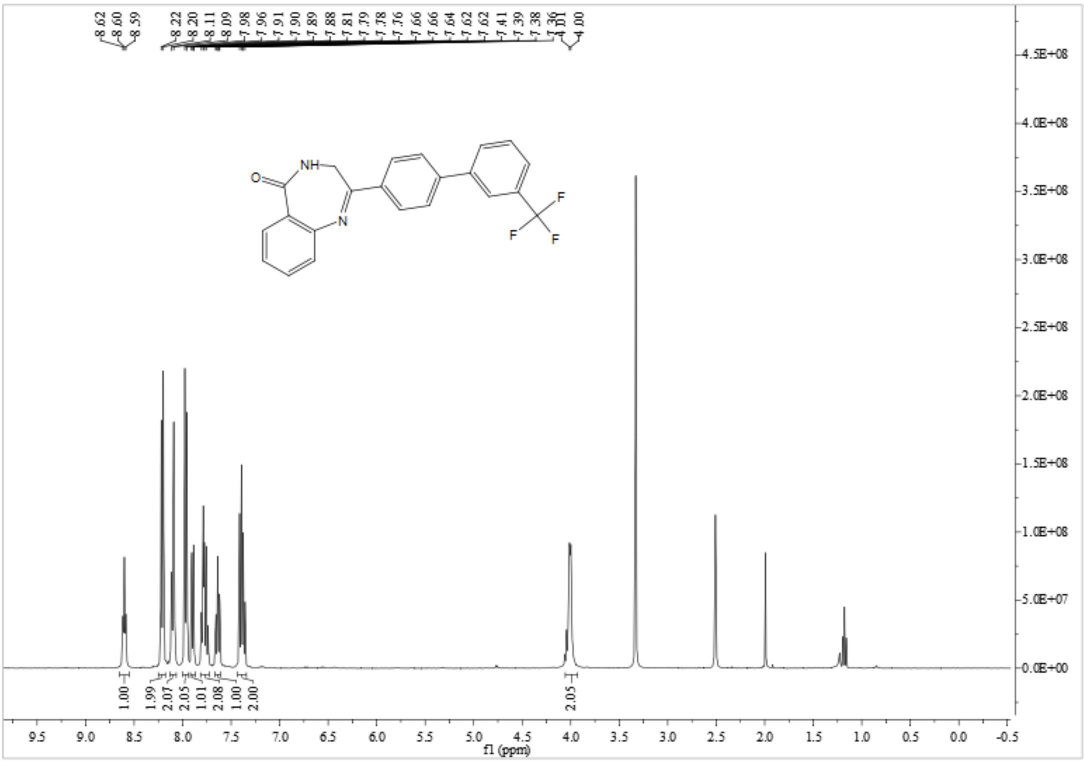

^{1}H-NMR spectrum of compound H43

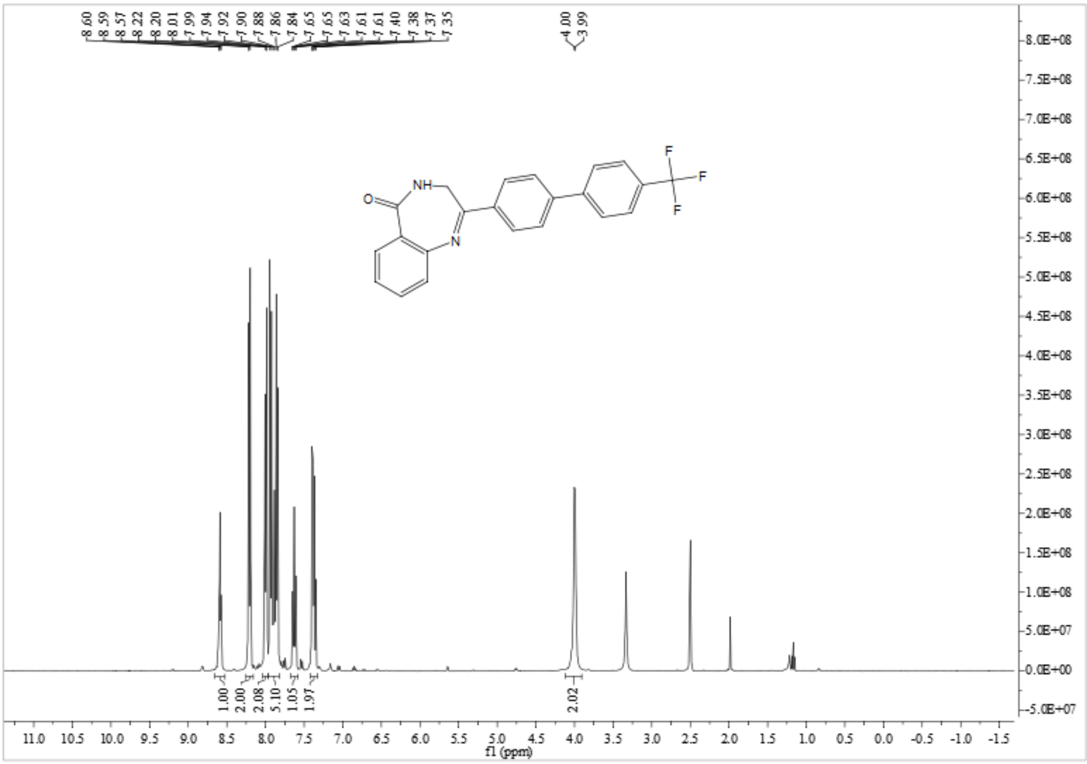

^{1}H-NMR spectrum of compound H44

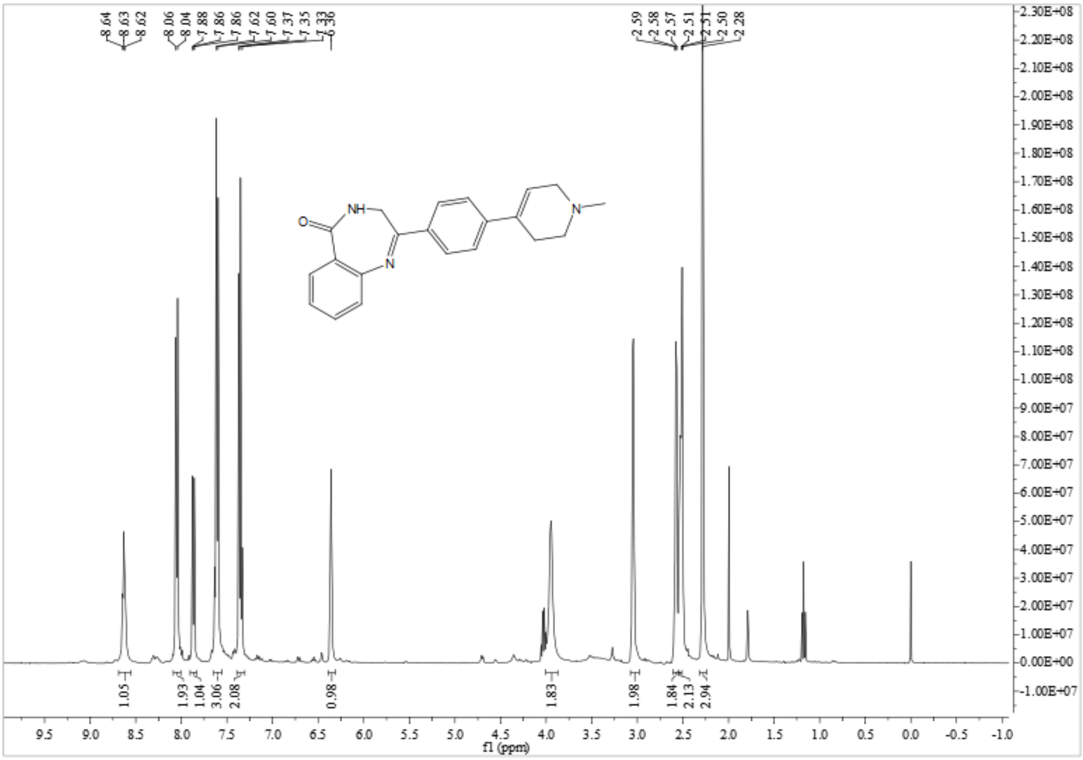

^{1}H-NMR spectrum of compound H45

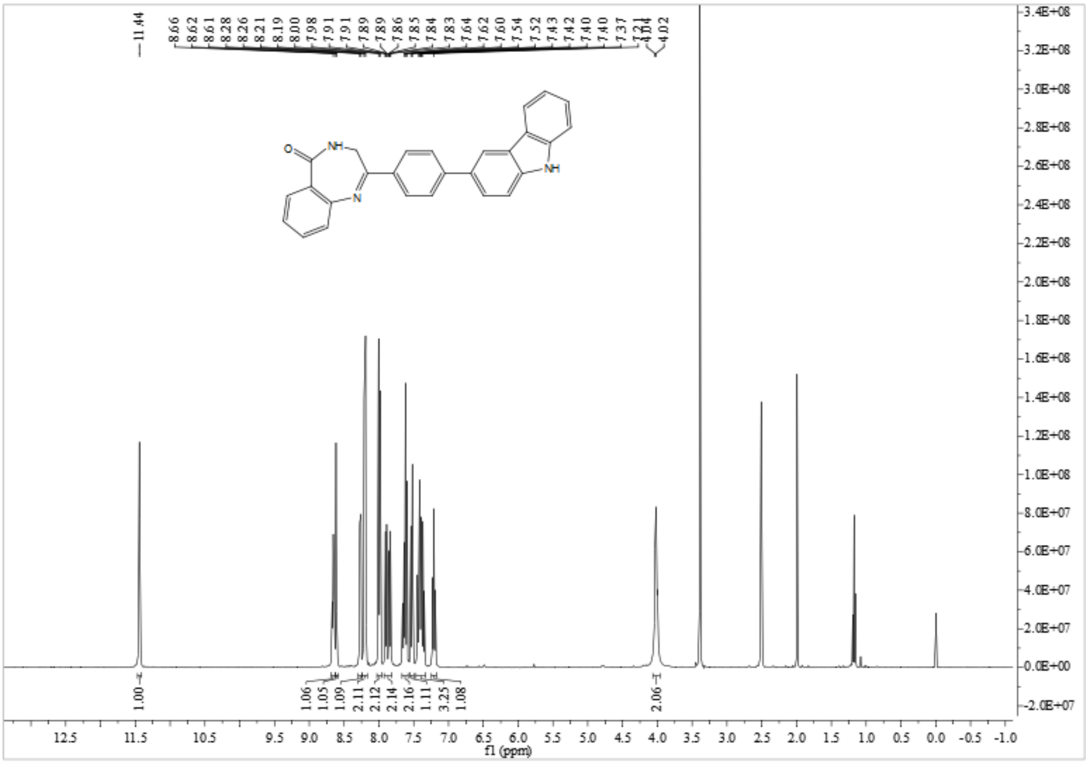

^{1}H-NMR spectrum of compound H46

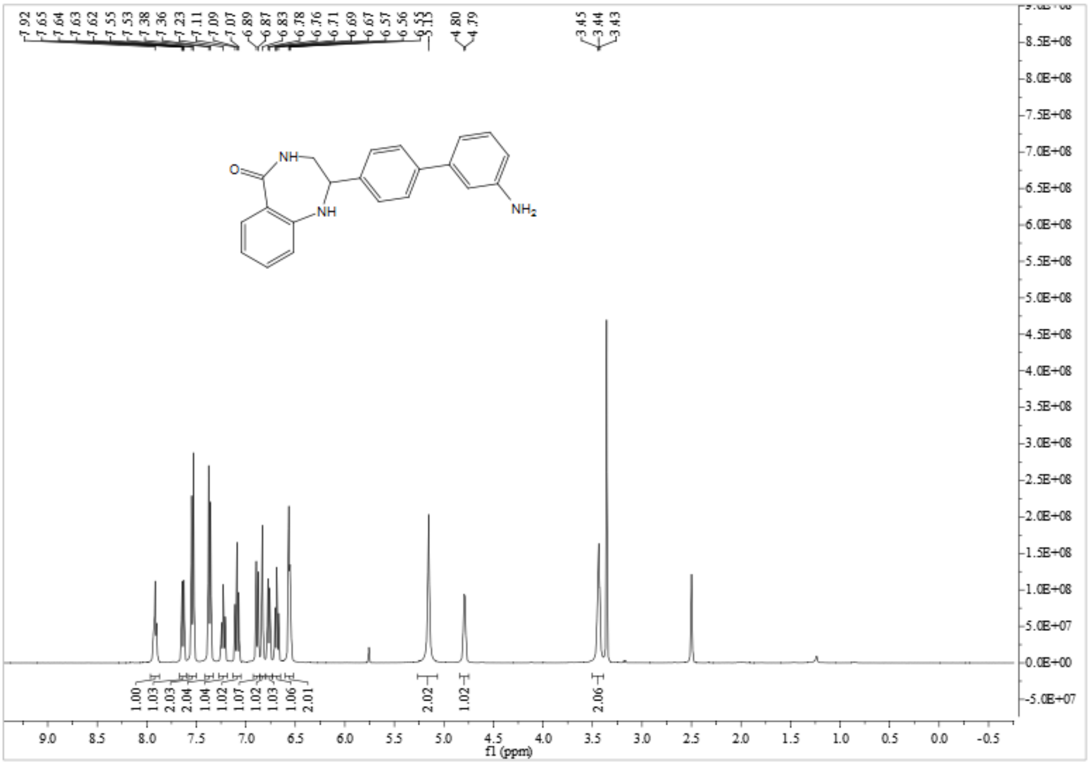

^{1}H-NMR spectrum of compound H47

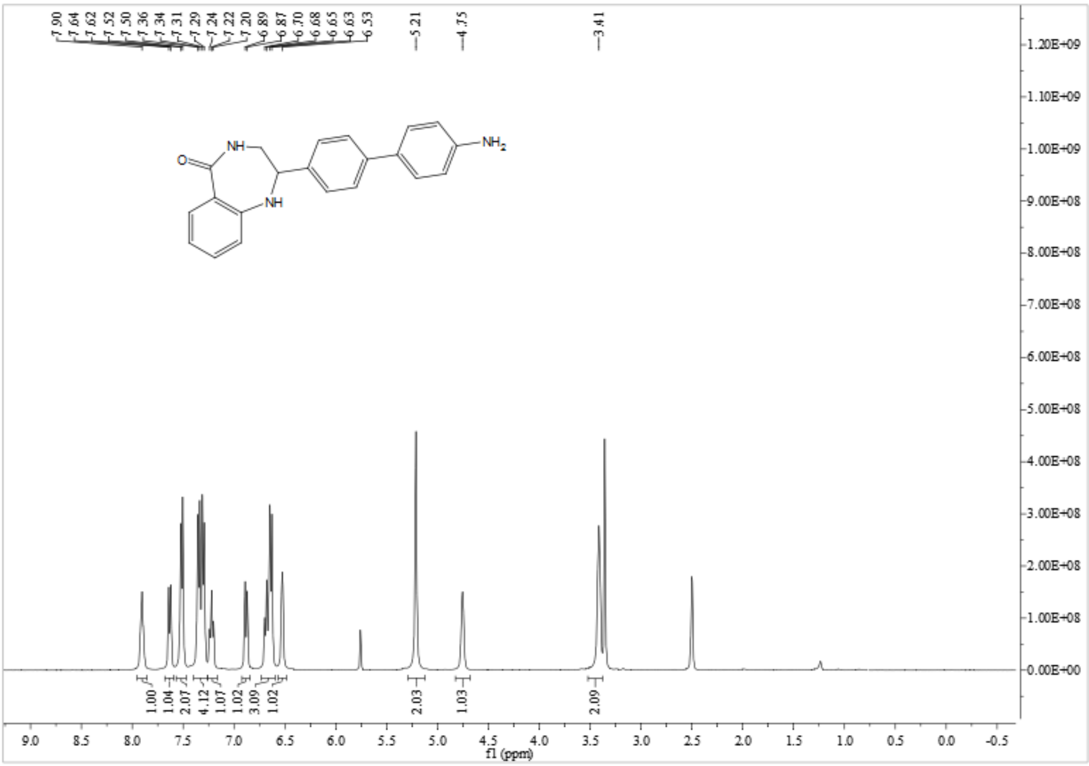

^{1}H-NMR spectrum of compound H48

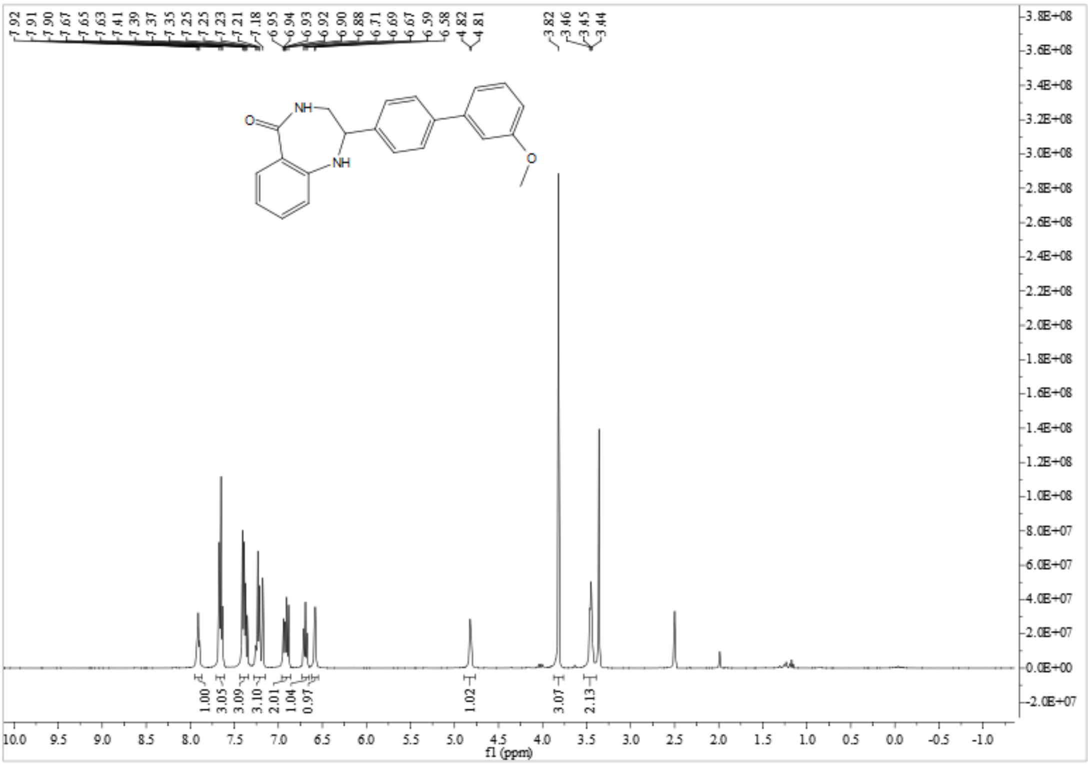

^{1}H-NMR spectrum of compound H49

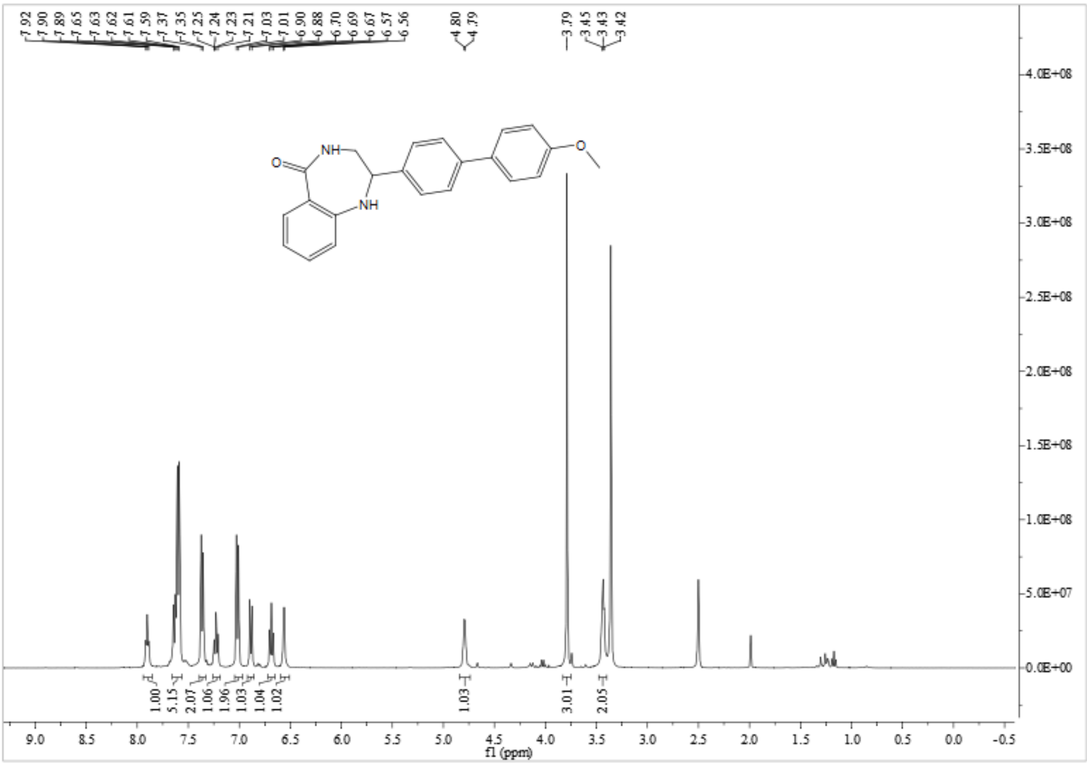

^{1}H-NMR spectrum of compound H50

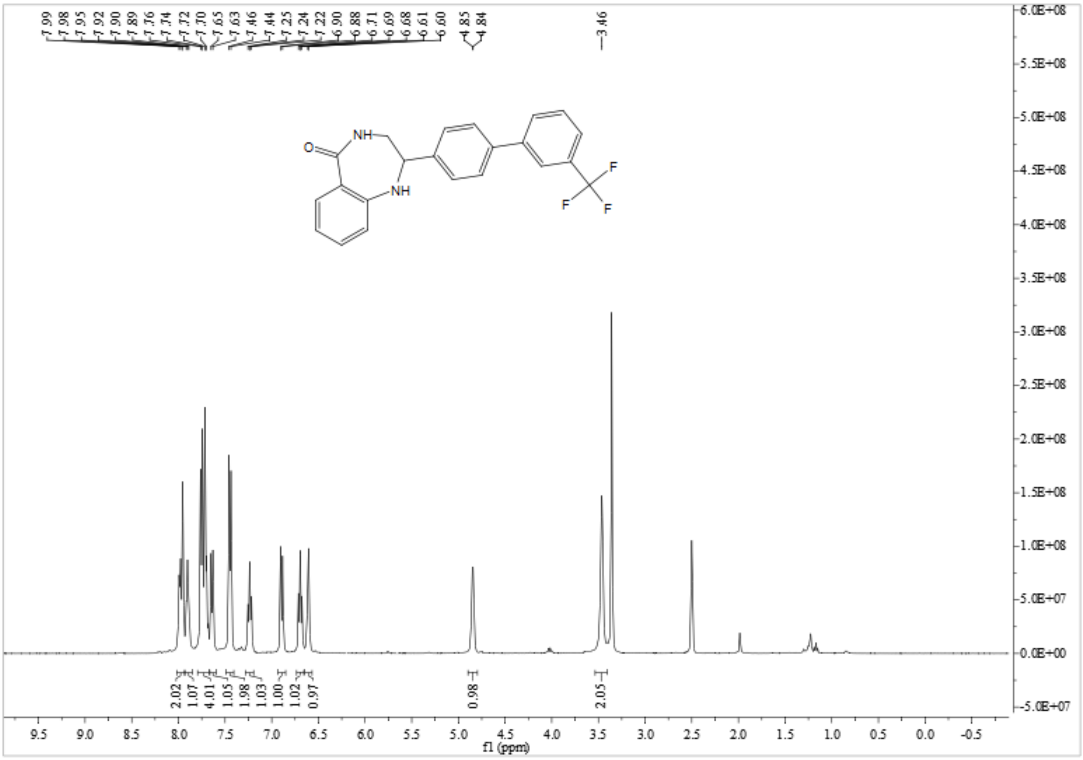

^{1}H-NMR spectrum of compound H51

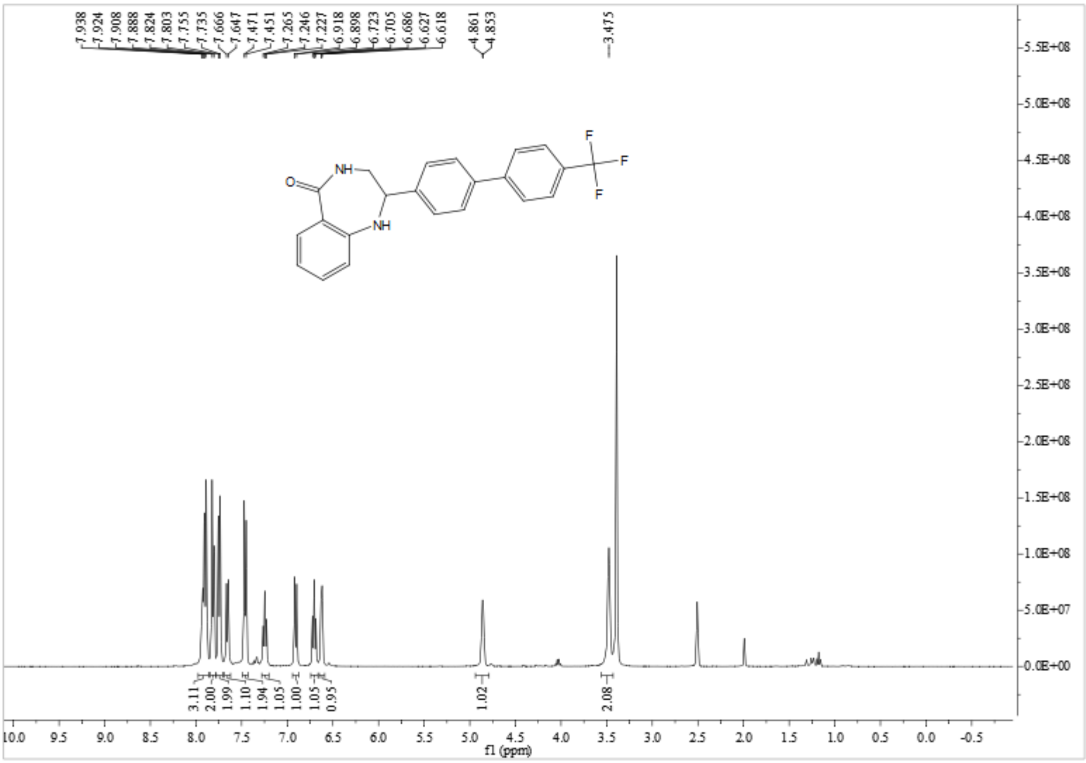

^{1}H-NMR spectrum of compound H52

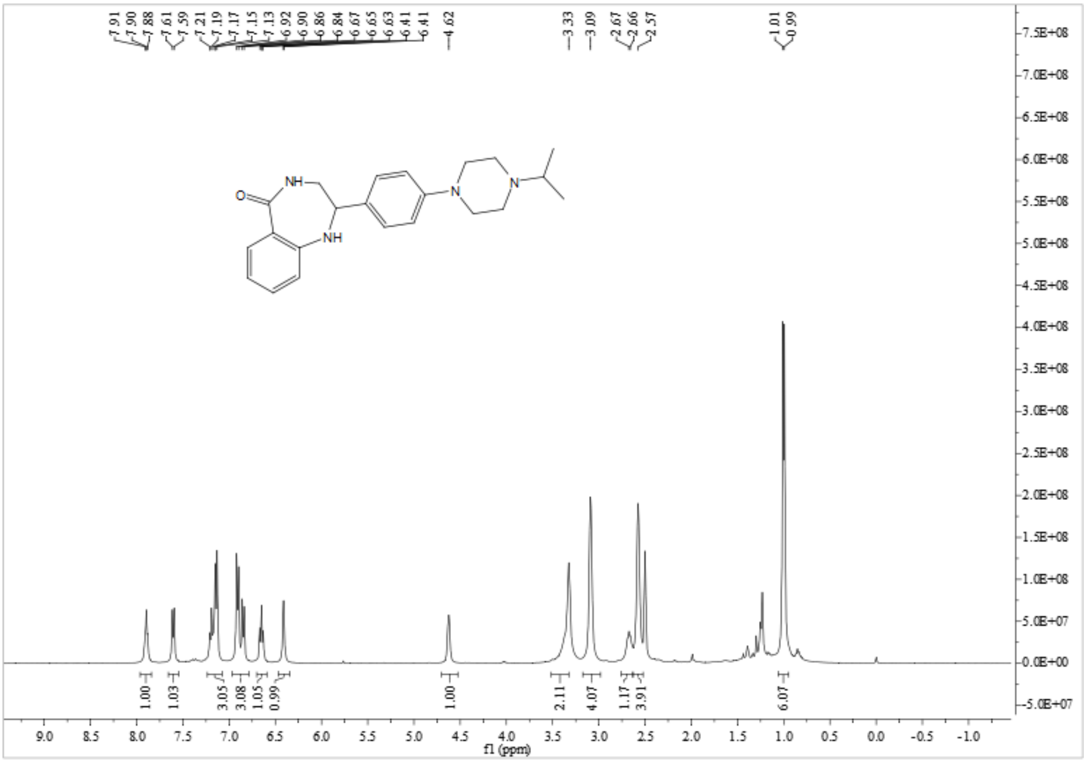

^{1}H-NMR spectrum of compound H53

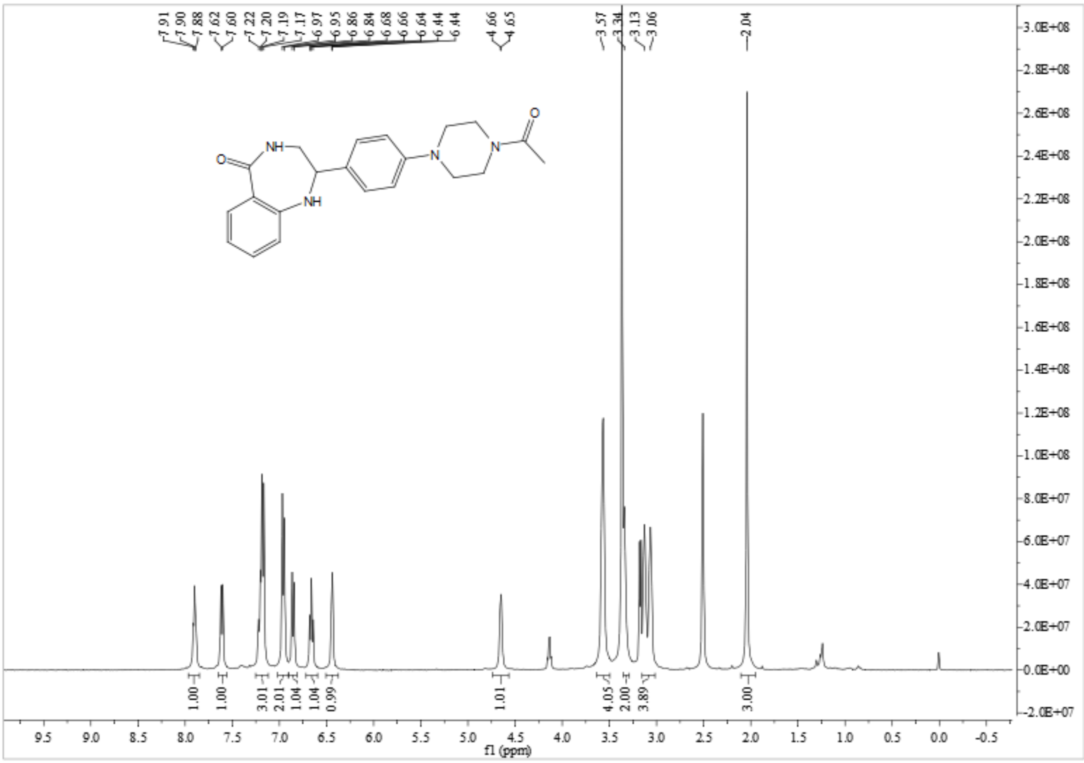

^{1}H-NMR spectrum of compound H54

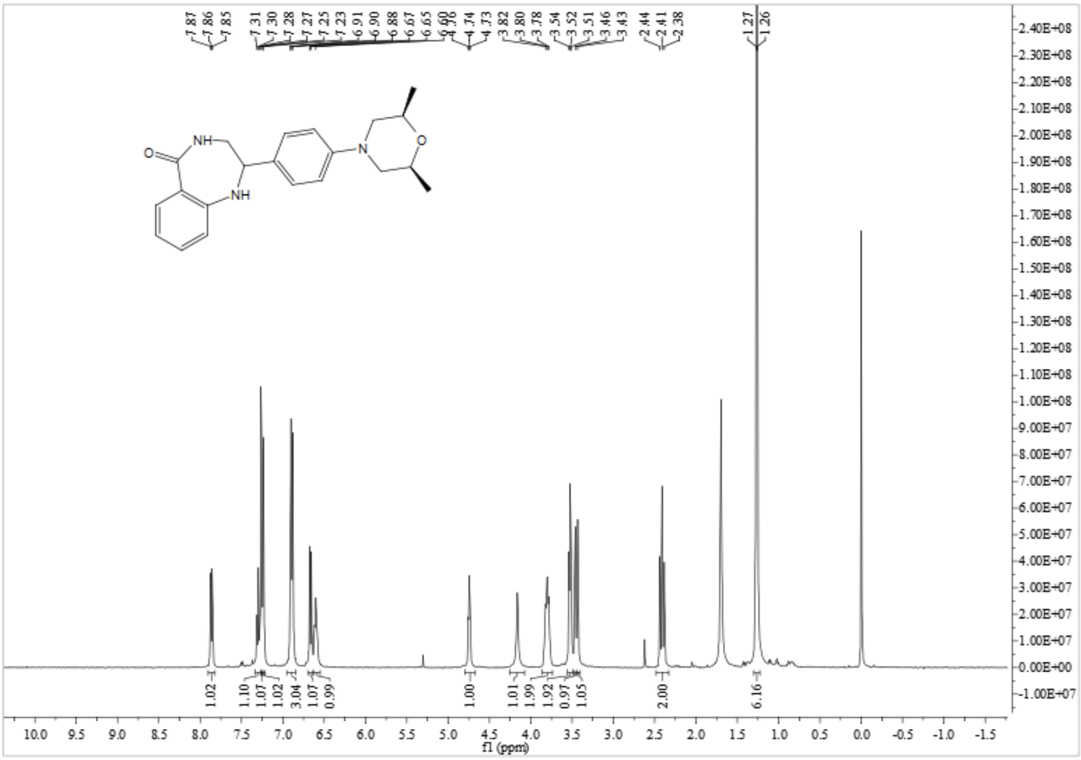

^{1}H-NMR spectrum of compound H55

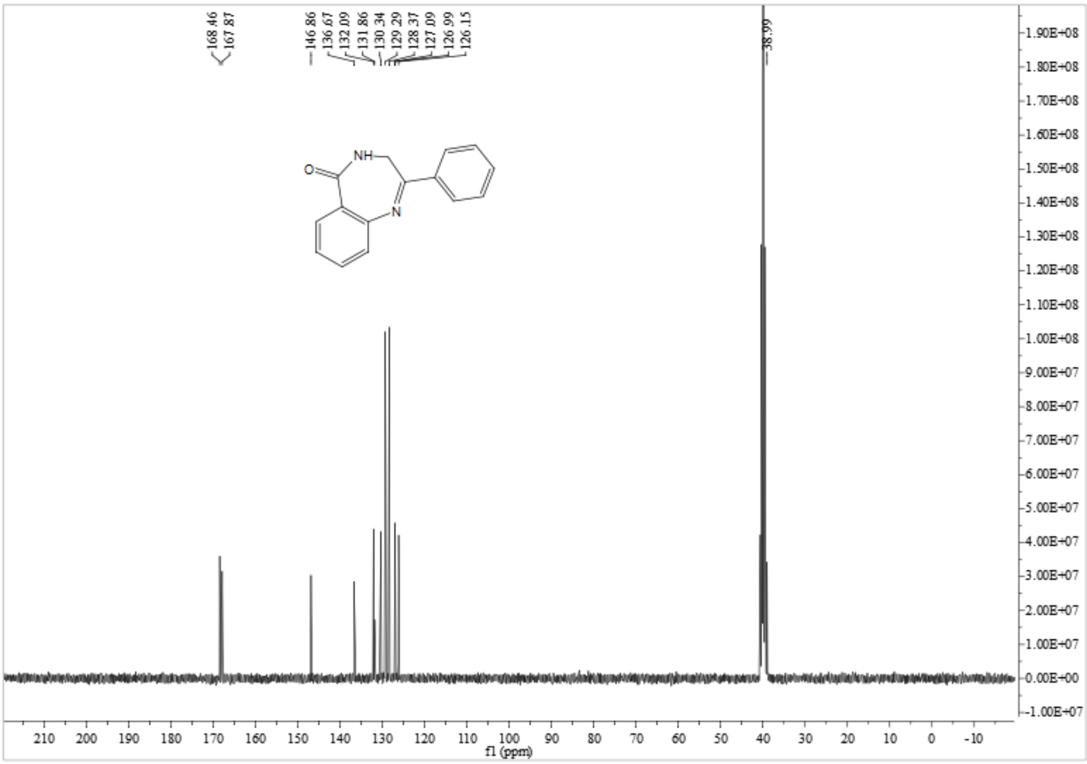

^{13}C-NMR spectrum of compound H4

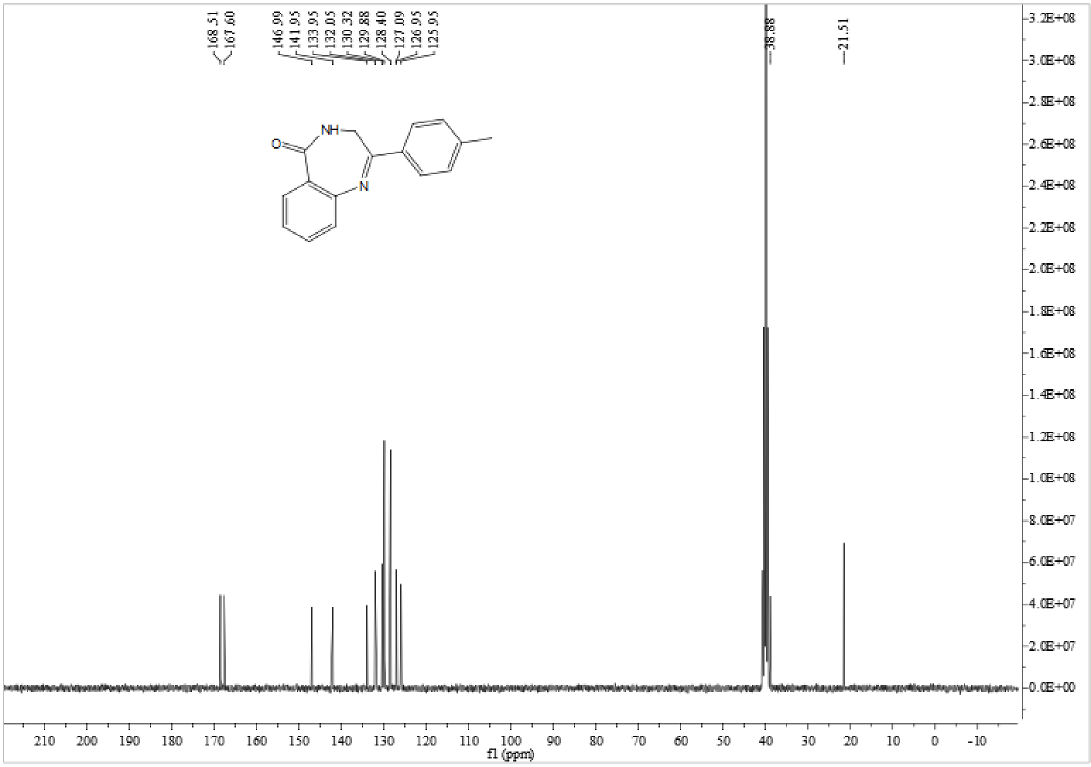

^{13}C-NMR spectrum of compound H5

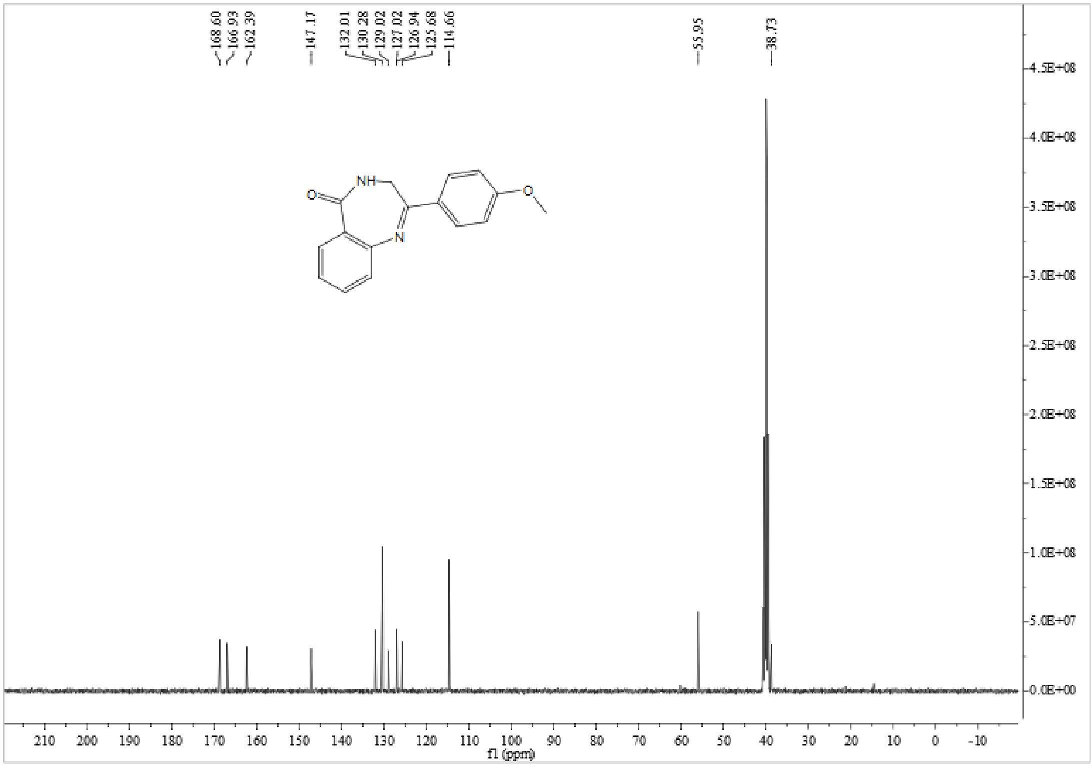

^{13}C-NMR spectrum of compound H6

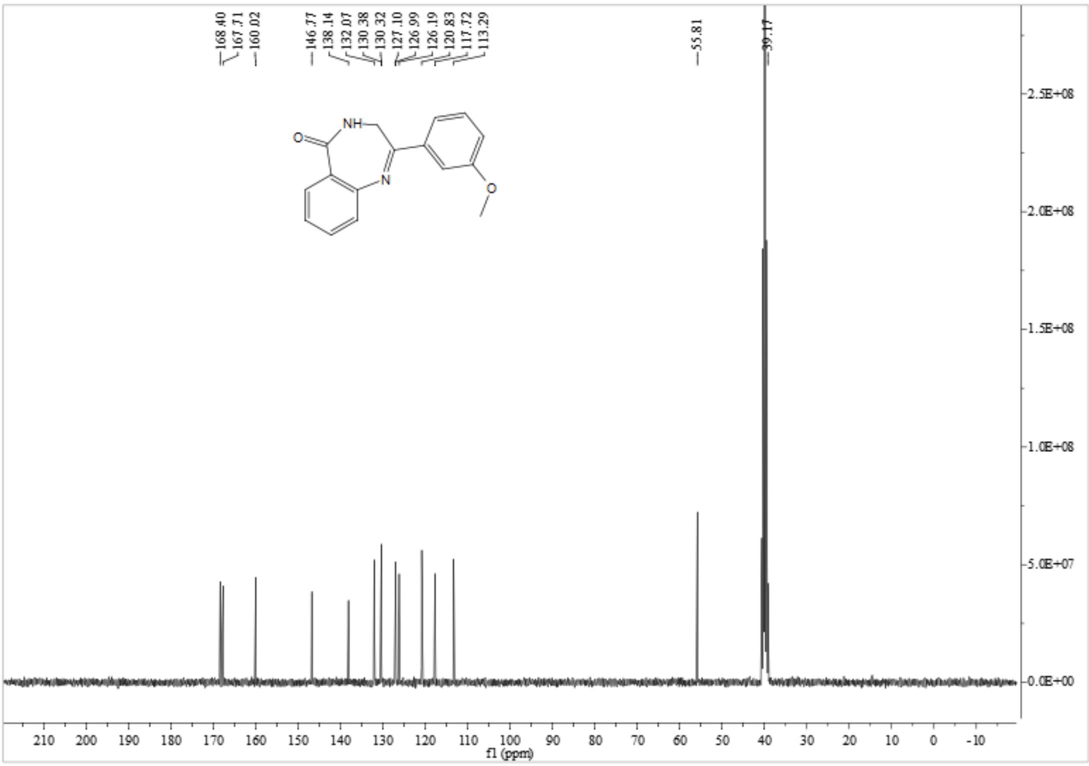

^{13}C-NMR spectrum of compound H7

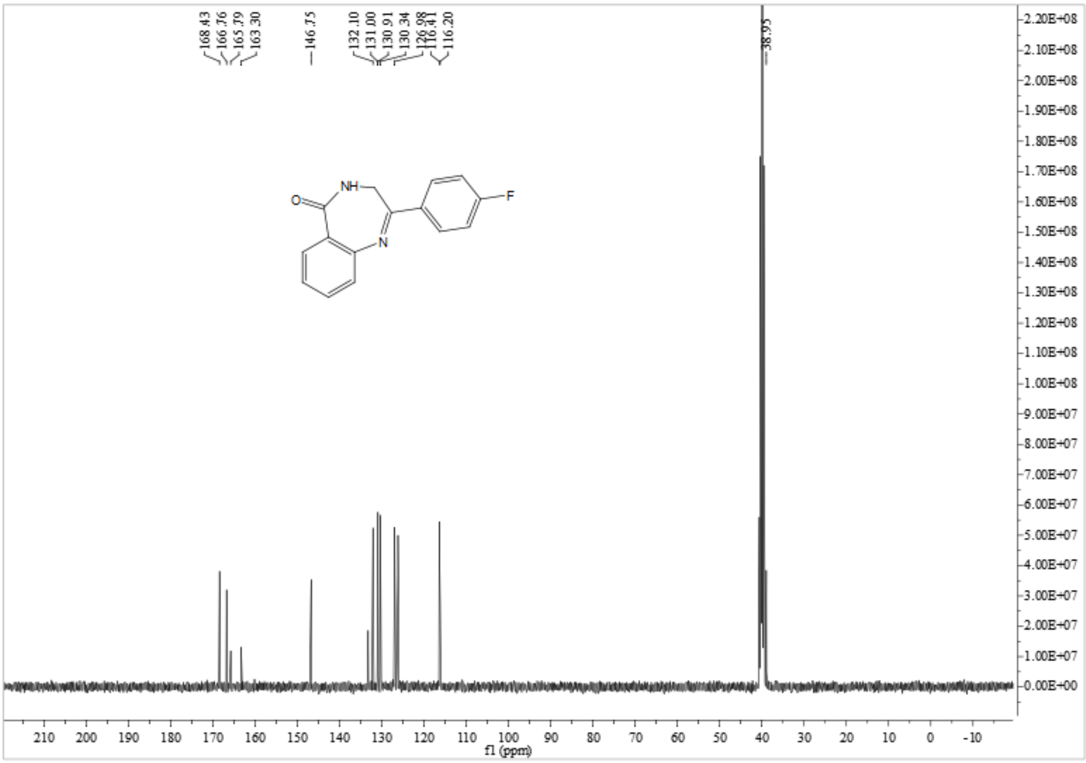

^{13}C-NMR spectrum of compound H8

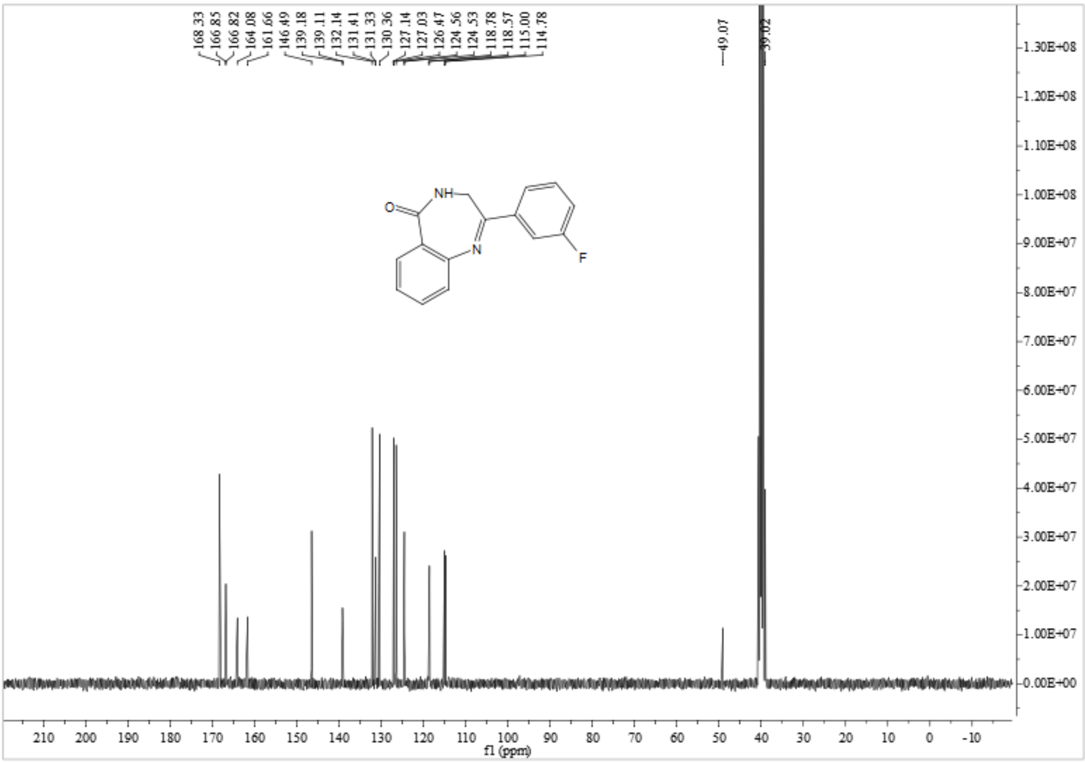

^{13}C-NMR spectrum of compound H9

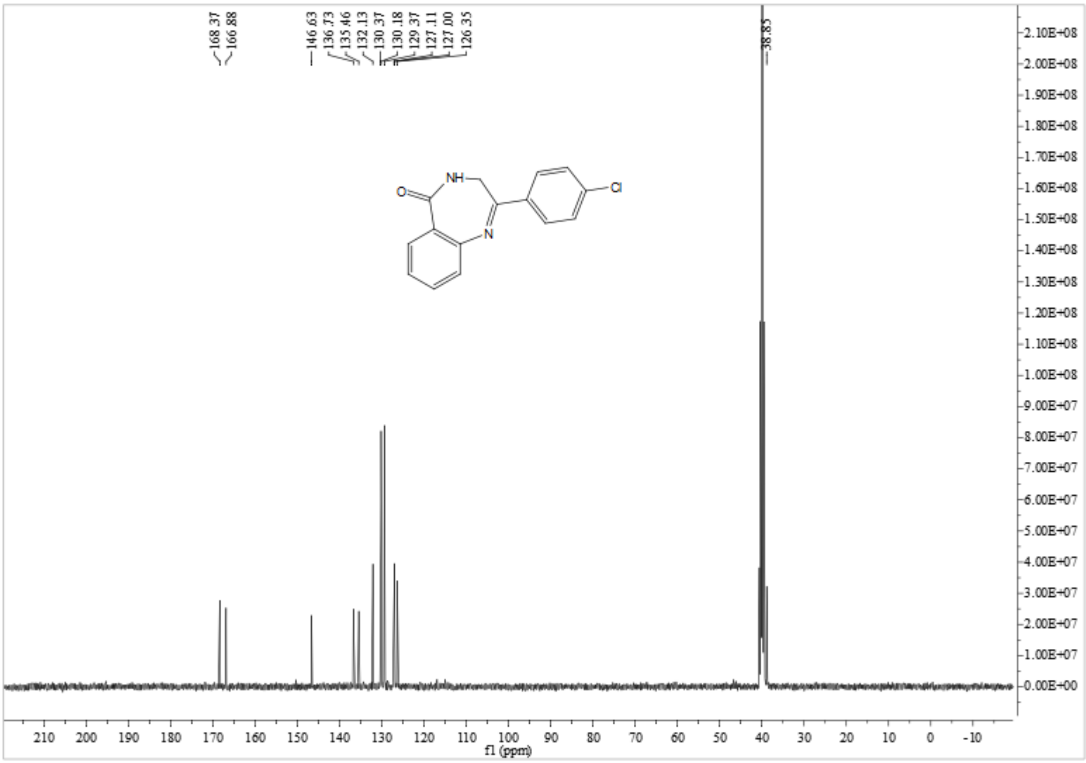

^{13}C-NMR spectrum of compound H10

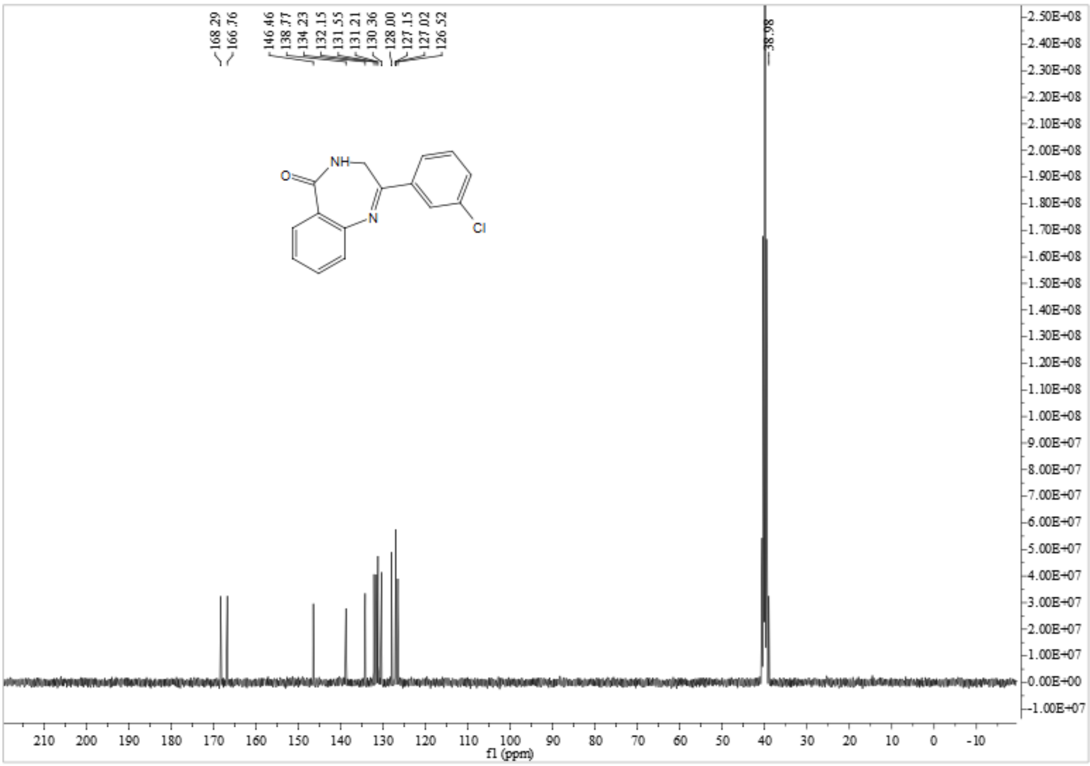

^{13}C-NMR spectrum of compound H11

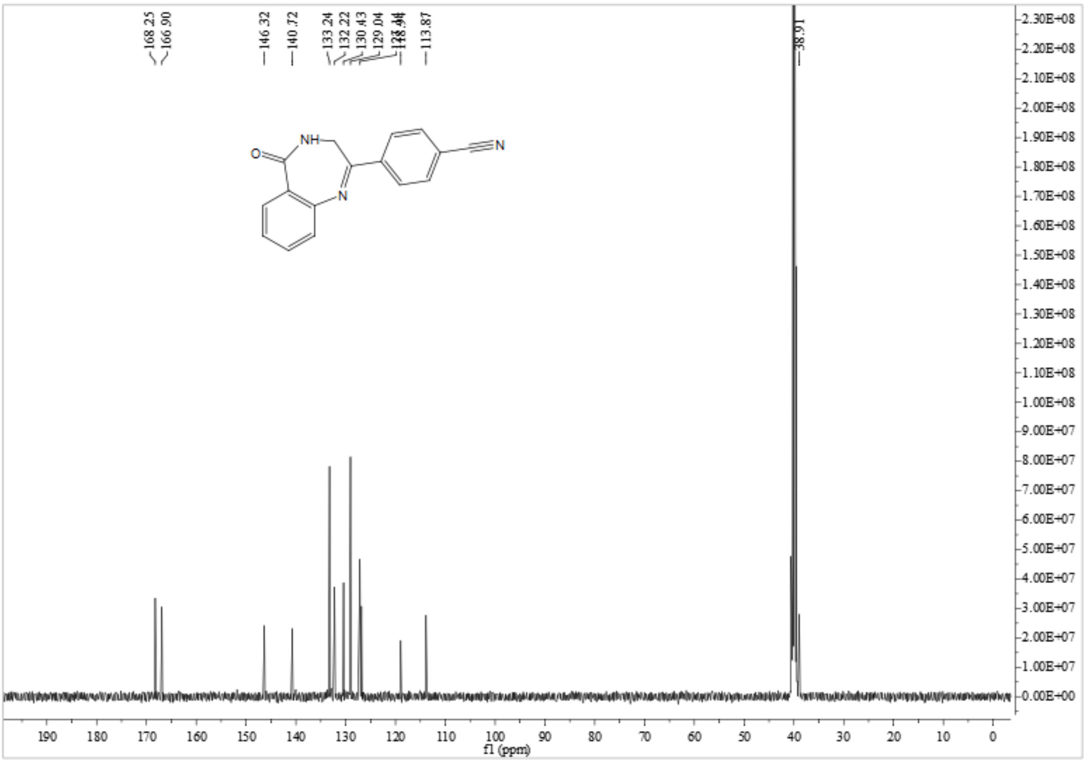

^{13}C-NMR spectrum of compound H12

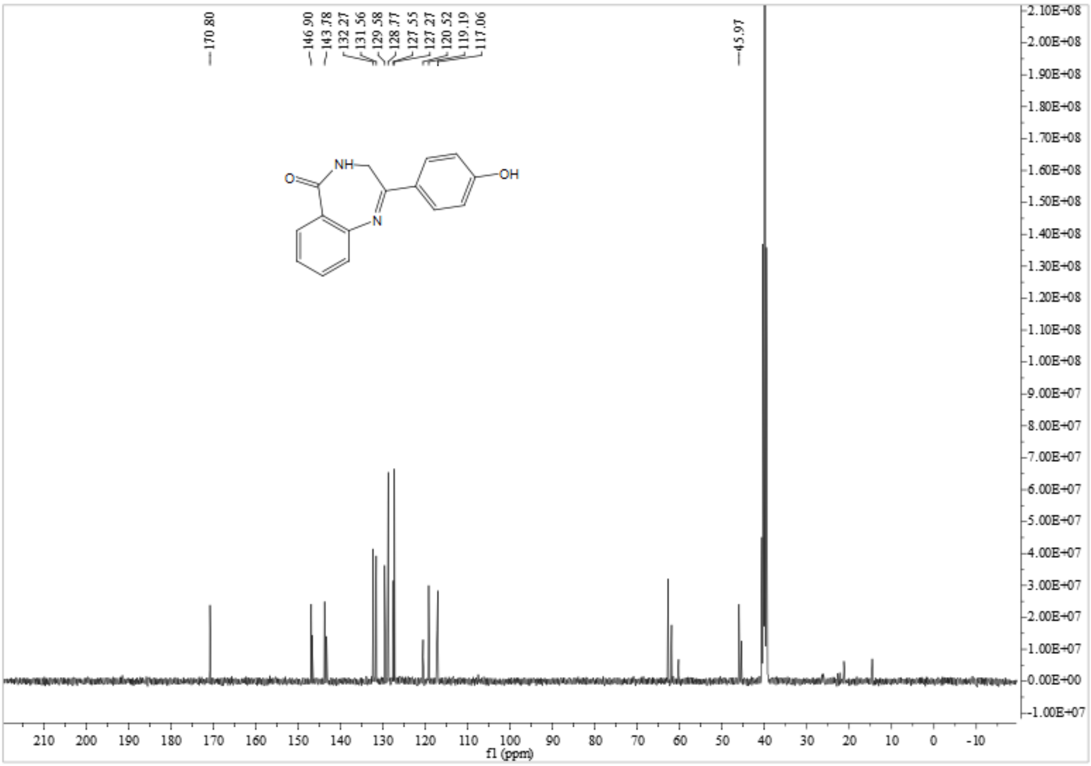

^{13}C-NMR spectrum of compound H13

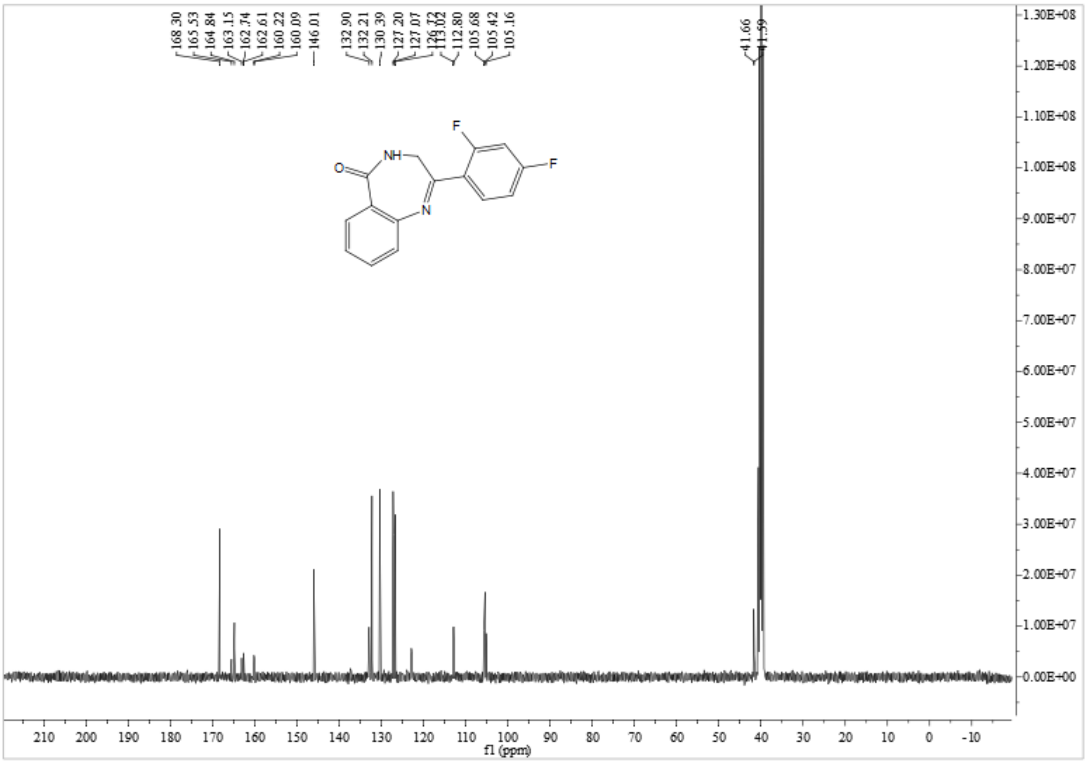

^{13}C-NMR spectrum of compound H14

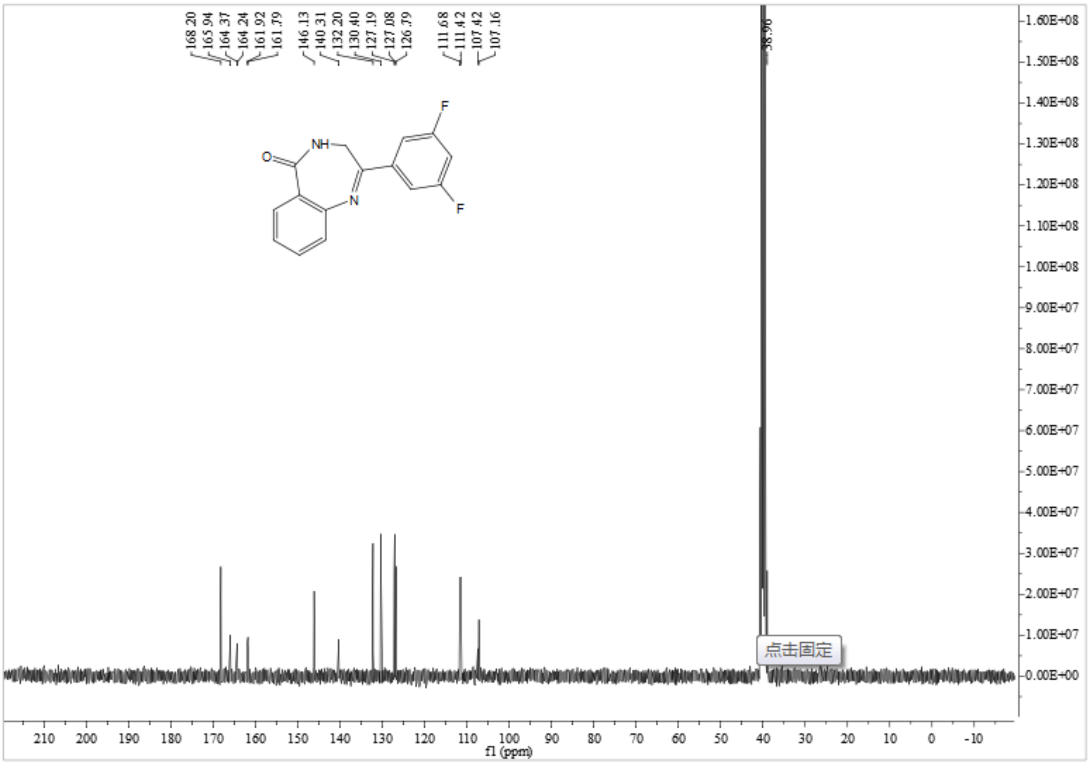

^{13}C-NMR spectrum of compound H15

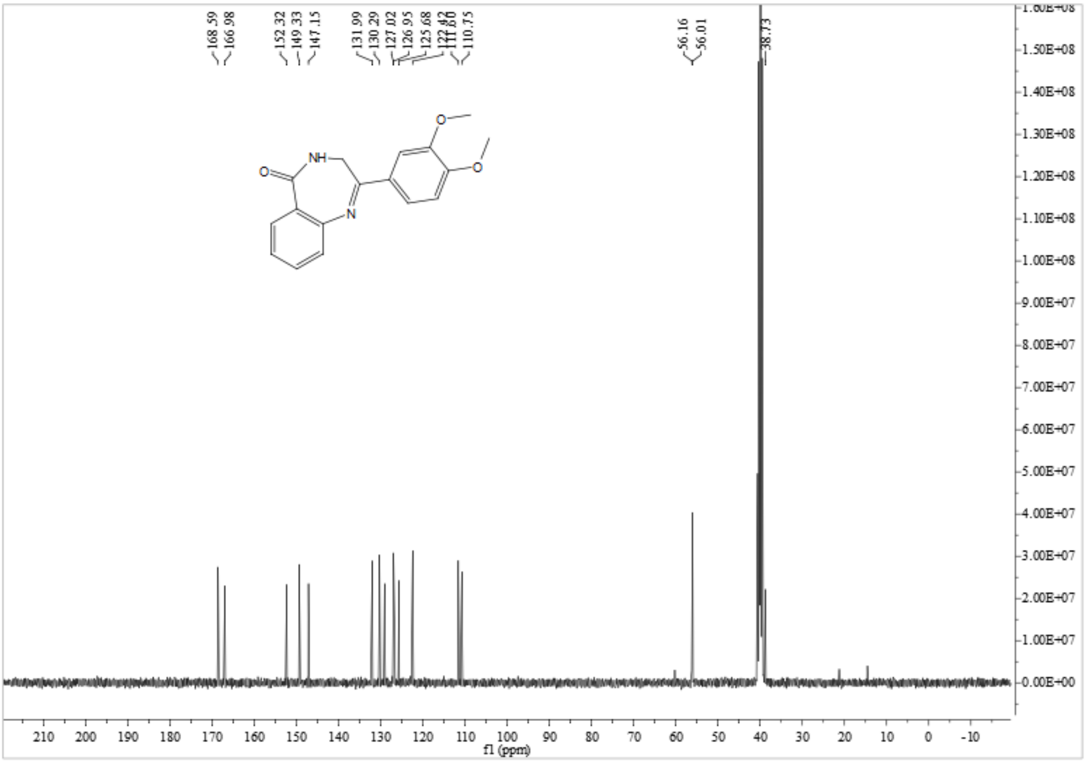

^{13}C-NMR spectrum of compound H16

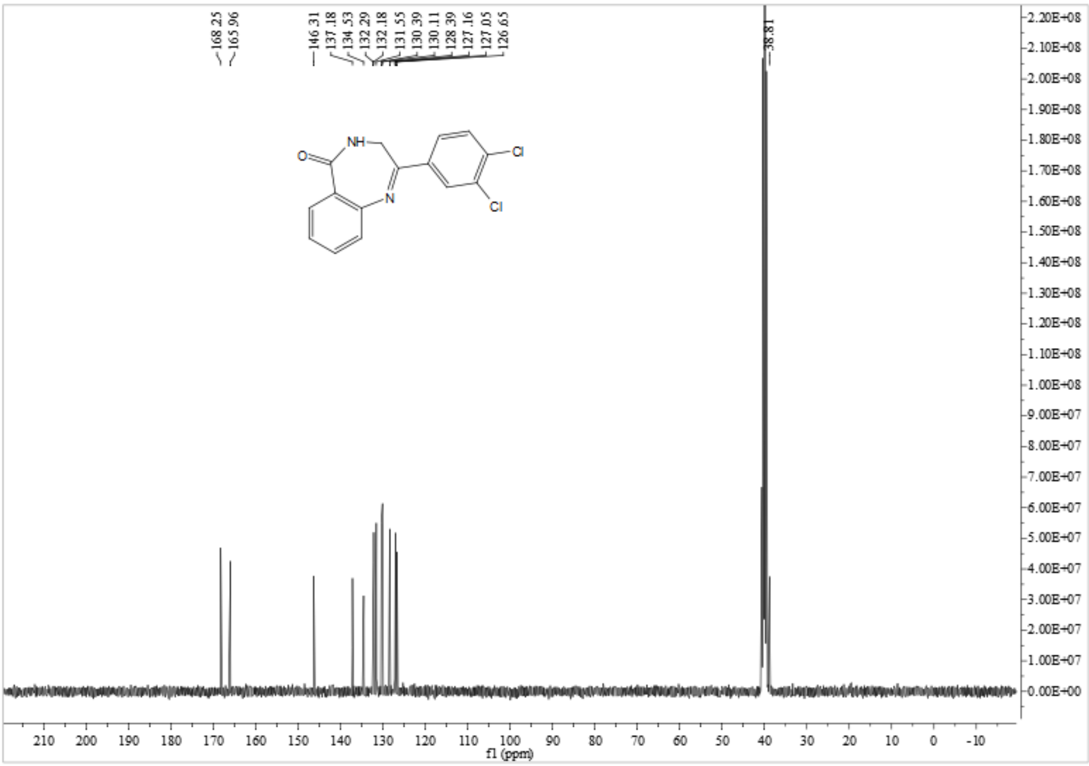

^{13}C-NMR spectrum of compound H17

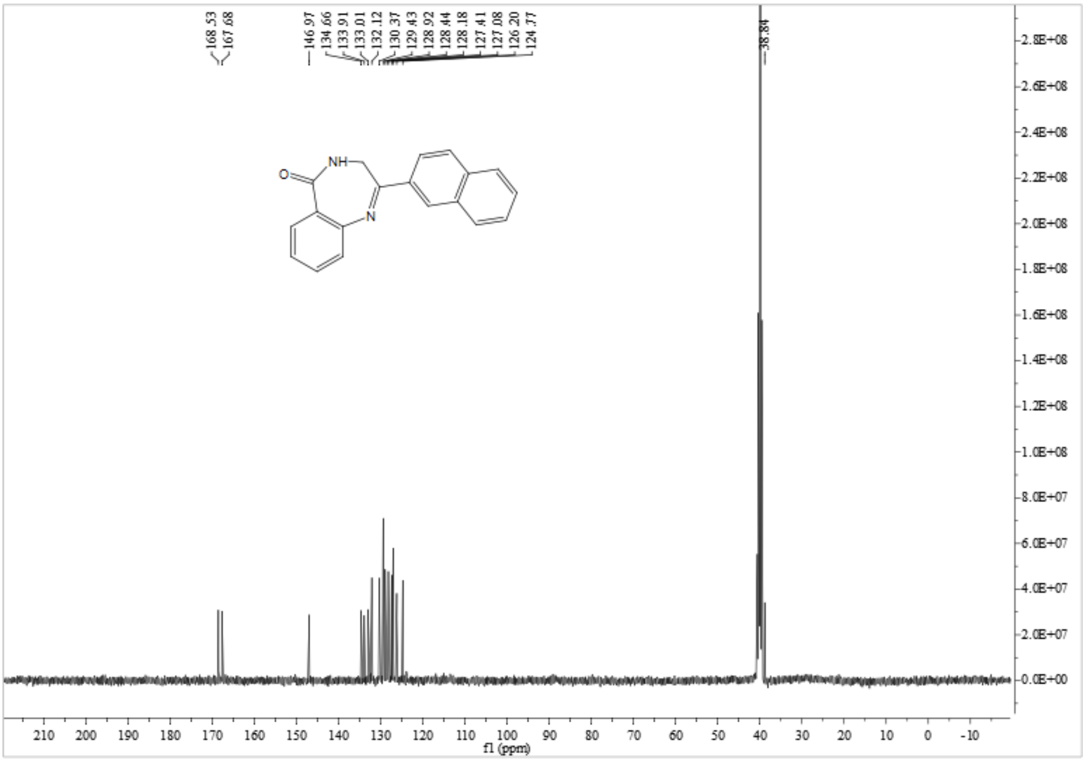

^{13}C-NMR spectrum of compound H18

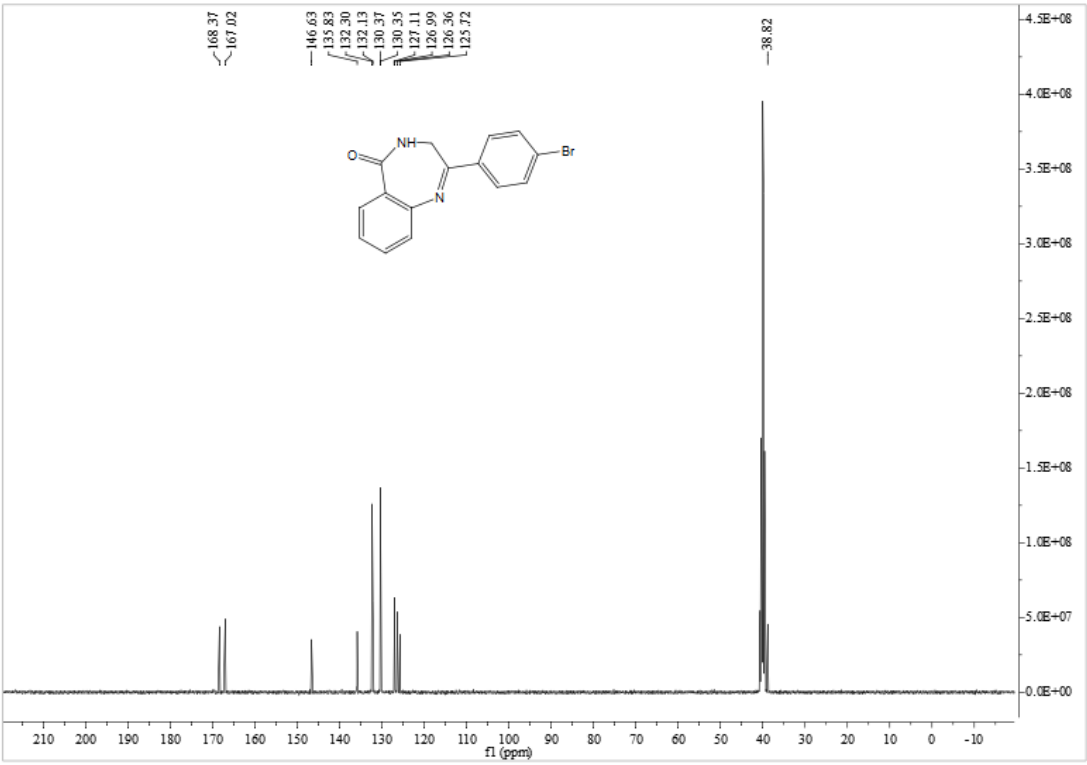

^{13}C-NMR spectrum of compound H19

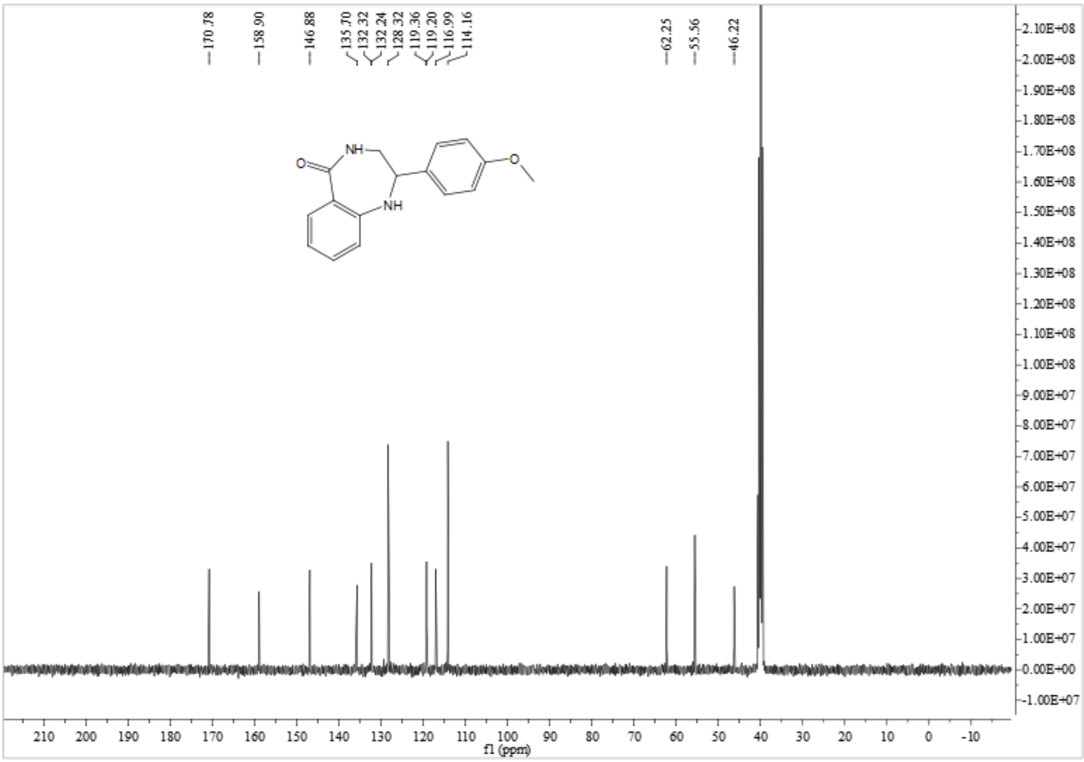

^{13}C-NMR spectrum of compound H20

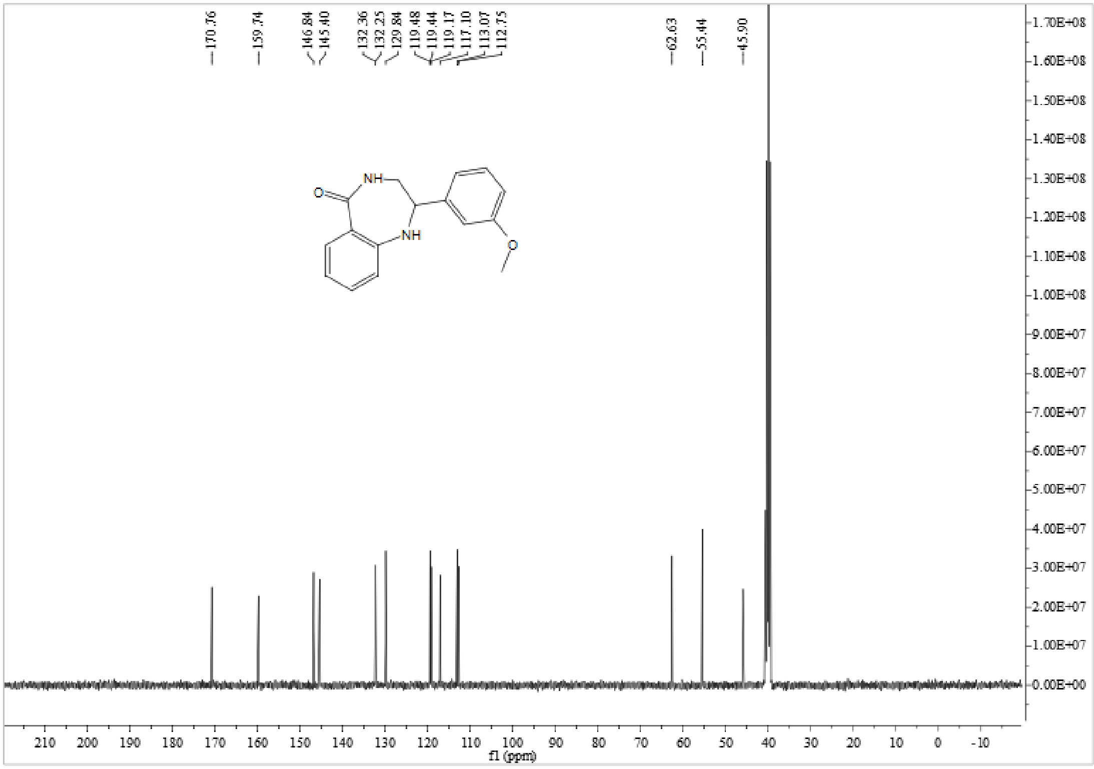

^{13}C-NMR spectrum of compound H21

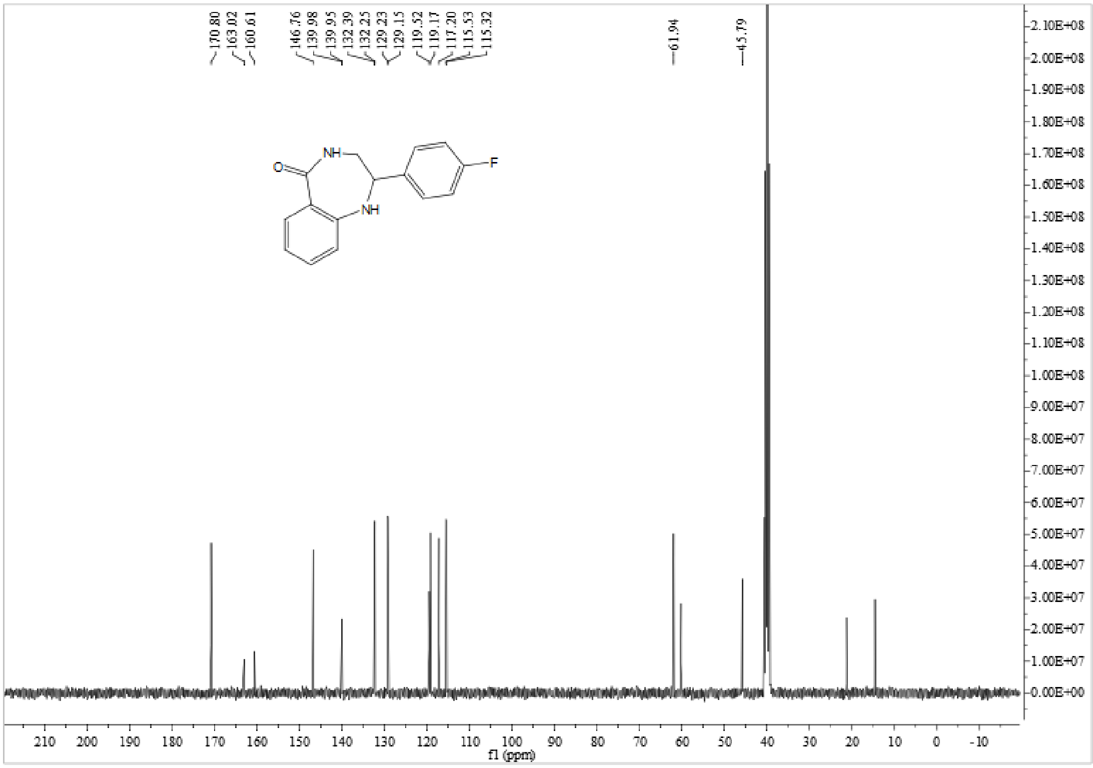

^{13}C-NMR spectrum of compound H22

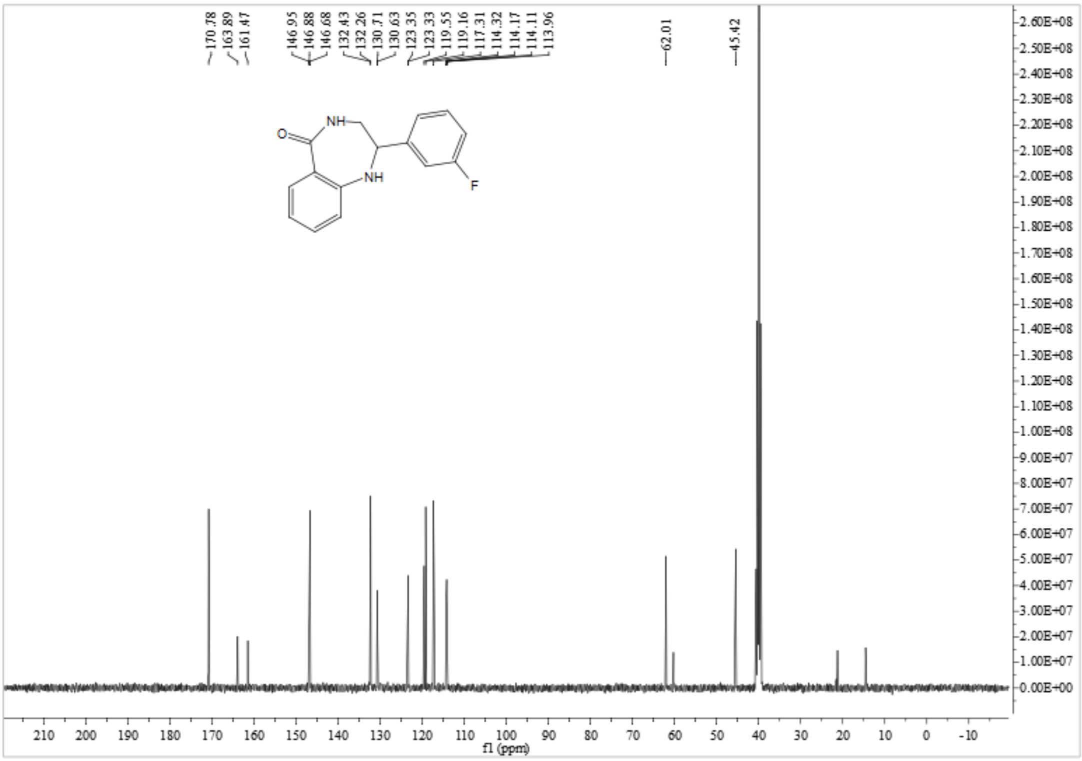

^{13}C-NMR spectrum of compound H23

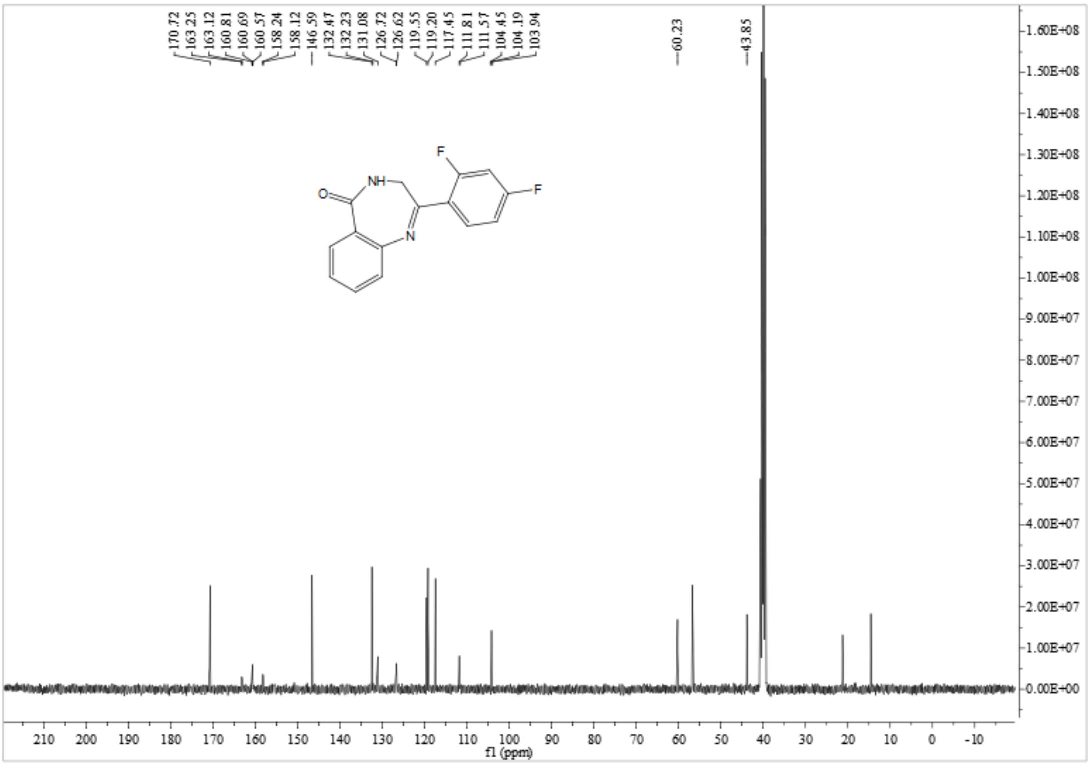

^{13}C-NMR spectrum of compound H24

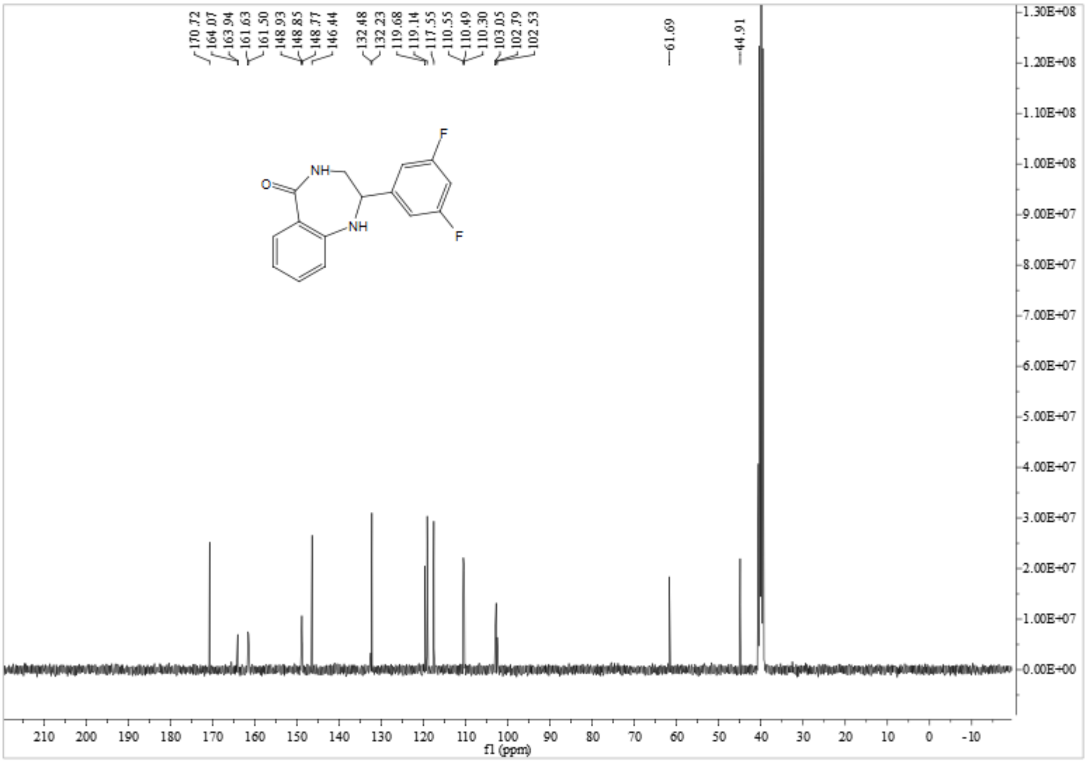

^{13}C-NMR spectrum of compound H25

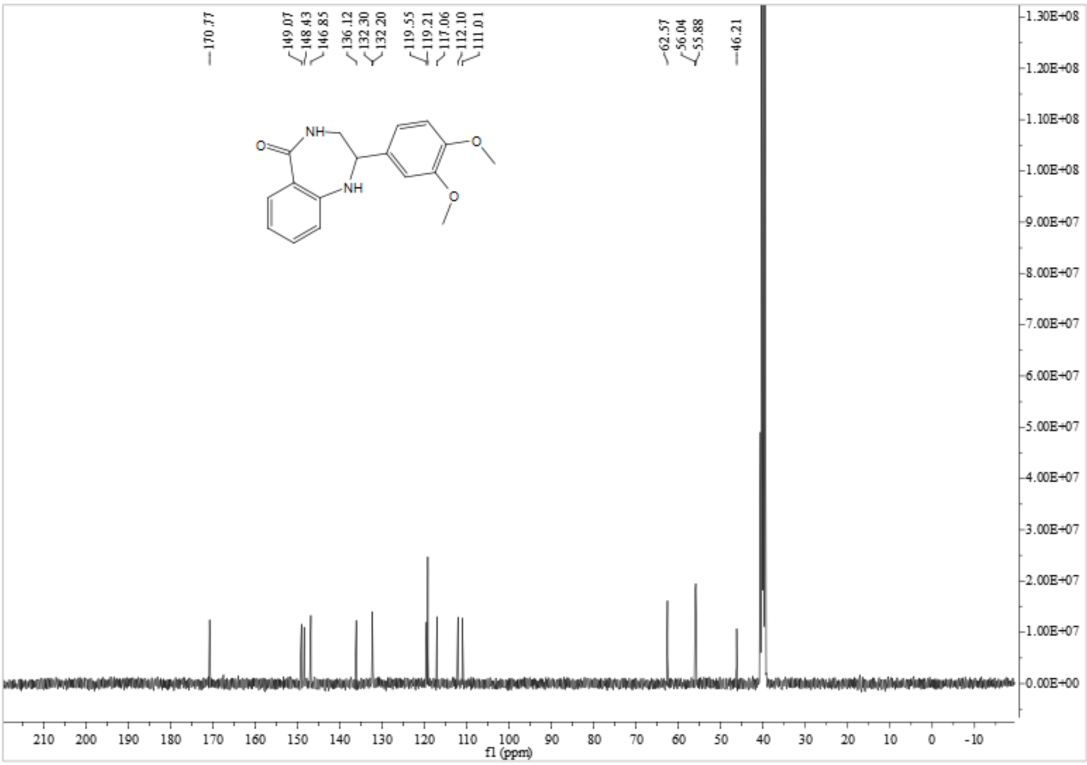

^{13}C-NMR spectrum of compound H26

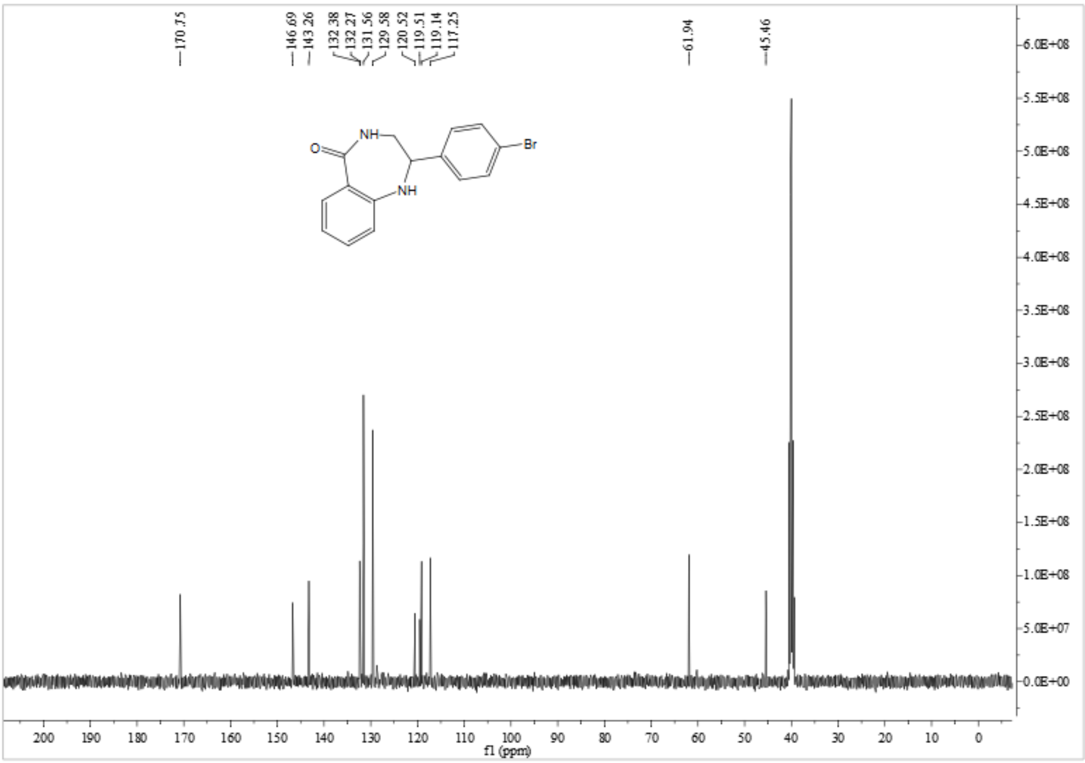

^{13}C-NMR spectrum of compound H27

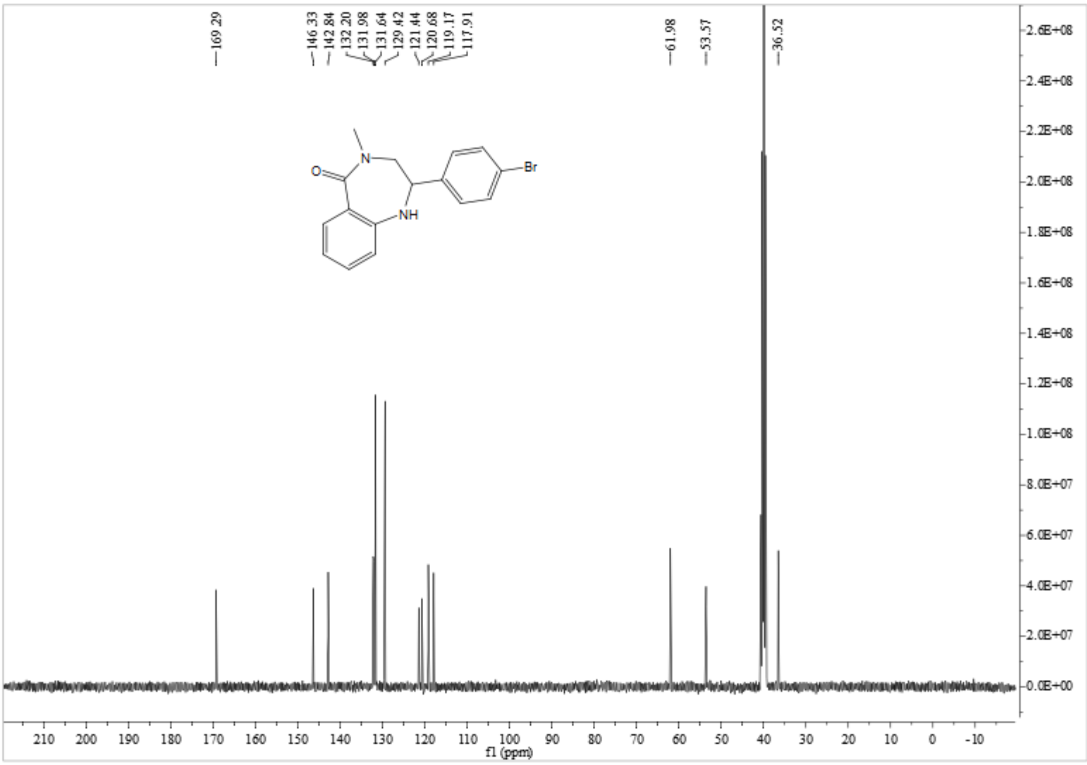

^{13}C-NMR spectrum of compound H28

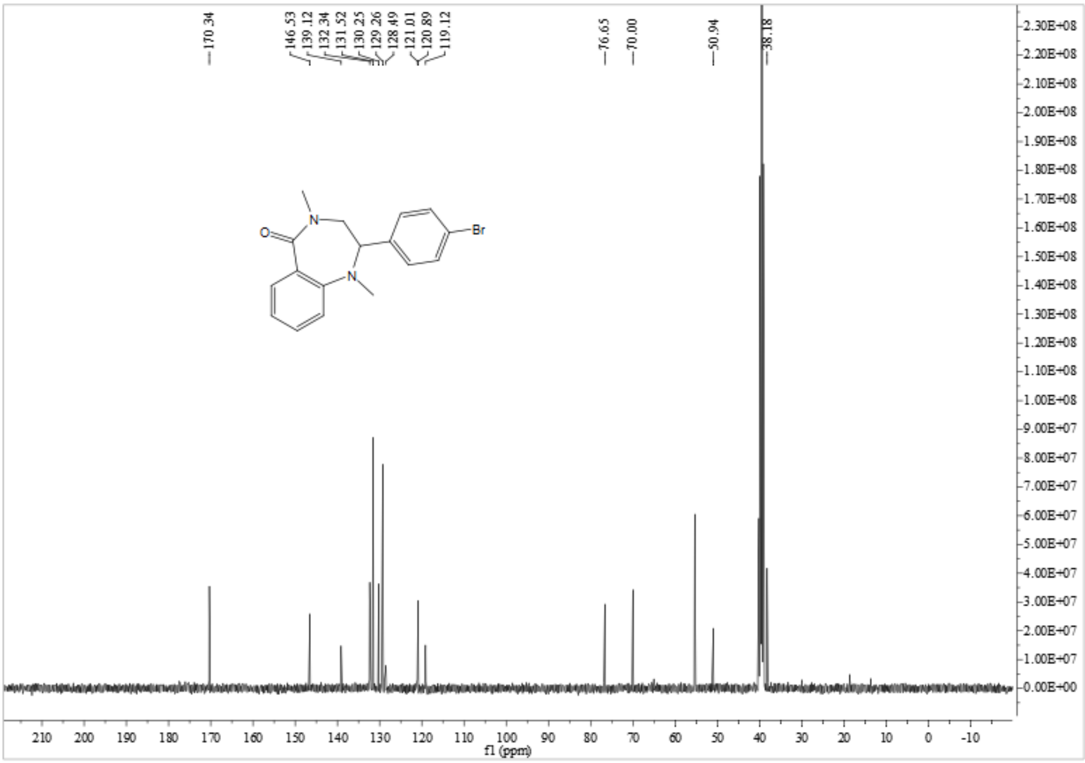

^{13}C-NMR spectrum of compound H29

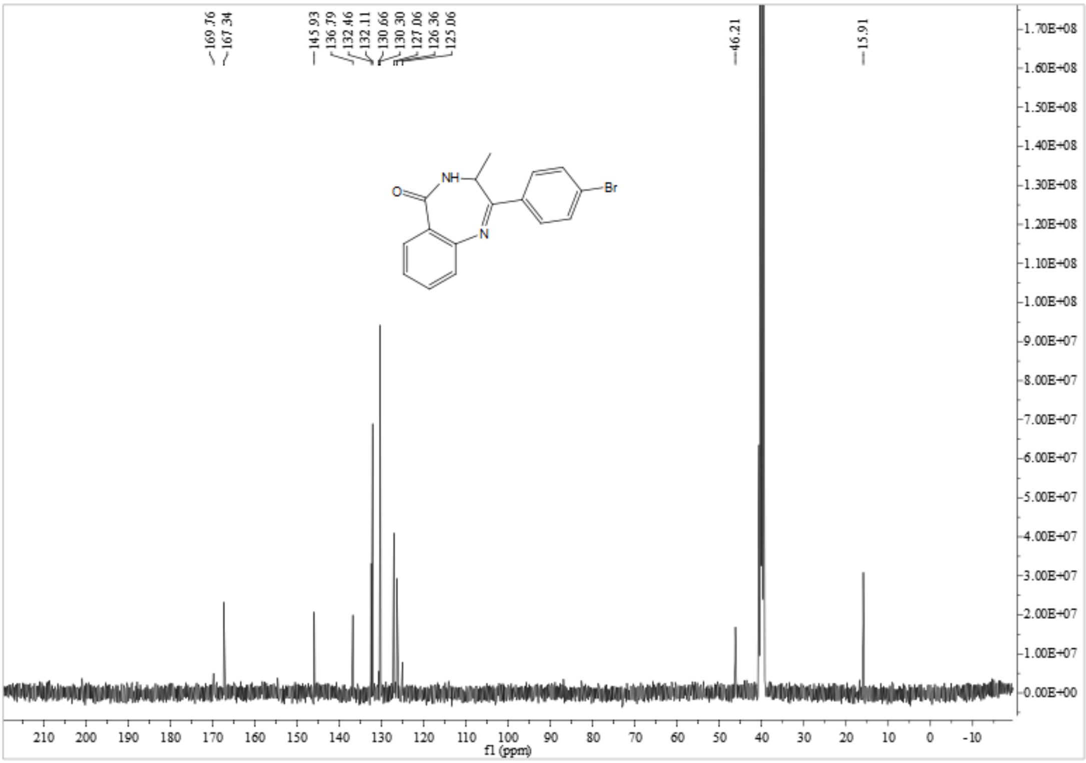

^{13}C-NMR spectrum of compound H30

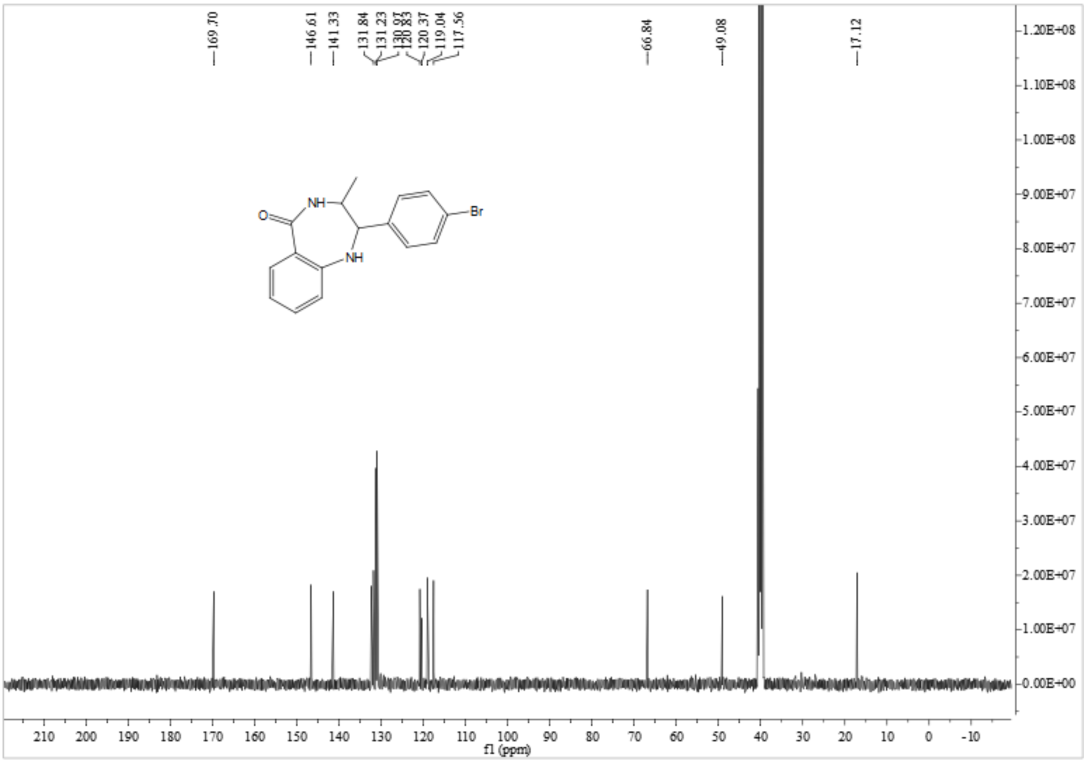

^{13}C-NMR spectrum of compound H31

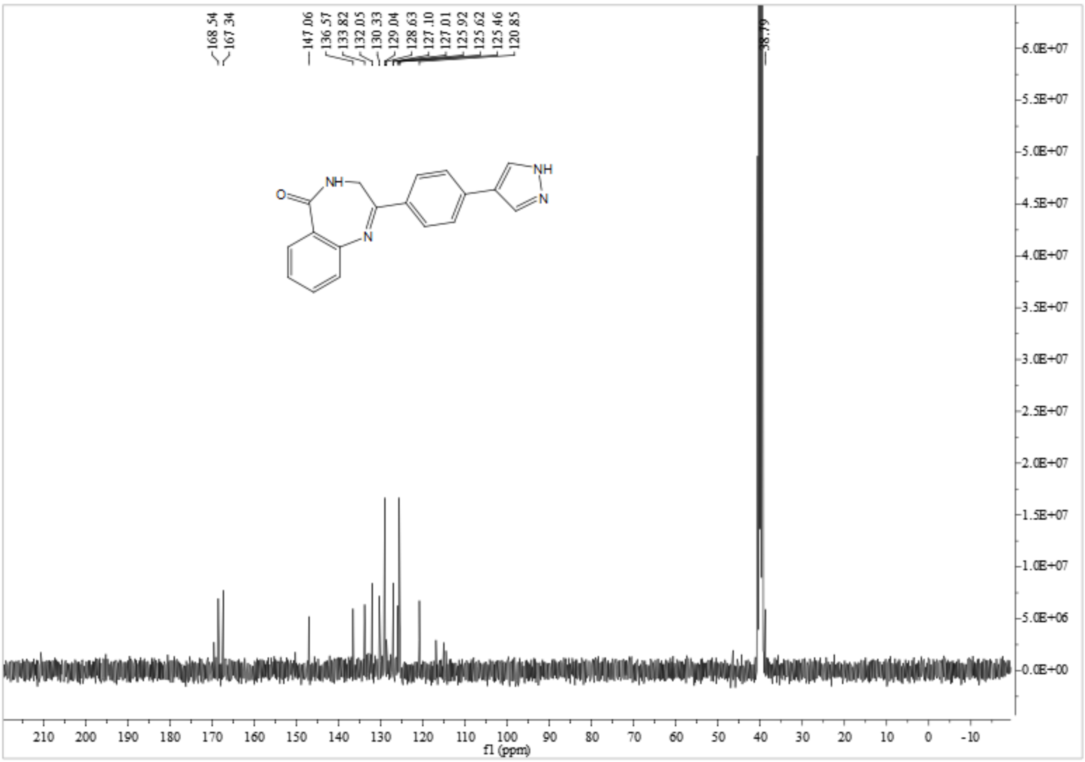

^{13}C-NMR spectrum of compound H32

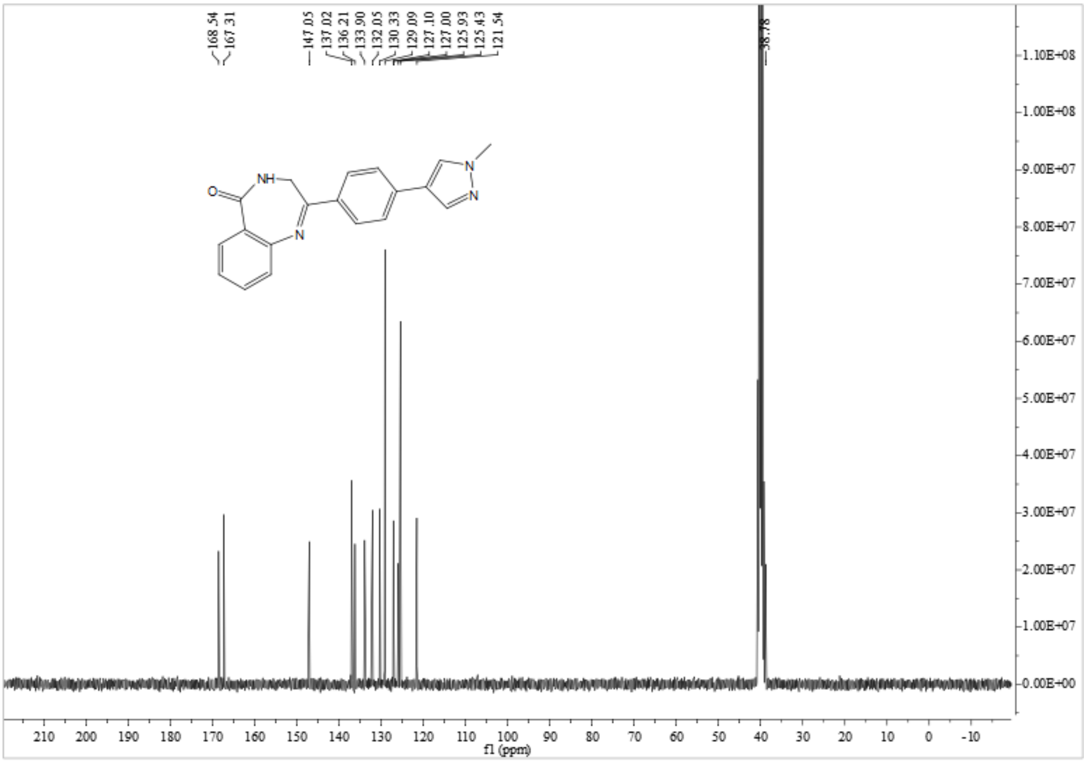

^{13}C-NMR spectrum of compound H33

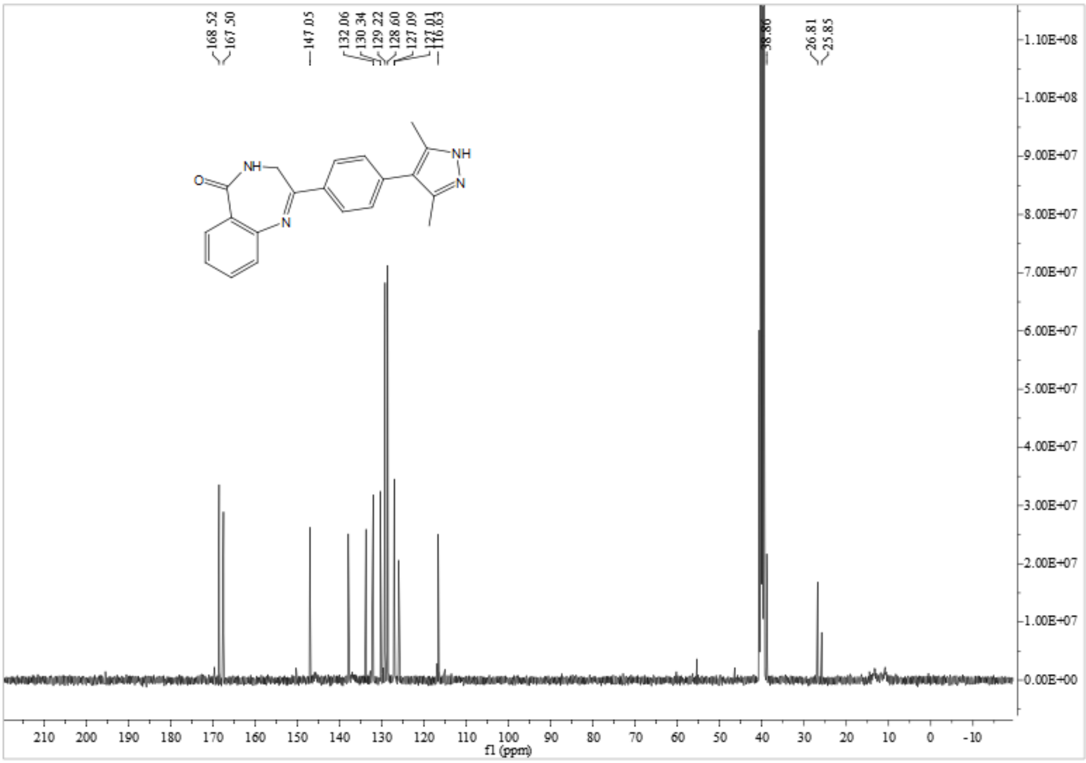

^{13}C-NMR spectrum of compound H34

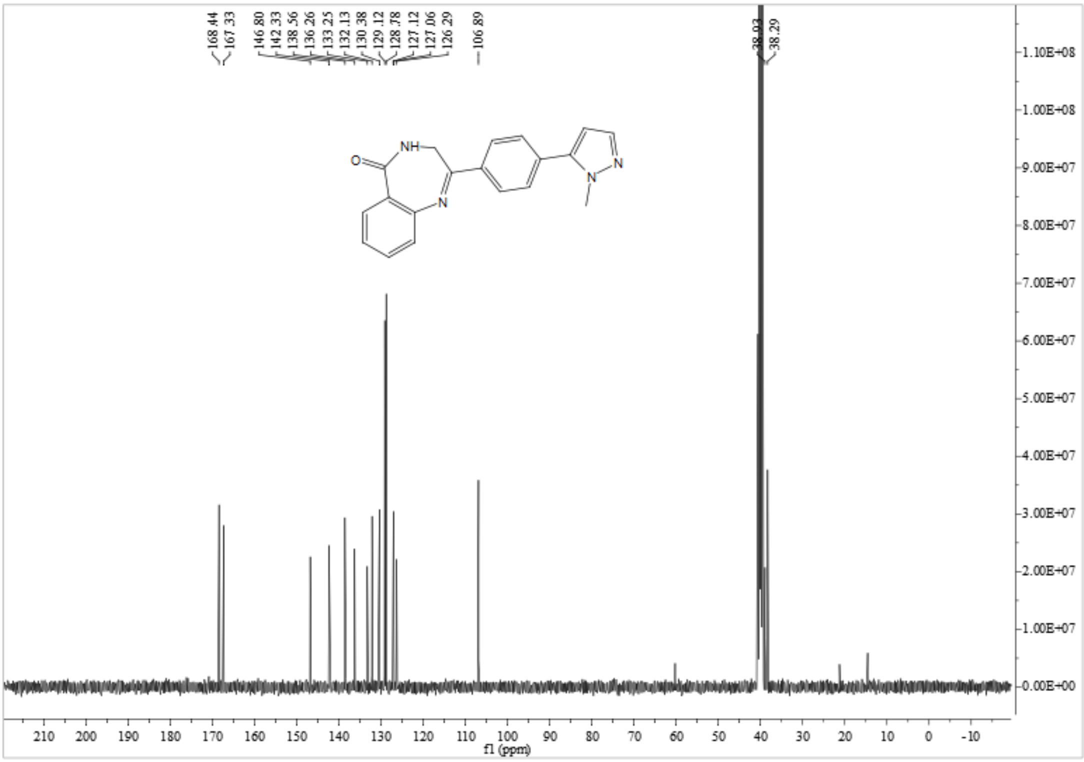

^{13}C-NMR spectrum of compound H35

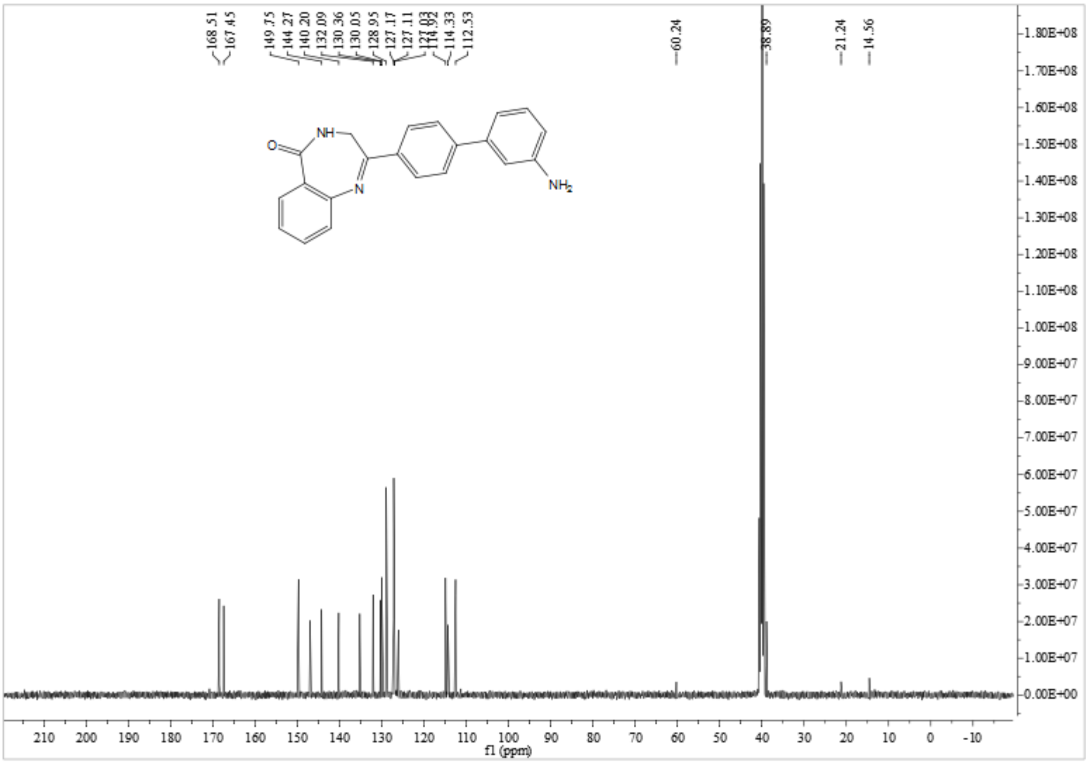

^{13}C-NMR spectrum of compound H36

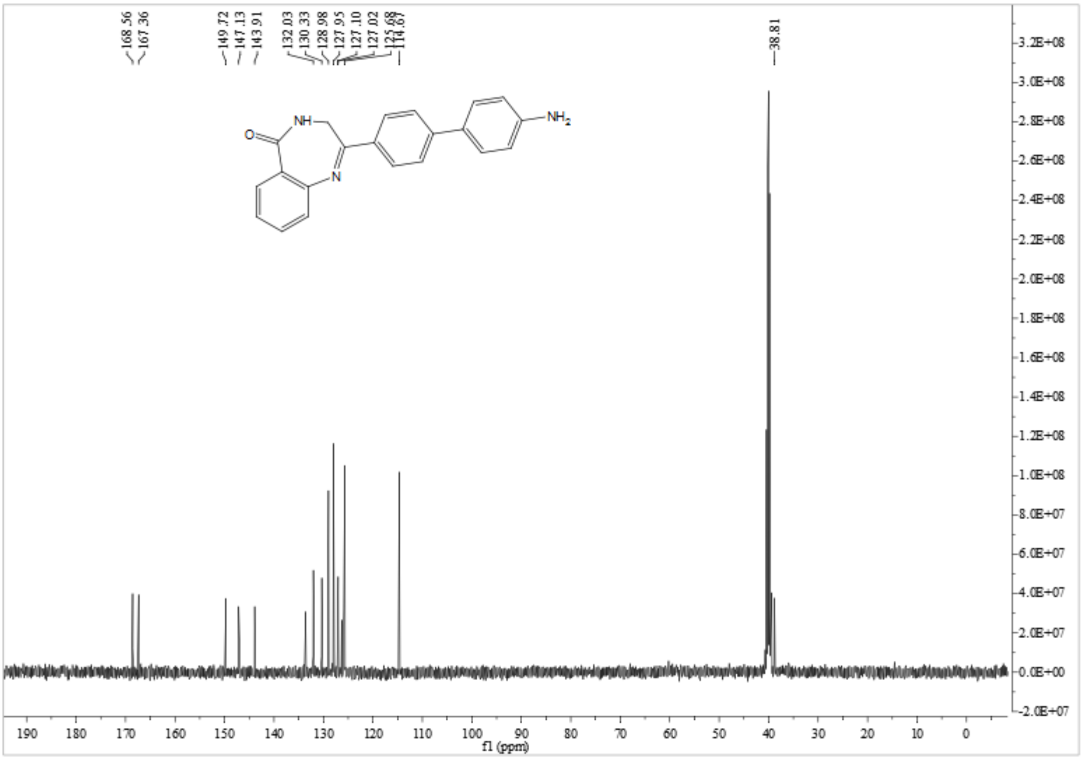

^{13}C-NMR spectrum of compound H37

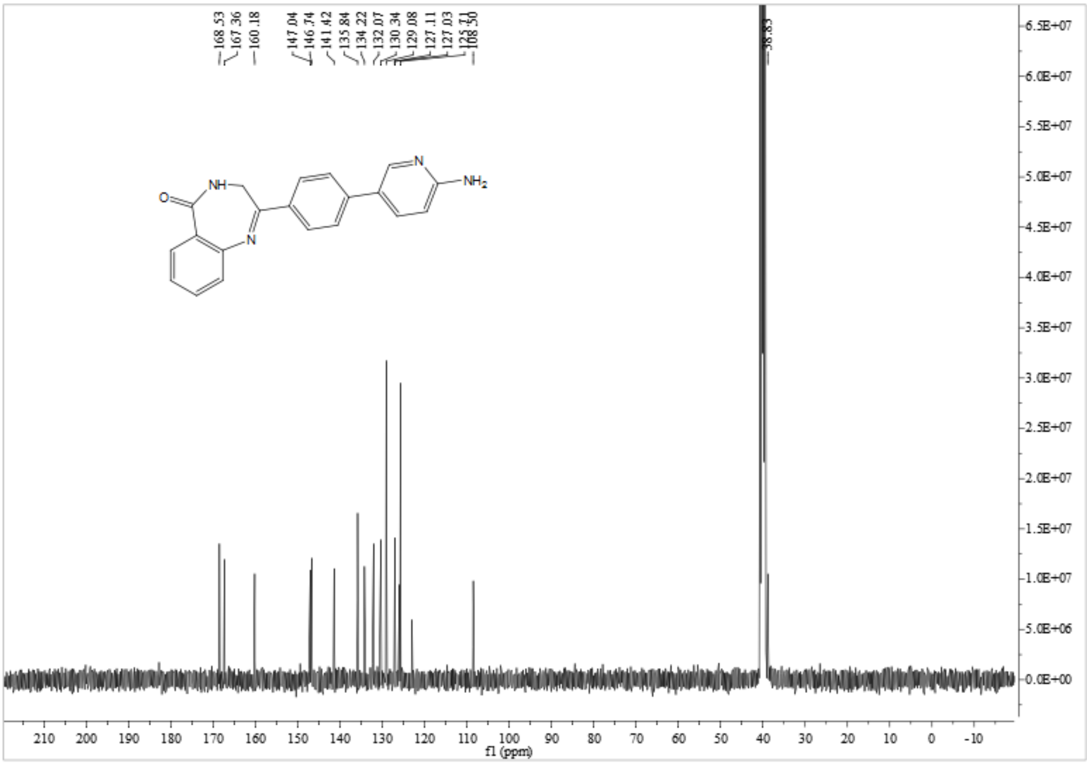

^{13}C-NMR spectrum of compound H38

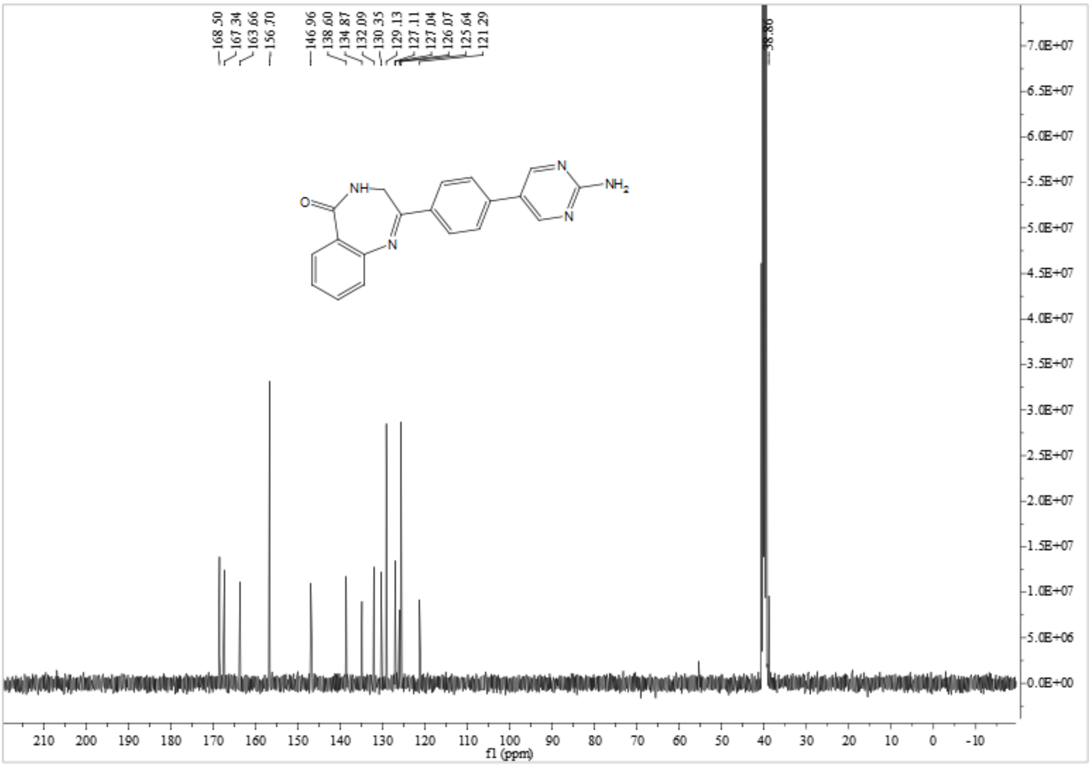

^{13}C-NMR spectrum of compound H39

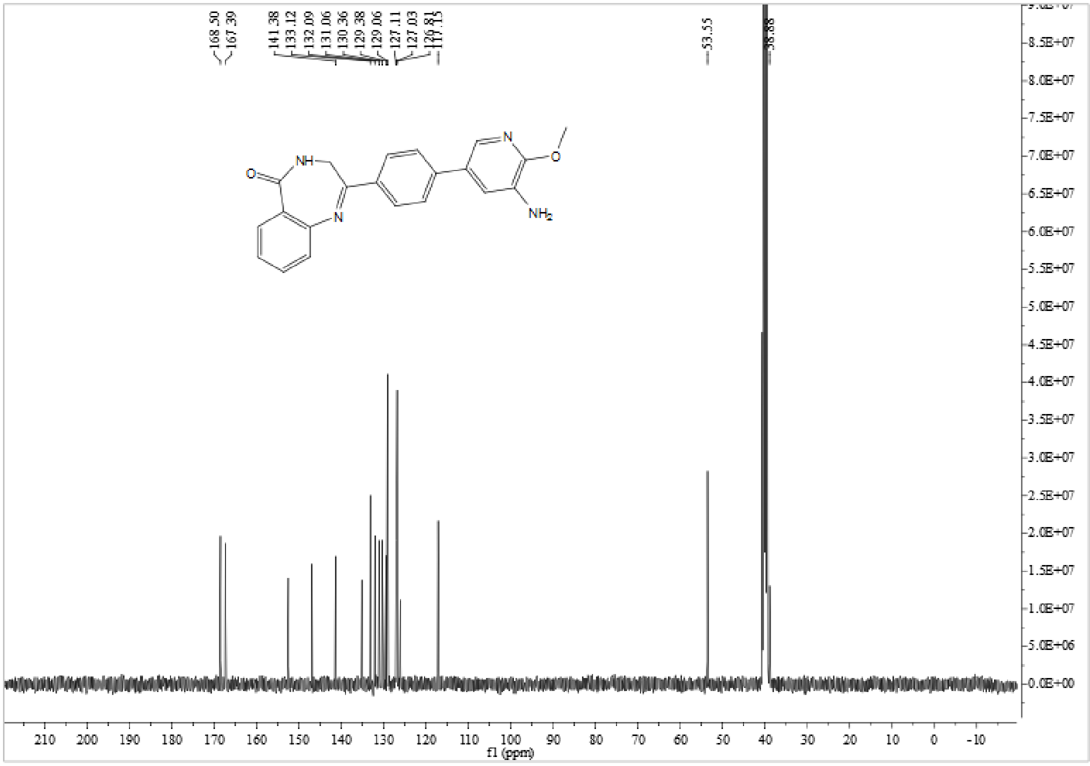

^{13}C-NMR spectrum of compound H40

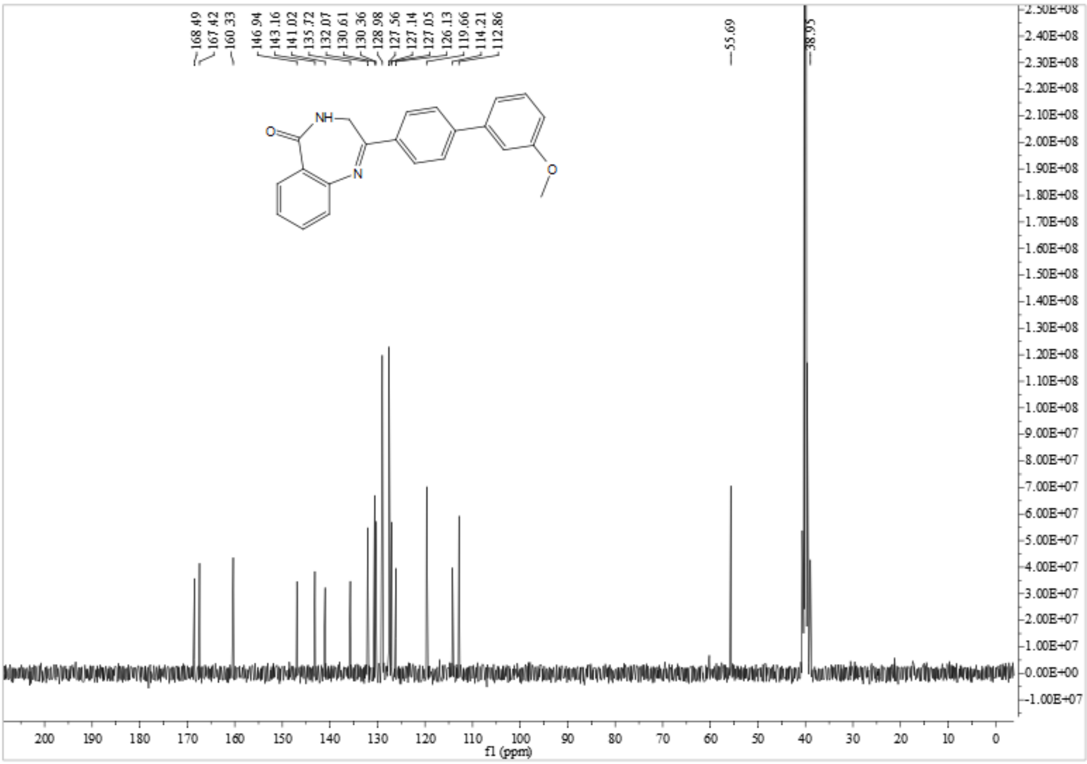

^{13}C-NMR spectrum of compound H41

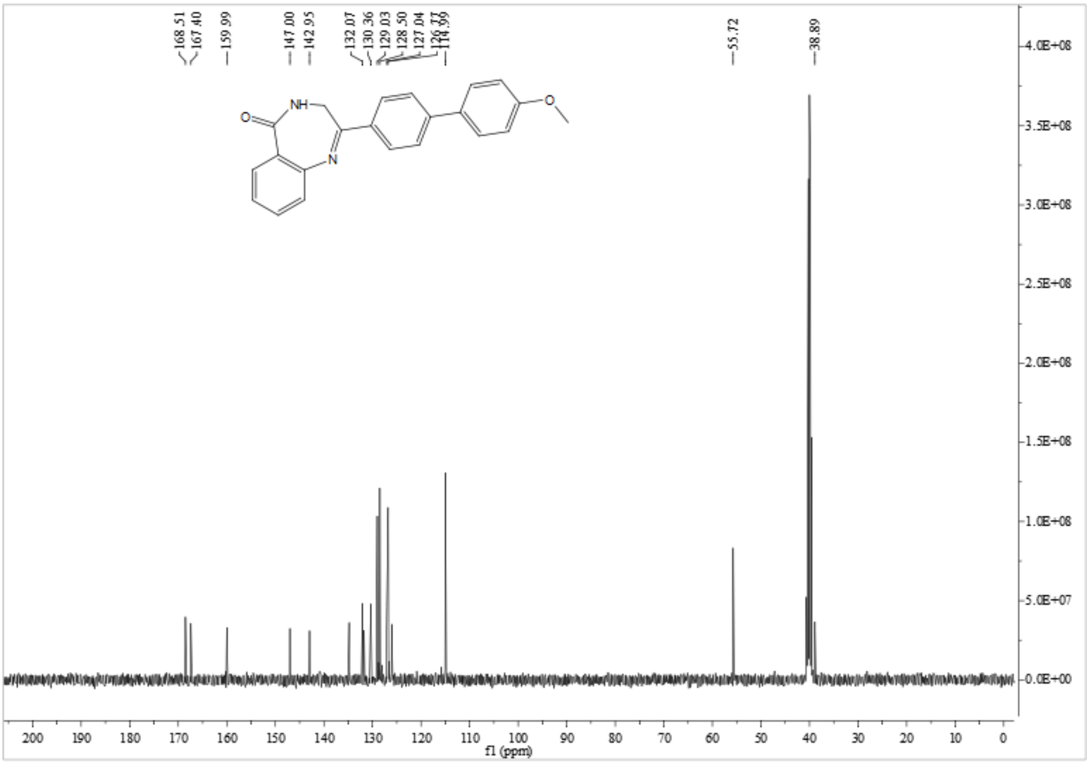

^{13}C-NMR spectrum of compound H42

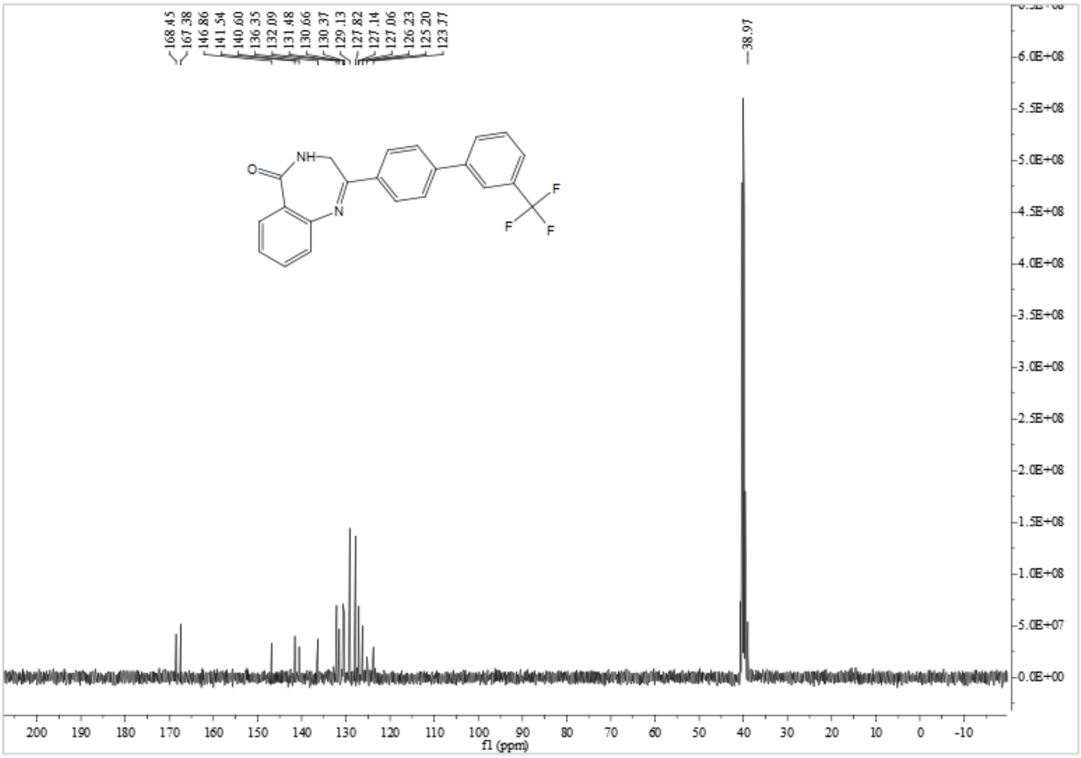

^{13}C-NMR spectrum of compound H43

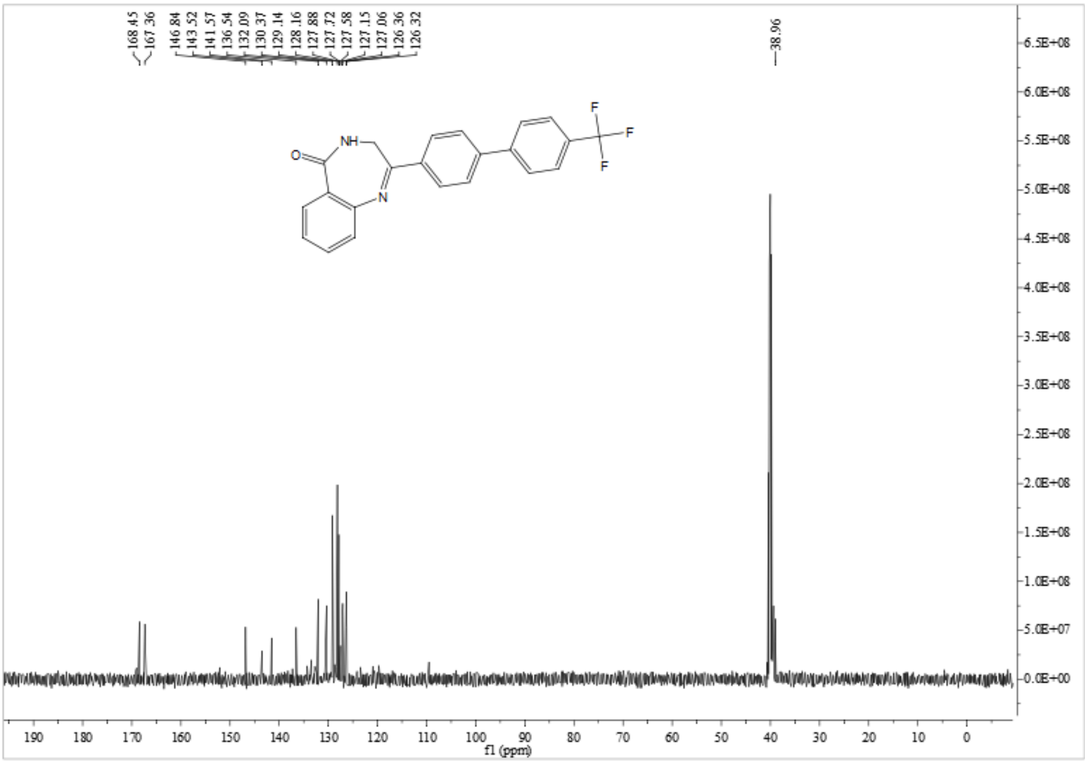

^{13}C-NMR spectrum of compound H44

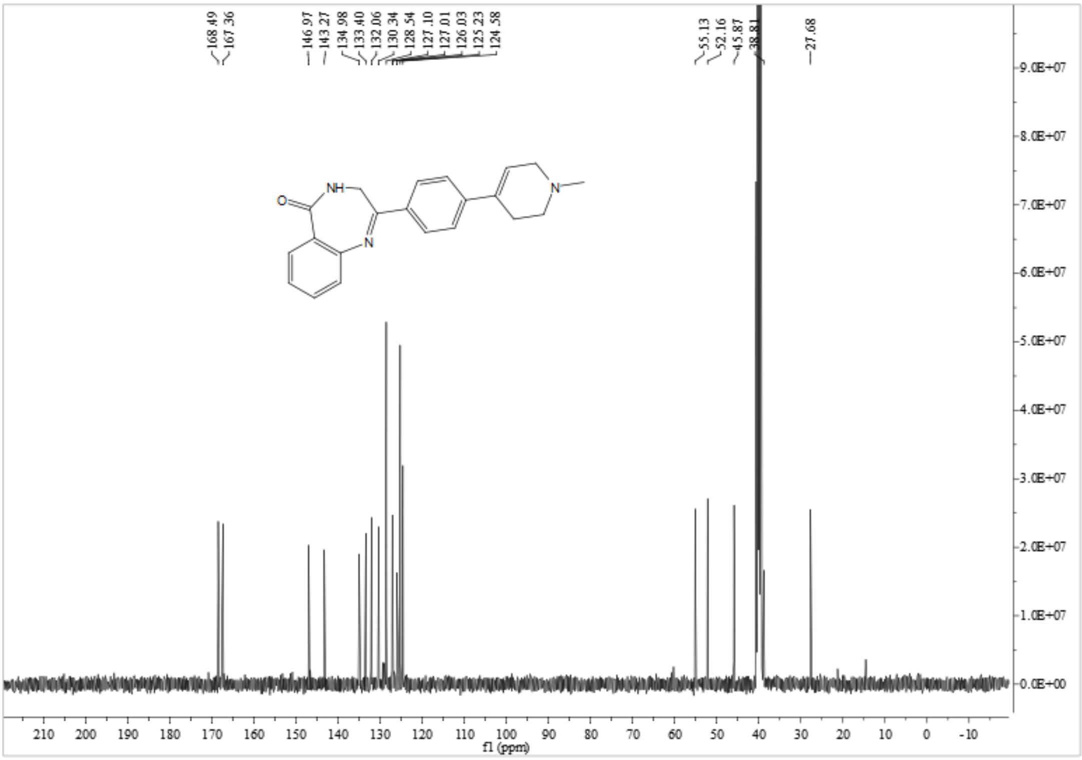

^{13}C-NMR spectrum of compound H45

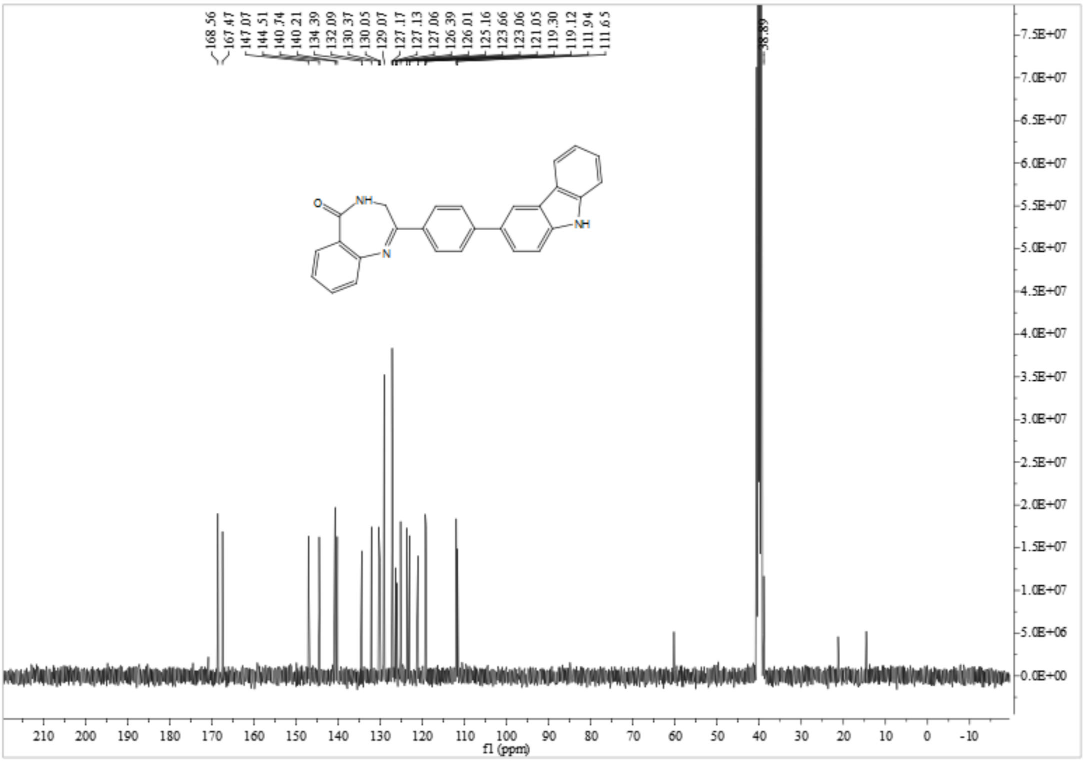

^{13}C-NMR spectrum of compound H46

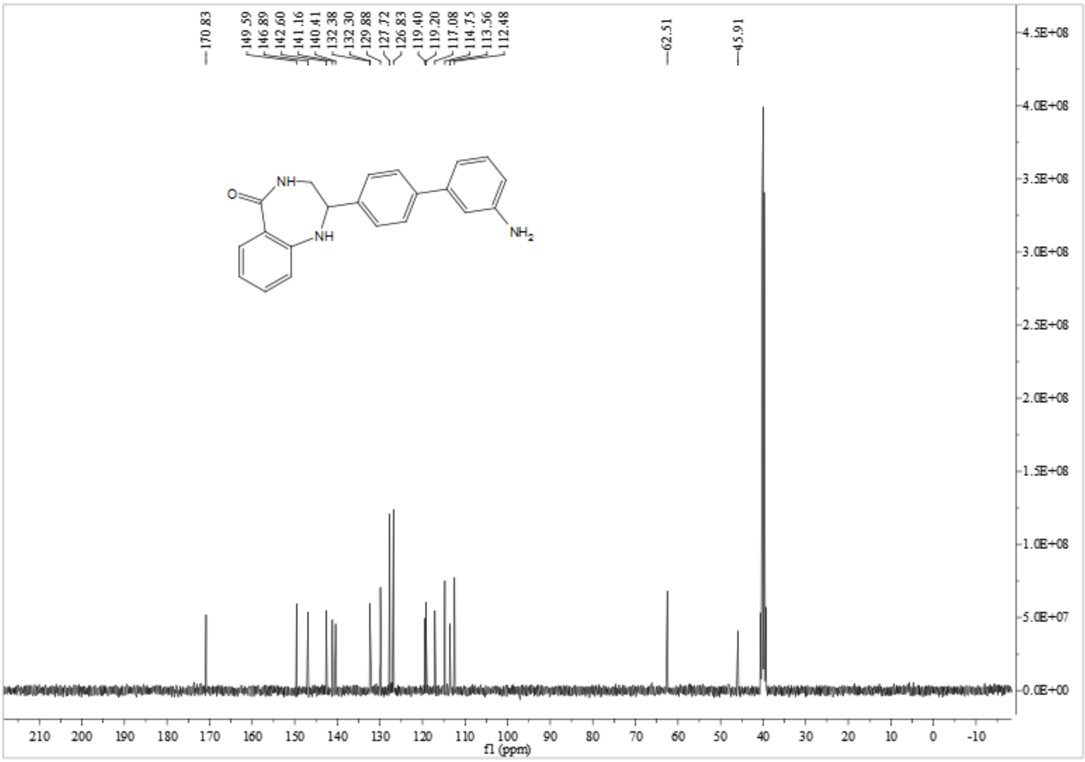

^{13}C-NMR spectrum of compound H47

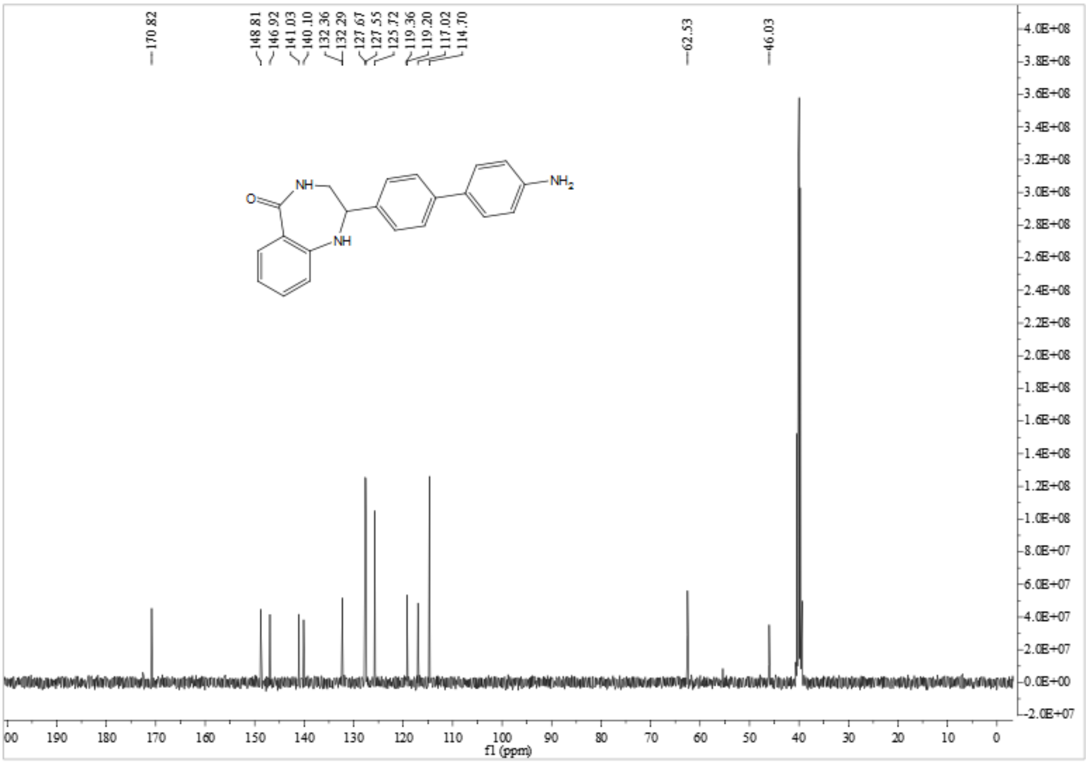

^{13}C-NMR spectrum of compound H48

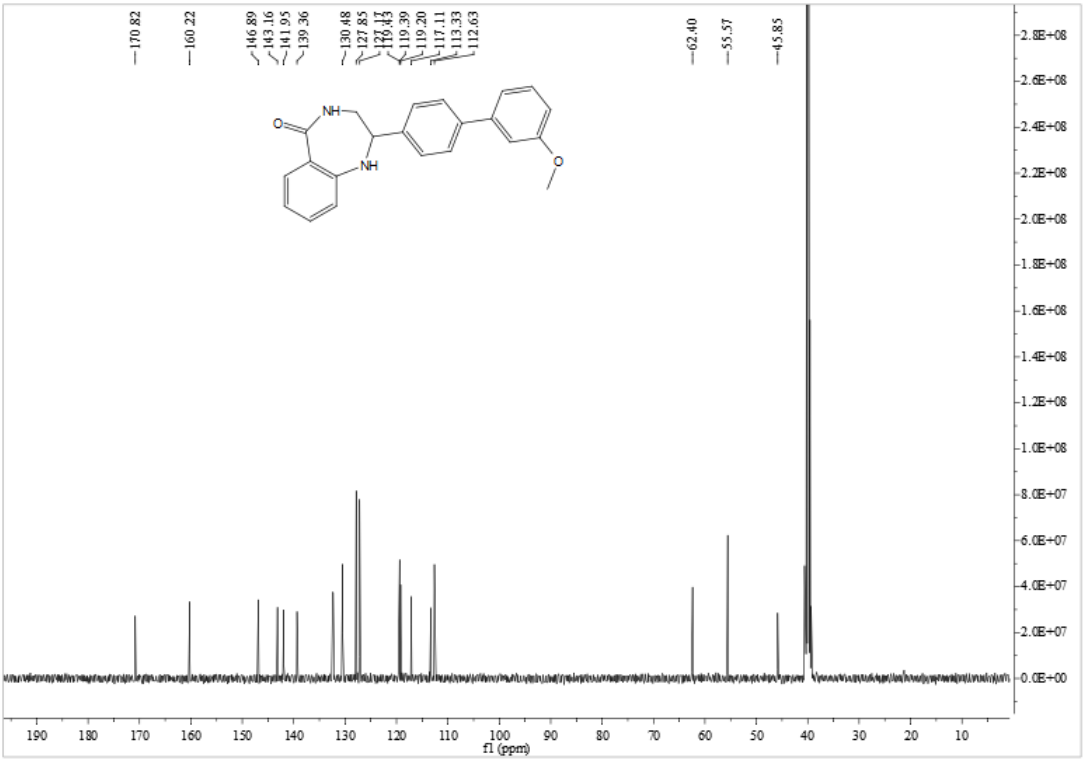

^{13}C-NMR spectrum of compound H49

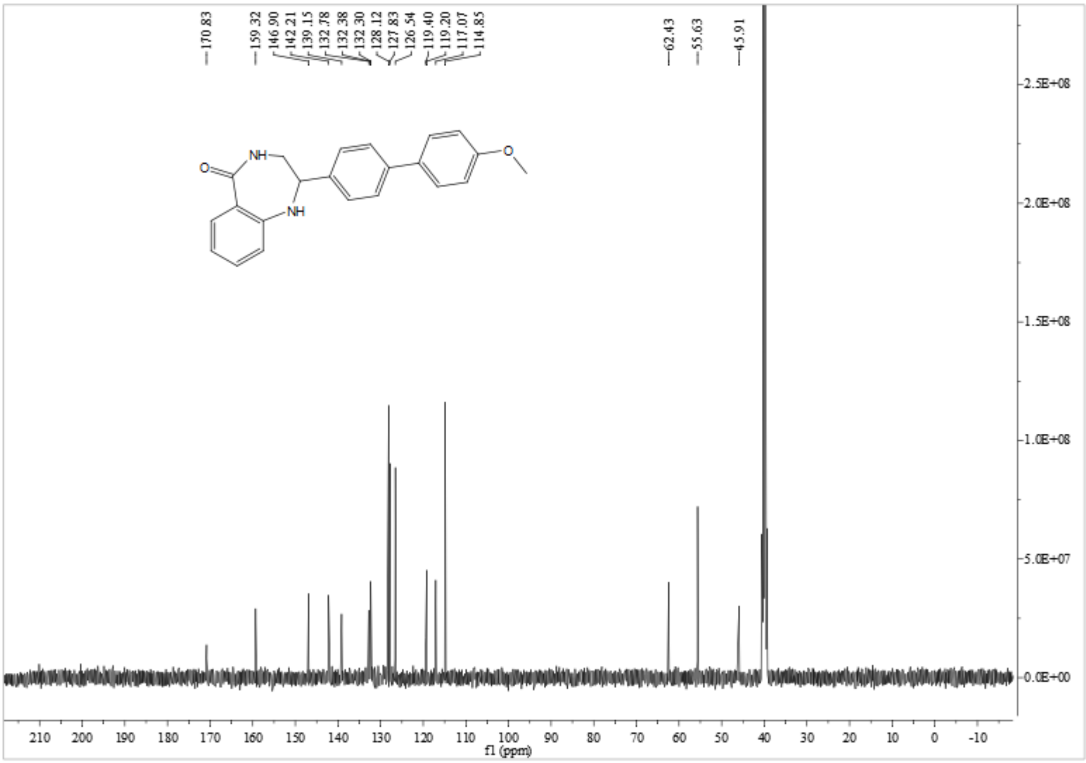

^{13}C-NMR spectrum of compound H50

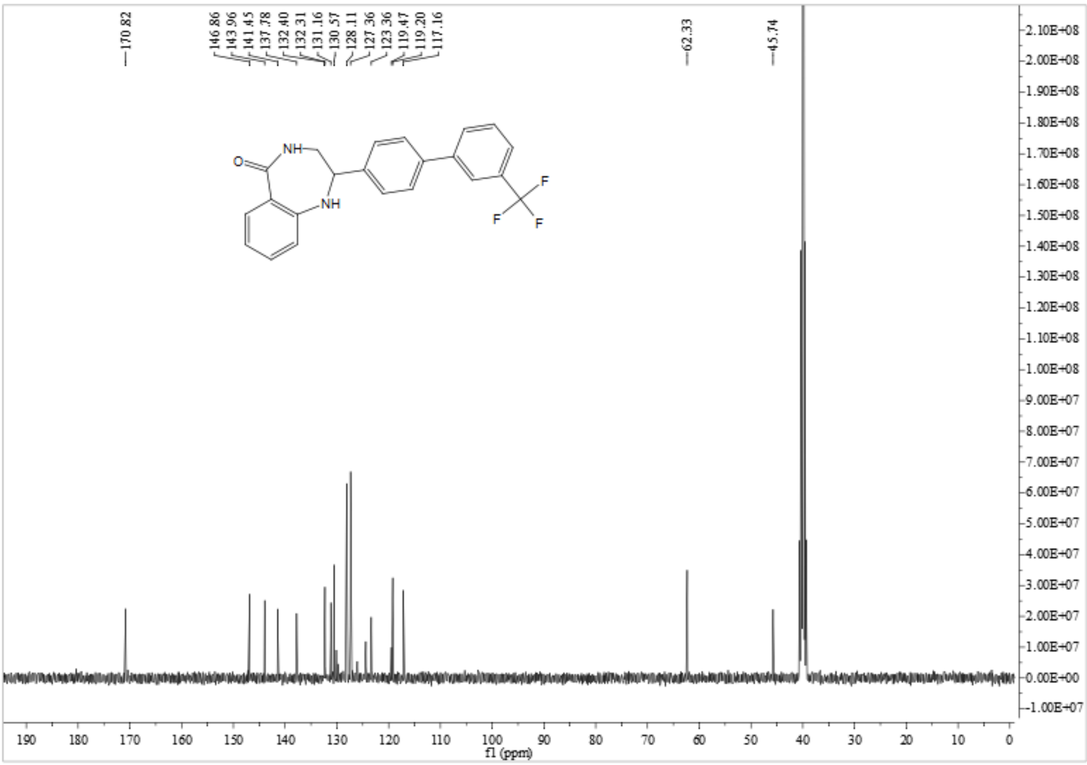

^{13}C-NMR spectrum of compound H51

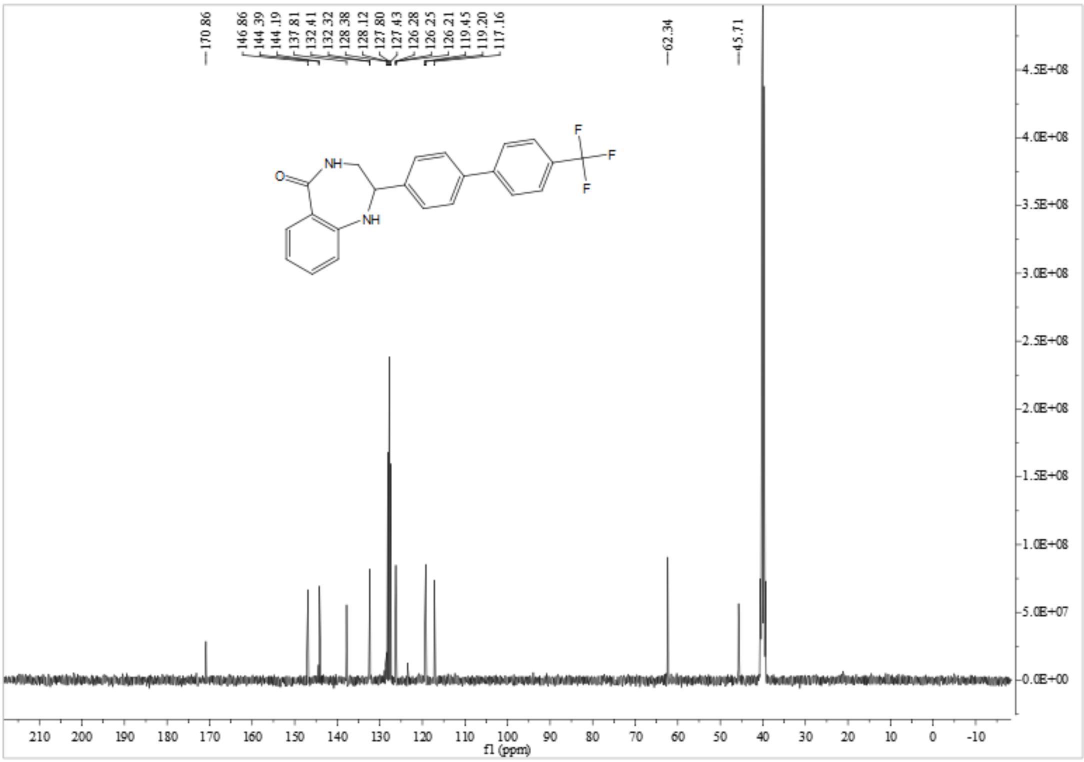

^{13}C-NMR spectrum of compound H52

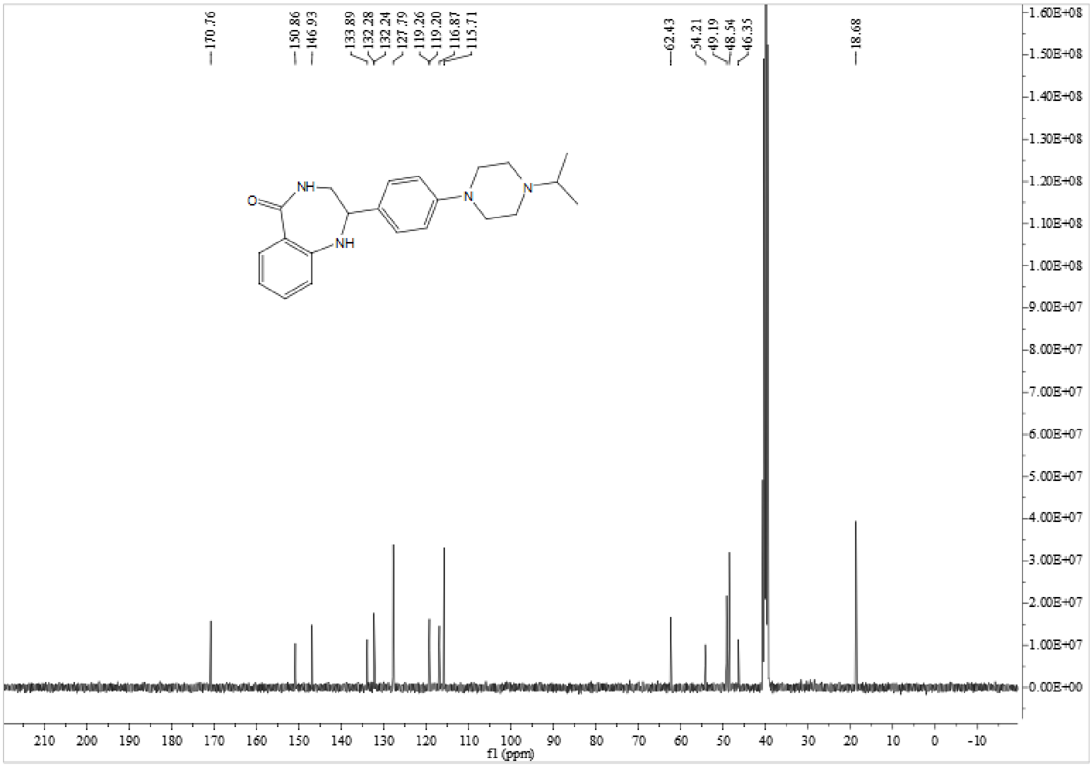

^{13}C-NMR spectrum of compound H53

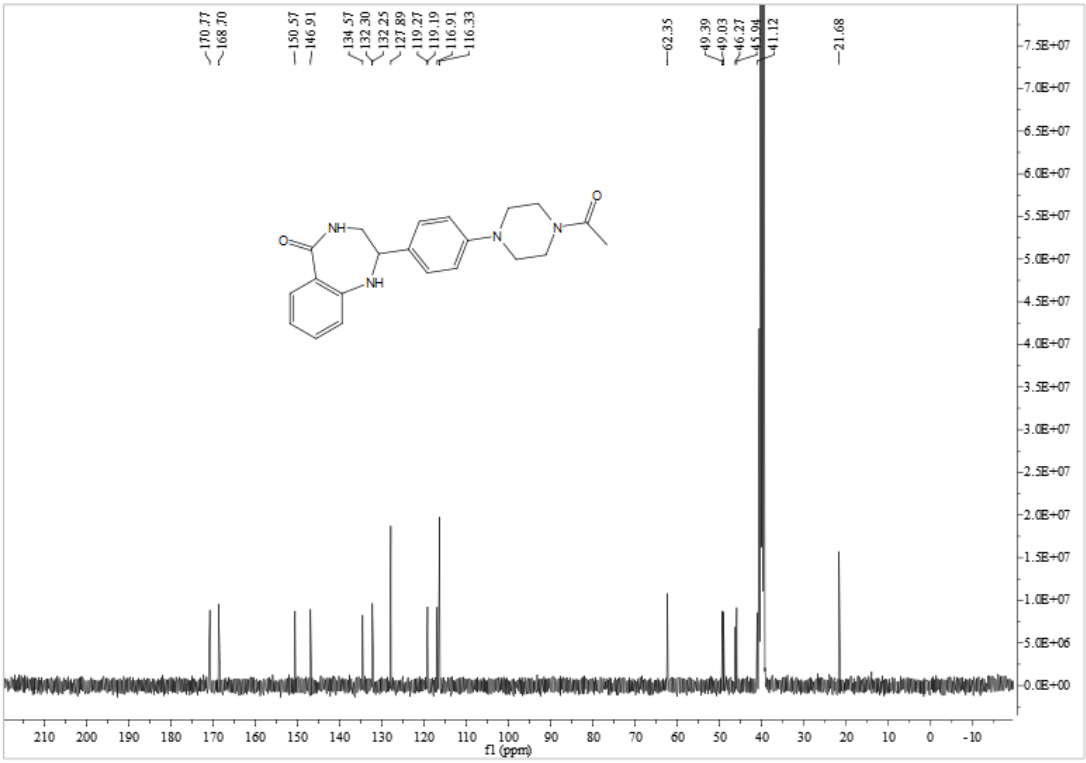

^{13}C-NMR spectrum of compound H54

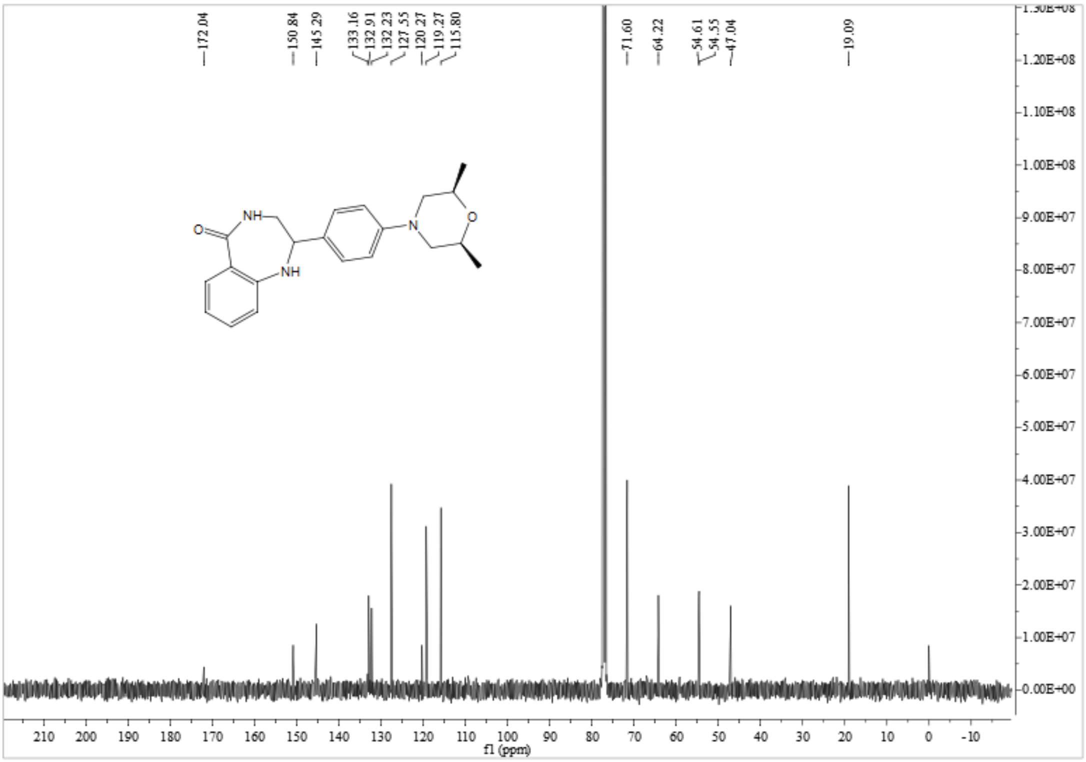

^{13}C-NMR spectrum of compound H55

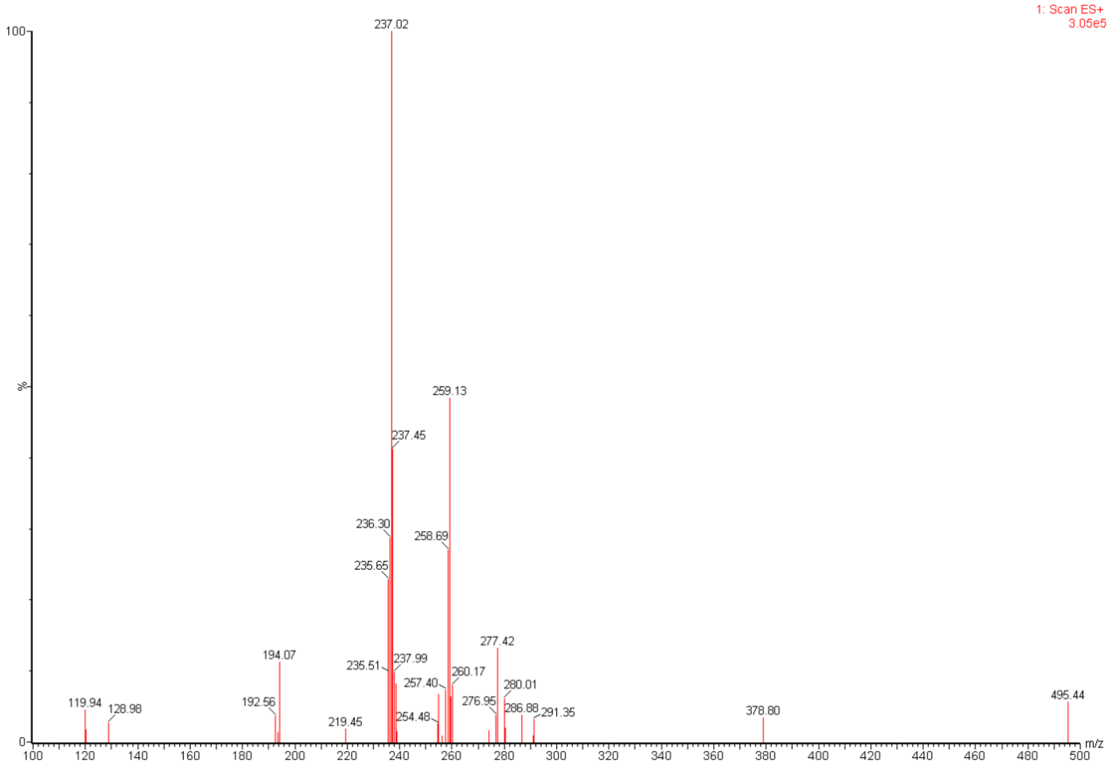

MS spectrum of compound H4

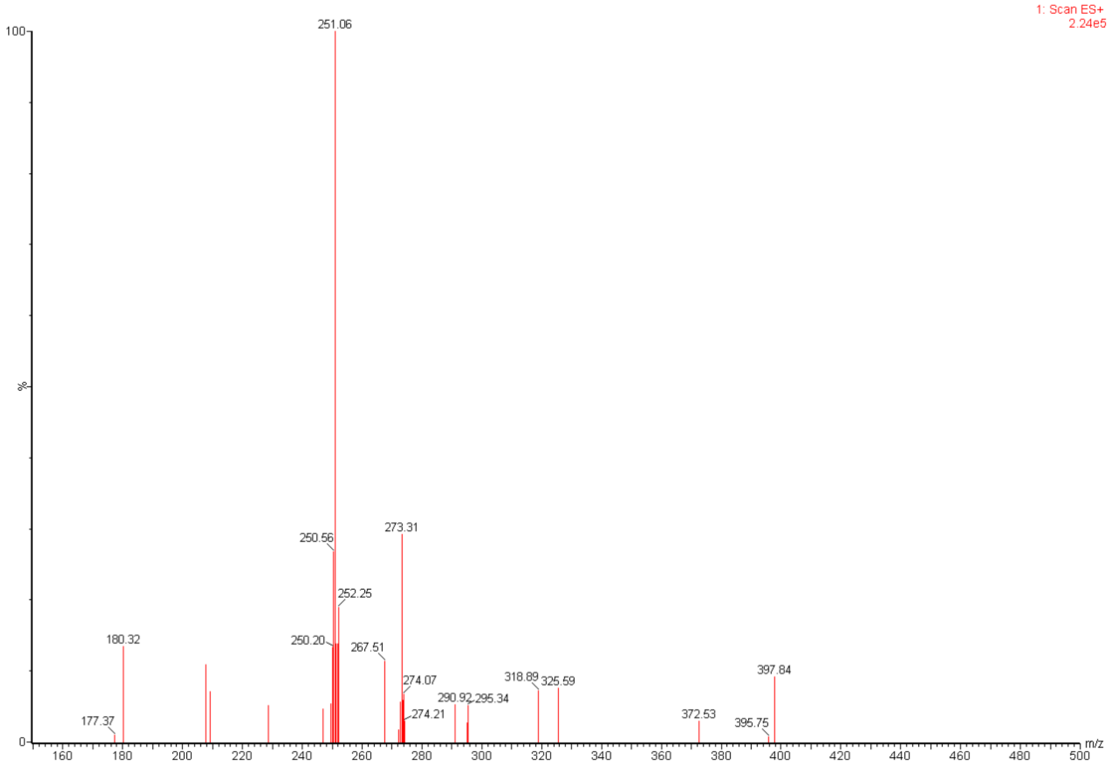

MS spectrum of compound H5

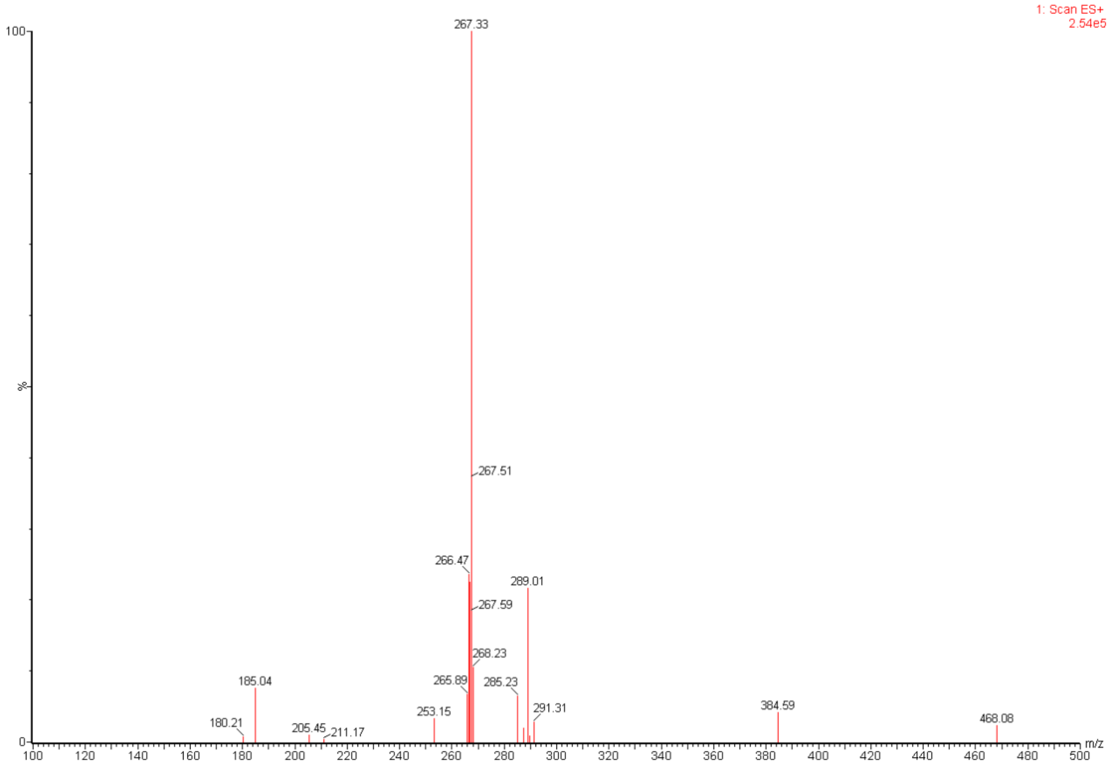

MS spectrum of compound H6

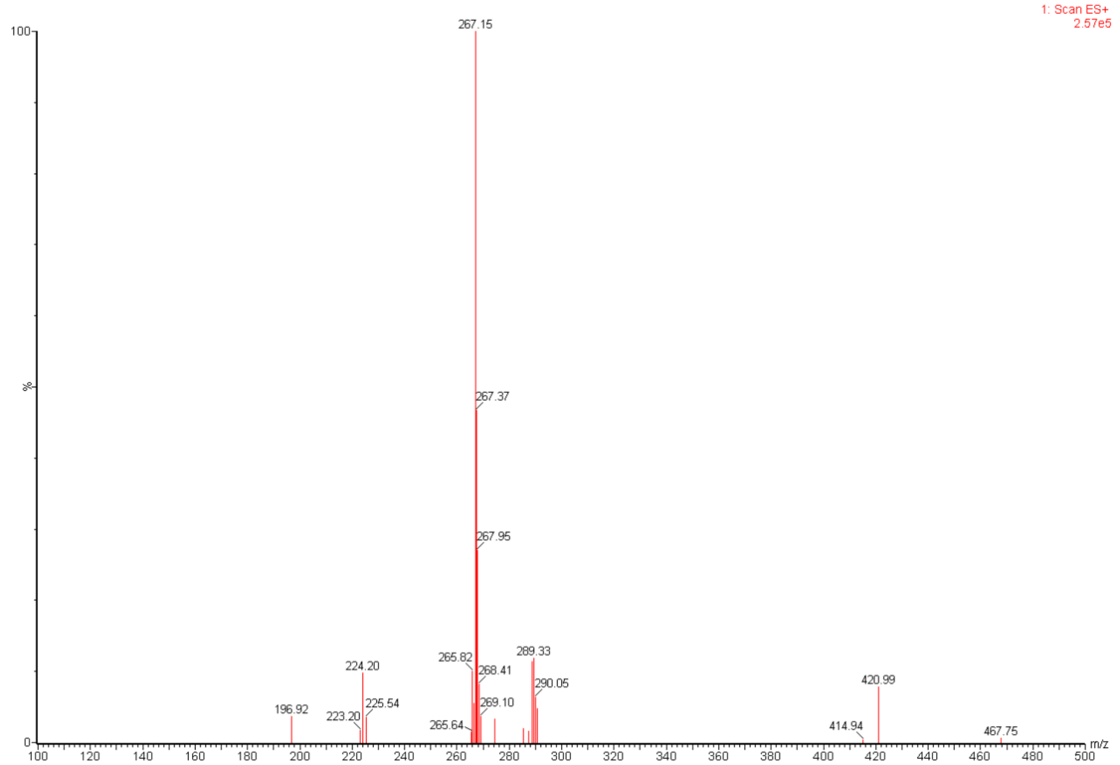

MS spectrum of compound H7

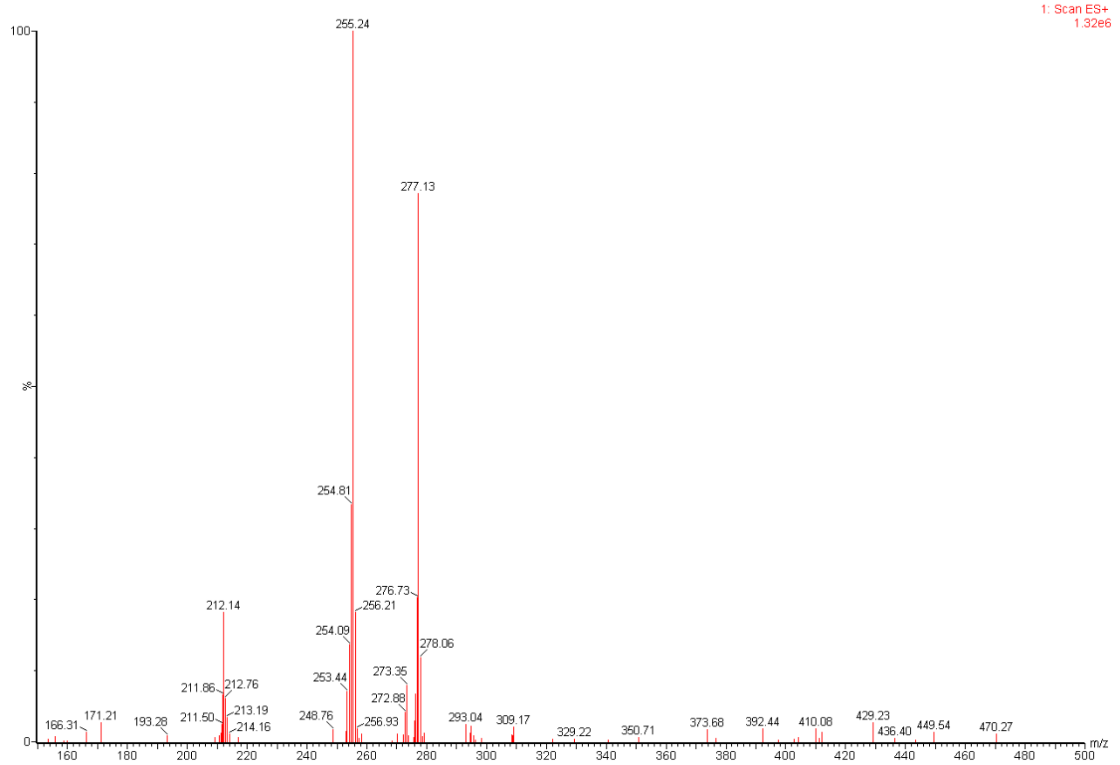

MS spectrum of compound H8

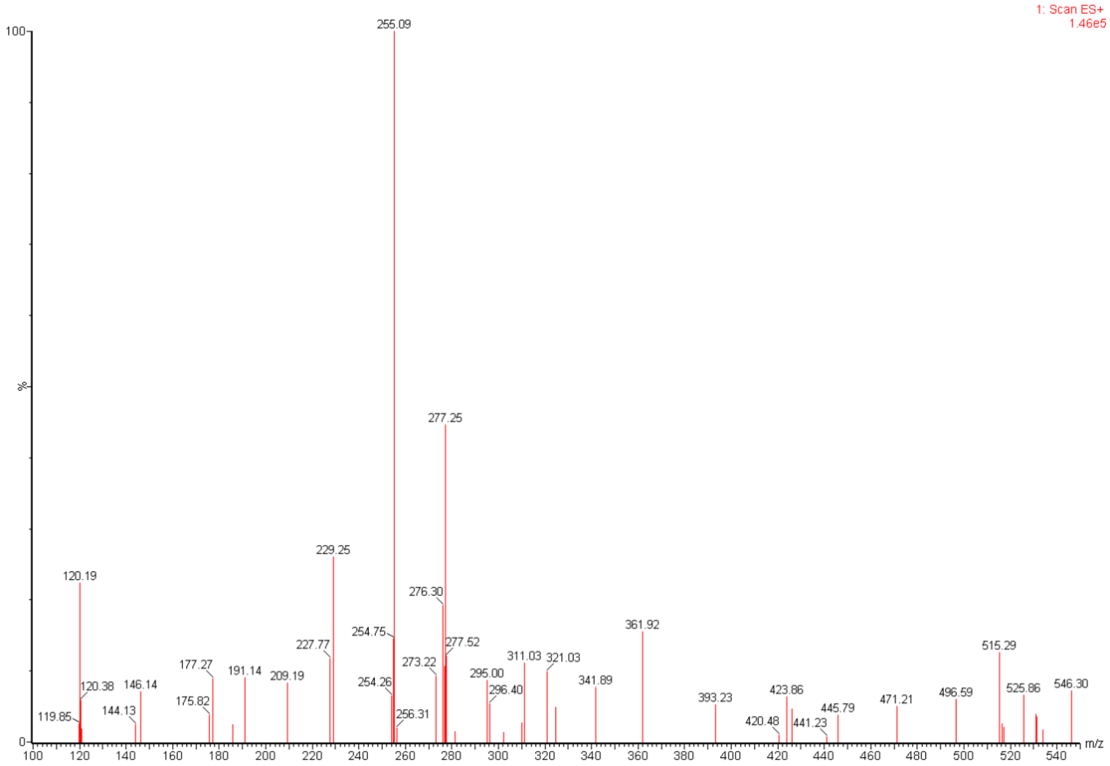

MS spectrum of compound H9

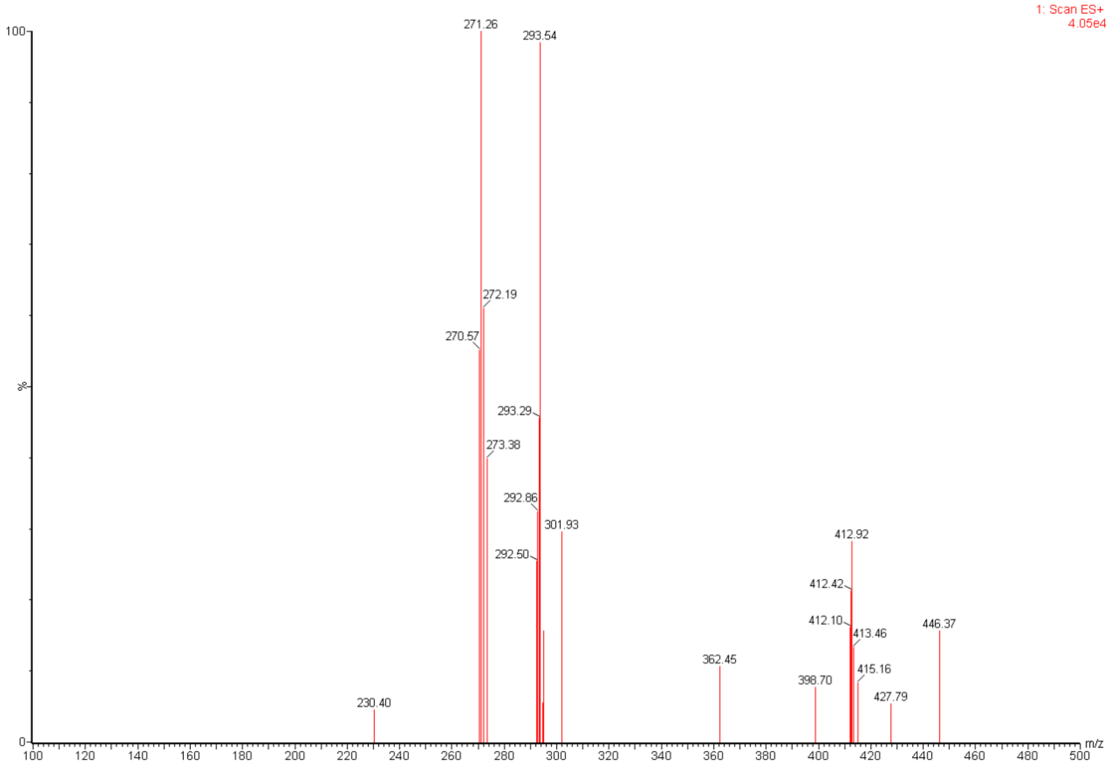

MS spectrum of compound H10

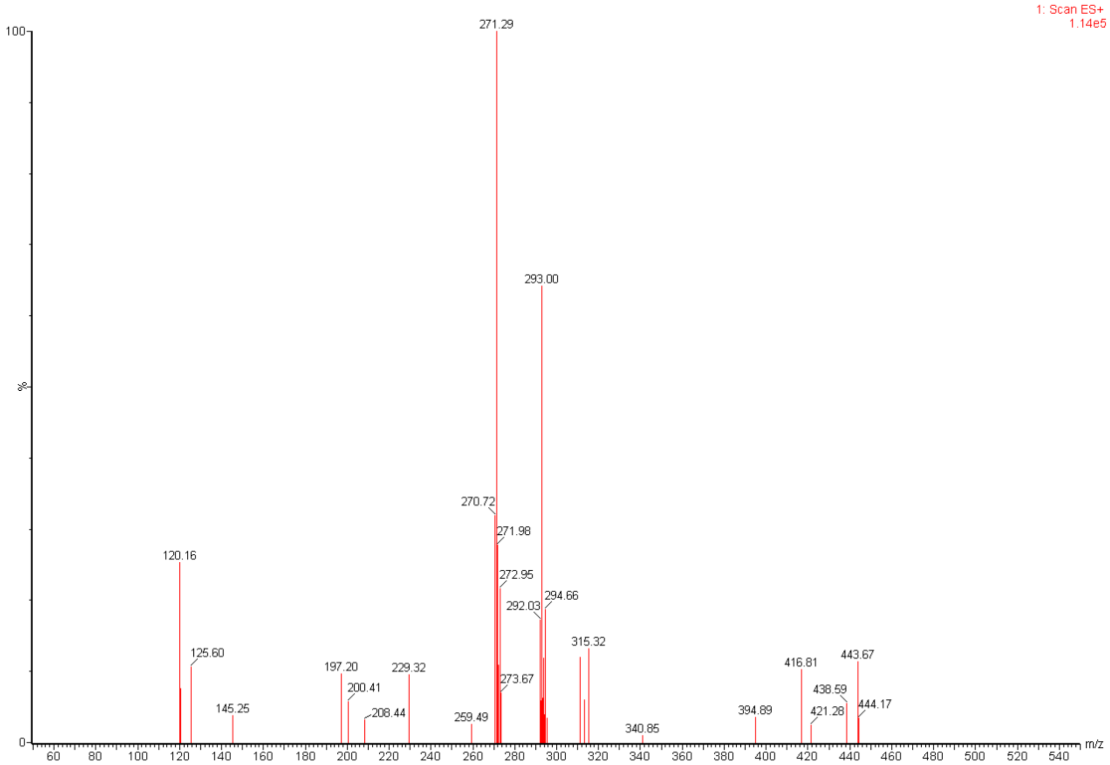

MS spectrum of compound H11

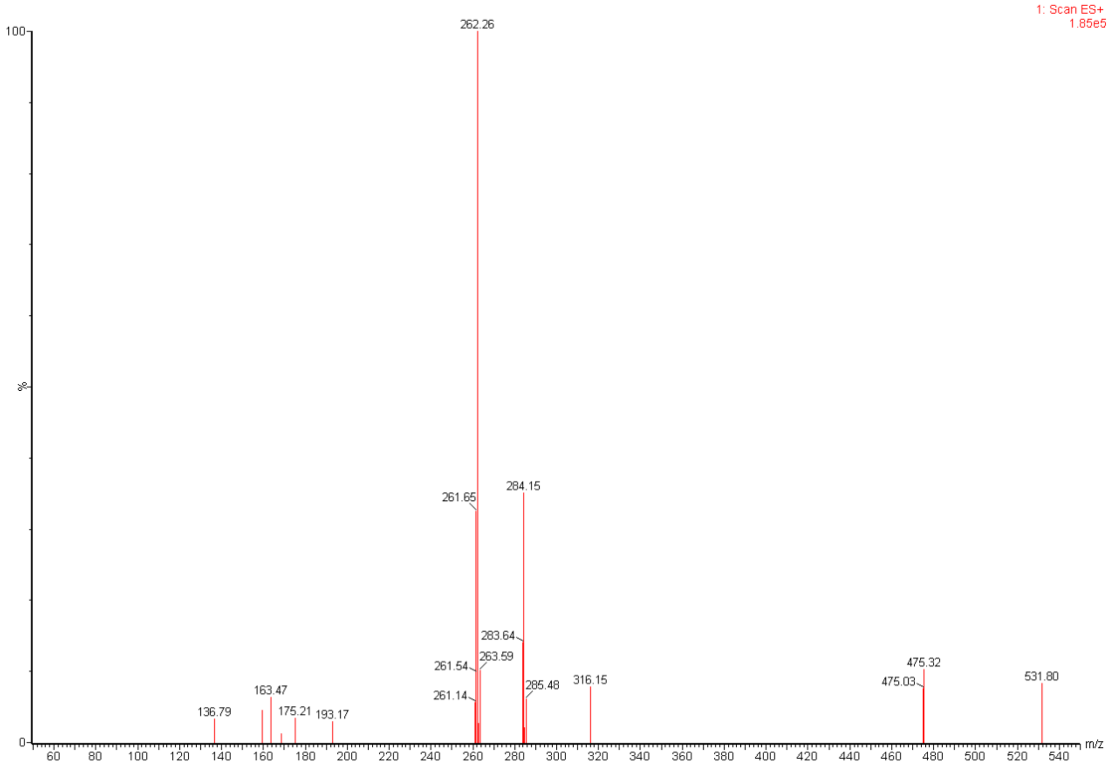

MS spectrum of compound H12

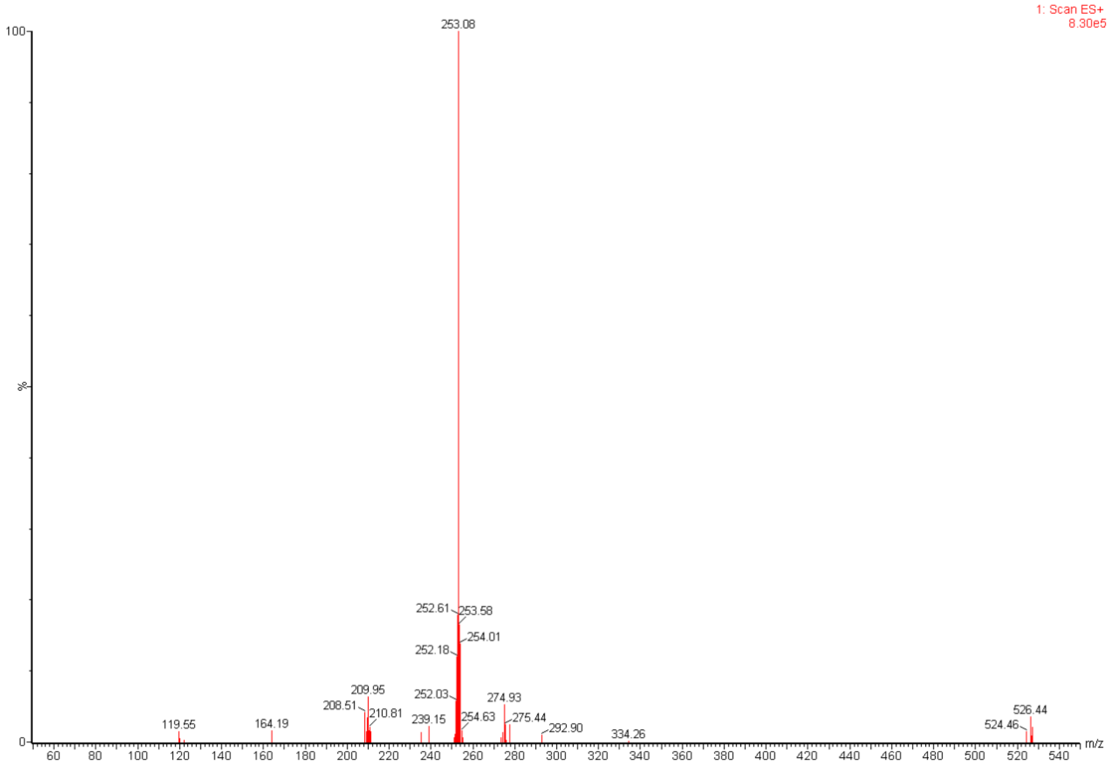

MS spectrum of compound H13

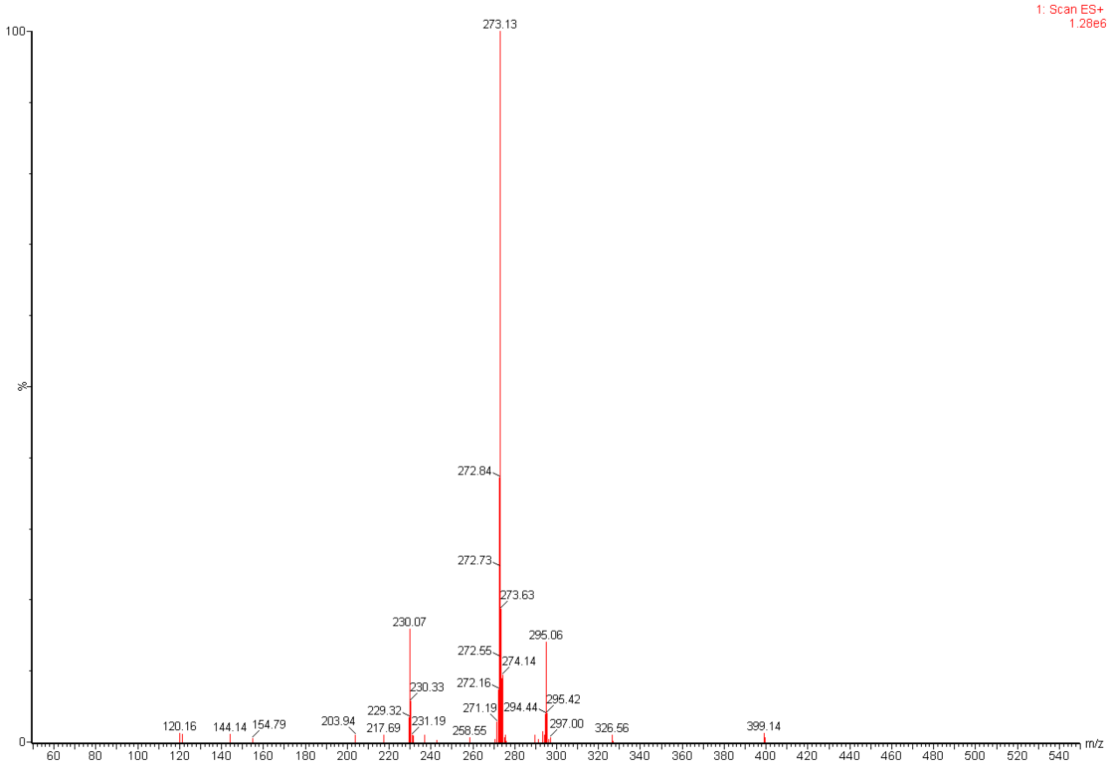

MS spectrum of compound H14

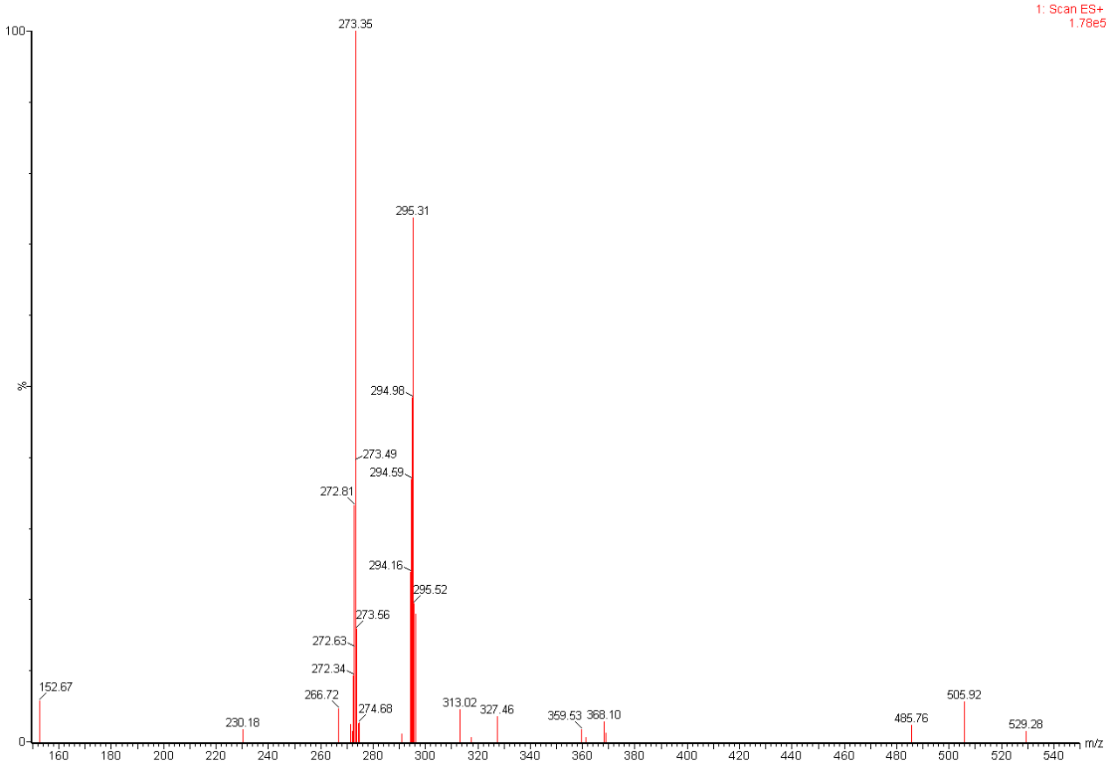

MS spectrum of compound H15

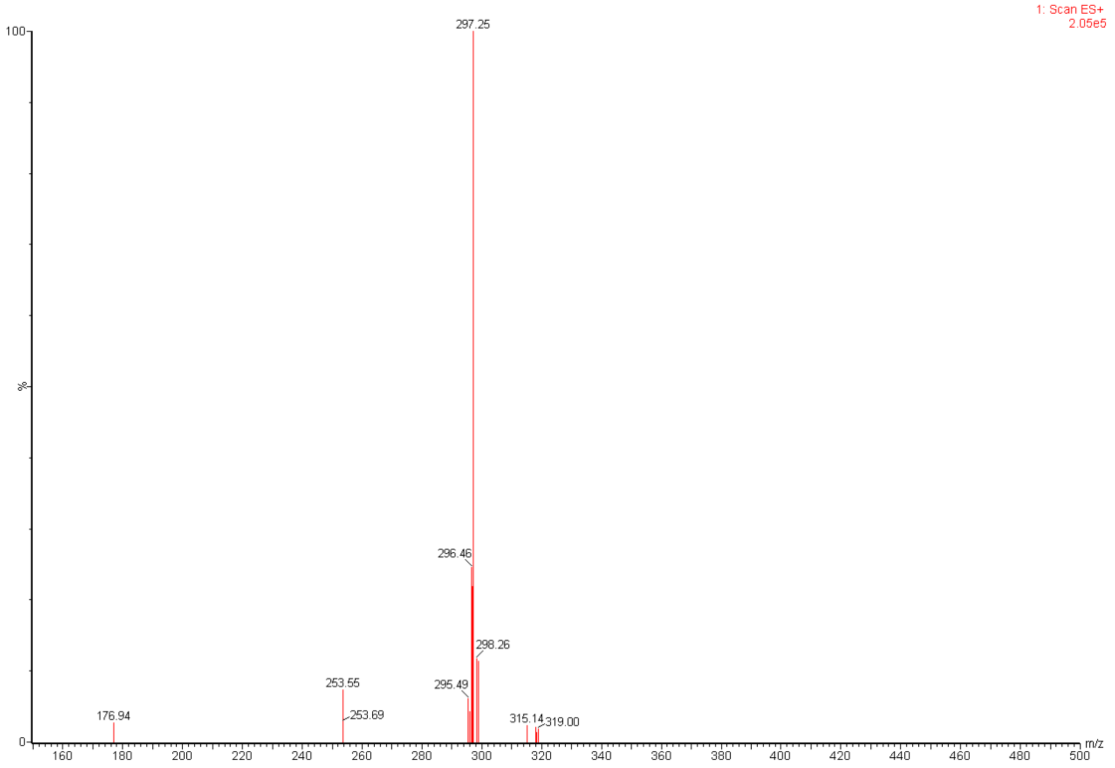

MS spectrum of compound H16

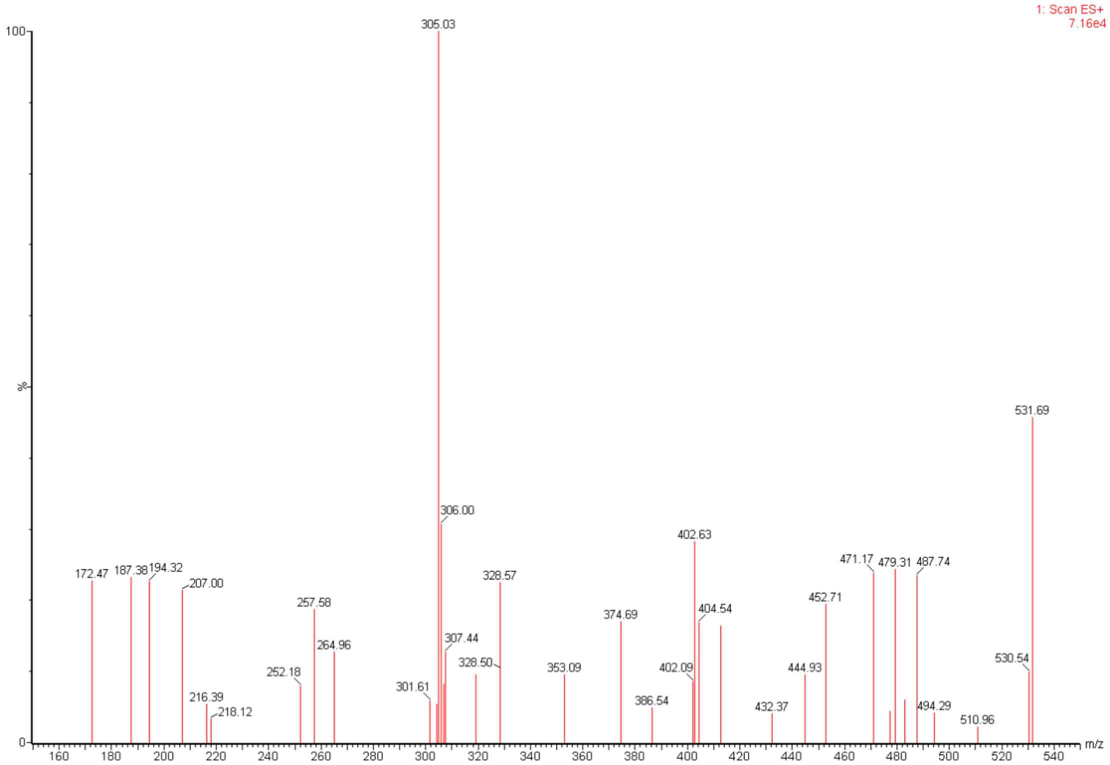

MS spectrum of compound H17

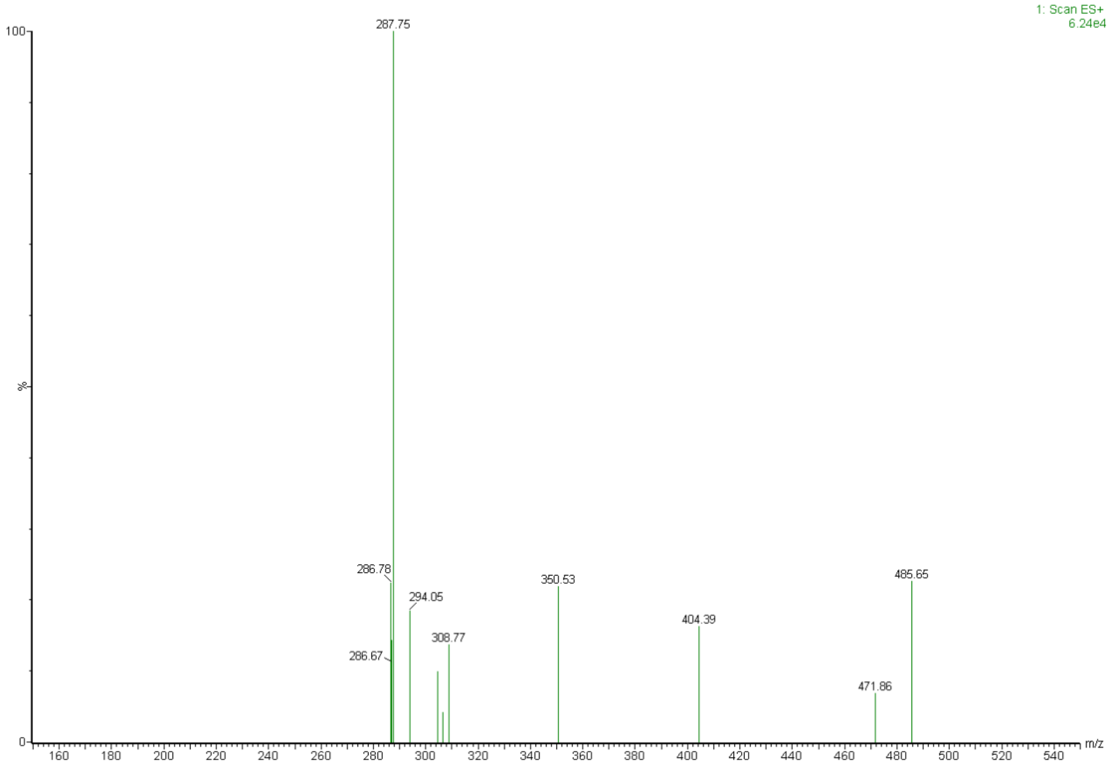

MS spectrum of compound H18

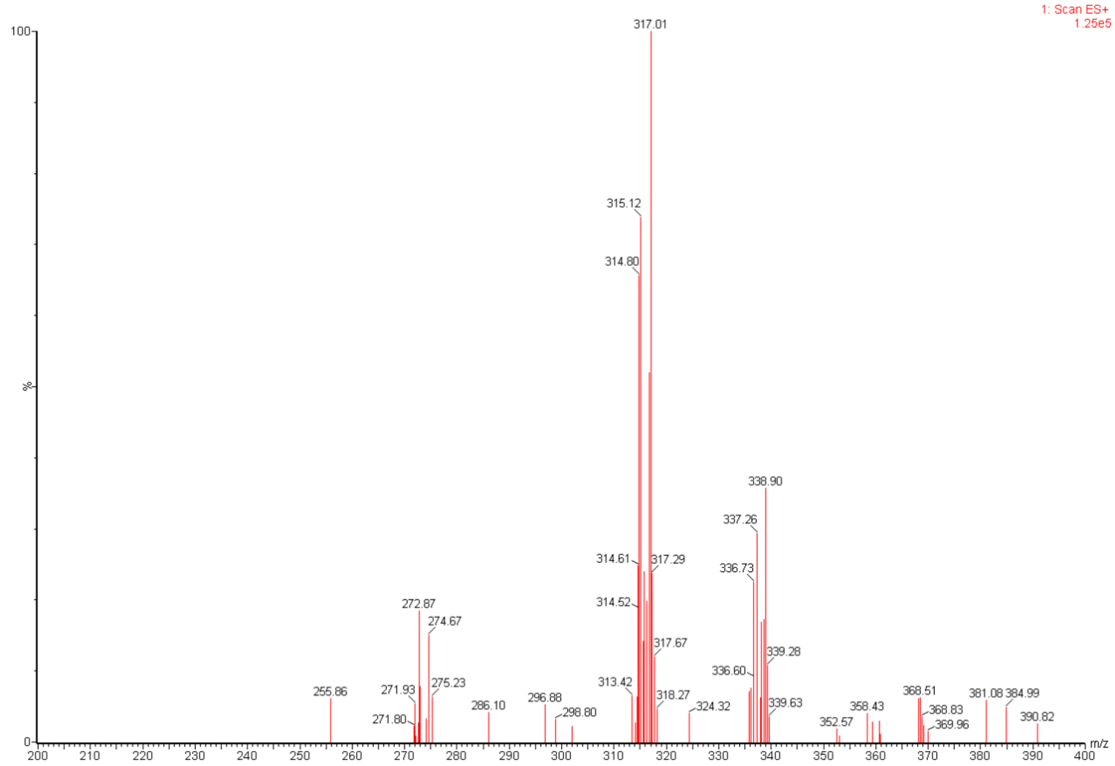

MS spectrum of compound H19

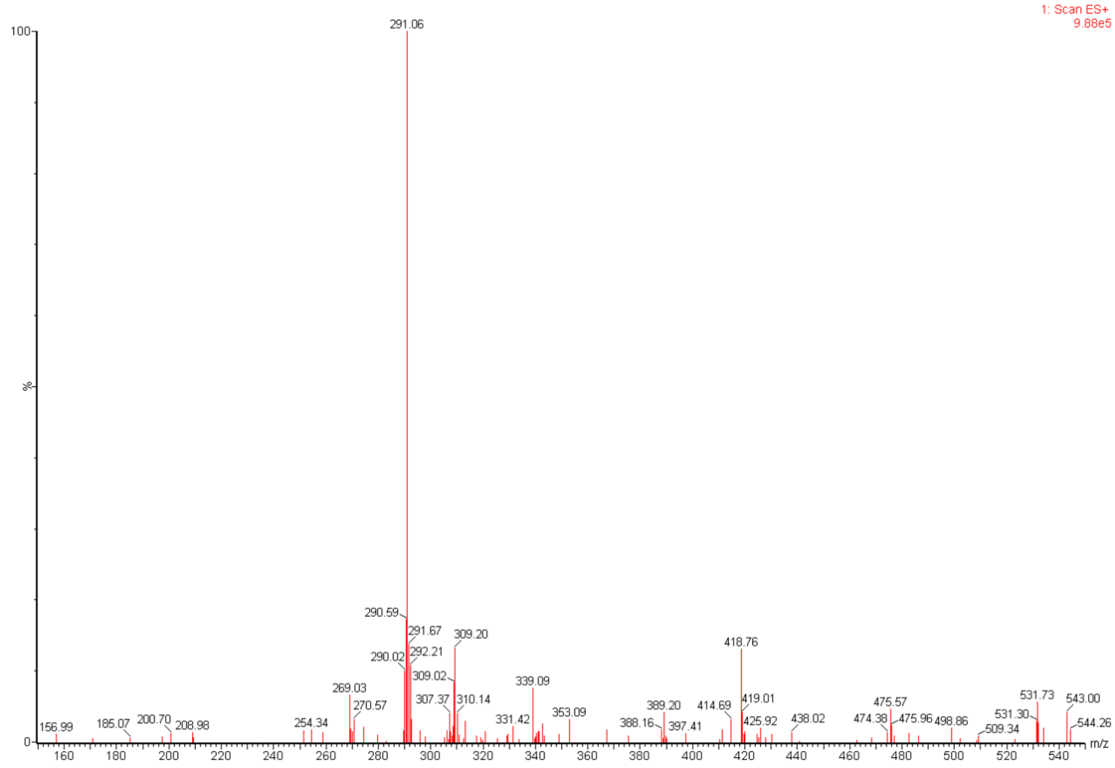

MS spectrum of compound H20

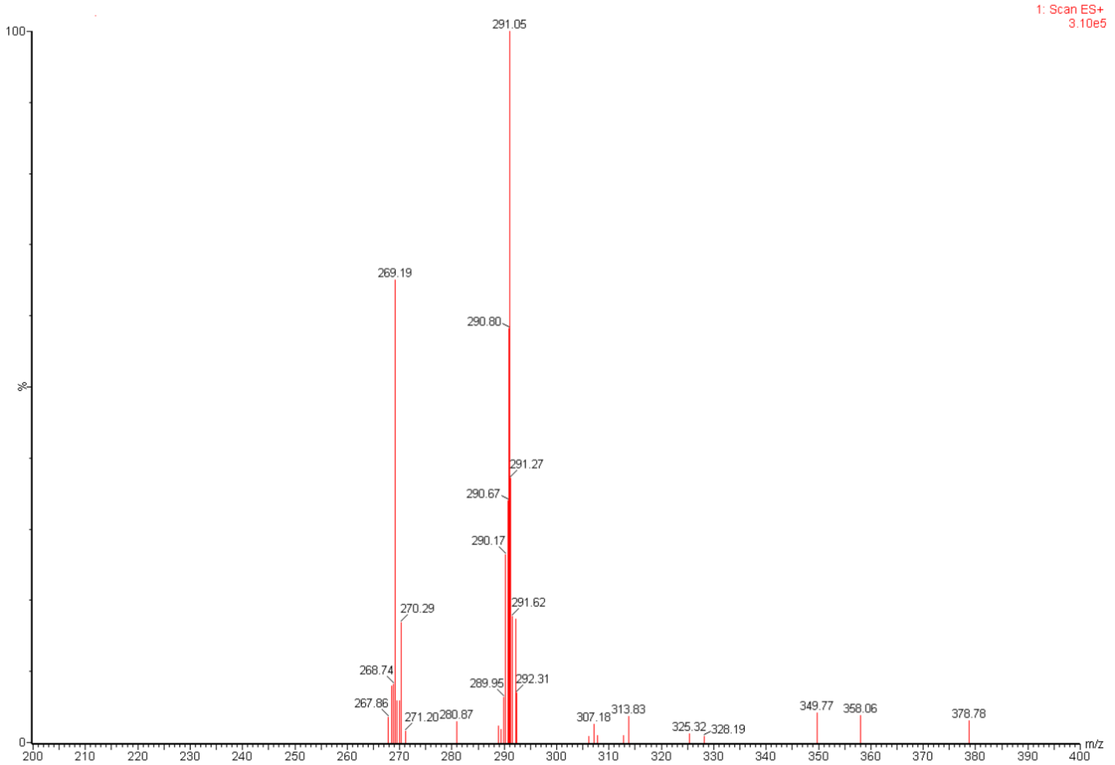

MS spectrum of compound H21

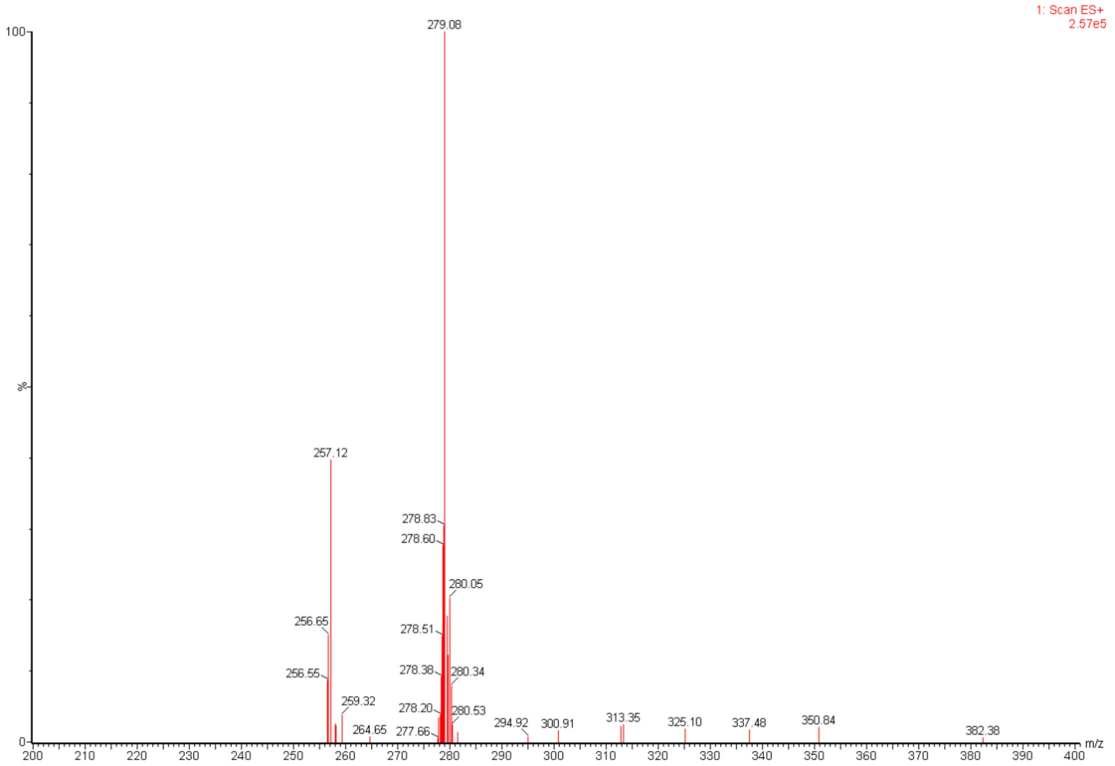

MS spectrum of compound H22

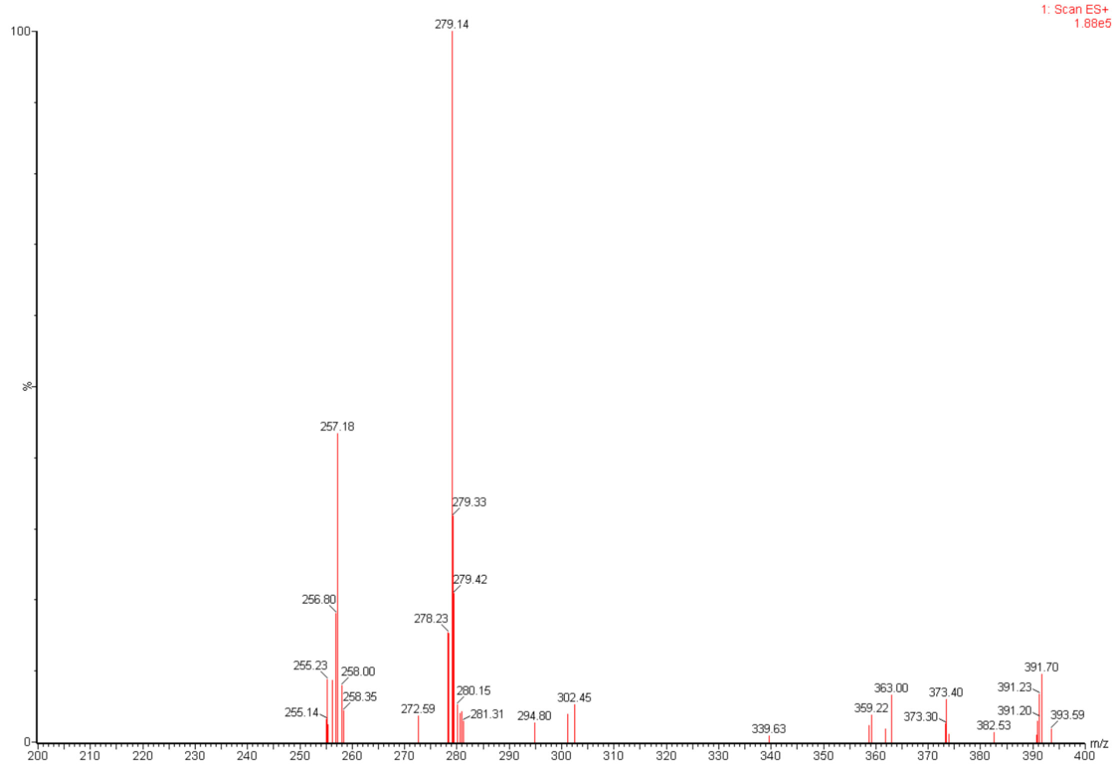

MS spectrum of compound H23

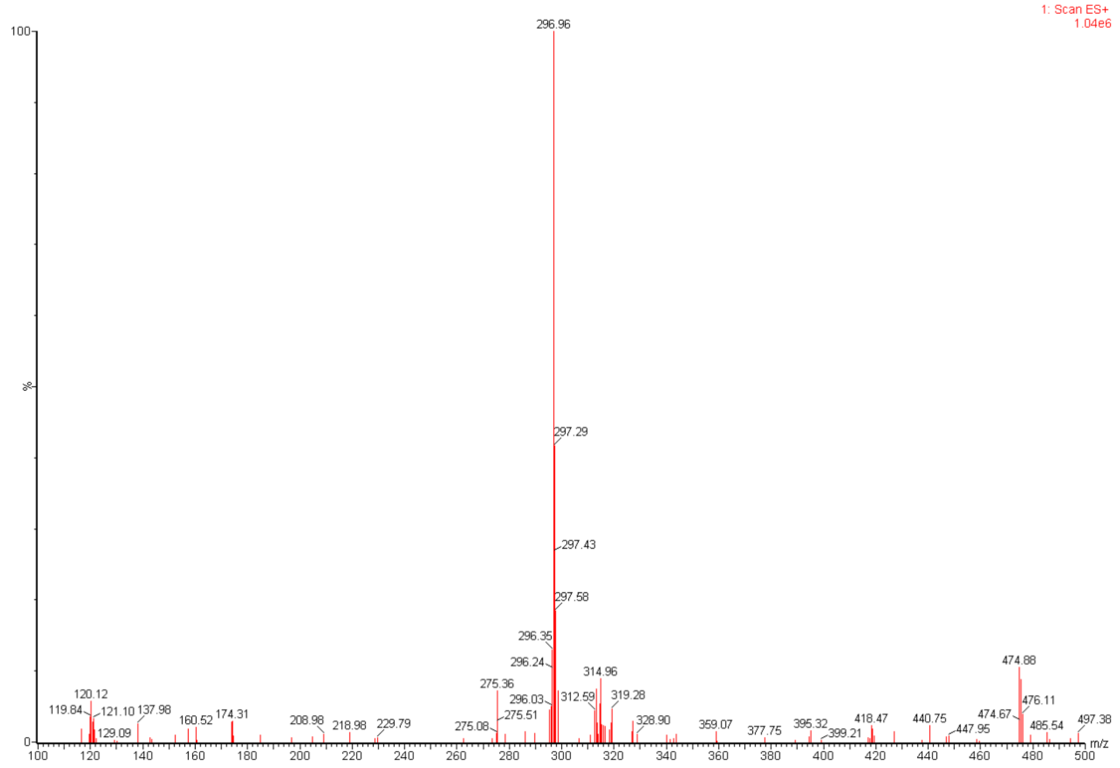

MS spectrum of compound H24

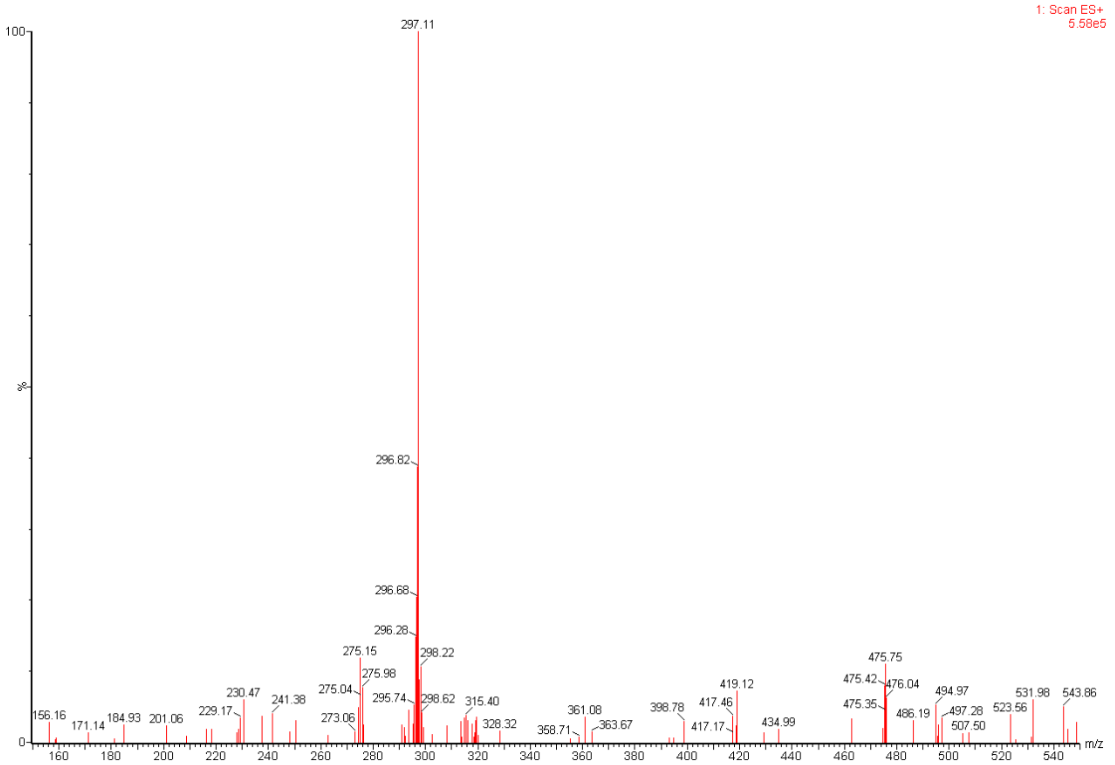

MS spectrum of compound H25

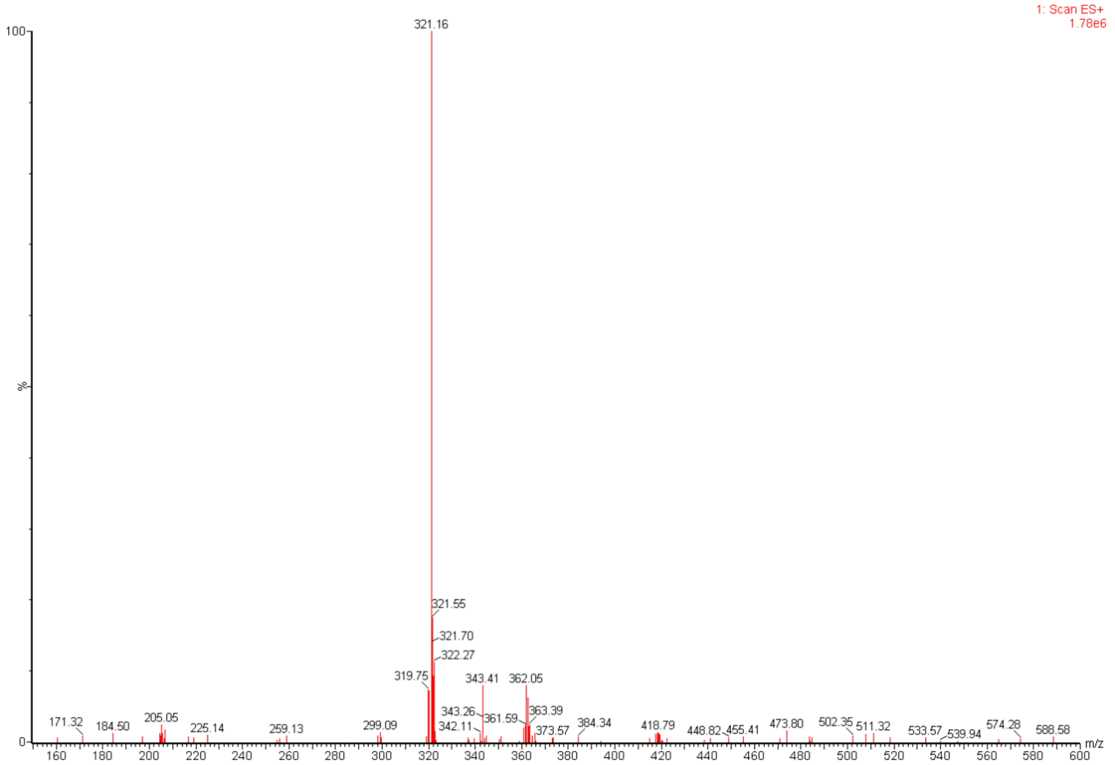

MS spectrum of compound H26

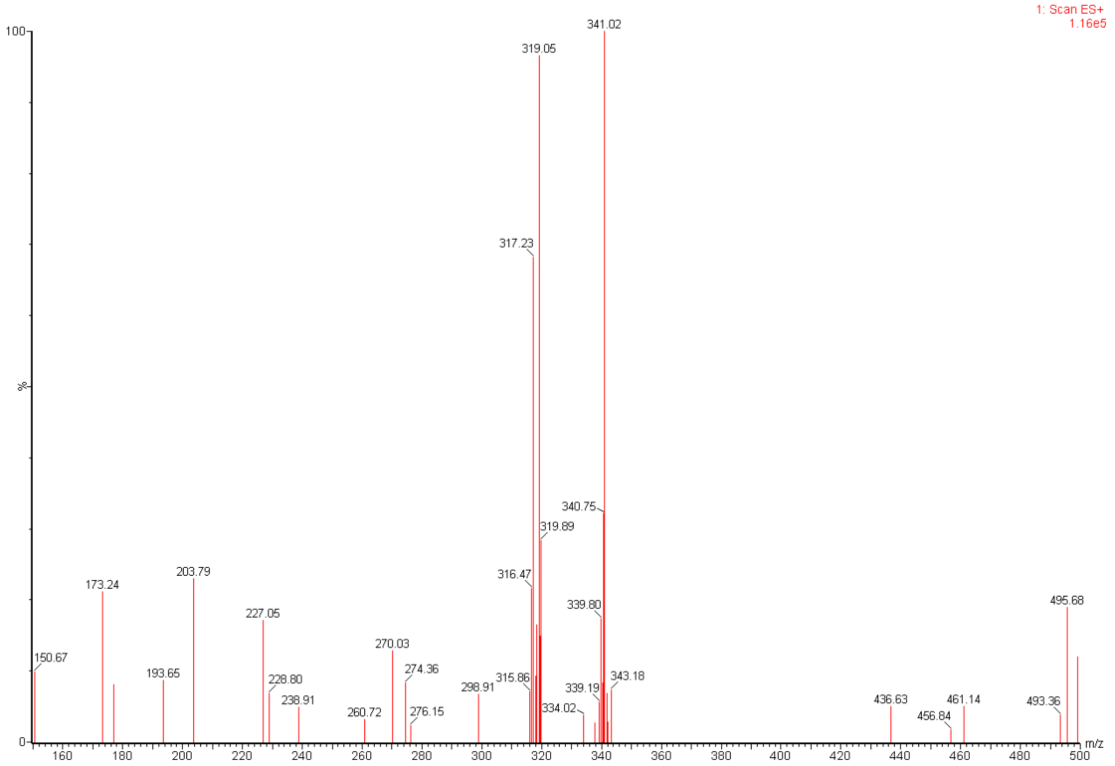

MS spectrum of compound H27

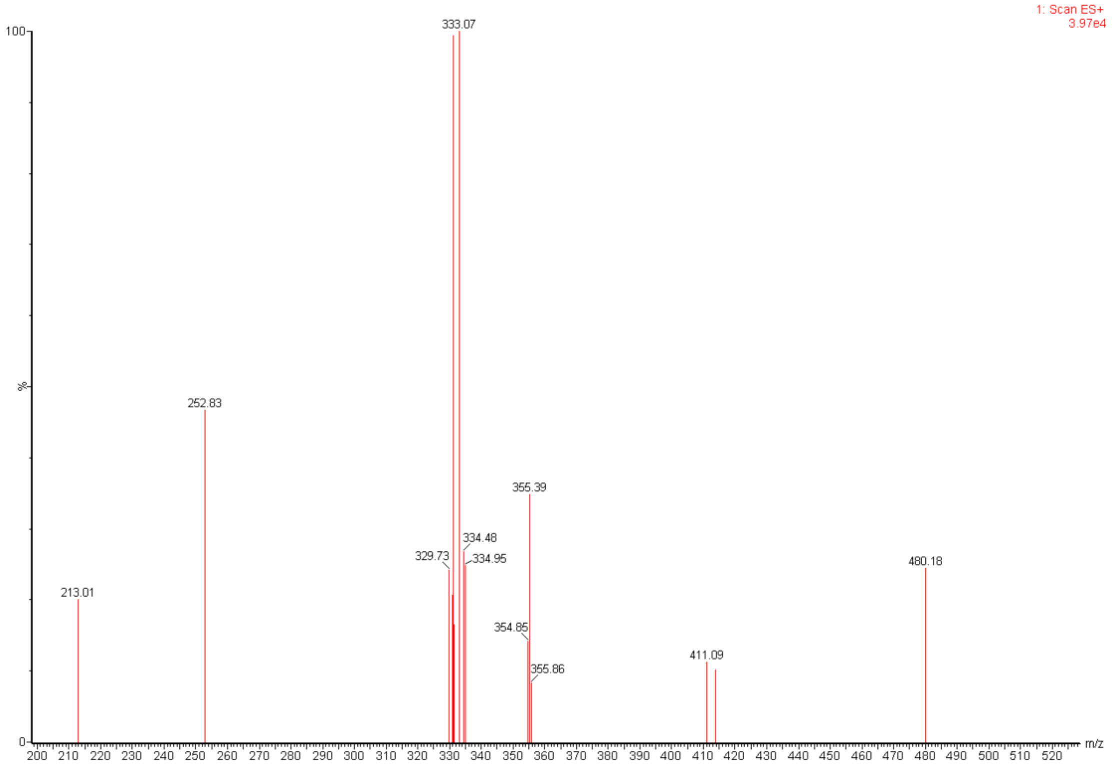

MS spectrum of compound H28

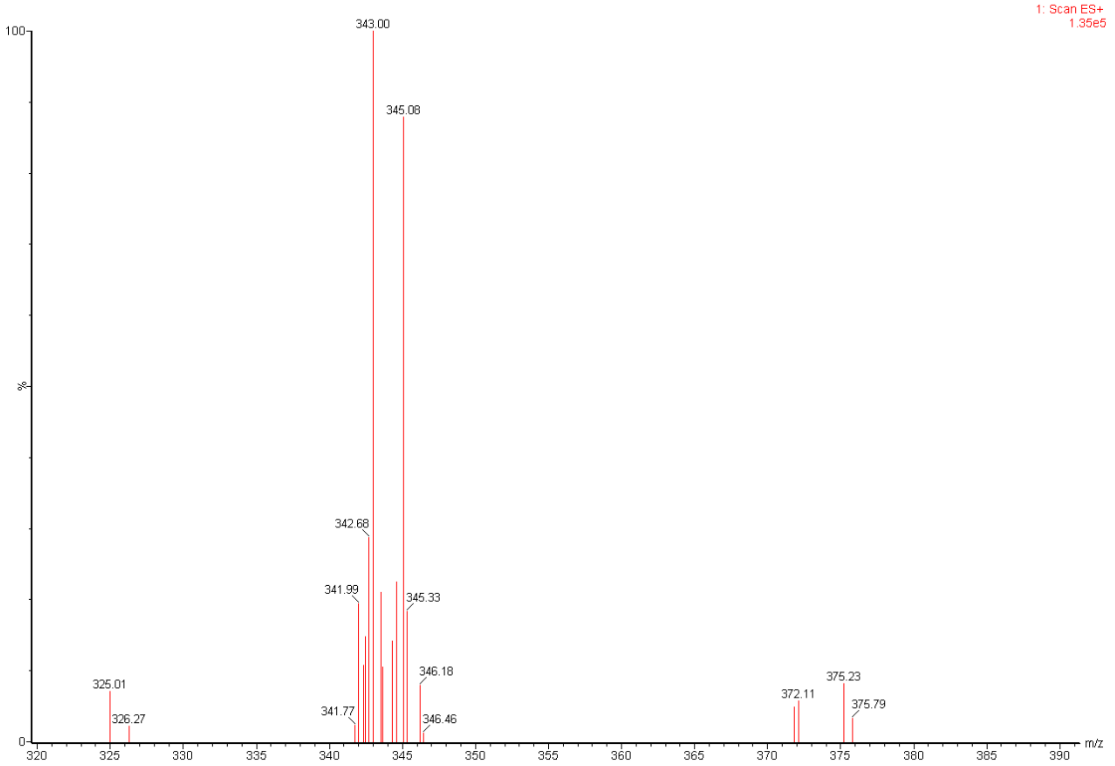

MS spectrum of compound H29

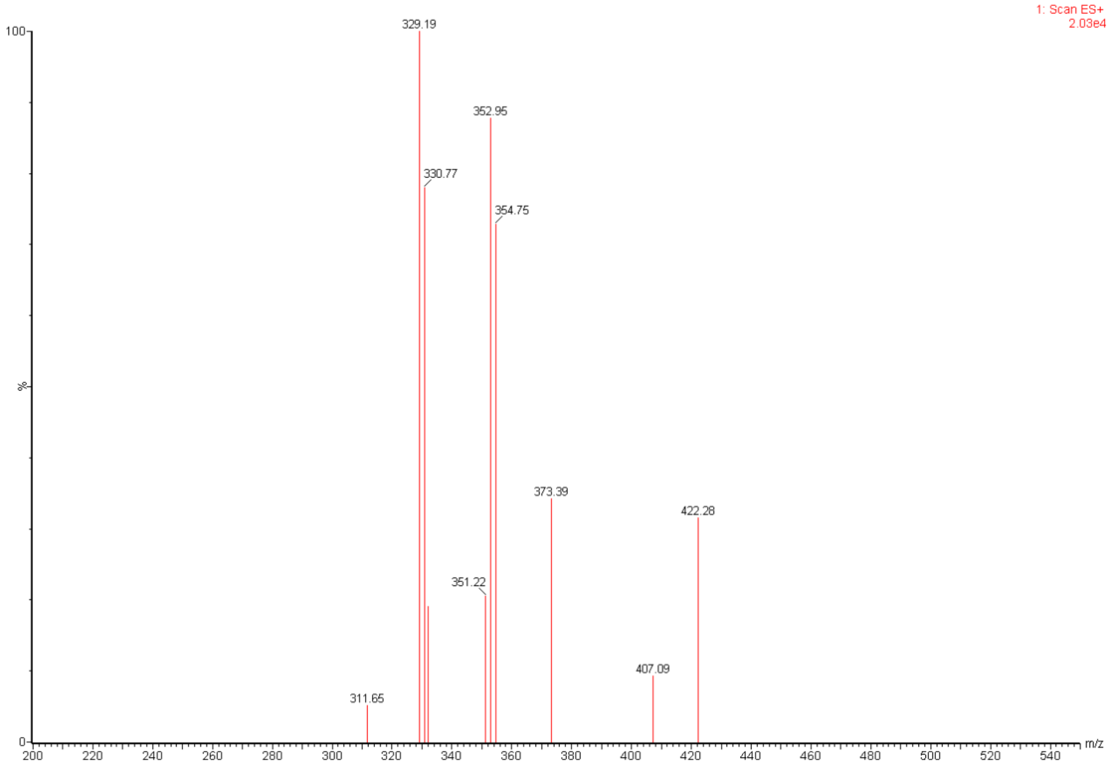

MS spectrum of compound H30

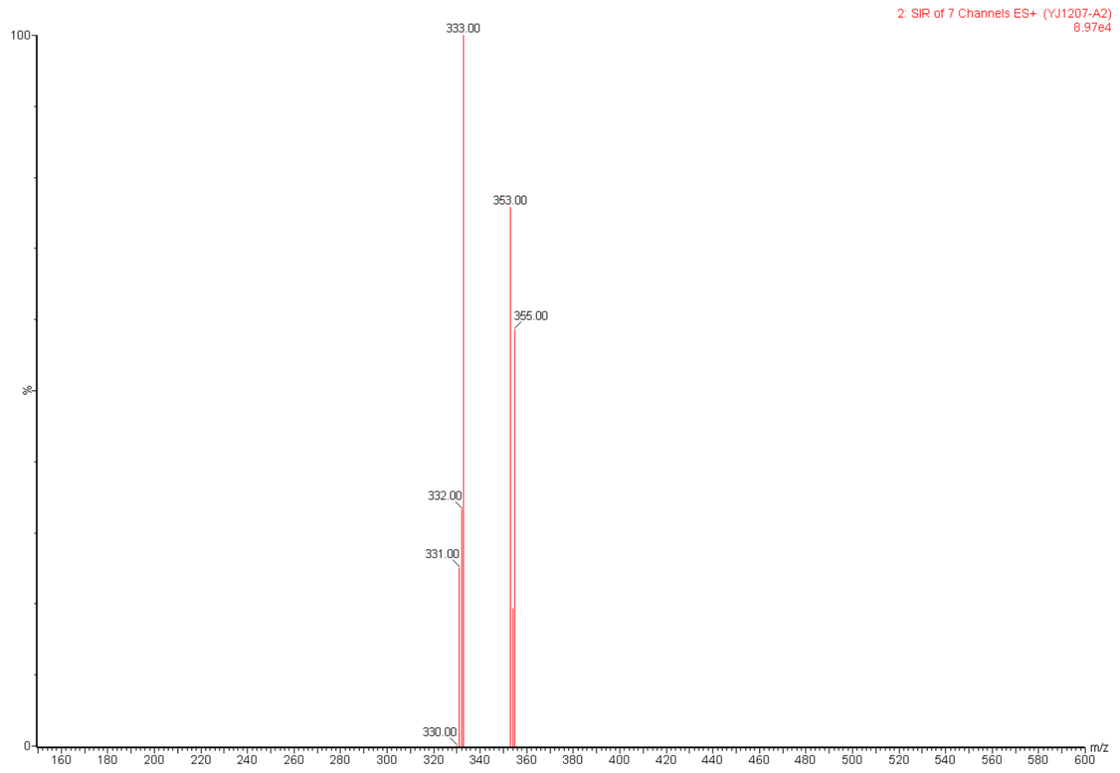

MS spectrum of compound H31

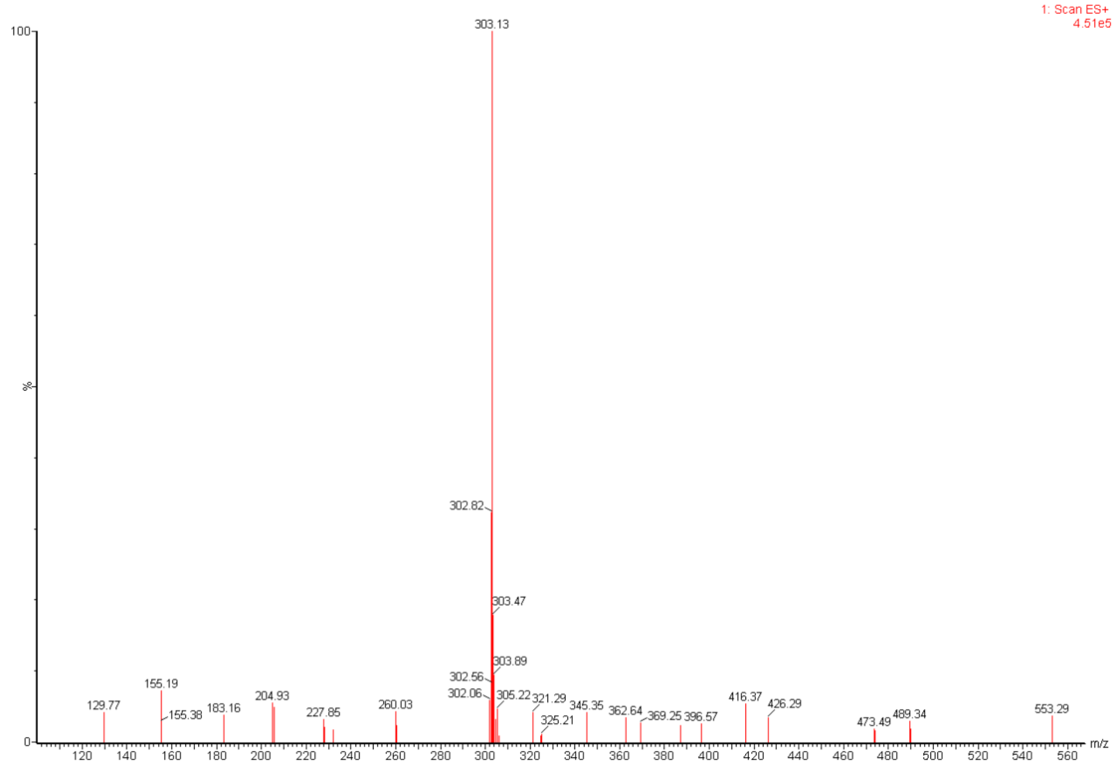

MS spectrum of compound H32

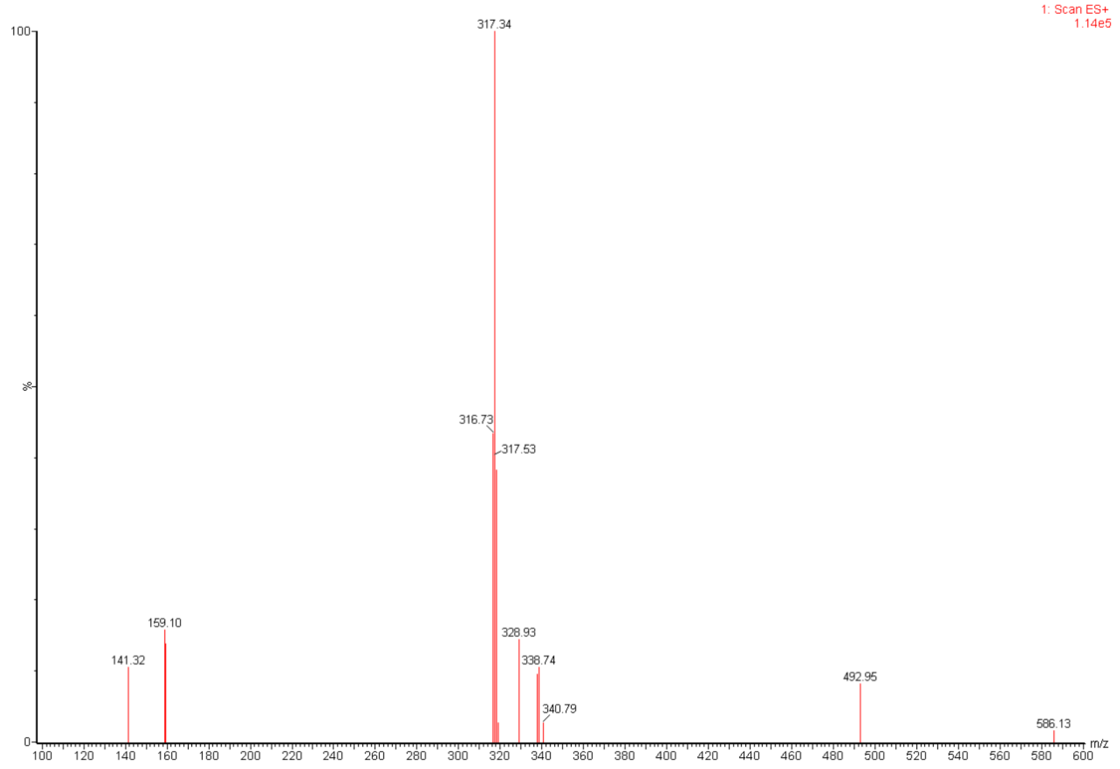

MS spectrum of compound H33

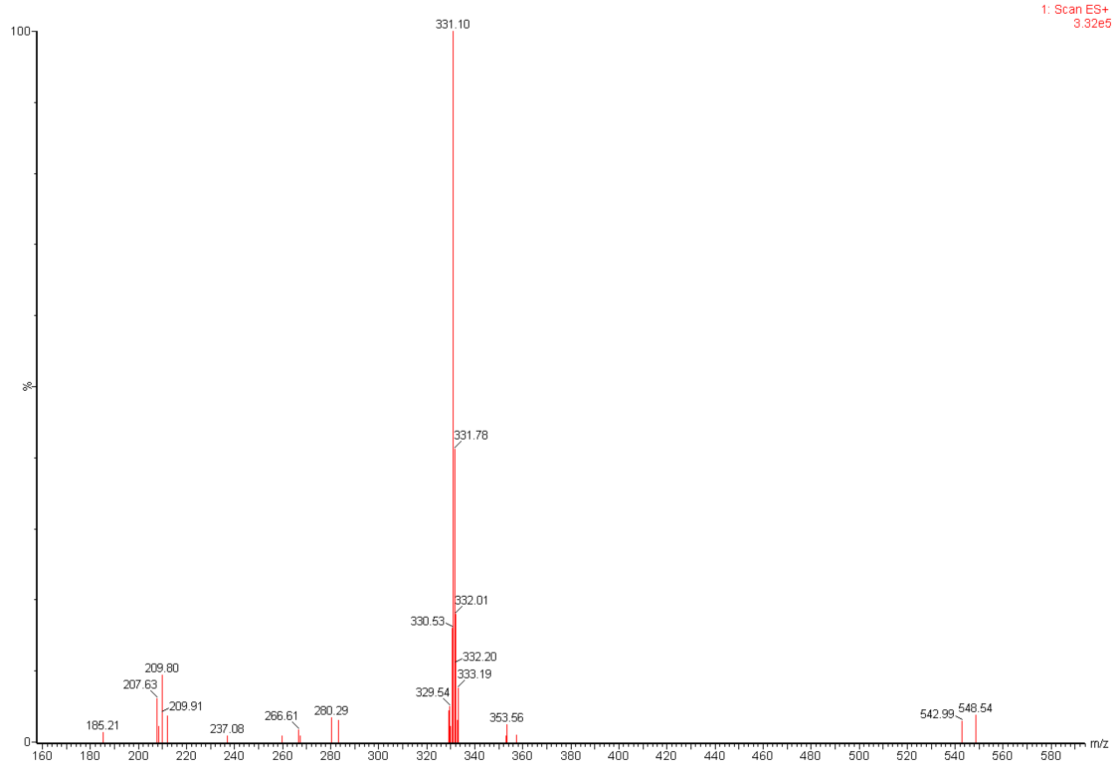

MS spectrum of compound H34

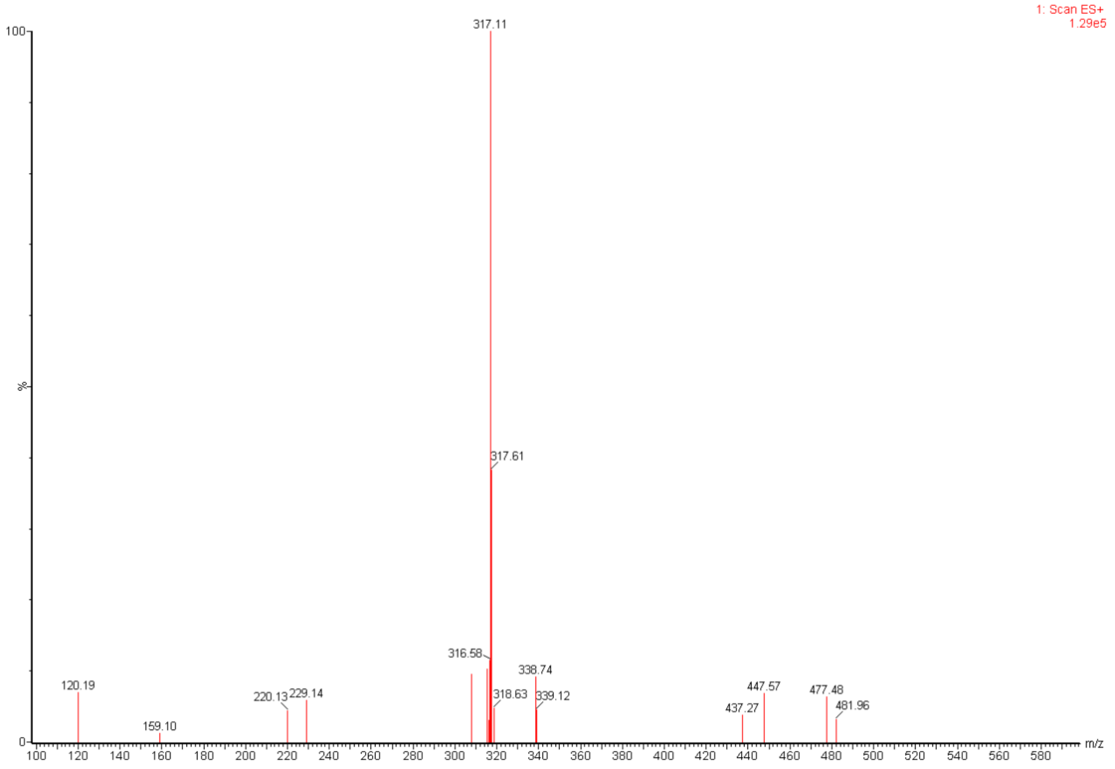

MS spectrum of compound H35

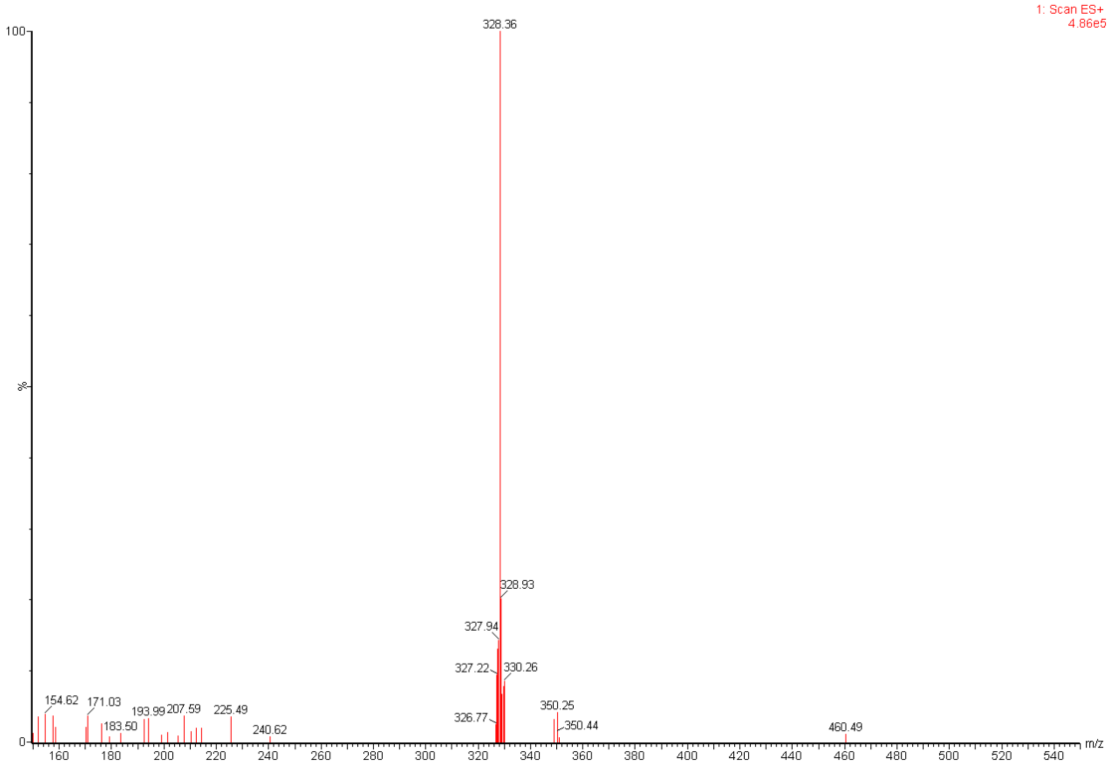

MS spectrum of compound H36

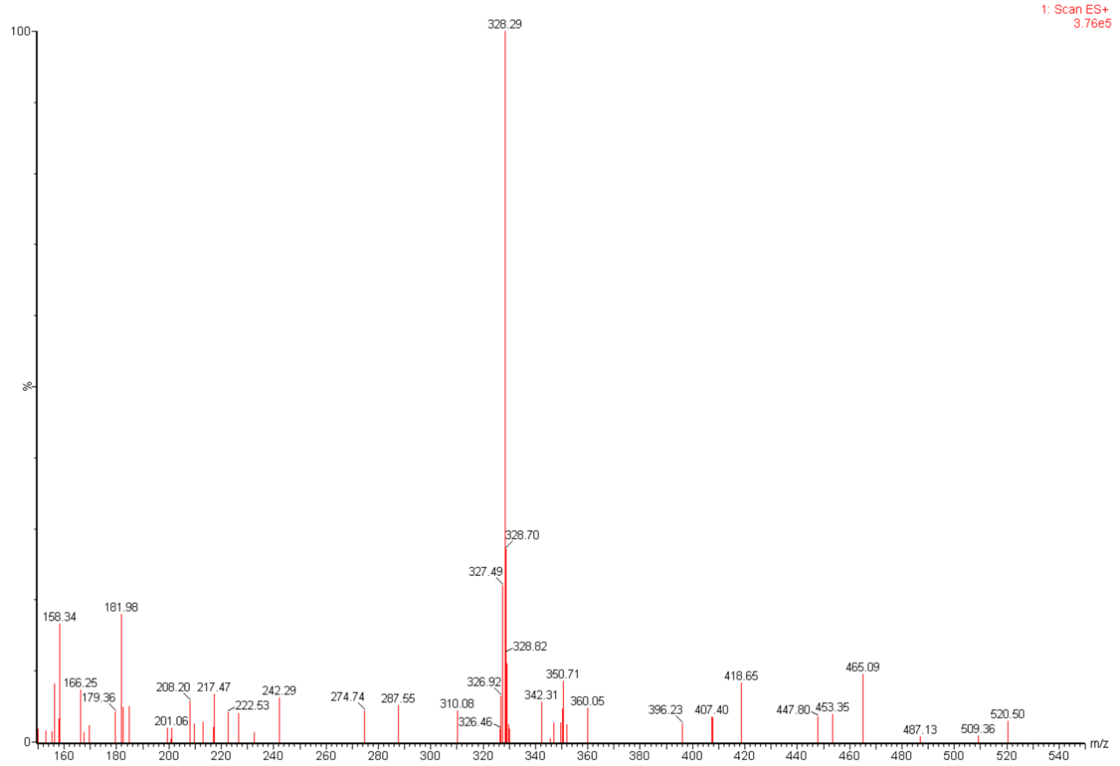

MS spectrum of compound H37

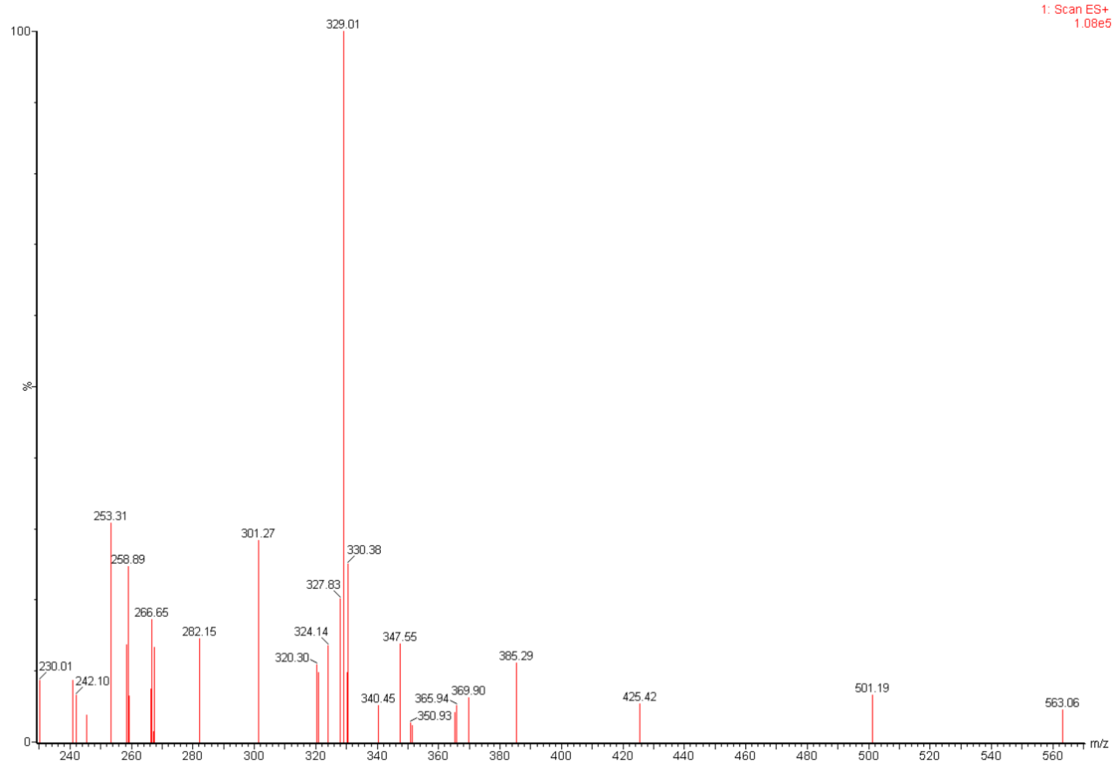

MS spectrum of compound H38

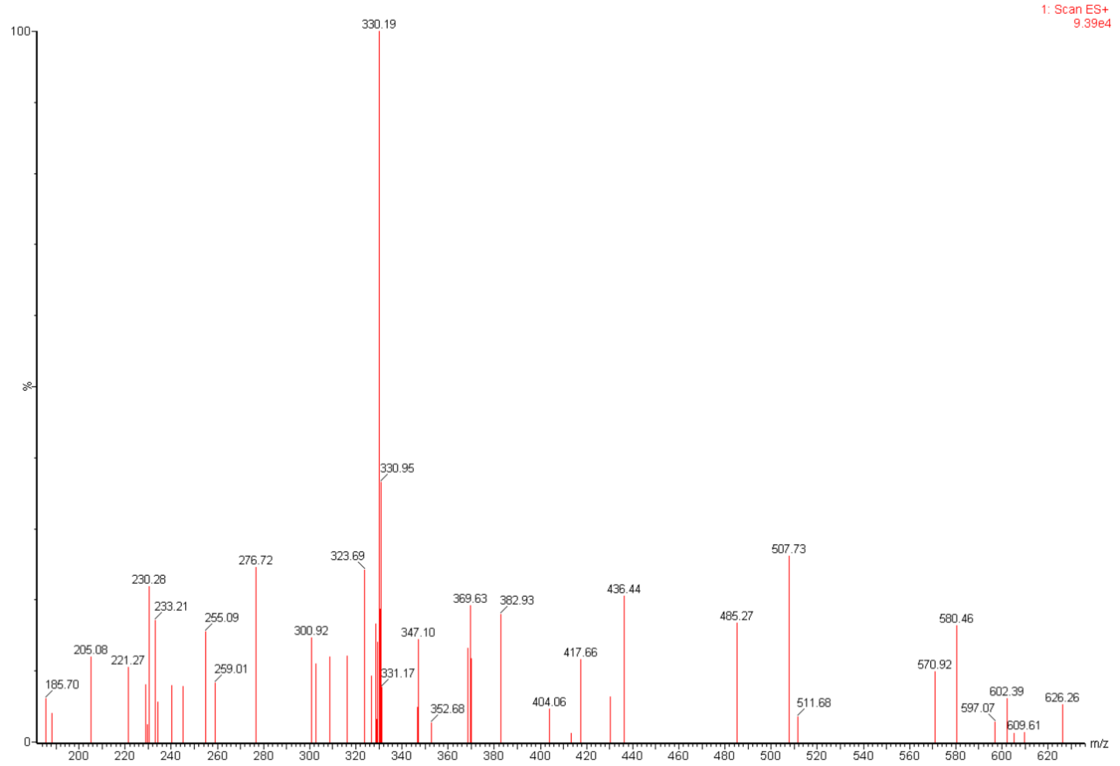

MS spectrum of compound H39

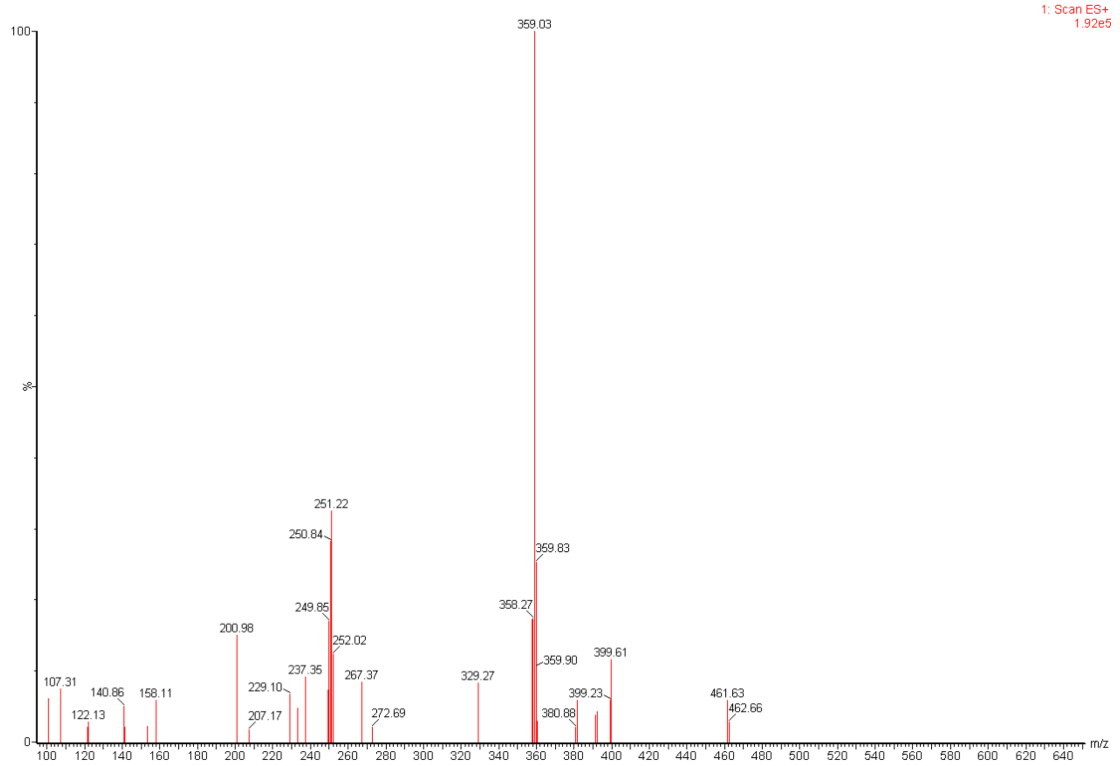

MS spectrum of compound H40

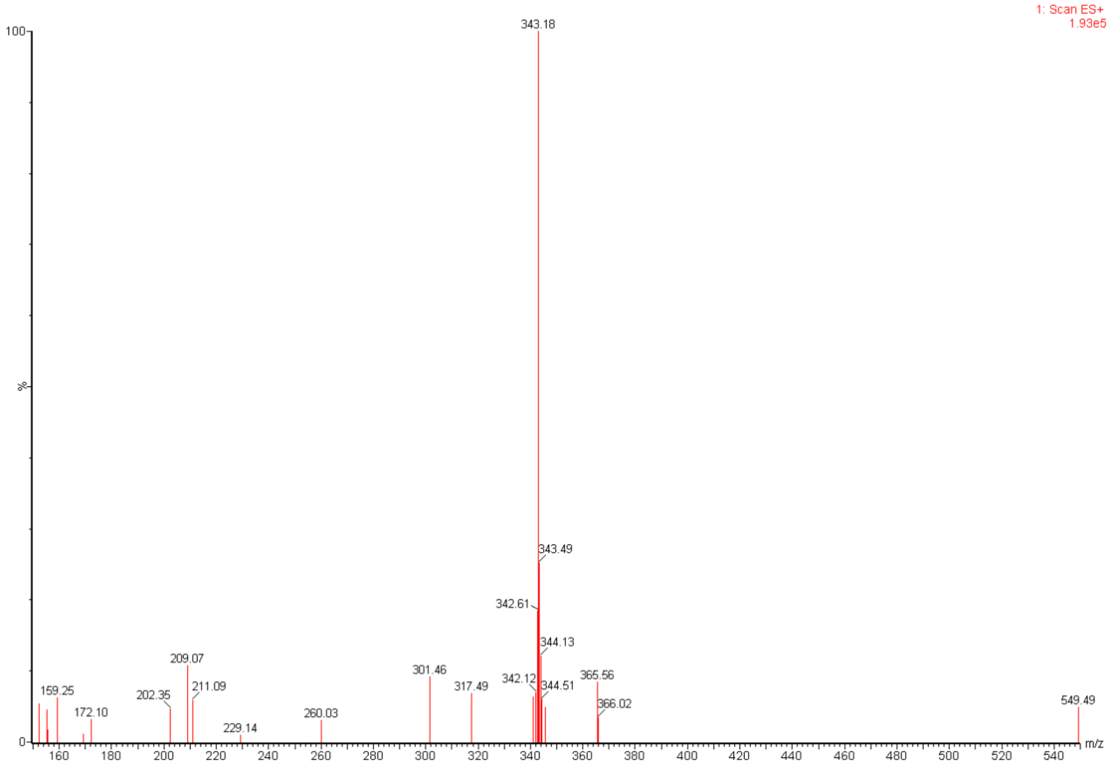

MS spectrum of compound H41

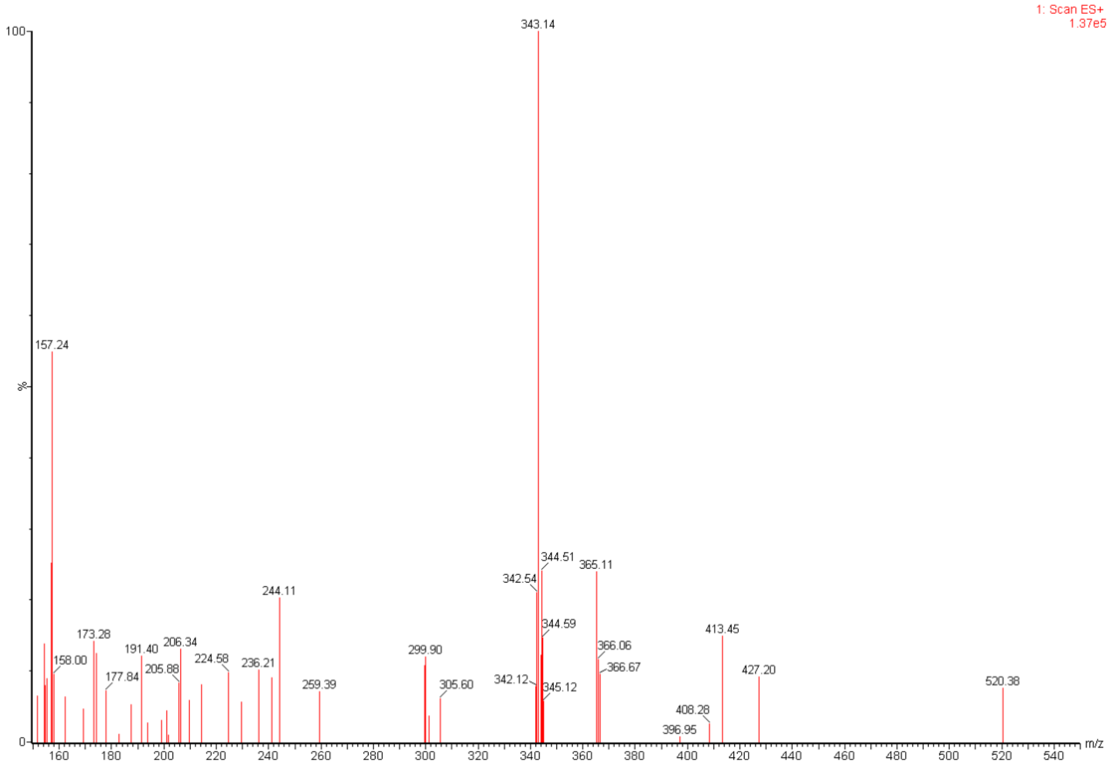

MS spectrum of compound H42

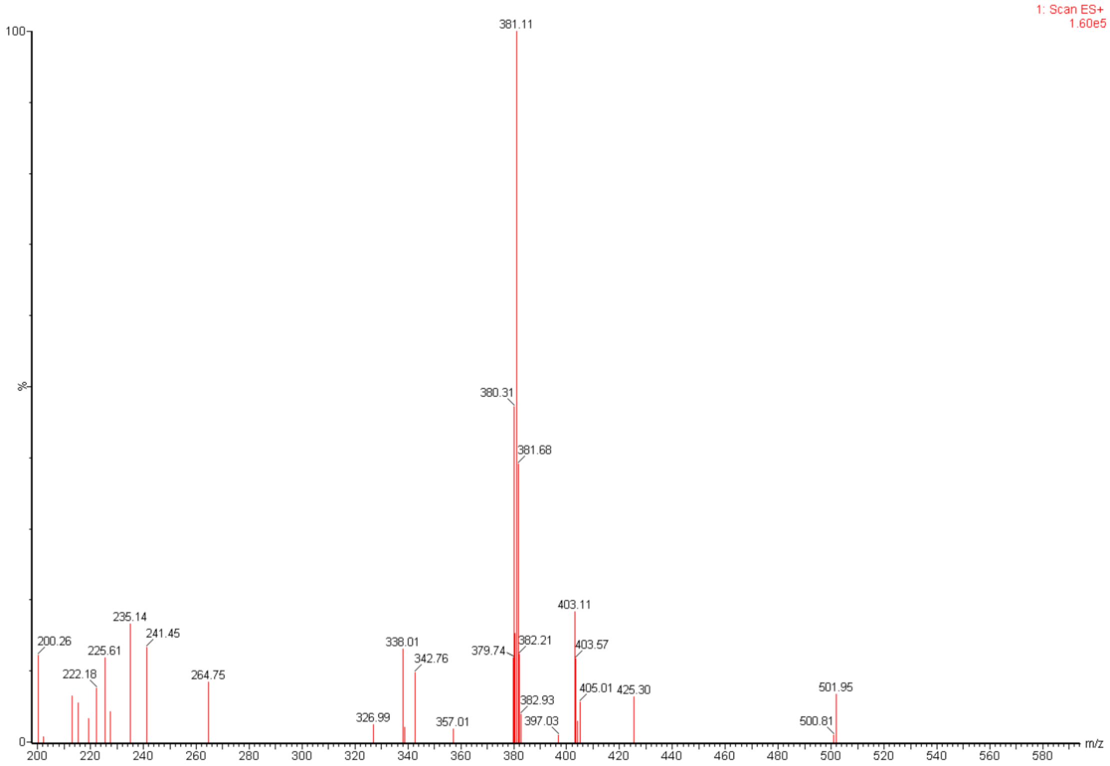

MS spectrum of compound H43

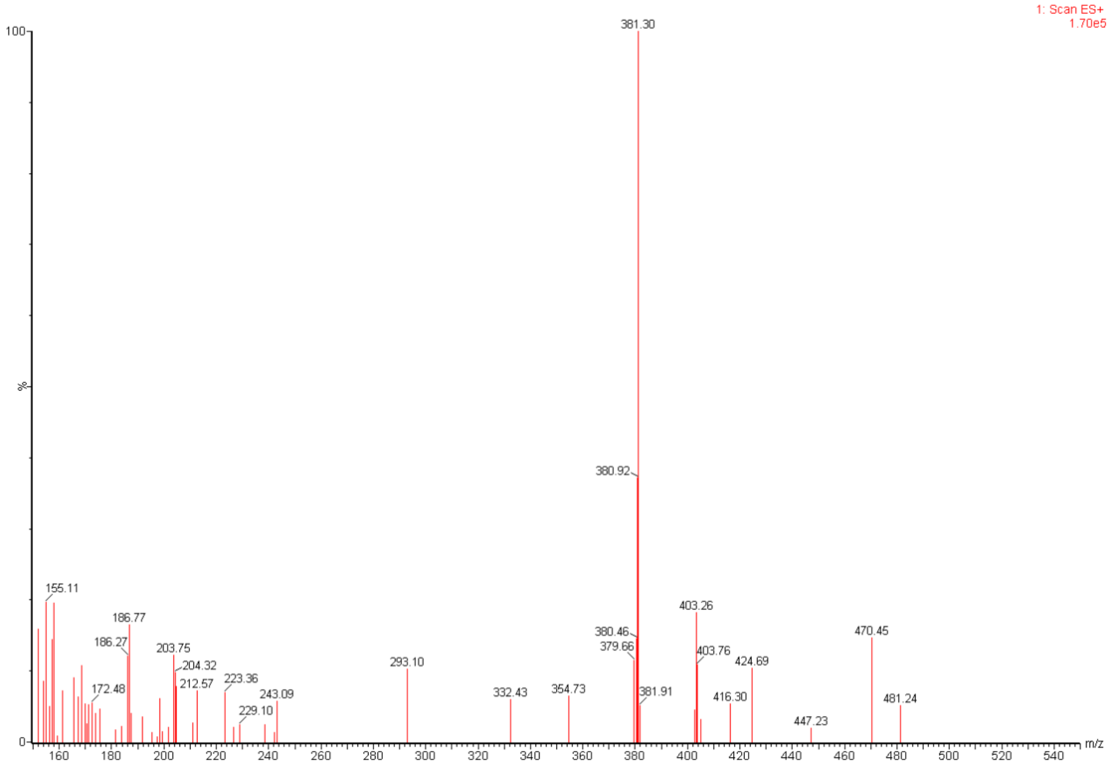

MS spectrum of compound H44

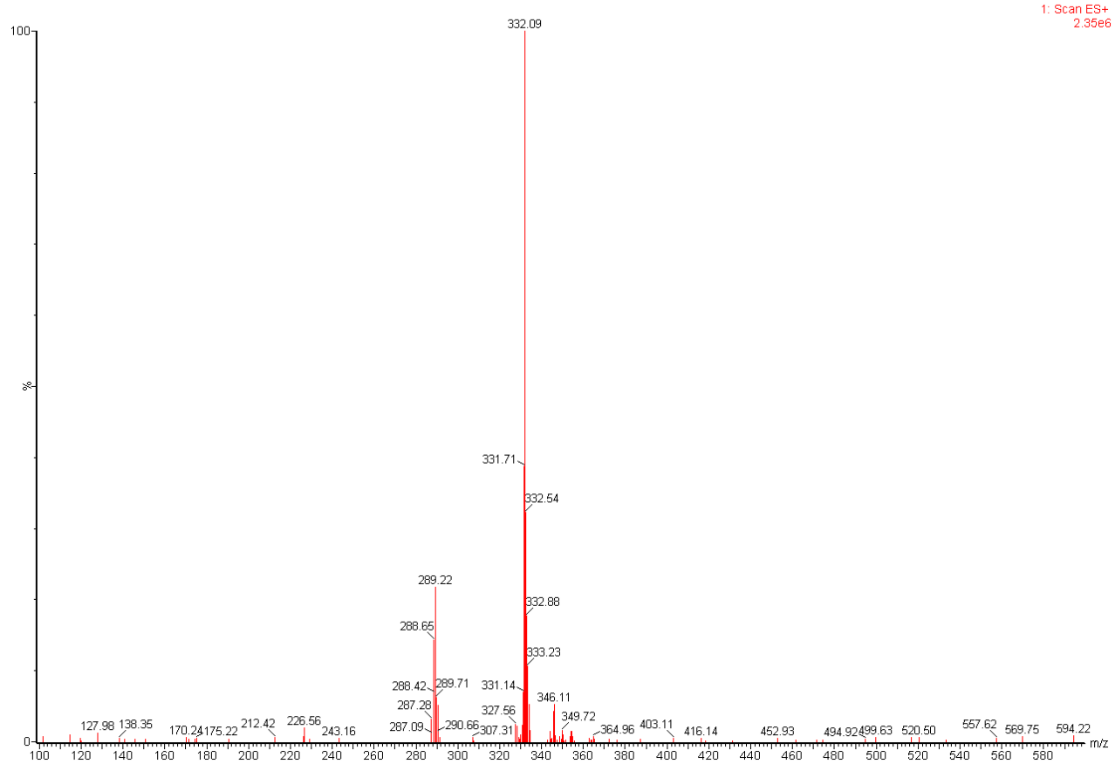

MS spectrum of compound H45

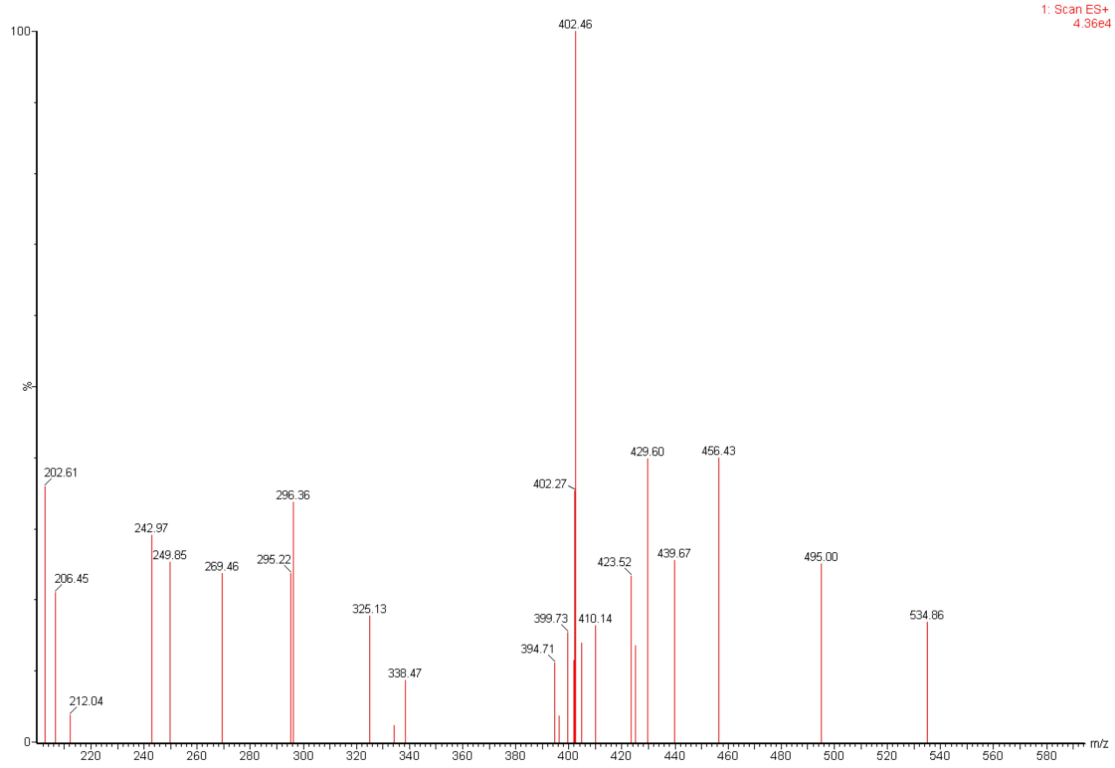

MS spectrum of compound H46

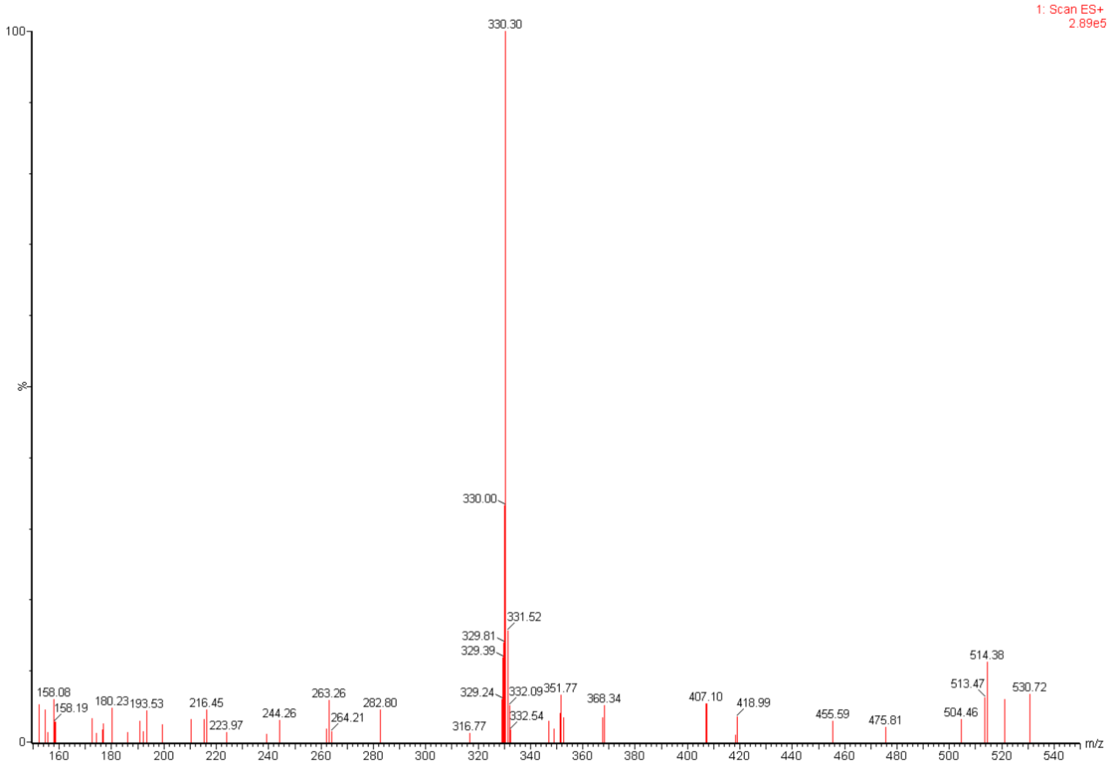

MS spectrum of compound H47

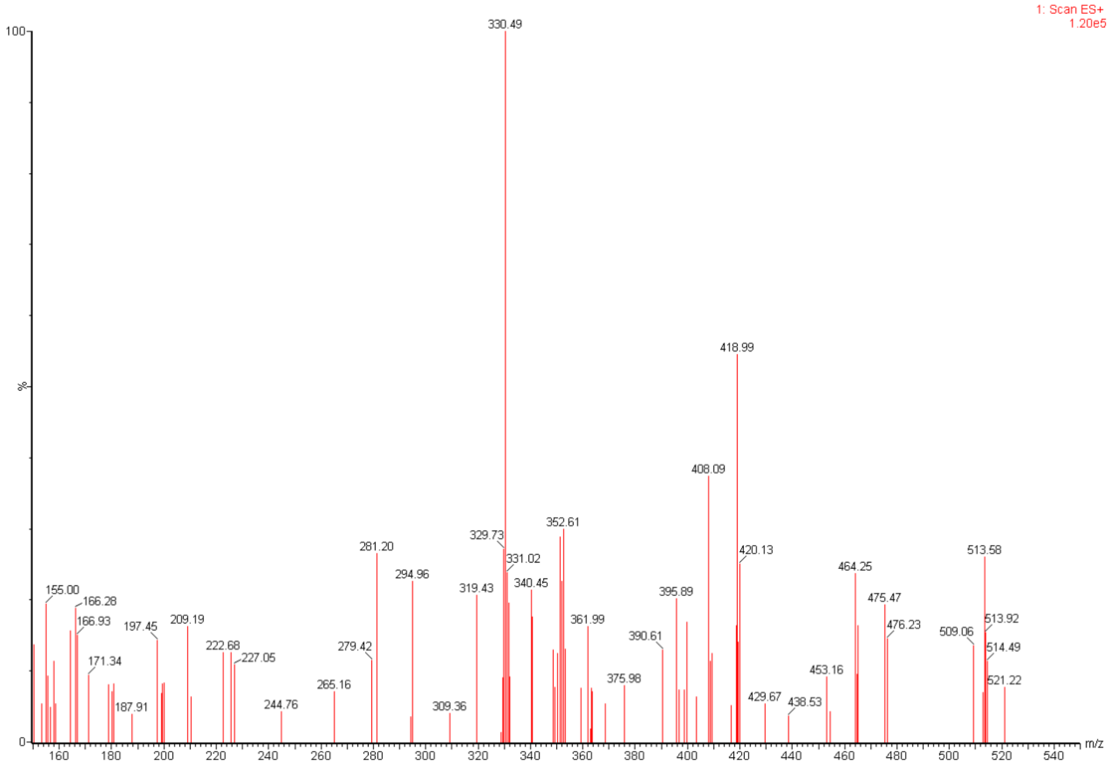

MS spectrum of compound H48

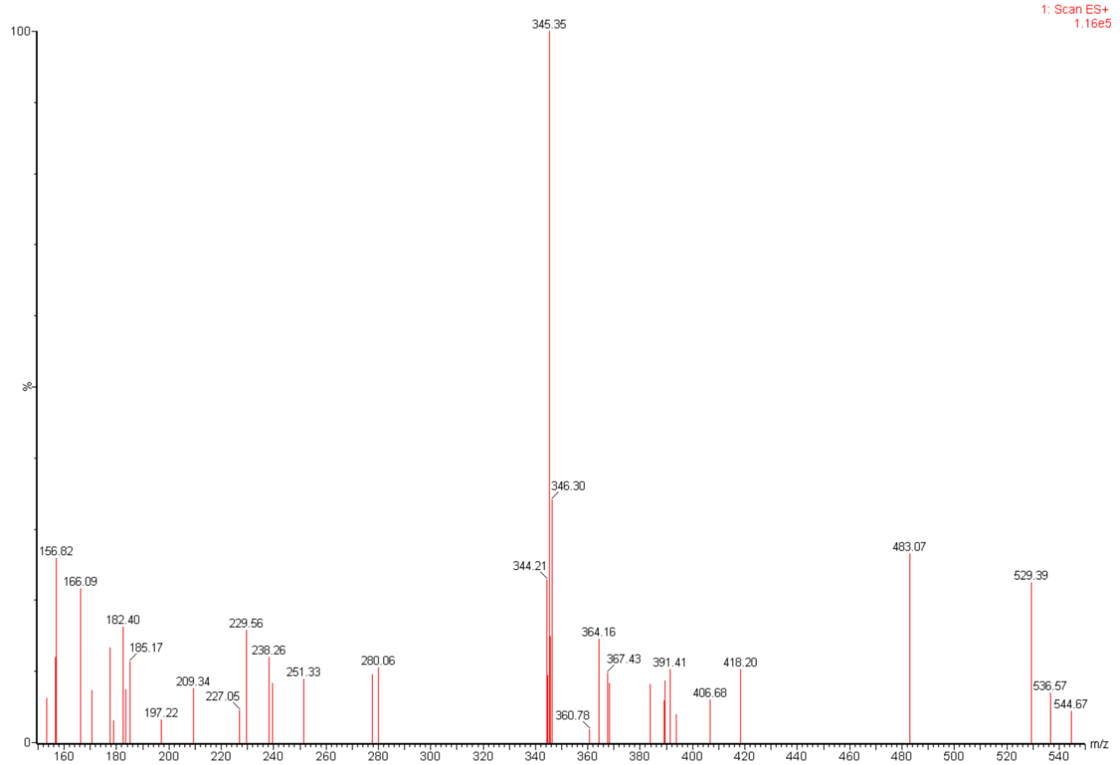

MS spectrum of compound H49

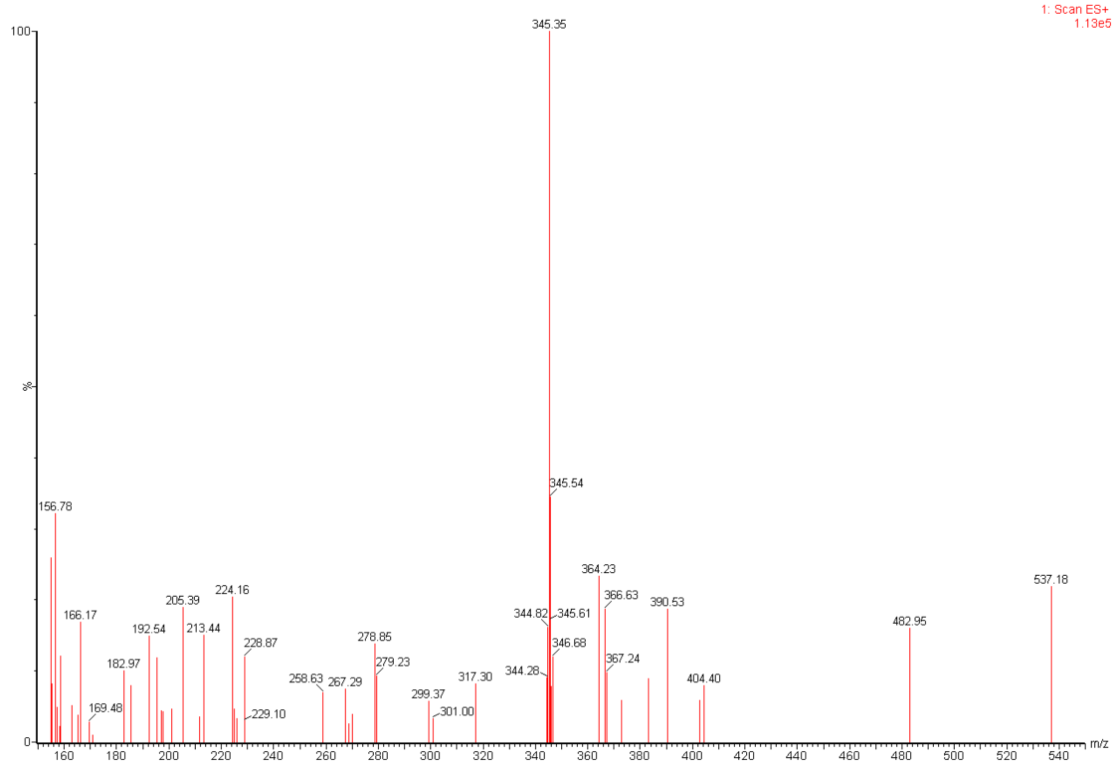

MS spectrum of compound H50

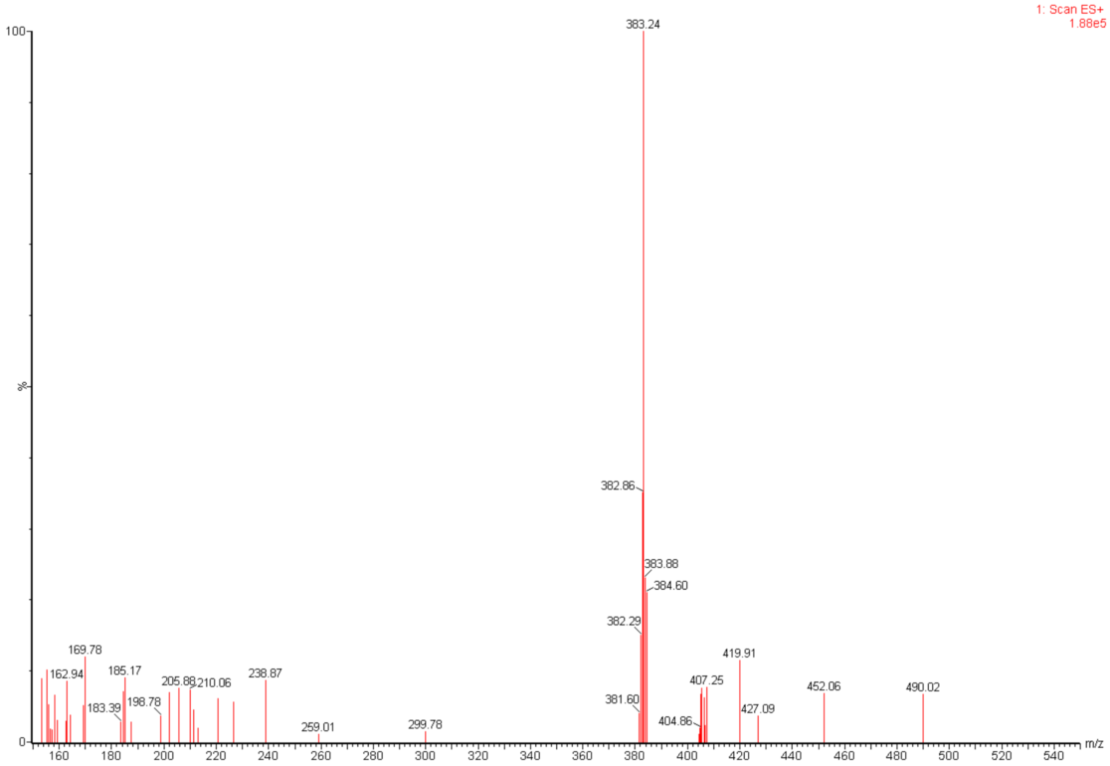

MS spectrum of compound H51

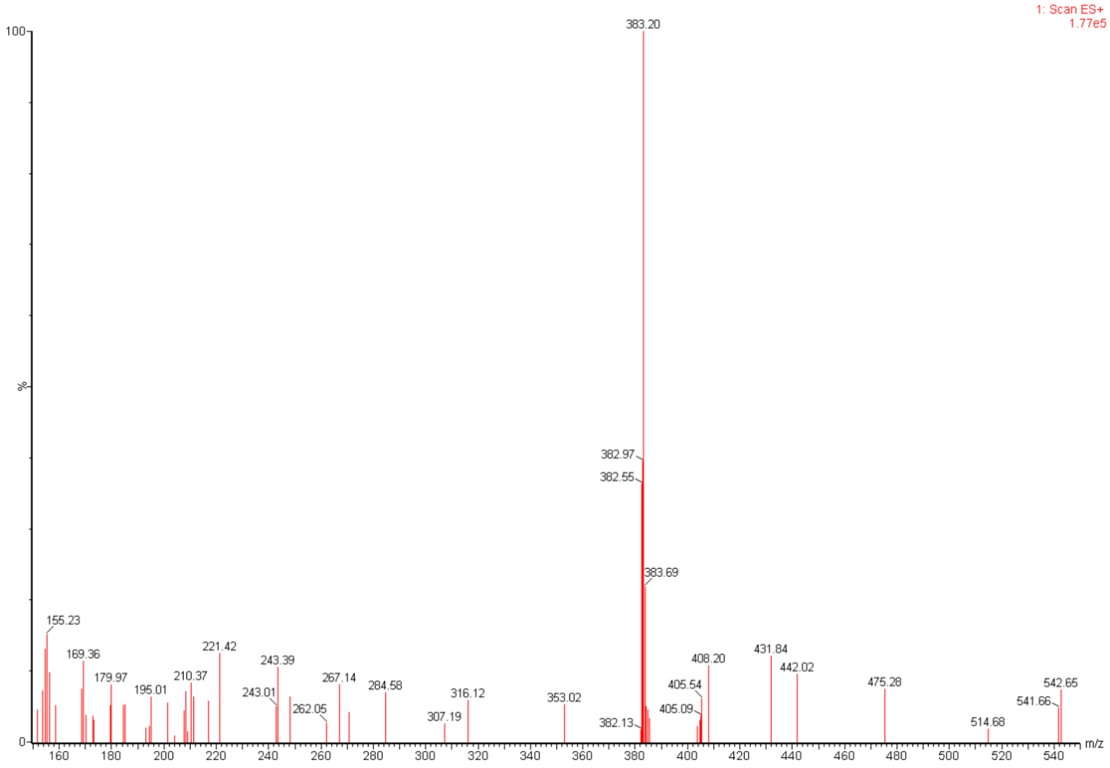

MS spectrum of compound H52

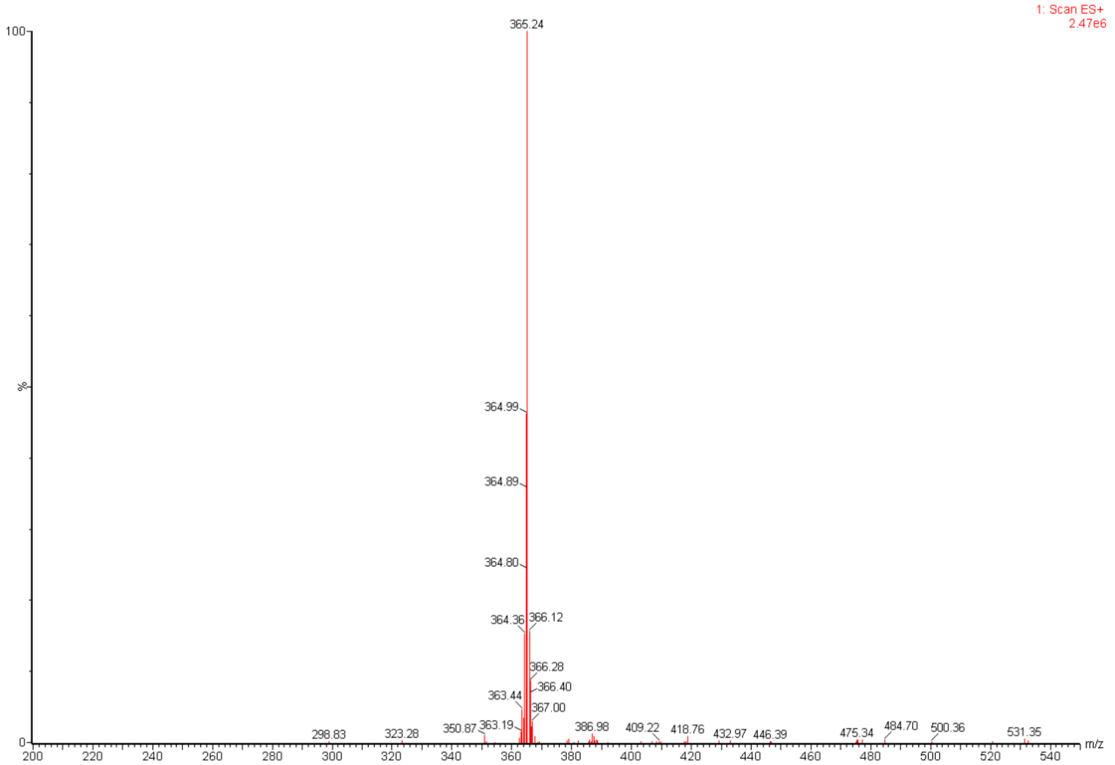

MS spectrum of compound H53

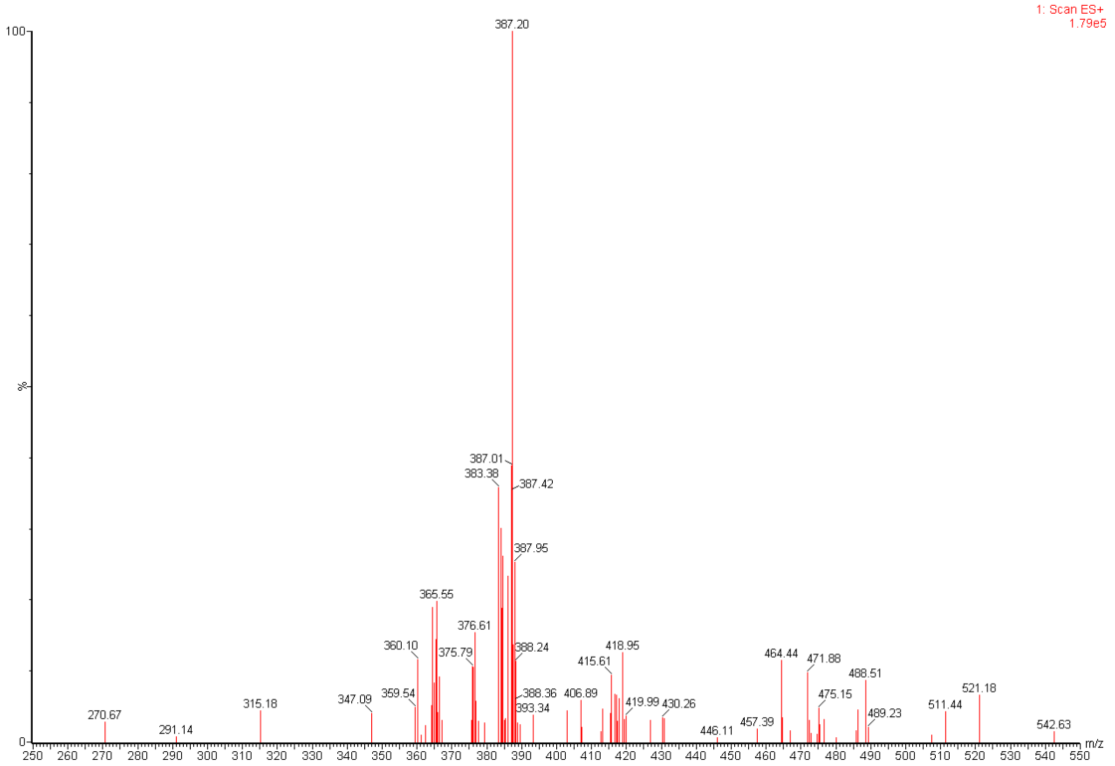

MS spectrum of compound H54

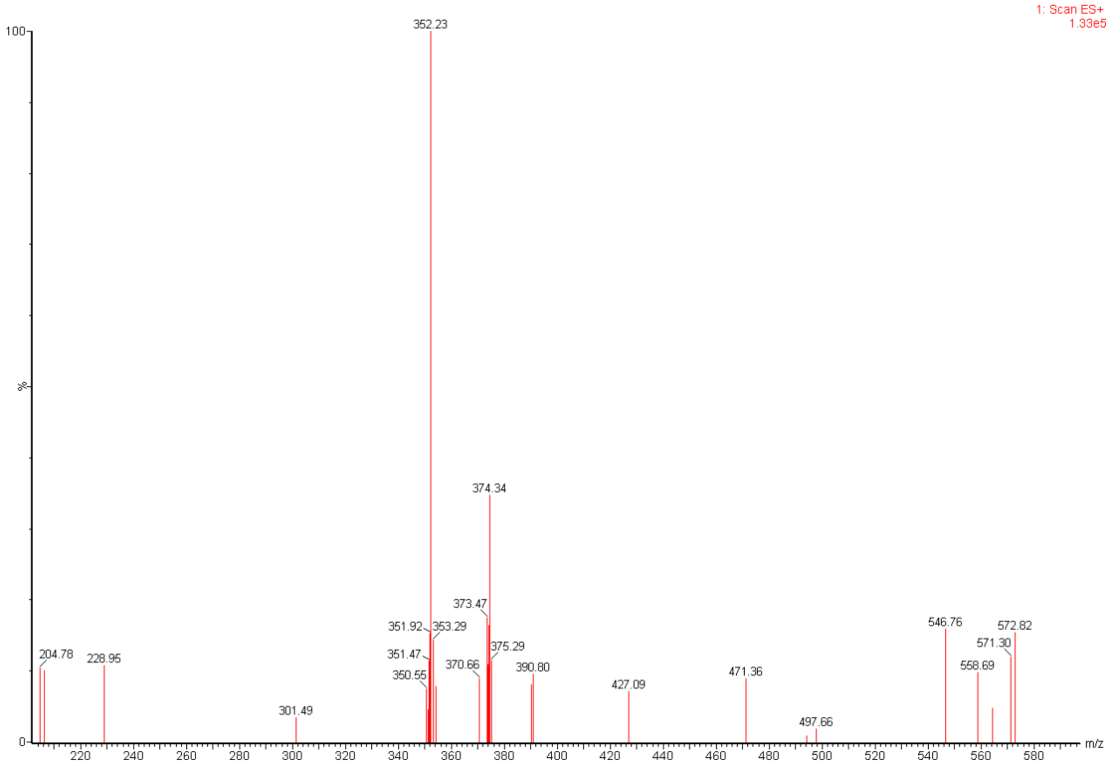

MS spectrum of compound H55
